# Phenotypic risk factors linked to acute radiation-induced toxicities: a phenome-wide Mendelian randomization study of 12,042 cancer patients

**DOI:** 10.7150/jca.127072

**Published:** 2026-04-23

**Authors:** Wenyi Jiang, Bo Li, Siyuan Zhang, Xin Sui, Yueting Liang, Minqi Jia, Shaoran Jiang, Lijuan Li, Huajing Teng, Weihu Wang

**Affiliations:** Department of Radiation Oncology, Key Laboratory of Carcinogenesis and Translational Research (Ministry of Education/Beijing), Peking University Cancer Hospital & Institute, Beijing 100142, China.

**Keywords:** MR-PheWAS, risk factors, acute radiation-induced toxicities, real-world validation

## Abstract

**Objective:**

This study aims to systematically evaluate phenome-wide clinical factors contributing to radiation-induced toxicities (RITs) and provide evidence to identify acute RITs-associated risk factors for personalized radiation treatment stratification.

**Methods:**

Leveraging genome-wide association study data from 12,042 patients with prostate, head and neck, breast, or lung cancer, we conducted MR-PheWAS (phenome-wide association study integrated with Mendelian randomization) to evaluate the impact of 22,872 phenotypic traits on the susceptibility to acute RITs.

**Results:**

MR-PheWAS pinpointed diverse acute RITs-associated phenotypic traits, including previously noted and novel ones. Proteins related to infection and immunity, like C-reactive protein and Interleukin-4 receptor alpha, were found to augment the risk of acute RITs, whereas growth differentiation factor 15, fibroblast growth factor-2 and Interleukin-16 seemingly reduce it. Notably, systolic/diastolic blood pressure, and lipoprotein or cholesterol levels could elevate the risk, but fatty acids, lipids and body mass index-adjusted leptin levels offered protection. Real-world validation in 1,078 breast cancer patients who underwent radiotherapy in our department showed that red blood cell count (lowering risk), serum urea and uric acid levels (increasing risk) before radiotherapy were linked to acute RITs. Moreover, we estimated the direct causal link between the associated traits and acute RITs through multivariate MR analyses, and unveiled that systemic lupus erythematosus, and high-density lipoprotein cholesterol levels remained significantly associated with acute RITs.

**Conclusion:**

This study identified the phenome-wide risk factors linked to acute RITs, and future research was needed to clarify their underlying mechanisms for effective prevention of acute RITs.

## Introduction

As an effective treatment modality for localized solid cancers, radiation therapy has been received by approximately 60% of all cancer patients for curative or palliative purposes^1^. Among them, some tolerate treatments well, while some suffer from severe radiation-induced toxicities (RITs) in the normal tissues, which might occur during or within 3 months (acute) or after 3 months (late) following radiation therapy^2^-^4^. Severe acute toxicities are liable to trigger dose reduction, treatment postponement or even termination, thereby compromising the therapeutic efficacy. Thus, it's critical to explore a deeper understanding of the risk factors related to acute RITs.

Apart from the dose distributions, evidence of patients' factors contributing to RITs has been provided by numerous reports, such as genetic susceptibility, comorbidities and lifestyles^5^-^7^. Genome-wide association study (GWAS) has been extensively employed to characterize genetic susceptibility variants associated with those RITs. Most of these GWASs focus on genetic variants associated with late RITs in a given tissue type^8^-^14^, and a few studies involve acute RITs^15^-^17^. Common susceptibility to acute RITs across tissues may exist given that it depends on DNA damage and cell death^16^, and a recent study reveals common genetic variations linked to acute RITs across four cancer types^18^. Numerous clinical factors, including age, body mass index (BMI), smoking behavior, hemoglobin levels, comorbidities such as diabetes mellitus and inflammatory bowel disease, have also been identified as potential risk factors for RITs following radiation therapy^19^-^23^. However, it is important to emphasize that the reported associations between clinical factors (e.g., BMI, age, and hemoglobin levels) and RITs are observational in nature. Such correlations may be confounded by underlying patient characteristics or influenced by reverse causation, and thus do not necessarily indicate a direct causal relationship. Therefore, whether these factors contribute causally to the occurrence of RITs remains unclear, and a systematic evaluation of the causal roles of phenome-wide clinical factors in RITs has yet to be conducted.

Randomized controlled trials (RCT) have been regarded as the gold standard for discerning causal relationships, but time constraints or ethical issues often make them impractical or even infeasible. As a genetic version of the RCT, Mendelian randomization (MR) utilizes genetic variants as instrumental variables to deduce causal associations between exposures and outcomes, overcoming key limitations of observational studies^24^, ^25^. By capitalizing on the stochastic assignment of genetic variants during conception, MR reduces confounding factors and circumvents reverse causation, as germline DNA remains unaffected by the presence of disease. The phenome-wide association study integrated with MR (MR-PheWAS) extends the approach of MR by systematically screening associations across a wide range of phenotypes, enabling hypothesis-free discovery of novel causal links^26^. Here, we carry out a large, cross-ancestry (including European, East Asian, and Hispanic/Latin American populations), MR-PheWAS, to elucidate prior unexplored risk factors linked to acute RITs, and provide novel evidence for identifying which acute RITs-associated risk factors might be used to stratify patients for personalized radiation treatment.

## Materials and Methods

### Study design

**Figure 1:**
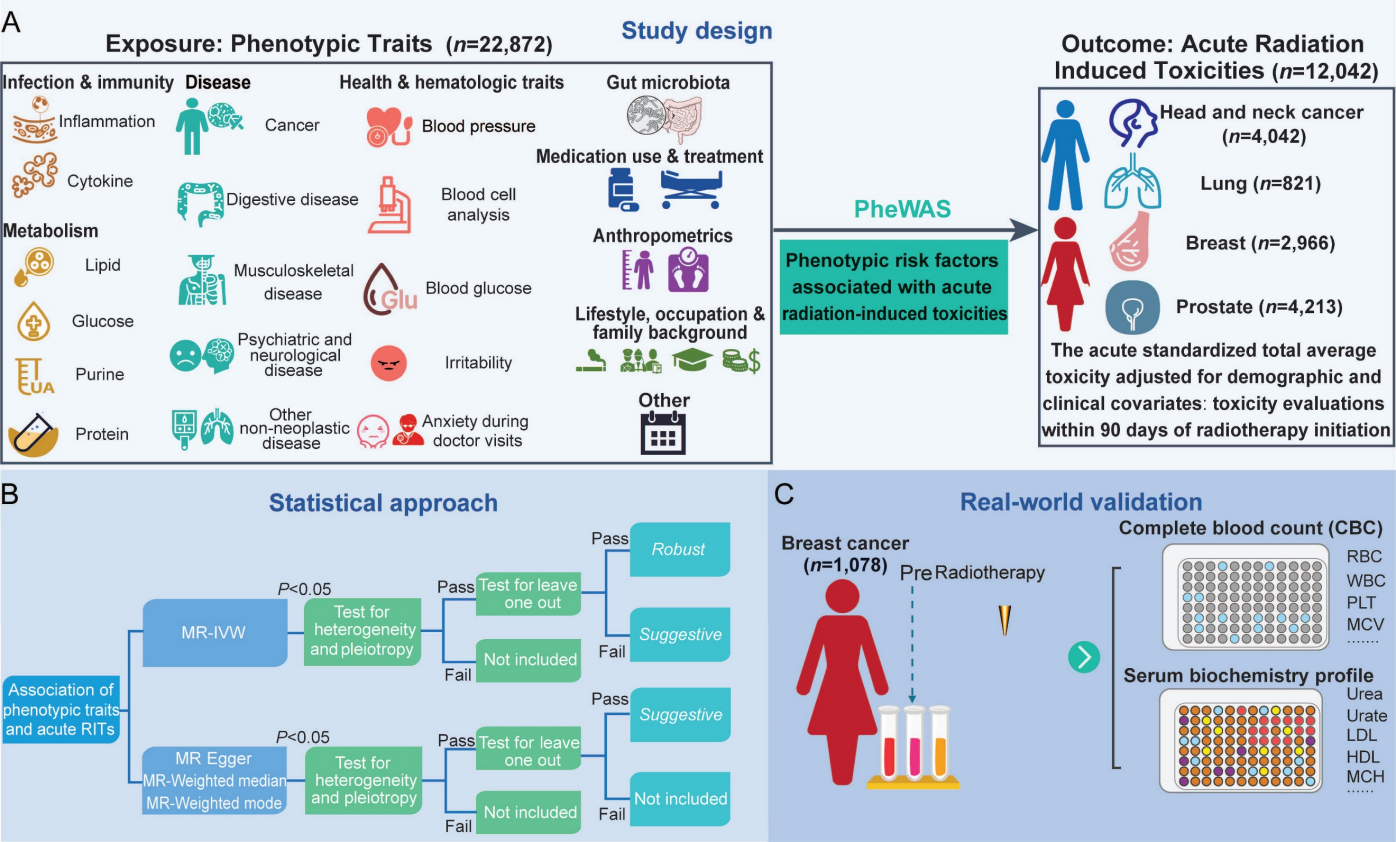
** Study design A.** Exposures and outcomes for MR-PheWAS association analysis. **B.** Hierarchical classification of associations. **C.** Association validation in Complete blood count (CBC) and serum biochemistry profile data collected from patients with breast cancer. RITs, radiation-induced toxicities; MR, Mendelian randomization; IVW, inverse-variance weighted; RBC, red blood cell; WBC, white blood cell; MCV, mean corpuscular volume; PLT, platelet; HDL, high density lipoprotein; LDL, low density lipoprotein; MCH, mean corpuscular hemoglobin.

A diagrammatic overview of the study is presented in **Figure 1**. Firstly, we conducted a MR-PheWAS to get all available variables in the IEU OpenGWAS (https://gwas.mrcieu.ac.uk/). Secondly, to ensure the reliability of the identified causal links, we implemented a rigorous quality control pipeline using multiple MR models, including inverse-variance weighted (IVW), fixed-effect inverse-variance weighted (IVW_fe), weighted mode, weighted median, MR Egger and Wald ratio methods. Sensitivity analyses, including Cochran's Q test for heterogeneity and the MR-Egger intercept test for horizontal pleiotropy, were performed to detect and minimize potential biases. A leave-one-out analysis was also conducted to evaluate the robustness of causal estimates and to identify whether individual SNPs disproportionately influenced the overall result. Finally, we employed multivariable MR analysis (MVMR) to account for horizontal pleiotropy across correlated traits, thereby enabling the identification of independent causal effects.

Our research follows three assumption principles below^27^. Firstly, there should be a significant and strong correlation between genetic variations and exposure factors. In this study, we defined “strong correlation” by selecting instrumental variables (IVs) that reached genome-wide significance (*P*-value <1×10⁻^5^). Additionally, to assess instrument strength and minimize weak instrument bias, we computed the F-statistic for each exposure, and an F-statistic > 10 was considered indicative of a sufficiently strong instrument. Secondly, genetic variations should not have a direct causal relationship with the outcomes. Thirdly, genetic variants must not be connected to any confounding factors.

Additionally, to assess instrument strength and minimize weak instrument bias, we computed the F-statistic for each exposure; an F-statistic > 10 was considered indicative of a sufficiently strong instrument.

To further validate the association of the identified hematologic or metabolic risk factors with acute RITs, we collected complete blood count (CBC) and serum biochemistry profile data of breast cancer patients prior to radiotherapy in the Department of Radiation Oncology, Peking University Cancer Hospital, and analyzed their associations with the patients' acute radiation toxicity. The analysis was approved by the Ethics Committee of Peking University Cancer Hospital.

### Outcome data collection

The summary-level data on acute RITs (GCST90435417) was downloaded from the GWAS Catalog (https://www.ebi.ac.uk/gwas/). This data was obtained from a study that was conducted spanning across different tissues among 19 cohorts with a total of 12,042 patients from head and neck cancer, lung cancer, prostate cancer, and breast cancer^18^. These cohorts were all from the Radiogenomics Consortium (https://epi.grants.cancer.gov/radiogenomics/). Patients received prescribed doses for radiation therapy according to specific clinical guidelines, and were tracked for the development of acute RITs. The acute standardized total average toxicity adjusted for demographic and clinical covariates (rSTAT_acute_) was computed using the toxicity evaluations amassed over a period of 90 days starting from the commencement of radiation therapy.

### Exposure data collection

We obtained GWAS data of 50,045 traits (repeated traits included) from the IEU Open GWAS database via the “TwoSampleMR” and “ieugwasr” package in R (version 4.4.2). We conducted our analysis by including all GWAS datasets, with the exception of those associated with imaging or NA phenotypes, those with a sample size of less than 3000, or those derived from the eQTL database.

### Instrumental selection

Before conducting MR-PheWAS, we implemented quality control to determine suitable instrumental variables. The study incorporated single-nucleotide polymorphisms (SNPs) with effect allele frequency >0.01 and association *P*-value <1×10⁻^5^ as previously reported^28^. Moreover, SNPs that were correlated at a linkage disequilibrium of *r²* > 0.001 within 10,000 kb were excluded, and only those with the most potent effect were selected. This clumping procedure was performed to ensure the independence of the instrumental variables, thereby preventing over-representation of any single genetic signal and reducing bias in causal estimates due to multi-collinearity. Subsequently, we retrieved SNPs from outcome data for harmonization.

### Mendelian randomization analyses

In the two-sample MR analyses, six methods, including IVW, IVW_fe, weighted mode, weighted median, MR Egger and Wald ratio, were utilized. The selection of the method was customized according to the traits being investigated and the number of genetic instruments.

When the traits were affected by multiple SNPs acting as instrumental variables, the IVW and IVW_fe method was mainly employed^29^. Moreover, the IVW_fe method was preferentially used. When heterogeneity occurred among the instrumental variables, the IVW was utilized as a substitute for the IVW_fe method. For traits with only one SNP, the Wald ratio method was used.

Given that the results of IVW may introduce bias if certain SNPs exhibited horizontal pleiotropy, three additional MR methods were incorporated to enhance the robustness. The MR-Egger approach, using the gradient estimate from Egger regression to calculate causal effects, offers a reliable estimate even when the instrumental variables are invalid^30^. The weighted median approach can counteract up to 50% of invalid instrumental variables. The weighted mode approach is suitable when the relaxed assumption of instrumental variables results in reduced bias and a lower type I error rate^31^. All analyses were carried out using the package TwoSampleMR^32^ in R (version 4.4.2).

### Sensitivity analyses and hierarchical classification of associations

To evaluate heterogeneity in instrument effects, which may indicate potential violations of the instrumental variable assumptions underlying two-sample MR^33^, we used Cochran's Q test and determined heterogeneity with a *P*≤0.05. Next, a *P*≤0.05 was used to identify potential horizontal pleiotropy by using the “mr_pleiotropy_test () function” in the TwoSampleMR package. To test the compliance with the exclusion restriction assumption, we carried out the leave-one-out approach (Additional file3). This was done to identify potential pleiotropy for each SNP. Subsequently, we assessed the results both before and after removing the SNP. In addition, to cope with the diverse set of traits that had been analyzed, we set up a tiered categorization system for evaluating putative causal links between phenotypic traits and RITs (**Figure 1A**). *Robust* associations are defined as those demonstrating statistical significance in both IVW (including IVW_fe) analyses and multiple sensitivity analyses. *Suggestive* traits, which are significant at *P*<0.05, do not succeed in passing the leave-one-out test despite having passed the other two tests or rely on additional MR methods.

To facilitate interpretation, we categorized traits, which were significant at *P*<0.05 and passed the test of heterogeneity and horizontal pleiotropy analysis, into the following groups similar to Markozannes *et al.*^34^: Anthropometrics, Health and hematologic traits, Gut microbiota, Infection and immunity, Lifestyle, occupation and family background, Disease, Metabolism, Medication use and treatment, and Other.

### Association validation of hematologic/metabolic risk factors with acute RITs in breast cancer patients

Complete blood count (CBC) and serum biochemistry profile data of 1,078 female breast cancer patients who received radiotherapy in the Department of Radiation Oncology from the January 2023 to June 2024 were retrieved from the hospital information system (HIS) of Peking University Cancer Hospital. The median age of these patients was 50 years old ranging from 21 to 79, and 96% of them had undergone postoperative adjuvant radiotherapy. Approximately 50% of patients received hypofractionated radiotherapy of 40 Gy in 15 fractions, and the remaining 50% underwent whole-breast irradiation of 50 Gy with a simultaneous integrated boost of 60 Gy in 25 fractions to the tumor bed. Radiation plans were generated using either intensity-modulated radiation therapy (IMRT) or volumetric-modulated arc therapy (VMAT), selected by medical physicists to ensure 95% of the planning target volume (PTV) was covered by the prescribed dose, with optimal organ-at-risk sparing and homogeneous dose distribution. Acute radiation skin toxicity was graded according to the Common Terminology Criteria for Adverse Events (CTCAE) v5.0, based on clinical assessments documented in the EMR system. Patients were evaluated at baseline (prior to treatment initiation), weekly during radiotherapy, and at treatment completion between January 2023 and June 2024. To ensure objectivity, two radiation oncologists independently graded toxicity in a blinded, retrospective manner, with no access to hematologic or metabolic laboratory results to minimize observer bias. Discrepancies were resolved by consensus with a third senior radiation oncologist. Patients were stratified into two groups based on their acute radiation toxicity grade (< 2 degree vs. ≥ 2 degree). Difference in abundance of each hematologic/metabolic indicator prior (two weeks to one day) to the radiotherapy in the two groups was compared using the Mann-Whitney test, and a *P*<0.05 was considered statistically significant.

### Analysis of multivariable Mendelian randomization

The MVMR can be utilized for multiple genetic instruments without considering their association with the exposure when certain assumptions are satisfied^35^-^38^. We employed it as a mediation analysis to explore the direct causal link between the associated traits and acute RITs in the *Robust* category. In this research, we carried out MVMR by using the “mv_residual () function” from the TwoSampleMR package in R.

## Results

### Data description and Mendelian randomization analysis

A total of 12,042 patients, with a median age over 60, from prostate, head and neck, breast, and lung cancer were recruited across 19 cohorts of the Radio-genomics Consortium. The acute standardized total average toxicity adjusted for demographic and clinical covariates of each patient was computed within 3 months following the commencement of radiotherapy. More detailed information could be obtained in the original study^18^. Then, the summary-level data on genetic variations linked to acute RITs (GCST90435417) was obtained and used as outcome data. The exposure data of 50,045 traits was obtained from the GWAS Catalog database, followed by removing those with imaging or NA phenotypes, sample sizes < 3000, or those derived from the eQTL database, and 22,872 traits were retained.

After MR analysis, we identified 2,093 traits (repeated traits included) taken for further phenome analysis. Following the heterogeneity and horizontal pleiotropy analysis, 990 traits in total were obtained and categorized into different groups (**Figure 1A** and Additional file1-**Table S1)**. The top 5 represented exposure categories were Infection and immunity (258), Metabolism (194), Disease (145), Lifestyle, occupation and family background (82), Health and hematologic traits (80). To classify the evidence levels, leave-one-out test was further applied. Finally, 181 traits were classified as *Robust*, 809 traits were categorized as *Suggestive*, and the rest were excluded for no evidence of association.

### Causal associations supported by *Robust* evidence

**Figure 2:**
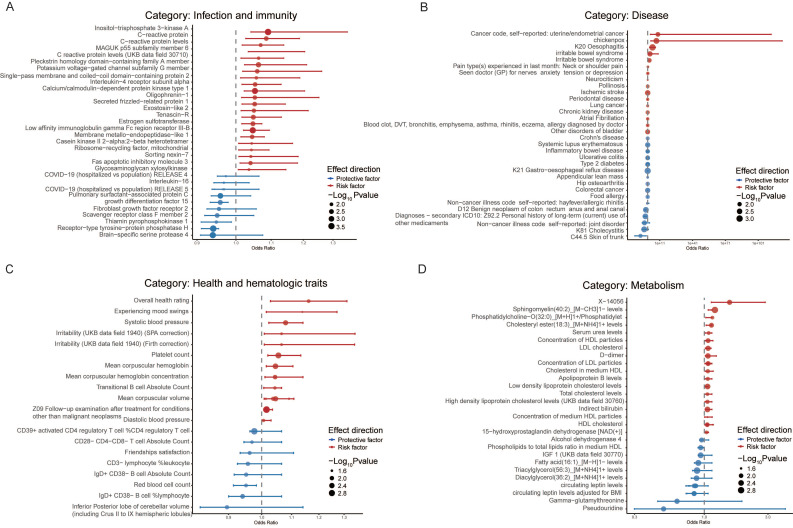
** Phenome-wide Mendelian randomization analysis of top 4 represented exposure categories for acute radiation-induced toxicities in the *Robust* category. A.** Analysis of Infection and immunity category**. B.** Analysis of Disease category. **C.** Health and hematologic traits category. **D.** Analysis of Metabolism category.

In the *Robust* category (**Figure 2 and** Additional file1-**Table S2**), the majority of traits were from the Disease (35), Infection and immunity (33), Metabolism (33), Health and hematologic traits (21), and Lifestyle, occupation and family background (17) groups. Specific protein of Infection and immunity (**Figure 2A**), such as C-reactive protein levels (CRP) (OR=1.08, 95% CI 1.02-1.13, *P*=0.01 in European populations; OR=1.09, 95% CI 1.03-1.15, *P*<0.01 in East Asian populations) and Interleukin-4 receptor subunit alpha (IL-4Rα) (OR=1.05, 95% CI 1.02-1.09, *P*<0.01), may cause an increased risk of acute RITs, whereas scavenger receptor class F member 2 (SCARF2) (OR=0.95, 95% CI 0.92-0.98, *P*<0.01), growth differentiation factor 15 (GDF15) (OR=0.96, 95% CI 0.93-0.98, *P*<0.01), fibroblast growth factor receptor 2 (FGF-2) (OR=0.95, 95% CI 0.92-0.99, *P*<0.01) and Interleukin-16 (IL-16) (OR=0.97, 95% CI 0.94-0.99, *P*=0.02) may bring benefits with a decreased risk.

Within the category of Disease (**Figure 2B**), ischemic stroke (OR=1.06, 95% CI 1.02-1.11, *P*<0.01) increased the risk of acute RITs in the East Asian population, and chronic kidney disease (OR=1.04, 95% CI 1.01-1.06, *P*=0.01) was associated with an elevated risk of acute RITs among Hispanic or Latin American people. Furthermore, strong correlations were discovered between acute RITs and diseases (European) such as chickenpox (OR=9.88×10^7^, 95% CI 8.04×10^2^-1.21×10^13^, *P*<0.01), oesophagitis (OR=2.75×10^4^, 95% CI 63.75-1.18×10^7^, *P*<0.01), irritable bowel syndrome (OR=1.23×10^2^, 95% CI 4.57-3.30×10^3^, *P*<0.01), pain type(s) experienced in last month: neck or shoulder pain (OR=1.71, 95% CI 1.07-2.73, *P*=0.02). Given that the symptoms of these diseases bear a resemblance to those of radiation side effects, elevated risks of acute RITs with extreme high OR value were obtained in our study, highlighting the need to consider them when making personalized radiotherapy plan from the perspective of radiation side effects. Meanwhile, it should be mentioned that the extremely wide confidence intervals for chickenpox and oesophagitis suggest statistical uncertainty, which requires careful consideration in practice.

It is noteworthy that mental health issues within Disease (**Figure 2B**) and Health and hematologic traits categories (**Figure 2C**) presented risks. Specifically, these mental factors included irritability (UKB data field 1940; SPA correction) (OR=1.07, 95% CI 1.01-1.13, *P*=0.02), experiencing mood swings (OR=1.14, 95% CI 1.02-1.28, *P*=0.03), and “seen doctor for nerves, anxiety, tension, or depression” (OR=1.38, 95% CI 1.06-1.81, *P*=0.02). Other risk factors such as systolic blood pressure (OR=1.08, 95% CI 1.02-1.14, *P*<0.01) and diastolic blood pressure (OR=1.01, 95% CI 1.001-1.012, *P*=0.02) were potentially linked to an increased risk of acute RITs separately in the European and Hispanic or Latin American population.

**Figure 3:**
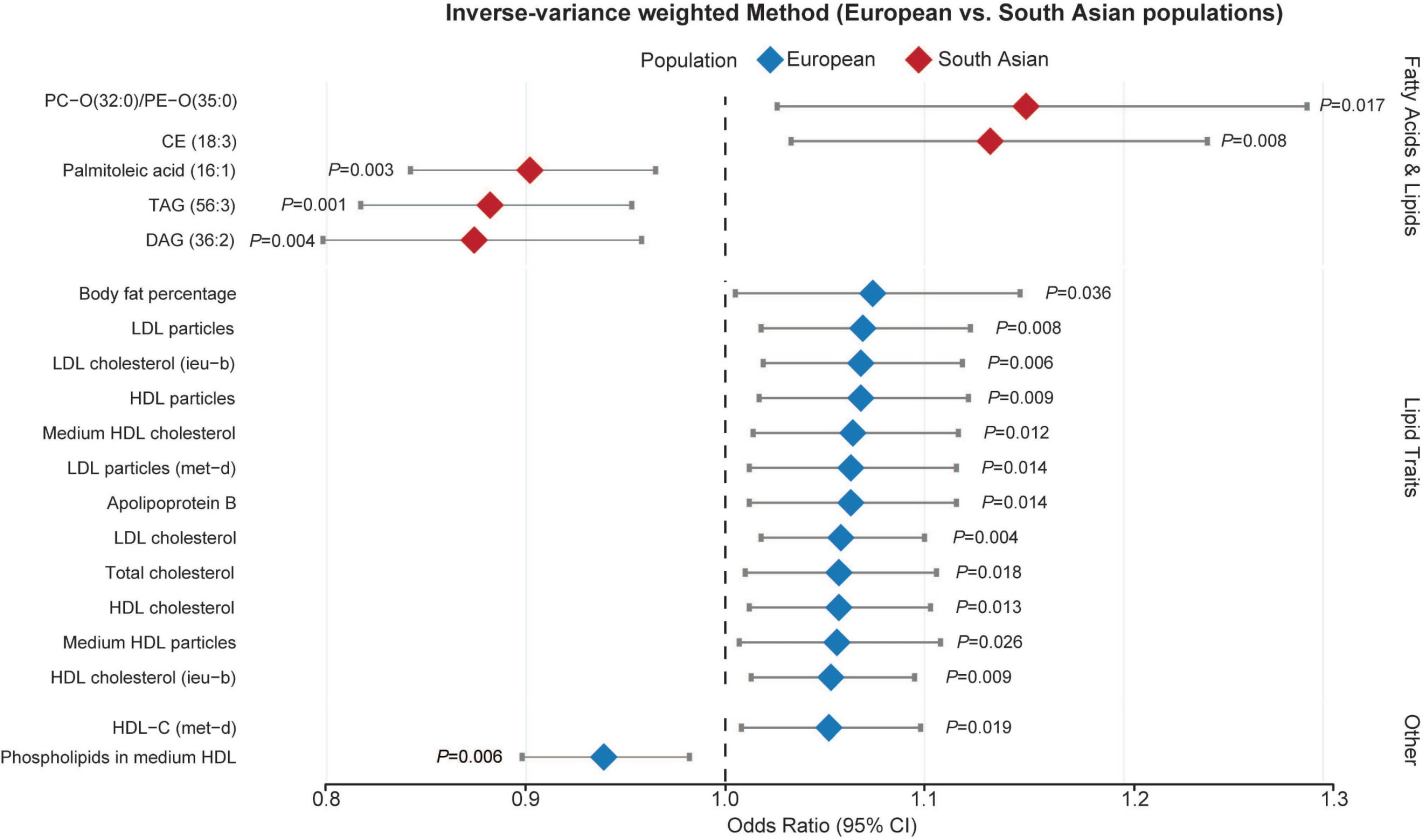
** Associations of lipoprotein or cholesterol levels with acute radiation-induced toxicities**. *Abbreviations*: OR, odds ratio; Se, standard error; lci95, 95% lower confidence interval; uci95, 95% upper confidence interval; IVW, inverse-variance weighted.

**Figure 4:**
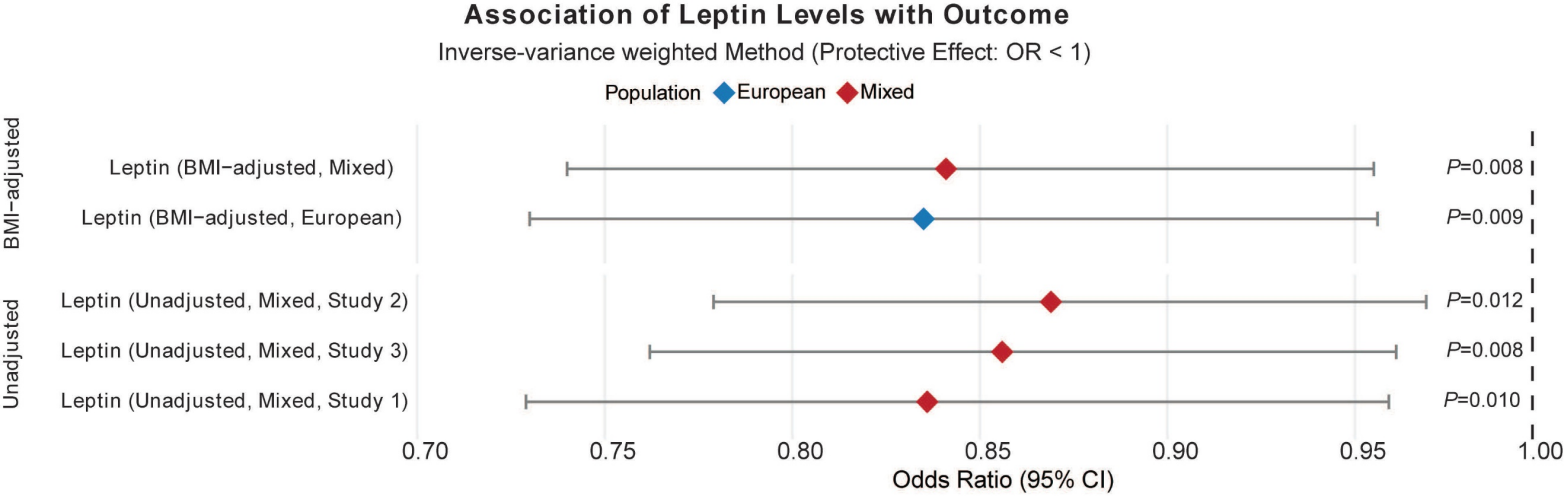
** Associations of circulating leptin levels with acute radiation-induced toxicities.** *Abbreviations*: OR, odds ratio; Se, standard error; lci95, 95% lower confidence interval; uci95, 95% upper confidence interval; IVW, inverse-variance weighted.

In the Metabolism category (**Figure 2D**), for the European population, lipoprotein or cholesterol levels was associated with an elevated risk of acute RITs while fatty acid and lipids brought benefit for South Asian people (**Figure 3 and** Additional file1-**Table S6**). Furthermore, circulating leptin levels, not or adjusted for BMI, otherwise offered protective effects against acute RITs for mixed populations (**Figure 4 and** Additional file1-**Table S7**). The detailed results of the sensitivity analysis are shown in Additional file1-**Table S3-4**.

### Real-world validation of the association between hematologic/metabolic risk factors and acute RITs in breast cancer patients

**Figure 5:**
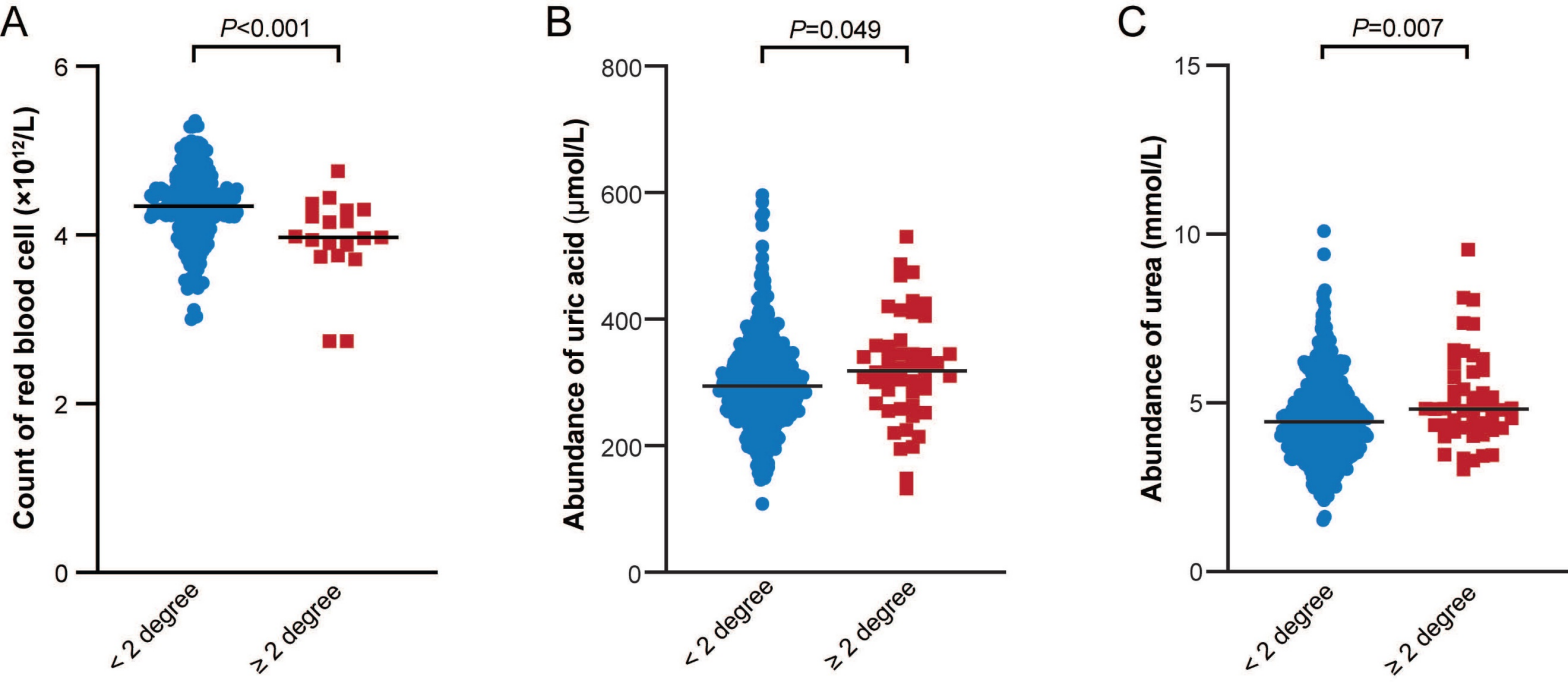
** Comparison of the three hematologic/metabolic indicators between groups with different acute radiation toxicity**.

The hematologic and metabolic risk factors supported by different levels of evidence, including red blood cell (RBC) count, mean corpuscular volume (MCV), platelet (PLT) count, mean corpuscular hemoglobin (MCH), D-dimer, serum urea levels in the *Robust* category, and glucose levels, serum alkaline phosphatase levels (APL), serum albumin (ALB) level, urate (salts of uric acid) in the *Suggestive* category, caught our attention. We collected complete blood count (CBC) and serum biochemistry profile data of 1,078 patients with breast cancer who received radiotherapy in our department, and compared the abundance of each hematologic/metabolic indicator prior to the radiotherapy between groups with different acute radiation toxicity. As shown in **Figure 5**, we observed that the RBC count was substantially higher in the < 2 degree group than in the ≥ 2 degree group. This finding was in line with the MR result, which indicated that a higher RBC count was associated with a decreased risk of acute RITs (OR=0.95, 95% CI 0.91-0.99, *P*=0.02) (Additional file1-**Table S1** and **S2**). Conversely, **Figure 5** also revealed that the serum urea and uric acid levels were significantly lower in the < 2 degree group compared to those in the ≥ 2 degree group. These results were congruent with the MR results as well. Specifically, an elevated urea level was linked to an increased risk of acute RITs (OR=1.08, 95% CI 1.01-1.15, *P*=0.02) (Additional file1-**Table S1** and** S2**). Similarly, an increased uric acid or urate level was associated with a heightened risk of acute RITs (OR=1.93, 95% CI 1.03-3.61, *P*=0.04) (Additional file1-**Table S1** and** S2**). What's more, we also identified several hematologic and metabolic risk factors that were either inconsistent with the MR results or statistically non-significant. For example, ALB, APL, CRP, D-dimer, Glu, HDL-C, MCH, MCHC, MCV, PLT, and triglyceride (TG) did not reach statistical significance (Additional file2-**Supplementary Figure 1**).

### Multivariable Mendelian randomization analysis of biological traits

**Figure 6:**
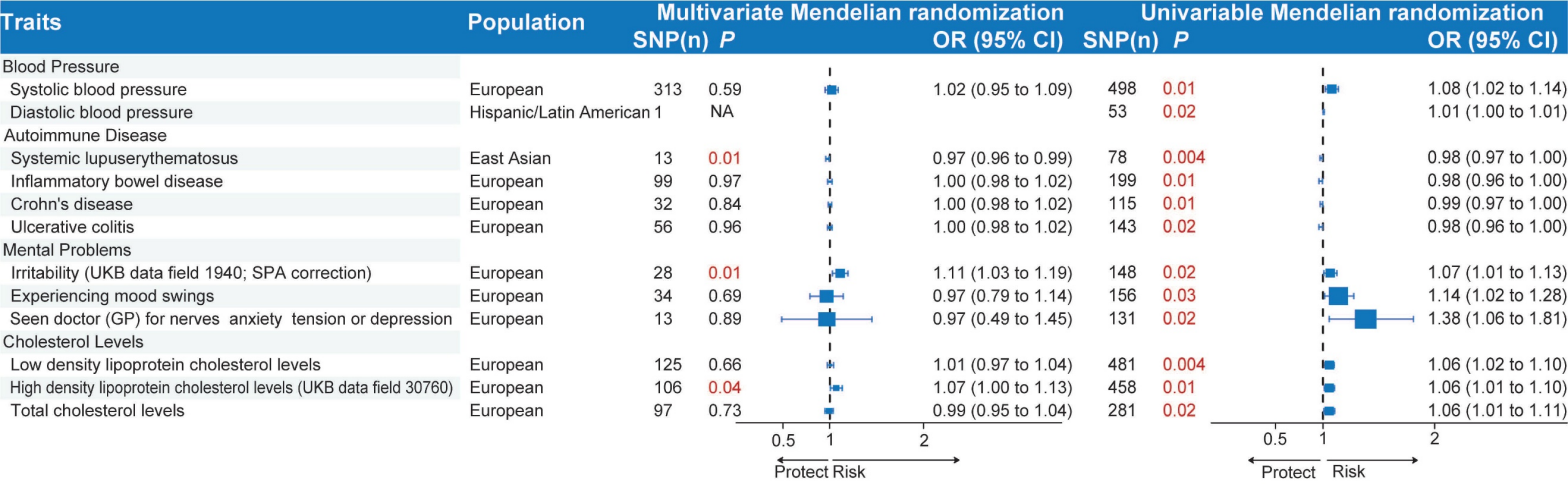
** Multivariable and univariable Mendelian randomization of biologically related traits for acute radiation-induced toxicities.** The *P* values less than 0.05 are marked in red. MVMR, multivariate Mendelian randomization; UVMR, univariable Mendelian; randomization; SNP, single-nucleotide polymorphism; OR, odds ratio; CI, confidence interval.

To deal with pleiotropy and explore potential interactions among the selected biological traits associated with acute RITs, we carried out MVMR as a type of mediation analysis, concentrating on biologically related traits (**Figure 6** and Additional file1-**Table S5**). Specifically, we made an analysis of blood pressure (diastolic blood pressure and systolic blood pressure), autoimmune diseases (inflammatory bowel disease, systemic lupus erythematosus, Crohn's disease and ulcerative colitis), mental problems (irritability (UKB data field 1940; SPA correction), experiencing mood swings, “seen doctor for nerves, anxiety, tension, or depression”), and cholesterol levels (total cholesterol levels, high density lipoprotein cholesterol levels, low density lipoprotein cholesterol levels) in their association with acute RITs. Our findings indicated that systemic lupus erythematosus (*P*_MVMR_=0.01; *P*_UVMR_<0.01), irritability (*P*_MVMR_=0.01; *P*_UVMR_=0.02) and high-density lipoprotein cholesterol levels (*P*_MVMR_=0.04; *P*_UVMR_=0.01) remained significantly associated with acute RITs in the two models. In contrast, the association of the other three of four autoimmune diseases, the other two of three mental problems and the other two of three cholesterol levels with acute RITs turned non-significant. What's more, blood pressure-related phenotypes failed to demonstrate independent impacts on the risk of acute RITs, suggesting alternative mediating pathways.

## Discussion

In this research, we utilized MR-PheWAS to decipher the intricate connections between a broad range of phenotypic traits linked to acute RITs, and detected numerous phenotypic risk factors affecting acute RITs. These factors consist of those that have been previously recorded as well as some newly uncovered ones. As a result, they offer valuable evidence that might be beneficial to the prevention and management of acute RITs.

Previous studies have indicated that the magnitude of the inflammatory response contributes to radiation toxicity, with the risk being associated with plasma transforming growth factor beta1 (TGF-β1)^39^, interleukin-6^40^, tumor necrosis factor alpha (TNF-α)^41^ and CRP^42^, ^43^. In line with the findings, this MR-PheWAS study revealed that some inflammatory factors, such as CRP and IL-4Rα were positively associated with acute RITs. We also found that certain inflammatory factors could offer protective effect, such as SCARF2, GDF15, FGF-2 and IL-16. Of them, the FGF-2^44^-^46^ and IL-16^46^ had been reported to protect against radiation toxicity. While there was no direct evidence on the associations of SCARF2 and GDF15 with radiation toxicity, some studies reported that the SCARF2 protein could reduce the risk of pulmonary fibrosis^47^ and GDF15 could ameliorate liver fibrosis by reprogramming macrophage metabolism to make them anti-inflammatory^48^, which provided the indirect evidence to support our observation.

Additionally, this research identified a number of novel biomarkers associated with acute RITs, including traits related to lipid metabolism (cholesterol and circulating leptin), blood pressure regulation, gut microbiota, and systemic autoimmune diseases (e.g., SLE). While these genetic susceptibilities highlight complex, interconnected pathways driving radioresistance or radiosensitivity, translating these broad MR-PheWAS findings into actionable clinical practice requires bridging the gap between genetic risk networks and routine physiological metrics.

To biologically validate and translate our genetic findings, we analyzed routine pre-radiotherapy hematologic and metabolic profiles in a real-world validation cohort of 1,078 breast cancer patients. Notably, serum urate (uric acid), serum urea, and red blood cell (RBC) count showed significant predictive value for acute RITs, serving as downstream clinical surrogates for the immune-inflammatory and metabolic genetic networks identified in our MR analysis. Our clinical validation revealed that elevated baseline serum urate and urea levels were associated with significantly increased risk of acute RITs. Biologically, uric acid crystals act as potent danger-associated molecular patterns (DAMPs) and strong pro-inflammatory triggers^49^-^51^. In addition, dysregulation of the urea cycle is closely linked to oxidative stress and elevated pro-inflammatory cytokines^52^, ^53^. Importantly, these clinical observations align well with our MR results, which showed that genetic predisposition to heightened inflammation (marked by CRP, IL-4Rα) and systemic autoimmune dysregulation (highlighted notably by SLE) increase acute RIT risk. Thus, elevated baseline urea and urate may serve as phenotypic "red flags" reflecting underlying enhanced immune reactivity and oxidative stress, which sensitize normal tissues to radiation-induced damage. In contrast, higher baseline RBC count was protective against acute RITs. Mechanistically, RBC membrane integrity is critical for buffering oxidative stress and dampening systemic inflammation^54^. Beyond their role in oxygen transport, RBCs possess robust antioxidant systems that scavenge radiation-induced reactive oxygen species (ROS)^55^. This clinical protective effect is consistent with our MR-PheWAS data, which linked higher genetically determined levels of tissue-repair cytokines (FGF-2, GDF15) and favorable metabolic resilience markers to reduced RIT risk. An elevated RBC count therefore reflects enhanced baseline metabolism, improved tissue oxygenation, and stronger cellular repair capacity. Collectively, our real-world clinical validation physically translates the broad genetic vulnerabilities identified by MR. As RBC count, serum urea, and uric acid are readily available in routine pre-treatment testing, they carry high translational potential. After further multi-center validation, these markers could guide pre-treatment metabolic or inflammatory modulation to reduce acute RITs and enable personalized radiotherapy.

This study's principal advantage is its hypothesis-free approach. This approach facilitates a comprehensive and unbiased exploration of the phenotypic traits related to acute RITs, and the findings are further verified by strict sensitivity analyses. Additionally, the limited overlap between outcome and exposure samples minimizes potential biases. What's more, we present the first large-scale, cross-ancestry, phenome-wide association study to elucidate prior unexplored risk factors associated with radiation-induced toxicities.

## Conclusion

In summary, this research identified phenotypic risk factors for acute RITs and provided genetic evidence consistent with several prior clinical observations. However, given the complexity of radiation-induced biological responses and potential confounding or cross-linked effects, these findings should be regarded as candidate markers rather than established causal mediators. Further large-scale prospective trials and mechanistic studies are needed to validate these associations and disentangle the interplay between host phenotypes and radiation sensitivity, thereby improving the prevention and management of acute RITs.

## Supplementary Material

Supplementary figure and tables.

## Data Availability

The exposure data was sourced from the IEU-OpenGWAS project (https://gwas.mrcieu.ac.uk/datasets/ebi-a-GCST90025994/). The summary-level data on acute RITs (GCST90435417) was downloaded from the GWAS Catalog (https://www.ebi.ac.uk/gwas/).

## Abbreviations

rSTATacute: Acute standardized total average toxicity (adjusted for covariates)
MVMR: Multivariable Mendelian randomization
UVMR: Univariable Mendelian randomization
GWAS: Genome-wide association study
MR-PheWAS: Phenome-wide association study integrated with Mendelian randomization
RITs: Acute radiation-induced toxicities
IL-4Rα: Interleukin-4 receptor alpha
GDF15: Growth differentiation factor 15
FGF-2: Fibroblast growth factor-2
IL-16: Interleukin-16
TGF-α: Transforming growth factor beta1
TNF-α: Tumor necrosis factor alpha
CRP: C-reactive protein levels
SCARF2: Scavenger receptor class F member 2
RBC: Red blood cell count
MCV: Mean corpuscular volume
PLT: Platelet count
MCH: Mean corpuscular hemoglobin
APL: Alkaline phosphatase levels
ALB: Serum albumin
CBC: Complete blood count
