# Supplementary material for: Phenotypic risk factors linked to acute radiation-induced toxicities: a phenome-wide Mendelian randomization study of 12,042 cancer patients
Source: J Cancer. 2026 Apr 23;17(5):979–89. doi: 10.7150/jca.127072 (PMC13189849; doi:10.7150/jca.127072)
Supplement: Supplementary file 1 — Supplementary figure and tables. [file jcav17p0979s1.pdf]

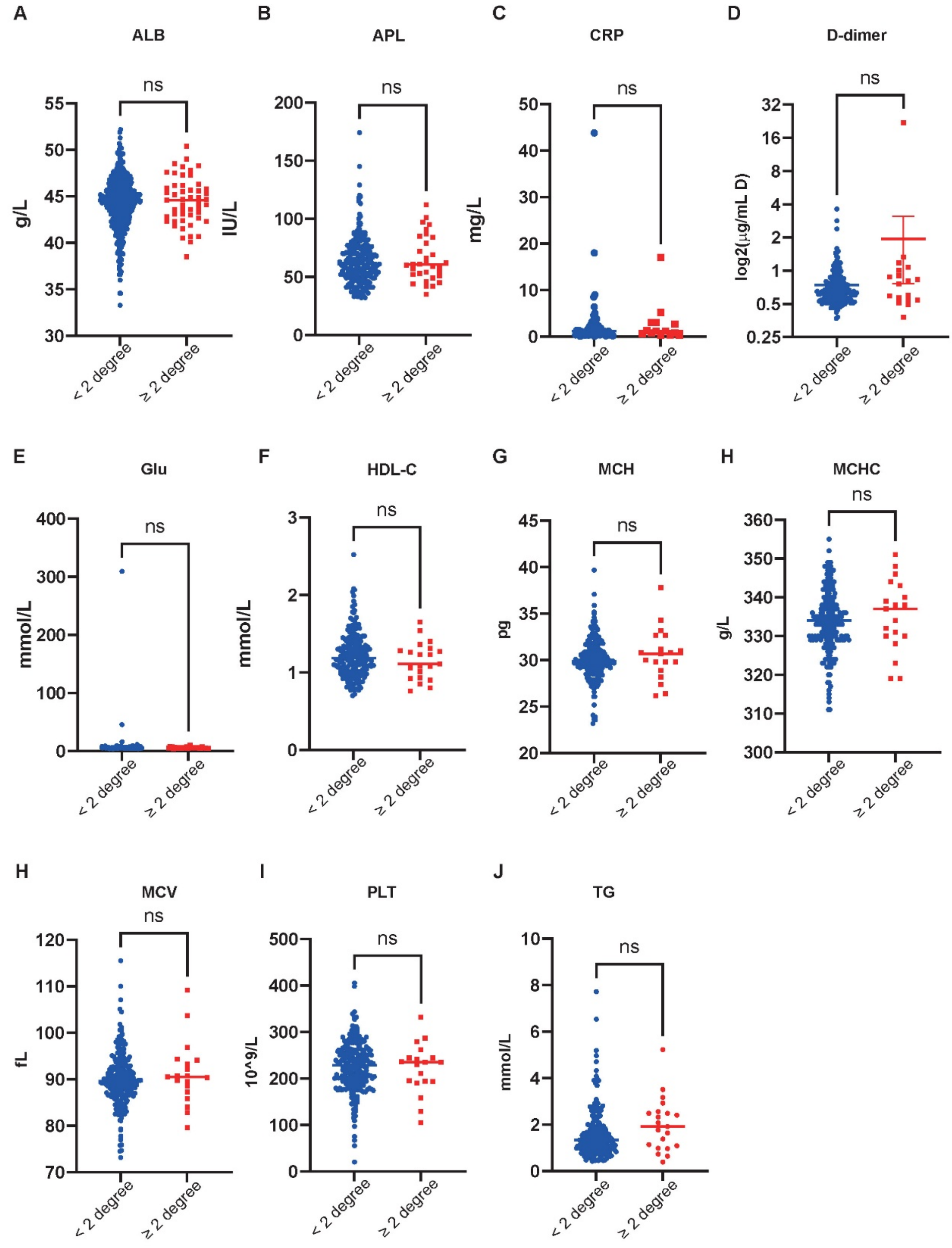

Supplementary Figure 1 ALB, APL, CRP, D-dimer, Glu, HDL-C, MCH, MCHC, MCV, PLT, and TG did not reach statistical significance. ALB, serum albumin; APL, serum alkaline phosphatase levels; CRP, C-reactive protein levels; Glu, glucose levels; HDL-C, high-density lipoprotein cholesterol; MCH, mean corpuscular hemoglobin; MCHC, Mean Corpuscular Hemoglobin Concentration; MCV, mean corpuscular volume; PLT, platelet count; TG, triglyceride.

Table S1 Results of phenome-wide Mendelian randomization analysis of 990 traits linked to acute radiation-induced toxicities (RITs)

Exposure ID	Trait name	Outcome	Trait Population	Trait Category	SNPn	Method	*β*	Se	*P* value	Lo_ci	Up_ci	OR	OR_lci95	OR_uci95
**ukb-e-23119_CSA**	Arm fat percentage (right)	acute RITs	South Asian	Anthropometrics	21	IVW	0.0844	0.0379	0.0261	0.0100	0.2864	1.0880	1.0101	1.1720
**ukb-e-23121_AFR**	Arm fat-free mass (right)	acute RITs	African American or Afro-Caribbean	Anthropometrics	32	IVW (fixed effects)	-0.0492	0.0223	0.0272	-0.0929	-0.0055	0.9520	0.9113	0.9945
**ukb-b-8909**	Body fat percentage	acute RITs	European	Anthropometrics	644	IVW	0.0711	0.0339	0.0360	0.0046	0.2587	1.0737	1.0047	1.1476
**ukb-e-23099_AFR**	Body fat percentage	acute RITs	African American or Afro-Caribbean	Anthropometrics	22	IVW (fixed effects)	-0.0455	0.0229	0.0465	-0.0903	-0.0007	0.9555	0.9137	0.9993
**ukb-e-23104_AFR**	Body mass index (BMI)	acute RITs	African American or Afro-Caribbean	Anthropometrics	25	Weighted median	-0.0477	0.0211	0.0236	-0.0891	-0.0064	0.9534	0.9148	0.9936
**ukb-e-46_AFR**	Hand grip strength (left)	acute RITs	African American or Afro-Caribbean	Anthropometrics	17	IVW (fixed effects)	-0.0571	0.0280	0.0418	-0.1121	-0.0021	0.9445	0.8940	0.9979
**ukb-b-8875**	Heel bone mineral density (BMD)	acute RITs	European	Anthropometrics	545	Weighted median	-0.0646	0.0292	0.0272	-0.1219	-0.0073	0.9375	0.8853	0.9927
**ukb-a-500**	Heel bone mineral density (BMD) T-score automated	acute RITs	European	Anthropometrics	400	Weighted median	-0.0703	0.0318	0.0273	-0.1326	-0.0079	0.9322	0.8758	0.9922
**ukb-a-500**	Heel bone mineral density (BMD) T-score automated	acute RITs	European	Anthropometrics	400	Weighted mode	-0.0835	0.0382	0.0294	-0.1583	-0.0086	0.9199	0.8536	0.9914
**ukb-a-361**	Heel bone mineral density (BMD) T-score automated (left)	acute RITs	European	Anthropometrics	257	Weighted median	-0.0699	0.0331	0.0347	-0.1347	-0.0050	0.9325	0.8739	0.9950
**ukb-a-361**	Heel bone mineral density (BMD) T-score automated (left)	acute RITs	European	Anthropometrics	257	Weighted mode	-0.0829	0.0362	0.0229	-0.1539	-0.0119	0.9204	0.8574	0.9881
**ukb-a-362**	Heel bone mineral density (BMD) T-score automated (right)	acute RITs	European	Anthropometrics	267	Weighted median	-0.0778	0.0313	0.0128	-0.1391	-0.0165	0.9251	0.8702	0.9836
**ukb-a-362**	Heel bone mineral density (BMD) T-score automated (right)	acute RITs	European	Anthropometrics	267	Weighted mode	-0.0789	0.0359	0.0290	-0.1493	-0.0085	0.9241	0.8613	0.9916
**ukb-b-20124**	Heel bone mineral density (BMD) T-score, automated	acute RITs	European	Anthropometrics	541	Weighted median	-0.0709	0.0312	0.0228	-0.1320	-0.0098	0.9315	0.8763	0.9902
**ukb-b-11364**	Heel bone mineral density (BMD), manual entry	acute RITs	European	Anthropometrics	118	IVW	-0.0547	0.0182	0.0027	-0.0904	0.0399	0.9468	0.9135	0.9812
**ukb-b-17612**	Heel bone ultrasound T-score, manual entry	acute RITs	European	Anthropometrics	83	Weighted median	-0.0720	0.0358	0.0444	-0.1422	-0.0018	0.9305	0.8674	0.9982
**ukb-b-17612**	Heel bone ultrasound T-score, manual entry	acute RITs	European	Anthropometrics	83	Weighted mode	-0.0971	0.0440	0.0300	-0.1833	-0.0109	0.9074	0.8325	0.9891
**ukb-b-17952**	Heel Broadband ultrasound attenuation (BUA), manual entry	acute RITs	European	Anthropometrics	92	Weighted median	-0.0785	0.0360	0.0291	-0.1491	-0.0080	0.9245	0.8615	0.9920
**ukb-b-17952**	Heel Broadband ultrasound attenuation (BUA), manual entry	acute RITs	European	Anthropometrics	92	Weighted mode	-0.0987	0.0471	0.0389	-0.1911	-0.0064	0.9060	0.8261	0.9936
**ukb-b-5447**	Heel broadband ultrasound attenuation (left)	acute RITs	European	Anthropometrics	304	Weighted median	-0.0900	0.0357	0.0117	-0.1599	-0.0200	0.9140	0.8522	0.9802
**ukb-b-5447**	Heel broadband ultrasound attenuation (left)	acute RITs	European	Anthropometrics	304	Weighted mode	-0.0980	0.0378	0.0099	-0.1720	-0.0239	0.9067	0.8420	0.9763
**ukb-b-6027**	Heel broadband ultrasound	acute RITs	European	Anthropometrics	311	MR	-0.0785	0.0393	0.0466	-0.1555	-0.0015	0.9245	0.8560	0.9985

	attenuation (right)					Egger								
**ukb-b-6027**	Heel broadband ultrasound attenuation (right)	acute RITs	European	Anthropometrics	311	Weighted median	-0.0788	0.0343	0.0215	-0.1459	-0.0116	0.9242	0.8642	0.9885
**ukb-b-6027**	Heel broadband ultrasound attenuation (right)	acute RITs	European	Anthropometrics	311	Weighted mode	-0.0880	0.0405	0.0307	-0.1674	-0.0085	0.9158	0.8459	0.9915
**ukb-b-15851**	Heel Broadband ultrasound attenuation, direct entry	acute RITs	European	Anthropometrics	480	Weighted median	-0.0701	0.0333	0.0356	-0.1354	-0.0047	0.9323	0.8734	0.9953
**ukb-b-19234**	Heel quantitative ultrasound index (QUI), direct entry	acute RITs	European	Anthropometrics	541	Weighted median	-0.0712	0.0308	0.0209	-0.1317	-0.0108	0.9312	0.8766	0.9893
**ukb-b-19234**	Heel quantitative ultrasound index (QUI), direct entry	acute RITs	European	Anthropometrics	541	Weighted mode	-0.0740	0.0367	0.0440	-0.1459	-0.0021	0.9287	0.8643	0.9979
**ukb-b-17848**	Heel quantitative ultrasound index (QUI), manual entry	acute RITs	European	Anthropometrics	114	IVW	-0.0583	0.0183	0.0014	-0.0941	0.0528	0.9434	0.9102	0.9777
**ukb-e-49_AFR**	Hip circumference	acute RITs	African American or Afro-Caribbean	Anthropometrics	16	IVW	-0.0558	0.0283	0.0487	-0.1113	-0.0003	0.9457	0.8947	0.9997
**ukb-e-23110_AFR**	Impedance of arm (left)	acute RITs	African American or Afro-Caribbean	Anthropometrics	22	IVW	-0.0512	0.0216	0.0178	-0.0935	0.0070	0.9501	0.9107	0.9912
**ebi-a-GCST005349**	Total body bone mineral density (age over 60)	acute RITs	Mixed	Anthropometrics	73	IVW	-0.0384	0.0173	0.0266	-0.0724	0.0328	0.9623	0.9301	0.9955
**ukb-e-23128_AFR**	Trunk fat mass	acute RITs	African American or Afro-Caribbean	Anthropometrics	23	IVW	-0.0525	0.0189	0.0056	-0.0896	0.0901	0.9489	0.9143	0.9848
**ukb-e-23127_AFR**	Trunk fat percentage	acute RITs	African American or Afro-Caribbean	Anthropometrics	33	IVW	-0.0469	0.0164	0.0043	-0.0791	0.0540	0.9542	0.9239	0.9854
**ebi-a-GCST90013422**	Ultradistal forearm bone mineral density	acute RITs	European	Anthropometrics	45	IVW	-0.0673	0.0228	0.0031	-0.1119	0.0715	0.9349	0.8941	0.9776
**ieu-a-61**	Waist circumference	acute RITs	European	Anthropometrics	105	IVW	0.0969	0.0409	0.0179	0.0167	0.3838	1.1017	1.0168	1.1937
**ieu-a-72**	Waist-to-hip ratio	acute RITs	Mixed	Anthropometrics	83	IVW (fixed effects)	0.1008	0.0497	0.0425	0.0034	0.1982	1.1061	1.0034	1.2192
**ebi-a-GCST90095035**	Waist-to-hip ratio adjusted for BMI	acute RITs	Hispanic or Latin American	Anthropometrics	31	MR Egger	0.4584	0.2095	0.0369	0.0477	0.8691	1.5815	1.0488	2.3847
**ieu-b-4810**	Breast cancer	acute RITs	European	Disease	78	IVW	0.4160	0.1980	0.0357	0.0279	1.6259	1.5159	1.0282	2.2349
**ukb-a-57**	Cancer code self-reported: prostate cancer	acute RITs	European	Disease	42	IVW (fixed effects)	-2.3201	1.1506	0.0437	-4.5752	-0.0650	0.0983	0.0103	0.9370
**ukb-b-13545**	Cancer code, self-reported: uterine/endometrial cancer	acute RITs	European	Disease	5	IVW	21.5551	7.9916	0.0070	5.8915	259.8108	2297414605.8295	361.9402	14582834247476100.0000
**ebi-a-GCST90018817**	Cervical cancer	acute RITs	European	Disease	21	IVW	-0.0227	0.0104	0.0288	-0.0431	0.1315	0.9775	0.9578	0.9977
**ebi-a-GCST012876**	Colorectal cancer	acute RITs	European	Disease	36	IVW	-0.0535	0.0188	0.0044	-0.0903	0.0635	0.9479	0.9136	0.9834
**ebi-a-GCST012877**	Colorectal cancer	acute RITs	European	Disease	38	MR Egger	-0.1147	0.0473	0.0206	-0.2075	-0.0219	0.8916	0.8126	0.9783
**ebi-a-GCST90018588**	Colorectal cancer	acute RITs	East Asian	Disease	41	IVW	0.0355	0.0142	0.0126	0.0076	0.1315	1.0361	1.0076	1.0655
**ebi-a-GCST90018838**	Endometrial cancer	acute RITs	European	Disease	15	IVW	0.0456	0.0203	0.0249	0.0058	0.1315	1.0467	1.0058	1.0893
**ieu-a-1163**	ER- Breast cancer (Survival)	acute RITs	European	Disease	22	MR Egger	0.0519	0.0234	0.0379	0.0061	0.0977	1.0533	1.0062	1.1027
**ebi-a-GCST90018875**	Lung cancer	acute RITs	European	Disease	25	IVW	0.0498	0.0217	0.0215	0.0073	0.1315	1.0511	1.0074	1.0967
**ieu-a-985**	Lung cancer	acute RITs	European	Disease	41	MR Egger	0.0857	0.0418	0.0472	0.0038	0.1676	1.0894	1.0038	1.1824
**ebi-a-GCST004747**	Lung cancer in never smokers	acute RITs	European	Disease	23	MR	0.0676	0.0292	0.0311	0.0102	0.1249	1.0699	1.0103	1.1330

						Egger								
**ukb-b-14956**	Type of cancer: ICD10: C54.1 Endometrium	acute RITs	European	Disease	4	IVW	-16.5961	7.9460	0.0367	-32.1703	190.0431	0.0000	0.0000	0.3599
**ukb-d-M13_ADHCAPSULITIS**	Adhesive capsulitis of shoulder	acute RITs	European	Disease	25	IVW (fixed effects)	5.9587	2.8699	0.0379	0.3337	11.5837	387.1109	1.3961	107338.1333
**ebi-a-GCST90086041**	Allergic rhinitis	acute RITs	European	Disease	20	IVW	0.0180	0.0073	0.0136	0.0037	0.1315	1.0181	1.0037	1.0328
**ebi-a-GCST005529**	Ankylosing spondylitis	acute RITs	European	Disease	41	IVW	-0.0962	0.0373	0.0099	-0.1694	-0.0400	0.9082	0.8442	0.9772
**ieu-a-45**	Anorexia nervosa	acute RITs	European	Disease	9	IVW	0.0093	0.0044	0.0323	0.0008	0.0151	1.0094	1.0008	1.0180
**ukb-d-KRA_PSY_ANYMENTAL**	Any mental disorder	acute RITs	European	Disease	19	IVW	5.3061	2.3638	0.0248	0.6731	20.0915	201.5536	1.9603	20723.2291
**ebi-a-GCST90000026**	Appendicular lean mass	acute RITs	European	Disease	570	IVW	-0.0422	0.0186	0.0233	-0.0786	0.0635	0.9587	0.9244	0.9943
**bbj-a-86**	Arrhythmia	acute RITs	East Asian	Disease	53	IVW	0.0263	0.0126	0.0363	0.0017	0.0840	1.0267	1.0017	1.0523
**bbj-a-71**	Atrial Fibrillation	acute RITs	East Asian	Disease	67	IVW	0.0199	0.0084	0.0184	0.0033	0.0488	1.0201	1.0034	1.0370
**ukb-b-8463**	Back pain for 3+ months	acute RITs	European	Disease	25	IVW	-0.5981	0.2715	0.0276	-1.1302	-0.0659	0.5499	0.3230	0.9362
**ukb-e-41_CSA**	Bacterial infection NOS	acute RITs	South Asian	Disease	12	IVW	0.0188	0.0082	0.0221	0.0027	0.0348	1.0189	1.0027	1.0355
**ukb-a-444**	Blood clot DVT bronchitis emphysema asthma rhinitis eczema allergy diagnosed by doctor: Emphysema/chronic bronchitis	acute RITs	European	Disease	21	IVW (fixed effects)	-2.8848	1.2936	0.0257	-5.4203	-0.3494	0.0559	0.0044	0.7051
**ukb-e-6152_p1_AFR**	Blood clot, DVT, bronchitis, emphysema, asthma, rhinitis, eczema, allergy diagnosed by doctor	acute RITs	African American or Afro-Caribbean	Disease	16	IVW	0.0176	0.0073	0.0159	0.0033	0.0341	1.0178	1.0033	1.0324
**ebi-a-GCST90038652**	Bone disorder	acute RITs	NA	Disease	65	MR Egger	6.1253	3.0435	0.0484	0.1601	12.0904	457.2597	1.1736	178156.3734
**ebi-a-GCST90018591**	Cardiac valvular disease	acute RITs	East Asian	Disease	19	IVW (fixed effects)	-0.0070	0.0032	0.0274	-0.0132	-0.0008	0.9930	0.9868	0.9992
**ebi-a-GCST90018812**	Cardiomegaly	acute RITs	European	Disease	21	IVW	-0.0462	0.0210	0.0277	-0.0873	0.1315	0.9549	0.9164	0.9950
**ukb-e-366_CSA**	Cataract	acute RITs	South Asian	Disease	13	IVW	-0.0202	0.0102	0.0471	-0.0401	0.0119	0.9800	0.9607	0.9997
**ieu-a-1058**	Celiac disease	acute RITs	European	Disease	29	MR Egger	-0.0240	0.0098	0.0217	-0.0433	-0.0047	0.9763	0.9576	0.9953
**ebi-a-GCST90018818**	Cholecystitis	acute RITs	European	Disease	48	Weighted median	-0.0460	0.0203	0.0237	-0.0859	-0.0061	0.9550	0.9177	0.9939
**ieu-b-4971**	Cholecystitits	acute RITs	European	Disease	21	Weighted mode	-0.0697	0.0319	0.0411	-0.1323	-0.0071	0.9327	0.8761	0.9929
**ebi-a-GCST90013939**	Cholelithiasis gall stones (SPA correction)	acute RITs	European	Disease	75	Weighted median	-0.0477	0.0192	0.0131	-0.0854	-0.0100	0.9534	0.9181	0.9900
**ebi-a-GCST90013939**	Cholelithiasis gall stones (SPA correction)	acute RITs	European	Disease	75	Weighted mode	-0.0414	0.0187	0.0299	-0.0781	-0.0047	0.9594	0.9249	0.9953
**ebi-a-GCST008026**	Chronic kidney disease	acute RITs	Hispanic or Latin American	Disease	31	IVW	0.0347	0.0137	0.0113	0.0079	0.0472	1.0353	1.0079	1.0636
**ebi-a-GCST90018809**	Chronic suppurative otitis media	acute RITs	European	Disease	17	IVW	-0.0371	0.0142	0.0090	-0.0649	0.1315	0.9636	0.9372	0.9908
**ukb-e-851_CSA**	Complications of transplants and reattached limbs	acute RITs	South Asian	Disease	16	MR Egger	0.0345	0.0154	0.0422	0.0042	0.0647	1.0351	1.0043	1.0668
**ukb-e-5264_AFR**	Corneal hysteresis (left)	acute RITs	African American or Afro-Caribbean	Disease	30	MR Egger	0.0745	0.0350	0.0424	0.0058	0.1432	1.0773	1.0059	1.1539
**ebi-a-GCST004132**	Crohn's disease	acute RITs	Mixed	Disease	183	IVW	-0.0122	0.0059	0.0371	-0.0237	0.0083	0.9878	0.9765	0.9993
**ieu-a-30**	Crohn's disease	acute RITs	European	Disease	115	IVW	-0.0142	0.0057	0.0136	-0.0254	0.0043	0.9859	0.9749	0.9971
**ukb-e-D64_CSA**	D64 Other anaemias	acute RITs	South Asian	Disease	12	IVW	-0.0153	0.0074	0.0379	-0.0297	-0.0027	0.9848	0.9707	0.9991
**ukb-b-3145**	Diagnoses - main ICD10:	acute RITs	European	Disease	7	IVW	-15.127	6.0226	0.0120	-26.931	-3.3228	0.0000	0.0000	0.0361

	C44.5 Skin of trunk						1			4				
**ukb-d-C67**	Diagnoses - main ICD10: C67 Malignant neoplasm of bladder	acute RITs	European	Disease	24	IVW	5.6009	2.4147	0.0204	0.8680	24.6032	270.6649	2.3821	30754.1321
**ukb-a-521**	Diagnoses - main ICD10: D12 Benign neoplasm of colon rectum anus and anal canal	acute RITs	European	Disease	24	IVW	-3.7761	1.2442	0.0024	-6.2148	-3.6729	0.0229	0.0020	0.2625
**ukb-d-D23**	Diagnoses - main ICD10: D23 Other benign neoplasms of skin	acute RITs	European	Disease	17	IVW	5.0976	2.3294	0.0286	0.5321	19.0869	163.6268	1.7024	15726.7962
**ukb-d-H33**	Diagnoses - main ICD10: H33 Retinal detachments and breaks	acute RITs	European	Disease	35	Weighted median	-4.6807	2.3671	0.0480	-9.3203	-0.0412	0.0093	0.0001	0.9597
**ukb-b-11771**	Diagnoses - main ICD10: K08.3 Retained dental root	acute RITs	European	Disease	3	IVW	-24.7170	11.2905	0.0286	-46.8465	1137.3306	0.0000	0.0000	0.0752
**ukb-b-19354**	Diagnoses - main ICD10: K20 Oesophagitis	acute RITs	European	Disease	9	IVW	10.2213	3.0951	0.0010	4.1550	16.2877	27483.6981	63.7536	11848011.6535
**ukb-b-11369**	Diagnoses - main ICD10: K40.2 Bilateral inguinal hernia, without obstruction or gangrene	acute RITs	European	Disease	7	IVW (fixed effects)	13.3763	6.5441	0.0410	0.5498	26.2027	644516.7889	1.7329	239720000000.0000
**ukb-b-19805**	Diagnoses - main ICD10: K62.1 Rectal polyp	acute RITs	European	Disease	10	MR Egger	-36.6931	15.5393	0.0459	-67.1502	-6.2360	0.0000	0.0000	0.0020
**ukb-b-8988**	Diagnoses - main ICD10: K80.1 Calculus of gallbladder with other cholecystitis	acute RITs	European	Disease	20	Weighted median	-4.8479	2.3563	0.0396	-9.4664	-0.2295	0.0078	0.0001	0.7949
**ukb-b-8988**	Diagnoses - main ICD10: K80.1 Calculus of gallbladder with other cholecystitis	acute RITs	European	Disease	20	Weighted mode	-5.0780	2.3343	0.0424	-9.6533	-0.5028	0.0062	0.0001	0.6049
**ukb-d-K81**	Diagnoses - main ICD10: K81 Cholecystitis	acute RITs	European	Disease	21	IVW	-7.3253	2.3565	0.0019	-11.9441	1.3929	0.0007	0.0000	0.0668
**ukb-d-K92**	Diagnoses - main ICD10: K92 Other diseases of digestive system	acute RITs	European	Disease	15	Weighted median	-5.2542	2.5422	0.0387	-10.2369	-0.2716	0.0052	0.0000	0.7622
**ukb-d-L57**	Diagnoses - main ICD10: L57 Skin changes due to chronic exposure to nonionising radiation	acute RITs	European	Disease	34	MR Egger	14.1263	6.4288	0.0354	1.5259	26.7267	1364441.6840	4.5991	404799000000.0000
**ukb-b-15003**	Diagnoses - main ICD10: L72.0 Epidermal cyst	acute RITs	European	Disease	8	IVW	12.5713	5.0035	0.0120	2.7645	93.4266	288168.5502	15.8713	5232143634.7479
**ukb-b-7700**	Diagnoses - main ICD10: M17.1 Other primary gonarthrosis	acute RITs	European	Disease	18	IVW	5.0592	2.4553	0.0394	0.2467	9.8717	157.4642	1.2798	19373.6011
**ukb-b-9694**	Diagnoses - main ICD10: M23.22 Derangement of meniscus due to old tear or injury (Posterior cruciate ligament or Posterior horn of medial meniscus)	acute RITs	European	Disease	6	IVW (fixed effects)	-13.8314	5.7574	0.0163	-25.1160	-2.5468	0.0000	0.0000	0.0783
**ukb-d-M70**	Diagnoses - main ICD10: M70 Soft tissue disorders related to use, overuse and pressure	acute RITs	European	Disease	15	Weighted median	-12.7987	5.9556	0.0316	-24.4717	-1.1256	0.0000	0.0000	0.3244
**ukb-b-18372**	Diagnoses - main ICD10: N20.0 Calculus of kidney	acute RITs	European	Disease	16	IVW	7.0409	2.9430	0.0167	1.2726	12.8092	1142.4020	3.5701	365562.8089
**ukb-b-18629**	Diagnoses - main ICD10: N20.1 Calculus of ureter	acute RITs	European	Disease	13	Weighted median	10.4286	5.1394	0.0424	0.3554	20.5018	33812.0328	1.4267	801310134.2000
**ukb-d-O80**	Diagnoses - main ICD10: O80 Single spontaneous delivery	acute RITs	European	Disease	17	MR Egger	20.4788	9.2934	0.0436	2.2637	38.6938	783097870.80	9.6190	637529000000

												00		00000.0000
**ukb-b-6608**	Diagnoses - main ICD10: R10.1 Pain localised to upper abdomen	acute RITs	European	Disease	9	IVW	-6.0722	2.6604	0.0225	-11.2866	3.5076	0.0023	0.0000	0.4241
**ukb-b-17456**	Diagnoses - main ICD10: R10.3 Pain localised to other parts of lower abdomen	acute RITs	European	Disease	12	IVW (fixed effects)	-5.2755	2.5970	0.0422	-10.3656	-0.1855	0.0051	0.0000	0.8307
**ukb-a-584**	Diagnoses - main ICD10: R14 Flatulence and related conditions	acute RITs	European	Disease	24	IVW (fixed effects)	15.6066	7.2203	0.0307	1.4548	29.7584	5995908.9760	4.2836	8392690000000.0000
**ukb-d-S61**	Diagnoses - main ICD10: S61 Open wound of wrist and hand	acute RITs	European	Disease	20	IVW	-5.6664	2.7878	0.0421	-11.1305	11.6002	0.0035	0.0000	0.8169
**ukb-d-Z45**	Diagnoses - main ICD10: Z45 Adjustment and management of implanted device	acute RITs	European	Disease	18	IVW	5.8853	2.6205	0.0247	0.7492	17.1844	359.7123	2.1153	61170.5388
**ukb-b-16056**	Diagnoses - secondary ICD10: F10.1 Harmful use	acute RITs	European	Disease	7	IVW	11.6276	5.5943	0.0377	0.6628	204.6921	112148.9245	1.9402	6482632595.1518
**ukb-b-19651**	Diagnoses - secondary ICD10: F10.2 Dependence syndrome	acute RITs	European	Disease	12	IVW	-11.8680	4.9205	0.0159	-21.5122	-2.2239	0.0000	0.0000	0.1082
**ukb-b-16751**	Diagnoses - secondary ICD10: J44.9 Chronic obstructive pulmonary disease, unspecified	acute RITs	European	Disease	28	IVW (fixed effects)	-3.8095	1.9161	0.0468	-7.5651	-0.0540	0.0222	0.0005	0.9475
**ukb-b-11675**	Diagnoses - secondary ICD10: K66.0 Peritoneal adhesions	acute RITs	European	Disease	5	IVW	14.9395	7.2846	0.0403	0.6617	846.5112	3076986.5844	1.9380	4885377374051.5700
**ukb-b-20233**	Diagnoses - secondary ICD10: M19.99 Arthrosis, unspecified (Site unspecified)	acute RITs	European	Disease	9	Weighted median	13.0185	5.6472	0.0211	1.9501	24.0870	450683.0305	7.0292	28895882033.0000
**ukb-b-6413**	Diagnoses - secondary ICD10: N73.6 Female pelvic peritoneal adhesions	acute RITs	European	Disease	6	Weighted median	16.5417	8.2476	0.0449	0.3764	32.7071	15275245.0200	1.4571	160139000000000.0000
**ukb-b-10490**	Diagnoses - secondary ICD10: Z03.8 Observation for other suspected diseases and conditions	acute RITs	European	Disease	11	IVW	-8.0717	4.1055	0.0493	-16.1185	237.2872	0.0003	0.0000	0.9754
**ukb-b-2895**	Diagnoses - secondary ICD10: Z60.2 Living alone	acute RITs	European	Disease	4	IVW	-10.9525	5.4614	0.0449	-21.6569	-0.2481	0.0000	0.0000	0.7803
**ukb-b-10911**	Diagnoses - secondary ICD10: Z92.2 Personal history of long-term (current) use of other medicaments	acute RITs	European	Disease	19	IVW	-4.9026	1.6296	0.0026	-8.0967	-3.0799	0.0074	0.0003	0.1811
**ukb-d-IX_CIRCULATORY**	Diseases of the circulatory system	acute RITs	European	Disease	61	IVW (fixed effects)	0.5234	0.2648	0.0481	0.0043	1.0425	1.6877	1.0043	2.8362
**ukb-a-254**	Doctor diagnosed hayfever or allergic rhinitis	acute RITs	European	Disease	67	MR Egger	-0.6609	0.3154	0.0400	-1.2791	-0.0427	0.5164	0.2783	0.9582
**ukb-d-20429**	Easily tired during worst period of anxiety	acute RITs	European	Disease	19	IVW	0.3237	0.1333	0.0152	0.0623	0.7877	1.3822	1.0643	1.7951
**ukb-e-F17_CSA**	F17 Mental and behavioral disorders due to use of tobacco	acute RITs	South Asian	Disease	17	IVW (fixed effects)	0.0094	0.0045	0.0374	0.0005	0.0182	1.0094	1.0005	1.0183
**ebi-a-GCST90018625**	Food allergy	acute RITs	East Asian	Disease	10	IVW	-0.0738	0.0289	0.0107	-0.1305	0.1315	0.9289	0.8777	0.9831
**ebi-a-GCST90000514**	Gastroesophageal reflux	acute RITs	European	Disease	226	Weighte	-0.0743	0.0325	0.0223	-0.1380	-0.0106	0.9284	0.8711	0.9895

	disease					d median								
**ebi-a-GCST90018635**	Hashimoto thyroiditis	acute RITs	East Asian	Disease	11	IVW (fixed effects)	0.0100	0.0051	0.0479	0.0001	0.0199	1.0101	1.0001	1.0201
**ukb-e-2247_p1_AFR**	Hearing difficulty/problems	acute RITs	African American or Afro-Caribbean	Disease	14	MR Egger	0.0279	0.0115	0.0317	0.0054	0.0504	1.0283	1.0054	1.0516
**ebi-a-GCST90038627**	Hepatitis	acute RITs	NA	Disease	23	IVW	6.7772	3.3551	0.0434	0.2011	0.1315	877.5658	1.2228	629821.7340
**ukb-d-K11_HERNIA**	Hernia	acute RITs	European	Disease	60	IVW	0.6948	0.3517	0.0482	0.0054	4.3434	2.0033	1.0054	3.9917
**ieu-a-1169**	Hip osteoarthritis	acute RITs	European	Disease	13	IVW	-0.0470	0.0183	0.0101	-0.0829	-0.0112	0.9540	0.9205	0.9888
**ebi-a-GCST90018860**	Hyperthyroidism	acute RITs	European	Disease	51	IVW	0.0220	0.0105	0.0359	0.0014	0.1315	1.0222	1.0014	1.0435
**ebi-a-GCST90018861**	Hypertrophic cardiomyopathy	acute RITs	European	Disease	16	IVW	-0.0176	0.0079	0.0266	-0.0331	0.1315	0.9826	0.9674	0.9980
**ukb-e-I25_AFR**	I25 Chronic ischaemic heart disease	acute RITs	African American or Afro-Caribbean	Disease	18	IVW	0.0083	0.0037	0.0225	0.0012	0.0172	1.0084	1.0012	1.0156
**ebi-a-GCST003043**	Inflammatory bowel disease	acute RITs	European	Disease	199	IVW	-0.0199	0.0080	0.0133	-0.0356	0.0097	0.9803	0.9650	0.9959
**ieu-a-292**	Inflammatory bowel disease	acute RITs	European	Disease	176	IVW	-0.0202	0.0078	0.0094	-0.0355	0.0196	0.9800	0.9651	0.9951
**ieu-a-294**	Inflammatory bowel disease	acute RITs	European	Disease	200	IVW	-0.0189	0.0080	0.0183	-0.0346	0.0091	0.9813	0.9660	0.9968
**ebi-a-GCST90038626**	Irritable bowel syndrome	acute RITs	NA	Disease	22	IVW	3.5530	1.3483	0.0084	0.9102	0.1315	34.9171	2.4849	490.6377
**bbj-a-129**	Ischemic stroke	acute RITs	East Asian	Disease	35	IVW	0.0621	0.0213	0.0035	0.0204	0.1563	1.0640	1.0206	1.1093
**ukb-e-K21_CSA**	K21 Gastro-oesophageal reflux disease	acute RITs	South Asian	Disease	10	IVW	-0.0351	0.0109	0.0013	-0.0565	0.0566	0.9656	0.9451	0.9865
**ukb-e-M17_CSA**	M17 Gonarthrosis [arthrosis of knee]	acute RITs	South Asian	Disease	15	IVW	-0.0153	0.0072	0.0331	-0.0293	0.0166	0.9848	0.9711	0.9988
**ukb-b-6458**	Mouth/teeth dental problems: Mouth ulcers	acute RITs	European	Disease	122	IVW (fixed effects)	0.3741	0.1908	0.0499	0.0002	0.7480	1.4537	1.0002	2.1128
**ukb-a-429**	Mouth/teeth dental problems: Painful gums	acute RITs	European	Disease	13	IVW	-2.9103	1.4035	0.0381	-5.6610	7.4257	0.0545	0.0035	0.8526
**ieu-b-18**	multiple sclerosis	acute RITs	European	Disease	136	IVW	-0.0140	0.0061	0.0228	-0.0260	0.0043	0.9861	0.9743	0.9981
**ukb-e-N17_CSA**	N17 Acute renal failure	acute RITs	South Asian	Disease	12	IVW	-0.0101	0.0047	0.0320	-0.0193	0.0055	0.9900	0.9809	0.9991
**ukb-e-N18_AFR**	N18 Chronic renal failure	acute RITs	African American or Afro-Caribbean	Disease	7	IVW (fixed effects)	0.0074	0.0036	0.0389	0.0004	0.0144	1.0074	1.0004	1.0145
**ebi-a-GCST006940**	Neurociticism	acute RITs	European	Disease	286	IVW	0.0929	0.0420	0.0268	0.0107	0.2398	1.0974	1.0108	1.1915
**ukb-a-230**	Neuroticism score	acute RITs	European	Disease	225	IVW	0.0254	0.0129	0.0493	0.0001	0.0506	1.0257	1.0001	1.0519
**ukb-a-81**	Non-cancer illness code self-reported: depression	acute RITs	European	Disease	37	IVW (fixed effects)	-1.1716	0.5653	0.0382	-2.2795	-0.0637	0.3099	0.1023	0.9383
**ukb-a-94**	Non-cancer illness code self-reported: hayfever/allergic rhinitis	acute RITs	European	Disease	72	IVW	-0.7422	0.3294	0.0243	-1.3878	2.4490	0.4761	0.2496	0.9080
**ukb-a-86**	Non-cancer illness code self-reported: joint disorder	acute RITs	European	Disease	25	IVW	-6.7112	2.4351	0.0059	-11.4839	3.9309	0.0012	0.0000	0.1439
**ukb-e-20002_p95_CSA**	Non-cancer illness code, self-reported	acute RITs	South Asian	Disease	15	IVW	-0.0120	0.0053	0.0240	-0.0224	0.0147	0.9881	0.9778	0.9984
**ukb-b-14027**	Non-cancer illness code, self-reported: chickenpox	acute RITs	European	Disease	6	IVW	18.4089	5.9791	0.0021	6.6898	283.0909	98831975.3985	804.1785	12146257123423.7000
**ukb-b-3044**	Non-cancer illness code, self-reported: colitis/not crohns or ulcerative colitis	acute RITs	European	Disease	6	IVW	-19.3235	7.2338	0.0076	-33.5017	104.2908	0.0000	0.0000	0.0058
**ukb-b-14452**	Non-cancer illness code,	acute RITs	European	Disease	7	IVW	12.093	5.1892	0.0198	1.9224	105.51	178660	6.8373	466843

	self-reported: inguinal hernia						2				56	.9307		7942.7830
**ukb-b-2592**	Non-cancer illness code, self-reported: irritable bowel syndrome	acute RITs	European	Disease	18	IVW	4.8102	1.6789	0.0042	1.5195	22.1987	122.7576	4.5699	3297.5570
**ukb-b-17670**	Non-cancer illness code, self-reported: multiple sclerosis	acute RITs	European	Disease	14	IVW	-5.2205	2.6174	0.0461	-10.3507	4.9046	0.0054	0.0000	0.9136
**ukb-b-6633**	Non-cancer illness code, self-reported: other renal/kidney problem	acute RITs	European	Disease	9	IVW	9.1802	4.6217	0.0470	0.1217	18.2386	9702.6444	1.1294	83357352.7826
**ukb-b-14210**	Non-cancer illness code, self-reported: rectal or colon adenoma/polyps	acute RITs	European	Disease	3	IVW	28.2307	9.9247	0.0044	8.7782	1247.8982	1821535322220.1400	6491.3272	511142150783303000000.0000
**ukb-b-15749**	Non-cancer illness code, self-reported: transient ischaemic attack (tia)	acute RITs	European	Disease	5	IVW	-17.3775	7.1686	0.0153	-31.4280	410.0071	0.0000	0.0000	0.0359
**ebi-a-GCST007091**	Osteoarthritis (hip)	acute RITs	European	Disease	71	IVW	0.0338	0.0168	0.0442	0.0009	0.1296	1.0343	1.0009	1.0689
**ukb-d-I9_ARTOTH**	Other diseases of arteries and capillaries	acute RITs	European	Disease	20	MR Egger	20.3201	9.6696	0.0499	1.3677	39.2725	668205162.4000	3.9262	114000000000000000.0000
**ukb-e-596_CSA**	Other disorders of bladder	acute RITs	South Asian	Disease	12	IVW	0.0136	0.0049	0.0060	0.0039	0.0344	1.0137	1.0039	1.0235
**ukb-e-569_AFR**	Other disorders of intestine	acute RITs	African American or Afro-Caribbean	Disease	14	IVW	0.0093	0.0035	0.0078	0.0025	0.0250	1.0094	1.0025	1.0164
**ukb-e-306_CSA**	Other mental disorder	acute RITs	South Asian	Disease	11	Weighted median	-0.0172	0.0074	0.0206	-0.0317	-0.0026	0.9830	0.9688	0.9974
**ukb-a-469**	Pain type(s) experienced in last month: Headache	acute RITs	European	Disease	111	MR Egger	1.0297	0.4963	0.0404	0.0570	2.0024	2.8001	1.0586	7.4065
**ukb-b-18596**	Pain type(s) experienced in last month: Neck or shoulder pain	acute RITs	European	Disease	90	IVW	0.5368	0.2377	0.0239	0.0709	1.0027	1.7106	1.0735	2.7257
**ukb-b-9130**	Pain type(s) experienced in last month: None of the above	acute RITs	European	Disease	177	IVW	-0.2839	0.1301	0.0291	-0.5388	0.0984	0.7528	0.5834	0.9715
**ukb-d-M13_DUPUTRYEN**	Palmar fascial fibromatosis [Dupuytren]	acute RITs	European	Disease	78	Weighted median	-1.8447	0.8925	0.0387	-3.5940	-0.0955	0.1581	0.0275	0.9089
**ebi-a-GCST90018897**	Periodontal disease	acute RITs	European	Disease	16	IVW	0.0594	0.0254	0.0192	0.0097	0.1315	1.0612	1.0097	1.1153
**ebi-a-GCST90018679**	Pleurisy	acute RITs	East Asian	Disease	11	IVW	0.0051	0.0026	0.0475	0.0001	0.1315	1.0051	1.0001	1.0103
**ebi-a-GCST90018683**	Pollinosis	acute RITs	East Asian	Disease	25	IVW	0.0907	0.0372	0.0149	0.0177	0.1315	1.0949	1.0178	1.1778
**ebi-a-GCST005581**	Primary biliary cirrhosis	acute RITs	European	Disease	43	IVW	-0.0144	0.0067	0.0322	-0.0275	-0.0120	0.9857	0.9729	0.9988
**ebi-a-GCST90013954**	Retinitis pigmentosa (SPA correction)	acute RITs	European	Disease	15	IVW (fixed effects)	-0.0396	0.0198	0.0459	-0.0784	-0.0007	0.9612	0.9246	0.9993
**ebi-a-GCST005538**	Sarcoidosis	acute RITs	European	Disease	13	IVW (fixed effects)	-0.0198	0.0100	0.0469	-0.0393	-0.0003	0.9804	0.9615	0.9997
**ukb-a-246**	Seen doctor (GP) for nerves anxiety tension or depression	acute RITs	European	Disease	131	IVW	0.3240	0.1376	0.0185	0.0544	0.5936	1.3826	1.0559	1.8105
**ebi-a-GCST90000255**	Severe COVID-19 infection with respiratory failure (analysis I)	acute RITs	European	Disease	15	IVW	-0.0207	0.0092	0.0247	-0.0388	0.0635	0.9795	0.9619	0.9974
**ebi-a-GCST90026414**	Severe insulin-resistant type 2 diabetes	acute RITs	European	Disease	12	IVW	-0.0214	0.0099	0.0305	-0.0407	0.1315	0.9789	0.9601	0.9980
**ukb-d-L12_NONION**	Skin changes due to chronic	acute RITs	European	Disease	34	MR	14.126	6.4288	0.0354	1.5259	26.726	136444	4.5991	405000

**RADISKIN**	exposure to nonionizing radiation					Egger	3				7	1.6840		000000.0000
**ebi-a-GCST003156**	Systemic lupus erythematosus	acute RITs	European	Disease	86	IVW (fixed effects)	-0.0091	0.0046	0.0485	-0.0181	-0.0001	0.9910	0.9821	0.9999
**ebi-a-GCST90011866**	Systemic lupus erythematosus	acute RITs	East Asian	Disease	78	IVW	-0.0155	0.0055	0.0044	-0.0262	-0.0093	0.9846	0.9741	0.9952
**ieu-a-815**	Systemic lupus erythematosus	acute RITs	European	Disease	10	IVW	-0.0223	0.0112	0.0471	-0.0443	0.0753	0.9779	0.9566	0.9997
**bbj-a-153**	Type 2 diabetes	acute RITs	East Asian	Disease	203	IVW	-0.0285	0.0104	0.0061	-0.0490	0.0049	0.9719	0.9522	0.9919
**ebi-a-GCST90018706**	Type 2 diabetes	acute RITs	East Asian	Disease	174	IVW	-0.0246	0.0098	0.0119	-0.0437	0.1315	0.9757	0.9572	0.9946
**ebi-a-GCST003045**	Ulcerative colitis	acute RITs	European	Disease	143	IVW	-0.0198	0.0083	0.0172	-0.0360	-0.0044	0.9804	0.9646	0.9965
**ieu-a-968**	Ulcerative colitis	acute RITs	European	Disease	118	IVW	-0.0180	0.0087	0.0399	-0.0351	0.0138	0.9822	0.9655	0.9992
**ieu-a-970**	Ulcerative colitis	acute RITs	European	Disease	145	IVW	-0.0221	0.0082	0.0067	-0.0381	-0.0045	0.9781	0.9626	0.9939
**ieu-b-5063**	URTI	acute RITs	European	Disease	15	IVW (fixed effects)	-0.0369	0.0182	0.0423	-0.0725	-0.0013	0.9638	0.9300	0.9987
**ebi-a-GCST90018718**	Uveitis	acute RITs	East Asian	Disease	5	IVW (fixed effects)	0.0021	0.0009	0.0168	0.0004	0.0039	1.0021	1.0004	1.0039
**ebi-a-GCST90011301**	C_Actinobacteria abundance	acute RITs	European	Gut microbiota	17	IVW	-0.0604	0.0266	0.0230	-0.1124	0.0657	0.9414	0.8937	0.9917
**ebi-a-GCST90011305**	C_Clostridia abundance	acute RITs	European	Gut microbiota	22	Weighted median	-0.0757	0.0367	0.0389	-0.1476	-0.0039	0.9271	0.8628	0.9962
**ebi-a-GCST90011316**	F_Rikenellaceae abundance	acute RITs	European	Gut microbiota	14	IVW	0.0840	0.0323	0.0092	0.0208	0.3167	1.0877	1.0210	1.1587
**ebi-a-GCST90011330**	G_Faecalibacterium abundance	acute RITs	European	Gut microbiota	12	IVW	0.0540	0.0260	0.0376	0.0031	0.1549	1.0555	1.0031	1.1106
**ebi-a-GCST90011333**	G_Lachnospiraceae abundance	acute RITs	European	Gut microbiota	12	IVW	0.0724	0.0341	0.0337	0.0056	0.3014	1.0751	1.0056	1.1493
**ebi-a-GCST90027467**	Gut bacterial pathway abundance (FUC.RHAMCAT.PWY..superpathway.of.fucose.and.rhamnose.degradation)	acute RITs	European	Gut microbiota	7	MR Egger	-0.3162	0.1166	0.0421	-0.5447	-0.0878	0.7289	0.5800	0.9160
**ebi-a-GCST90027487**	Gut bacterial pathway abundance (KETOGLUCONMET.PWY..ketogluconate.metabolism)	acute RITs	European	Gut microbiota	11	IVW	-0.0508	0.0205	0.0132	-0.0910	0.1315	0.9505	0.9130	0.9894
**ebi-a-GCST90027501**	Gut bacterial pathway abundance (P42.PWY..incomplete.reductive.TCA.cycle)	acute RITs	European	Gut microbiota	14	MR Egger	0.3388	0.1451	0.0377	0.0545	0.6232	1.4033	1.0560	1.8649
**ebi-a-GCST90027548**	Gut bacterial pathway abundance (PWY.5345..superpathway.of.L.methionine.biosynthesis..by.sulfhydrylation.)	acute RITs	European	Gut microbiota	10	IVW	-0.0753	0.0350	0.0316	-0.1440	0.1315	0.9274	0.8659	0.9934
**ebi-a-GCST90027577**	Gut bacterial pathway abundance (PWY.6285..superpathway.of.fatty.acids.biosynthesis..E..coli.)	acute RITs	European	Gut microbiota	12	IVW (fixed effects)	-0.0370	0.0171	0.0302	-0.0704	-0.0035	0.9637	0.9320	0.9965
**ebi-a-GCST90027623**	Gut bacterial pathway abundance (PWY.7371..1.4.dihydroxy.6.naphthoate.biosynthesis.II)	acute RITs	European	Gut microbiota	9	MR Egger	0.3686	0.1533	0.0471	0.0683	0.6690	1.4458	1.0706	1.9524
**ebi-a-GCST90027627**	Gut bacterial pathway abundance (PWY.7456..mannan.degradation)	acute RITs	European	Gut microbiota	5	IVW	-0.0999	0.0501	0.0464	-0.1981	0.1315	0.9050	0.8203	0.9984
**ebi-a-GCST90027635**	Gut bacterial pathway	acute RITs	European	Gut microbiota	11	IVW	-0.0500	0.0252	0.0476	-0.0995	-0.0005	0.9512	0.9053	0.9995

	abundance (PWY_REDCITCYC..TCA.cycle.VIII..helicobacter.)					(fixed effects)								
**ebi-a-GCST90027520**	Gut bacterial pathway abundance (PWY0.1338..polymyxin.resistance)	acute RITs	European	Gut microbiota	10	Weighted median	0.0609	0.0275	0.0265	0.0071	0.1148	1.0628	1.0071	1.1216
**ebi-a-GCST90027524**	Gut bacterial pathway abundance (PWY0.1586..peptidoglycan.maturation..meso.diaminopimelate.containing.)	acute RITs	European	Gut microbiota	9	IVW	-0.0883	0.0396	0.0257	-0.1659	0.1315	0.9155	0.8471	0.9893
**ebi-a-GCST90027640**	Gut bacterial pathway abundance (RHAMCAT.PWY..L.rhamnose.degradation.I)	acute RITs	European	Gut microbiota	12	IVW	0.0805	0.0394	0.0412	0.0032	0.1315	1.0838	1.0032	1.1708
**ebi-a-GCST90016910**	Gut microbiota abundance (class Bacilli id.1673)	acute RITs	European	Gut microbiota	18	IVW	0.0987	0.0466	0.0340	0.0075	0.1315	1.1038	1.0075	1.2093
**ebi-a-GCST90016927**	Gut microbiota abundance (family Bacteroidaceae id.917)	acute RITs	European	Gut microbiota	9	IVW	-0.1155	0.0575	0.0446	-0.2281	0.1315	0.8910	0.7960	0.9972
**ebi-a-GCST90016928**	Gut microbiota abundance (family Bacteroidales S24 7group id.11173)	acute RITs	European	Gut microbiota	9	IVW	-0.0765	0.0387	0.0481	-0.1524	0.1315	0.9263	0.8586	0.9994
**ebi-a-GCST90016952**	Gut microbiota abundance (family Streptococcaceae id.1850)	acute RITs	European	Gut microbiota	14	IVW	0.1190	0.0442	0.0071	0.0323	0.1315	1.1264	1.0329	1.2284
**ebi-a-GCST90016968**	Gut microbiota abundance (genus Bacteroides id.918)	acute RITs	European	Gut microbiota	9	IVW	-0.1155	0.0575	0.0446	-0.2281	0.1315	0.8910	0.7960	0.9972
**ebi-a-GCST90017008**	Gut microbiota abundance (genus Family XIII AD3011 group id.11293)	acute RITs	European	Gut microbiota	13	Weighted median	0.1214	0.0612	0.0472	0.0015	0.2412	1.1290	1.0015	1.2728
**ebi-a-GCST90017009**	Gut microbiota abundance (genus Family XIII UCG001 id.11294)	acute RITs	European	Gut microbiota	8	IVW	-0.1112	0.0502	0.0266	-0.2096	0.1315	0.8947	0.8109	0.9872
**ebi-a-GCST90017028**	Gut microbiota abundance (genus Lachnospiraceae UCG010 id.11330)	acute RITs	European	Gut microbiota	10	Weighted median	-0.1319	0.0591	0.0255	-0.2476	-0.0162	0.8764	0.7806	0.9840
**ebi-a-GCST90017040**	Gut microbiota abundance (genus Paraprevotella id.962)	acute RITs	European	Gut microbiota	13	IVW	-0.0802	0.0337	0.0174	-0.1463	0.1315	0.9229	0.8639	0.9860
**ebi-a-GCST90017046**	Gut microbiota abundance (genus Rikenellaceae RC9 gut group id.11191)	acute RITs	European	Gut microbiota	11	IVW	-0.0478	0.0239	0.0453	-0.0946	0.1315	0.9533	0.9098	0.9990
**ebi-a-GCST90027748**	Gut microbiota abundance (k_Bacteria.p_Actinobacteria)	acute RITs	European	Gut microbiota	10	IVW	-0.0761	0.0351	0.0301	-0.1448	0.1315	0.9267	0.8652	0.9927
**ebi-a-GCST90027651**	Gut microbiota abundance (k_Bacteria.p_Actinobacteria.c_Actinobacteria)	acute RITs	European	Gut microbiota	10	IVW	-0.0761	0.0351	0.0301	-0.1448	0.1315	0.9267	0.8652	0.9927
**ebi-a-GCST90027824**	Gut microbiota abundance (k_Bacteria.p_Bacteroidetes.c_Bacteroidia.o_Bacteroidales.f_Bacteroidaceae.g_Bacteroides.s_Bacteroides_dorei)	acute RITs	European	Gut microbiota	15	IVW	-0.1066	0.0309	0.0006	-0.1671	0.1315	0.8989	0.8461	0.9550
**ebi-a-GCST90027765**	Gut microbiota abundance (k_Bacteria.p_Bacteroidetes.c_Bacteroidia.o_Bacteroidales.f_Porphyromonadaceae.g_Odorib	acute RITs	European	Gut microbiota	14	IVW	-0.0723	0.0277	0.0090	-0.1266	0.1315	0.9302	0.8811	0.9821

	acter.s_Odoribacter_splanchnicus)													
**ebi-a-GCST90027803**	Gut microbiota abundance (k_Bacteria.p_Firmicutes.c_Erysipelotrichia.o_Erysipelotrichales.f_Erysipelotrichaceae.g_Erysipelotrichaceae_noname.s_Eubacterium_biforme)	acute RITs	European	Gut microbiota	8	IVW	-0.0547	0.0229	0.0167	-0.0996	0.1315	0.9467	0.9052	0.9901
**ebi-a-GCST90027805**	Gut microbiota abundance (k_Bacteria.p_Firmicutes.c_Erysipelotrichia.o_Erysipelotrichales.f_Erysipelotrichaceae.g_Holdemania.s_Holdemania_unclassified)	acute RITs	European	Gut microbiota	9	IVW (fixed effects)	0.0484	0.0237	0.0412	0.0019	0.0949	1.0496	1.0019	1.0995
**ebi-a-GCST90017101**	Gut microbiota abundance (order Lactobacillales id.1800)	acute RITs	European	Gut microbiota	15	IVW	0.1347	0.0439	0.0022	0.0486	0.1315	1.1442	1.0498	1.2470
**ebi-a-GCST90017112**	Gut microbiota abundance (phylum Cyanobacteria id.1500)	acute RITs	European	Gut microbiota	8	IVW	0.1106	0.0455	0.0149	0.0215	0.1315	1.1170	1.0218	1.2211
**ebi-a-GCST90017079**	Gut microbiota abundance (unknown genus id.1000005479)	acute RITs	European	Gut microbiota	9	IVW	-0.0765	0.0387	0.0481	-0.1524	0.1315	0.9263	0.8586	0.9994
**ebi-a-GCST90017082**	Gut microbiota abundance (unknown genus id.2001)	acute RITs	European	Gut microbiota	10	IVW	0.1053	0.0418	0.0117	0.0234	0.1315	1.1111	1.0237	1.2059
**ebi-a-GCST90032456**	Lactobacillus B abundance in stool	acute RITs	European	Gut microbiota	16	MR Egger	0.1822	0.0814	0.0419	0.0227	0.3416	1.1998	1.0230	1.4073
**ebi-a-GCST90032488**	Negativibacillus massiliensis abundance in stool	acute RITs	European	Gut microbiota	15	IVW	-0.1690	0.0638	0.0081	-0.2941	0.1315	0.8445	0.7452	0.9571
**ebi-a-GCST90011556**	OTU97_100 (Ruminococcaceae) prevalence	acute RITs	European	Gut microbiota	8	IVW	-0.0417	0.0166	0.0120	-0.0743	0.2470	0.9591	0.9284	0.9909
**ebi-a-GCST90011359**	OTU97_106 (Ruminococcaceae) abundance	acute RITs	European	Gut microbiota	8	IVW	-0.0471	0.0237	0.0467	-0.0936	0.3014	0.9540	0.9107	0.9993
**ebi-a-GCST90011570**	OTU97_130 (Butyrivibrio) prevalence	acute RITs	European	Gut microbiota	7	IVW	0.0315	0.0124	0.0110	0.0072	0.2470	1.0320	1.0073	1.0574
**ebi-a-GCST90011572**	OTU97_138 (Oscillibacter) prevalence	acute RITs	European	Gut microbiota	12	IVW	-0.0364	0.0147	0.0133	-0.0652	0.2470	0.9643	0.9369	0.9925
**ebi-a-GCST90011372**	OTU97_140 (Bacteroides) abundance	acute RITs	European	Gut microbiota	15	IVW	-0.0216	0.0108	0.0461	-0.0427	-0.0048	0.9787	0.9582	0.9996
**ebi-a-GCST90011385**	OTU97_24 (Prevotella) abundance	acute RITs	European	Gut microbiota	10	IVW	0.0299	0.0133	0.0251	0.0037	0.2577	1.0303	1.0037	1.0576
**ebi-a-GCST90011604**	OTU97_53 (Bacteroides) prevalence	acute RITs	European	Gut microbiota	15	IVW	0.0307	0.0140	0.0287	0.0032	0.2470	1.0312	1.0032	1.0599
**ebi-a-GCST90011605**	OTU97_56 (Ruminococcaceae) prevalence	acute RITs	European	Gut microbiota	10	IVW	-0.0371	0.0163	0.0232	-0.0691	0.2470	0.9636	0.9332	0.9949
**ebi-a-GCST90011636**	OTU99_155 (Butyrivibrio) prevalence	acute RITs	European	Gut microbiota	8	IVW	0.0307	0.0117	0.0088	0.0077	0.2470	1.0312	1.0078	1.0551
**ebi-a-GCST90011440**	OTU99_171 (Bacteroides) abundance	acute RITs	European	Gut microbiota	15	MR Egger	-0.0627	0.0257	0.0299	-0.1132	-0.0123	0.9392	0.8930	0.9878
**ebi-a-GCST90011655**	OTU99_34 (Holdemanella) prevalence	acute RITs	European	Gut microbiota	14	IVW	0.0282	0.0129	0.0290	0.0029	0.2470	1.0286	1.0029	1.0549
**ebi-a-GCST90011465**	OTU99_558 (Bacteroidales) abundance	acute RITs	European	Gut microbiota	19	IVW	-0.0104	0.0052	0.0451	-0.0205	0.0325	0.9897	0.9797	0.9998
**ebi-a-GCST90011675**	OTU99_8 (Ruminococcaceae) prevalence	acute RITs	European	Gut microbiota	9	IVW	-0.0425	0.0180	0.0181	-0.0778	0.2470	0.9584	0.9251	0.9928
**ebi-a-GCST90011481**	P_Actinobacteria abundance	acute RITs	European	Gut microbiota	17	IVW	-0.0604	0.0266	0.0230	-0.1124	0.0524	0.9414	0.8937	0.9917

**ebi-a-GCST90011482**	P_Bacteroidetes abundance	acute RITs	European	Gut microbiota	10	IVW	0.0869	0.0369	0.0186	0.0145	0.2470	1.0908	1.0146	1.1727
**ebi-a-GCST90032500**	Parabacteroides sp000436495 abundance in stool	acute RITs	European	Gut microbiota	12	IVW (fixed effects)	0.0586	0.0268	0.0288	0.0061	0.1110	1.0603	1.0061	1.1174
**ebi-a-GCST90032502**	Parachlamydiales abundance in stool	acute RITs	European	Gut microbiota	10	IVW	0.3480	0.1506	0.0209	0.0527	0.1315	1.4162	1.0541	1.9026
**ebi-a-GCST90032540**	Roseibacillus abundance in stool	acute RITs	European	Gut microbiota	5	IVW (fixed effects)	0.2951	0.1458	0.0429	0.0094	0.5808	1.3433	1.0095	1.7875
**ebi-a-GCST90032569**	Succiniclasticum abundance in stool	acute RITs	European	Gut microbiota	30	IVW	-0.1233	0.0504	0.0145	-0.2221	0.1315	0.8840	0.8008	0.9759
**ebi-a-GCST90032578**	Terrisporobacter abundance in stool	acute RITs	European	Gut microbiota	15	IVW (fixed effects)	0.0769	0.0475	0.1054	-0.0162	0.1699	1.0799	0.9840	1.1852
**ebi-a-GCST90011687**	TestASV_14 (Lachnospiraceae) prevalence	acute RITs	European	Gut microbiota	7	IVW (fixed effects)	-0.0405	0.0202	0.0456	-0.0802	-0.0008	0.9603	0.9230	0.9992
**ebi-a-GCST90032590**	UBA1033 sp001695555 abundance in stool	acute RITs	European	Gut microbiota	12	IVW (fixed effects)	0.2164	0.0980	0.0272	0.0244	0.4084	1.2416	1.0247	1.5045
**ebi-a-GCST90032601**	UBA1409 abundance in stool	acute RITs	European	Gut microbiota	12	IVW	0.1042	0.0517	0.0440	0.0028	0.1315	1.1098	1.0028	1.2282
**ebi-a-GCST90032608**	UBA1777 sp900316255 abundance in stool	acute RITs	European	Gut microbiota	14	IVW (fixed effects)	0.1844	0.0915	0.0438	0.0051	0.3637	1.2025	1.0051	1.4386
**ebi-a-GCST90032616**	UBA3855 sp900316885 abundance in stool	acute RITs	European	Gut microbiota	15	IVW	-0.2145	0.0839	0.0106	-0.3789	0.1315	0.8069	0.6846	0.9511
**ebi-a-GCST90032618**	UBA6382 abundance in stool	acute RITs	European	Gut microbiota	13	IVW	0.1427	0.0648	0.0275	0.0158	0.1315	1.1534	1.0160	1.3095
**ebi-a-GCST90032628**	UBA737 sp002451855 abundance in stool	acute RITs	European	Gut microbiota	17	IVW	0.1357	0.0671	0.0431	0.0042	0.1315	1.1454	1.0042	1.3064
**ebi-a-GCST90092504**	Carotid Intima-media thickness (mean of the maximum cIMT)	acute RITs	Sub-Saharan African	Health and hematologic traits	22	MR Egger	1.1514	0.5340	0.0434	0.1048	2.1980	3.1626	1.1105	9.0067
**ebi-a-GCST008029**	Diastolic blood pressure	acute RITs	Hispanic or Latin American	Health and hematologic traits	53	IVW	0.0064	0.0028	0.0198	0.0010	0.0301	1.0064	1.0010	1.0119
**ieu-b-106**	FEV1/FVC < 0.7	acute RITs	European	Health and hematologic traits	245	IVW	0.2098	0.1028	0.0413	0.0083	0.7268	1.2335	1.0084	1.5088
**ieu-b-4853**	Forced expiratory volume in 1-second	acute RITs	European	Health and hematologic traits	8	MR Egger	-0.6589	0.2684	0.0495	-1.1849	-0.1328	0.5174	0.3058	0.8756
**ebi-a-GCST90020194**	Inferior Posterior lobe of cerebellar volume (including Crus II to IX hemispheric lobules)	acute RITs	European	Health and hematologic traits	73	IVW	-0.1103	0.0435	0.0112	-0.1955	0.1315	0.8955	0.8224	0.9752
**ukb-b-19277**	Intra-ocular pressure, corneal-compensated (right)	acute RITs	European	Health and hematologic traits	143	IVW	-0.0602	0.0301	0.0454	-0.1192	-0.0012	0.9416	0.8876	0.9988
**bbj-a-37**	Mean arterial pressure	acute RITs	East Asian	Health and hematologic traits	83	IVW	0.0915	0.0454	0.0438	0.0025	0.3606	1.0959	1.0025	1.1978
**ebi-a-GCST90018743**	Mean arterial pressure	acute RITs	East Asian	Health and hematologic traits	99	IVW	0.1049	0.0481	0.0293	0.0106	0.1315	1.1106	1.0106	1.2204
**ukb-e-MAP_p2_AFR**	Mean arterial pressure,	acute RITs	African	Health and	24	Weighte	-0.0531	0.0261	0.0419	-0.1043	-0.0019	0.9483	0.9009	0.9981

	automated reading, adjusted by medication		American or Afro-Caribbean	hematologic traits		d median								
**ukb-e-MAP_p4_AFR**	Mean arterial pressure, combined automated + manual reading, adjusted by medication	acute RITs	African American or Afro-Caribbean	Health and hematologic traits	25	Weighted median	-0.0546	0.0243	0.0248	-0.1022	-0.0069	0.9469	0.9028	0.9931
**ukb-d-30050_irnt**	Mean corpuscular haemoglobin	acute RITs	European	Health and hematologic traits	457	MR Egger	0.0722	0.0288	0.0126	0.0157	0.1288	1.0749	1.0158	1.1374
**ebi-a-GCST004630**	Mean corpuscular hemoglobin	acute RITs	European	Health and hematologic traits	296	IVW	0.0441	0.0149	0.0030	0.0150	0.0995	1.0451	1.0151	1.0759
**ebi-a-GCST90002390**	Mean corpuscular hemoglobin	acute RITs	European	Health and hematologic traits	547	IVW	0.0326	0.0154	0.0341	0.0024	0.1247	1.0332	1.0025	1.0648
**ebi-a-GCST90002391**	Mean corpuscular hemoglobin concentration	acute RITs	European	Health and hematologic traits	326	Weighted median	0.1090	0.0434	0.0121	0.0239	0.1940	1.1151	1.0242	1.2142
**ebi-a-GCST90002391**	Mean corpuscular hemoglobin concentration	acute RITs	European	Health and hematologic traits	326	Weighted mode	0.0859	0.0418	0.0407	0.0040	0.1678	1.0897	1.0040	1.1827
**ebi-a-GCST90025962**	Mean corpuscular hemoglobin concentration	acute RITs	European	Health and hematologic traits	498	IVW	0.0429	0.0165	0.0092	0.0106	0.1315	1.0439	1.0107	1.0781
**ebi-a-GCST004602**	Mean corpuscular volume	acute RITs	European	Health and hematologic traits	314	IVW	0.0366	0.0145	0.0117	0.0081	0.0873	1.0373	1.0082	1.0673
**ebi-a-GCST90002392**	Mean corpuscular volume	acute RITs	European	Health and hematologic traits	612	IVW	0.0318	0.0150	0.0336	0.0025	0.0808	1.0323	1.0025	1.0630
**ebi-a-GCST90025963**	Mean corpuscular volume	acute RITs	European	Health and hematologic traits	530	Weighted median	0.0656	0.0296	0.0267	0.0076	0.1236	1.0678	1.0076	1.1315
**ukb-d-30040_irnt**	Mean corpuscular volume	acute RITs	European	Health and hematologic traits	477	IVW	0.0445	0.0157	0.0047	0.0137	0.1025	1.0455	1.0138	1.0782
**ebi-a-GCST90013979**	Mean corpuscular volume (UKB data field 30040)	acute RITs	European	Health and hematologic traits	491	MR Egger	0.0662	0.0299	0.0274	0.0076	0.1248	1.0684	1.0076	1.1329
**ukb-a-251**	Overall health rating	acute RITs	European	Health and hematologic traits	262	IVW	0.1497	0.0621	0.0159	0.0280	0.2715	1.1615	1.0284	1.3119
**ieu-a-274**	Packed cell volume	acute RITs	Mixed	Health and hematologic traits	46	IVW	-0.0265	0.0126	0.0358	-0.0513	0.0482	0.9738	0.9500	0.9982
**ebi-a-GCST90000065**	Pulse pressure	acute RITs	European	Health and hematologic traits	286	IVW	0.0887	0.0450	0.0486	0.0006	0.0635	1.0928	1.0006	1.1935
**ukb-e-PP_p1_CSA**	Pulse pressure, automated reading	acute RITs	South Asian	Health and hematologic traits	17	IVW	-0.0769	0.0343	0.0247	-0.1441	0.1331	0.9260	0.8658	0.9903
**ukb-e-R19_CSA**	R19 Other symptoms and signs involving the digestive system and abdomen	acute RITs	South Asian	Health and hematologic traits	12	Weighted median	0.0249	0.0105	0.0171	0.0044	0.0454	1.0252	1.0044	1.0465
**ebi-a-GCST90020192**	Superior Posterior lobe of cerebellar volume (including VI to Crus I hemispheric	acute RITs	European	Health and hematologic traits	85	MR Egger	0.4087	0.1960	0.0401	0.0245	0.7929	1.5049	1.0248	2.2098

	lobules)													
**ebi-a-GCST90020190**	Total cerebellar volume (excluding Crus I vermis)	acute RITs	European	Health and hematologic traits	60	IVW	-0.1038	0.0521	0.0462	-0.2058	0.1315	0.9014	0.8140	0.9982
**ebi-a-GCST90002228**	Two-hour glucose	acute RITs	Hispanic or Latin American	Health and hematologic traits	16	IVW	0.0568	0.0232	0.0143	0.0114	0.2007	1.0585	1.0114	1.1077
**ukb-d-5610_3**	Which eye(s) affected by presbyopia: Both eyes	acute RITs	European	Health and hematologic traits	17	IVW	0.3845	0.1961	0.0499	0.0002	1.3641	1.4689	1.0002	2.1572
**ukb-e-Z09_CSA**	Z09 Follow-up examination after treatment for conditions other than malignant neoplasms	acute RITs	South Asian	Health and hematologic traits	10	IVW	0.0156	0.0046	0.0007	0.0066	0.0346	1.0158	1.0066	1.0250
**ukb-e-Z12_CSA**	Z12 Special screening examination for neoplasms	acute RITs	South Asian	Health and hematologic traits	12	IVW (fixed effects)	-0.0090	0.0044	0.0423	-0.0176	-0.0003	0.9911	0.9825	0.9997
**ieu-b-117**	HOMA-B	acute RITs	European	Health and hematologic traits	15	IVW (fixed effects)	-0.2387	0.1138	0.0360	-0.4618	-0.0155	0.7877	0.6302	0.9846
**ieu-a-1046**	Pallidum volume	acute RITs	European	Health and hematologic traits	12	MR Egger	0.0023	0.0010	0.0473	0.0003	0.0044	1.0023	1.0003	1.0044
**ukb-e-2010_AFR**	Suffer from 'nerves'	acute RITs	African American or Afro-Caribbean	Health and hematologic traits	16	IVW	0.0222	0.0094	0.0183	0.0037	0.0940	1.0224	1.0038	1.0414
**ebi-a-GCST90025968**	Systolic blood pressure	acute RITs	European	Health and hematologic traits	498	IVW	0.0763	0.0273	0.0052	0.0228	0.1315	1.0793	1.0230	1.1387
**ieu-b-38**	systolic blood pressure	acute RITs	European	Health and hematologic traits	642	Weighted median	0.0051	0.0024	0.0379	0.0003	0.0098	1.0051	1.0003	1.0099
**ebi-a-GCST90014018**	Systolic blood pressure automated reading (UKB data field 4080)	acute RITs	European	Health and hematologic traits	400	IVW	0.0679	0.0342	0.0473	0.0008	0.1315	1.0702	1.0008	1.1445
**ukb-e-SBP_p1_AFR**	Systolic blood pressure, automated reading, adjusted by medication	acute RITs	African American or Afro-Caribbean	Health and hematologic traits	29	IVW	-0.0373	0.0174	0.0315	-0.0714	0.0126	0.9634	0.9311	0.9967
**ukb-e-SBP_p2_AFR**	Systolic blood pressure, combined automated + manual reading	acute RITs	African American or Afro-Caribbean	Health and hematologic traits	19	IVW	-0.0450	0.0217	0.0383	-0.0877	0.0754	0.9560	0.9161	0.9976
**ukb-e-SBP_p3_AFR**	Systolic blood pressure, combined automated + manual reading, adjusted by medication	acute RITs	African American or Afro-Caribbean	Health and hematologic traits	21	IVW (fixed effects)	-0.0384	0.0196	0.0497	-0.0768	0.0000	0.9623	0.9261	1.0000
**ukb-d-20541**	Difficulty stopping worrying during worst period of anxiety	acute RITs	European	Health and hematologic traits	17	MR Egger	-1.7954	0.7582	0.0317	-3.2814	-0.3094	0.1661	0.0376	0.7339
**ukb-d-20425**	Ever worried more than most people would in similar situation	acute RITs	European	Health and hematologic traits	35	IVW	0.4002	0.1866	0.0320	0.0344	1.4147	1.4921	1.0350	2.1510
**ebi-a-GCST006944**	Experiencing mood swings	acute RITs	European	Health and hematologic traits	156	IVW	0.1296	0.0585	0.0267	0.0150	0.2398	1.1384	1.0151	1.2767
**ukb-a-47**	Irritability	acute RITs	European	Health and hematologic traits	132	IVW	0.3030	0.1434	0.0346	0.0219	1.9255	1.3540	1.0222	1.7934
**ebi-a-GCST90013875**	Irritability (UKB data field	acute RITs	European	Health and	148	IVW	0.0634	0.0280	0.0237	0.0085	0.2958	1.0654	1.0085	1.1255

	1940) (Firth correction)			hematologic traits										
**ebi-a-GCST90013925**	Irritability (UKB data field 1940) (SPA correction)	acute RITs	European	Health and hematologic traits	148	IVW	0.0634	0.0280	0.0235	0.0085	0.2973	1.0655	1.0086	1.1256
**ebi-a-GCST90013873**	Mood swings (UKB data field 1920) (Firth correction)	acute RITs	European	Health and hematologic traits	162	IVW	0.0587	0.0297	0.0484	0.0004	0.3815	1.0604	1.0004	1.1240
**ebi-a-GCST90013923**	Mood swings (UKB data field 1920) (SPA correction)	acute RITs	European	Health and hematologic traits	162	IVW	0.0587	0.0297	0.0484	0.0004	0.3863	1.0604	1.0004	1.1240
**ebi-a-GCST90001501**	Activated & secreting CD4 regulatory T cell Absolute Count	acute RITs	European	Health and hematologic traits	15	IVW (fixed effects)	0.0255	0.0091	0.0049	0.0077	0.0433	1.0258	1.0078	1.0442
**ebi-a-GCST90001694**	CD28- CD4-CD8- T cell %T cell	acute RITs	European	Health and hematologic traits	20	IVW	-0.0290	0.0129	0.0243	-0.0543	0.0635	0.9714	0.9472	0.9962
**ebi-a-GCST90001654**	CD28- CD4-CD8- T cell Absolute Count	acute RITs	European	Health and hematologic traits	21	IVW	-0.0308	0.0130	0.0180	-0.0564	0.0635	0.9696	0.9452	0.9947
**ebi-a-GCST90001641**	CD3- lymphocyte %leukocyte	acute RITs	European	Health and hematologic traits	16	IVW	-0.0437	0.0170	0.0102	-0.0771	0.0635	0.9572	0.9258	0.9897
**ebi-a-GCST90001491**	CD39+ activated CD4 regulatory T cell %CD4 regulatory T cell	acute RITs	European	Health and hematologic traits	20	IVW	-0.0234	0.0071	0.0010	-0.0374	0.0635	0.9769	0.9633	0.9906
**ebi-a-GCST90001489**	CD39+ activated CD4 regulatory T cell Absolute Count	acute RITs	European	Health and hematologic traits	25	IVW	-0.0191	0.0075	0.0113	-0.0339	0.0635	0.9811	0.9667	0.9957
**ebi-a-GCST90001495**	CD39+ secreting CD4 regulatory T cell Absolute Count	acute RITs	European	Health and hematologic traits	21	IVW	-0.0179	0.0069	0.0098	-0.0314	0.0635	0.9823	0.9690	0.9957
**ebi-a-GCST90001548**	Central Memory CD8+ T cell Absolute Count	acute RITs	European	Health and hematologic traits	21	IVW	-0.0351	0.0176	0.0460	-0.0695	0.0635	0.9655	0.9328	0.9994
**ebi-a-GCST90001556**	Effector Memory CD8+ T cell %T cell	acute RITs	European	Health and hematologic traits	12	IVW (fixed effects)	0.0224	0.0108	0.0376	0.0013	0.0435	1.0227	1.0013	1.0445
**ebi-a-GCST90001554**	Effector Memory CD8+ T cell Absolute Count	acute RITs	European	Health and hematologic traits	22	IVW	0.0287	0.0139	0.0391	0.0014	0.0635	1.0291	1.0014	1.0576
**ukb-e-30210_CSA**	Eosinophill percentage	acute RITs	South Asian	Health and hematologic traits	26	IVW	0.0403	0.0203	0.0476	0.0004	0.0659	1.0411	1.0004	1.0835
**ukb-e-30300_AFR**	High light scatter reticulocyte count	acute RITs	African American or Afro-Caribbean	Health and hematologic traits	28	MR Egger	0.1193	0.0536	0.0348	0.0143	0.2242	1.1267	1.0144	1.2513
**ebi-a-GCST90001441**	IgD+ CD24- B cell %lymphocyte	acute RITs	European	Health and hematologic traits	17	IVW	0.0398	0.0188	0.0339	0.0030	0.0635	1.0406	1.0030	1.0796
**ebi-a-GCST90001431**	IgD+ CD38- B cell %lymphocyte	acute RITs	European	Health and hematologic traits	11	IVW	-0.0607	0.0228	0.0076	-0.1053	0.0635	0.9411	0.9000	0.9840
**ebi-a-GCST90001396**	IgD+ CD38- B cell Absolute Count	acute RITs	European	Health and hematologic traits	12	IVW	-0.0493	0.0195	0.0114	-0.0874	0.0635	0.9519	0.9163	0.9890

**ebi-a-GCST90018962**	Lymphocyte count	acute RITs	European	Health and hematologic traits	499	Weighted mode	0.1222	0.0609	0.0452	0.0029	0.2415	1.1300	1.0029	1.2731
**ukb-d-30120_irnt**	Lymphocyte count	acute RITs	European	Health and hematologic traits	484	Weighted mode	0.1198	0.0546	0.0288	0.0127	0.2269	1.1273	1.0128	1.2547
**ebi-a-GCST004627**	Lymphocyte counts	acute RITs	European	Health and hematologic traits	285	IVW	0.0452	0.0207	0.0293	0.0046	0.1525	1.0463	1.0046	1.0897
**bbj-a-44**	Neutrophil count	acute RITs	East Asian	Health and hematologic traits	72	Weighted median	0.0916	0.0450	0.0420	0.0033	0.1798	1.0959	1.0033	1.1970
**ebi-a-GCST90002356**	Platelet count	acute RITs	Mixed	Health and hematologic traits	48	IVW	0.0534	0.0172	0.0019	0.0197	0.1247	1.0549	1.0199	1.0911
**ieu-a-1008**	Platelet count	acute RITs	European	Health and hematologic traits	72	MR Egger	0.0018	0.0009	0.0492	0.0000	0.0036	1.0018	1.0000	1.0036
**ebi-a-GCST004601**	Red blood cell count	acute RITs	European	Health and hematologic traits	313	IVW	-0.0497	0.0205	0.0153	-0.0898	-0.0166	0.9516	0.9141	0.9905
**ebi-a-GCST90025964**	Red blood cell count	acute RITs	European	Health and hematologic traits	568	IVW	-0.0474	0.0203	0.0198	-0.0872	0.1315	0.9537	0.9165	0.9925
**ebi-a-GCST90001578**	Transitional B cell %lymphocyte	acute RITs	European	Health and hematologic traits	22	IVW	0.0335	0.0171	0.0497	0.0000	0.0635	1.0341	1.0000	1.0693
**ebi-a-GCST90001577**	Transitional B cell Absolute Count	acute RITs	European	Health and hematologic traits	22	IVW	0.0423	0.0181	0.0191	0.0069	0.0635	1.0433	1.0070	1.0808
**ukb-d-30000_irnt**	White blood cell (leukocyte) count	acute RITs	European	Health and hematologic traits	483	Weighted median	0.0800	0.0405	0.0482	0.0006	0.1593	1.0832	1.0006	1.1727
**ebi-a-GCST90002407**	White blood cell count	acute RITs	European	Health and hematologic traits	583	MR Egger	0.0858	0.0428	0.0454	0.0020	0.1696	1.0896	1.0020	1.1849
**ebi-a-GCST90018978**	White blood cell count	acute RITs	European	Health and hematologic traits	520	MR Egger	0.1058	0.0499	0.0346	0.0079	0.2037	1.1116	1.0080	1.2260
**ebi-a-GCST90025985**	White blood cell count	acute RITs	European	Health and hematologic traits	500	MR Egger	0.1065	0.0485	0.0286	0.0114	0.2016	1.1124	1.0115	1.2234
**ebi-a-GCST90001449**	CD11c+ monocyte %monocyte	acute RITs	European	Health and hematologic traits	15	MR Egger	-0.0719	0.0328	0.0470	-0.1362	-0.0077	0.9306	0.8727	0.9923
**ukb-e-4570_AFR**	Friendships satisfaction	acute RITs	African American or Afro-Caribbean	Health and hematologic traits	22	IVW	-0.0382	0.0154	0.0133	-0.0683	0.1014	0.9626	0.9339	0.9921
**ebi-a-GCST010779**	COVID-19 (hospitalized vs population) RELEASE 4	acute RITs	European	Infection and immunity	42	IVW	-0.0276	0.0118	0.0189	-0.0507	0.0635	0.9727	0.9505	0.9955
**ebi-a-GCST011082**	COVID-19 (hospitalized vs population) RELEASE 5	acute RITs	European	Infection and immunity	42	IVW	-0.0322	0.0137	0.0191	-0.0591	0.0635	0.9683	0.9426	0.9948
**ebi-a-GCST011084**	COVID-19 (hospitalized vs population) RELEASE 5	acute RITs	European	Infection and immunity	45	IVW	-0.0356	0.0152	0.0193	-0.0655	0.0635	0.9650	0.9366	0.9942
**ebi-a-GCST90006919**	Anti-herpes simplex virus 2 IgG seropositivity	acute RITs	European	Infection and immunity	18	IVW	0.1767	0.0863	0.0407	0.0075	0.0657	1.1933	1.0075	1.4133

**ebi-a-GCST90006921**	Anti-polyomavirus 2 IgG seropositivity	acute RITs	European	Infection and immunity	18	Weighted median	0.1497	0.0722	0.0380	0.0083	0.2911	1.1615	1.0083	1.3379
**prot-a-180**	Arf-GAP with SH3 domain, ANK repeat and PH domain-containing protein 2	acute RITs	European	Infection and immunity	22	IVW	0.0357	0.0131	0.0065	0.0100	0.0947	1.0364	1.0100	1.0634
**prot-a-204**	Aurora kinase A	acute RITs	European	Infection and immunity	18	IVW	0.0394	0.0188	0.0363	0.0025	0.1354	1.0402	1.0025	1.0792
**ebi-a-GCST90001829**	BAFF-R on B cell	acute RITs	European	Infection and immunity	14	IVW	-0.0220	0.0085	0.0100	-0.0387	0.0284	0.9783	0.9620	0.9948
**ebi-a-GCST90001830**	BAFF-R on CD20- B cell	acute RITs	European	Infection and immunity	6	Weighted mode	-0.1015	0.0352	0.0346	-0.1706	-0.0324	0.9035	0.8432	0.9681
**ebi-a-GCST90001702**	BAFF-R on CD24+ CD27+ B cell	acute RITs	European	Infection and immunity	13	IVW	-0.0210	0.0088	0.0170	-0.0383	0.0635	0.9792	0.9624	0.9963
**ebi-a-GCST90001710**	BAFF-R on IgD- CD24- B cell	acute RITs	European	Infection and immunity	14	IVW	-0.0233	0.0100	0.0204	-0.0430	0.0635	0.9770	0.9579	0.9964
**ebi-a-GCST90001711**	BAFF-R on IgD- CD27- B cell	acute RITs	European	Infection and immunity	12	IVW	-0.0191	0.0097	0.0483	-0.0381	0.0635	0.9811	0.9626	0.9999
**ebi-a-GCST90001712**	BAFF-R on IgD- CD38- B cell	acute RITs	European	Infection and immunity	13	IVW	-0.0180	0.0087	0.0382	-0.0351	0.0635	0.9821	0.9655	0.9990
**ebi-a-GCST90001713**	BAFF-R on IgD- CD38+ B cell	acute RITs	European	Infection and immunity	13	IVW	-0.0459	0.0176	0.0092	-0.0804	0.0635	0.9551	0.9227	0.9887
**ebi-a-GCST90001719**	BAFF-R on IgD+ B cell	acute RITs	European	Infection and immunity	17	IVW	-0.0241	0.0084	0.0040	-0.0405	0.0635	0.9762	0.9603	0.9924
**ebi-a-GCST90001704**	BAFF-R on IgD+ CD24- B cell	acute RITs	European	Infection and immunity	15	IVW	-0.0241	0.0085	0.0045	-0.0406	0.0635	0.9762	0.9602	0.9925
**ebi-a-GCST90001703**	BAFF-R on IgD+ CD24+ B cell	acute RITs	European	Infection and immunity	13	IVW	-0.0249	0.0087	0.0044	-0.0420	0.0635	0.9754	0.9589	0.9923
**ebi-a-GCST90001705**	BAFF-R on IgD+ CD38- B cell	acute RITs	European	Infection and immunity	16	IVW	-0.0260	0.0085	0.0024	-0.0427	0.0635	0.9744	0.9582	0.9908
**ebi-a-GCST90001708**	BAFF-R on IgD+ CD38+ B cell	acute RITs	European	Infection and immunity	18	IVW	-0.0243	0.0081	0.0027	-0.0401	0.0635	0.9760	0.9607	0.9916
**ebi-a-GCST90001709**	BAFF-R on IgD+ CD38dim B cell	acute RITs	European	Infection and immunity	15	IVW	-0.0198	0.0094	0.0351	-0.0382	0.0635	0.9804	0.9625	0.9986
**ebi-a-GCST90001715**	BAFF-R on memory B cell	acute RITs	European	Infection and immunity	10	IVW	-0.0247	0.0094	0.0083	-0.0430	0.0635	0.9756	0.9579	0.9937
**ebi-a-GCST90001716**	BAFF-R on naive-mature B cell	acute RITs	European	Infection and immunity	16	IVW	-0.0210	0.0084	0.0120	-0.0374	0.0635	0.9792	0.9632	0.9954
**ebi-a-GCST90001718**	BAFF-R on switched memory B cell	acute RITs	European	Infection and immunity	12	IVW	-0.0197	0.0090	0.0281	-0.0374	0.0635	0.9805	0.9633	0.9979
**ebi-a-GCST90001720**	BAFF-R on transitional B cell	acute RITs	European	Infection and immunity	18	IVW	-0.0231	0.0101	0.0226	-0.0429	0.0635	0.9772	0.9580	0.9968
**ebi-a-GCST90001717**	BAFF-R on unswitched memory B cell	acute RITs	European	Infection and immunity	16	IVW	-0.0201	0.0087	0.0205	-0.0371	0.0635	0.9801	0.9635	0.9969
**prot-a-231**	Basal Cell Adhesion Molecule	acute RITs	European	Infection and immunity	25	Weighted median	-0.0432	0.0207	0.0373	-0.0839	-0.0025	0.9577	0.9196	0.9975
**prot-a-218**	Beta-1,4-galactosyltransferase 3	acute RITs	European	Infection and immunity	10	IVW (fixed effects)	-0.0558	0.0243	0.0218	-0.1036	-0.0081	0.9457	0.9016	0.9919
**prot-a-800**	Beta-defensin 128	acute RITs	European	Infection and immunity	22	IVW	0.0393	0.0181	0.0300	0.0038	0.1558	1.0401	1.0038	1.0776
**prot-a-801**	Beta-defensin 134	acute RITs	European	Infection and immunity	21	MR Egger	0.1138	0.0524	0.0428	0.0111	0.2166	1.1206	1.0111	1.2418
**prot-a-1396**	Bone sialoprotein 2	acute RITs	European	Infection and immunity	18	IVW	-0.0401	0.0192	0.0370	-0.0777	0.0360	0.9607	0.9253	0.9976
**prot-a-2391**	Brain-specific serine protease 4	acute RITs	European	Infection and	16	IVW	-0.0633	0.0173	0.0003	-0.0972	0.0739	0.9386	0.9074	0.9710

				immunity										
**prot-a-232**	Brevican core protein	acute RITs	European	Infection and immunity	17	IVW	-0.0489	0.0220	0.0266	-0.0921	0.1619	0.9523	0.9120	0.9943
**prot-a-2947**	Calcineurin B homologous protein 3	acute RITs	European	Infection and immunity	15	IVW (fixed effects)	0.0439	0.0201	0.0291	0.0045	0.0833	1.0448	1.0045	1.0868
**prot-a-2509**	Calcipressin-1	acute RITs	European	Infection and immunity	19	IVW	0.0433	0.0219	0.0477	0.0004	0.1960	1.0443	1.0004	1.0901
**prot-a-347**	Calcium/calmodulin-dependent protein kinase type 1D	acute RITs	European	Infection and immunity	21	IVW	0.0526	0.0149	0.0004	0.0233	0.1888	1.0540	1.0236	1.0853
**prot-a-697**	Casein kinase II 2-alpha:2-beta heterotetramer	acute RITs	European	Infection and immunity	19	IVW	0.0435	0.0182	0.0168	0.0079	0.1557	1.0444	1.0079	1.0823
**prot-a-721**	Cathepsin F	acute RITs	European	Infection and immunity	18	IVW	0.0394	0.0191	0.0393	0.0019	0.1744	1.0402	1.0019	1.0800
**prot-a-723**	Cathepsin G	acute RITs	European	Infection and immunity	19	IVW	0.0426	0.0210	0.0425	0.0014	0.2021	1.0435	1.0014	1.0873
**prot-a-724**	Cathepsin H	acute RITs	European	Infection and immunity	28	IVW	-0.0327	0.0152	0.0310	-0.0624	0.0733	0.9678	0.9395	0.9970
**prot-a-728**	Cathepsin L2	acute RITs	European	Infection and immunity	19	IVW (fixed effects)	-0.0377	0.0188	0.0448	-0.0745	-0.0009	0.9630	0.9282	0.9991
**prot-a-368**	Caveolin-2	acute RITs	European	Infection and immunity	16	IVW (fixed effects)	0.0436	0.0200	0.0293	0.0044	0.0829	1.0446	1.0044	1.0864
**prot-a-398**	C-C motif chemokine 22	acute RITs	European	Infection and immunity	22	IVW	-0.0396	0.0166	0.0169	-0.0720	-0.0188	0.9612	0.9305	0.9929
**prot-a-409**	C-C motif chemokine 5	acute RITs	European	Infection and immunity	26	IVW	-0.0390	0.0148	0.0085	-0.0680	0.0545	0.9618	0.9343	0.9901
**ebi-a-GCST90001748**	CD20 on IgD+ CD38- B cell	acute RITs	European	Infection and immunity	21	MR Egger	-0.1009	0.0454	0.0387	-0.1900	-0.0119	0.9040	0.8270	0.9882
**ebi-a-GCST90001751**	CD20 on IgD+ CD38+ B cell	acute RITs	European	Infection and immunity	21	IVW	-0.0254	0.0121	0.0351	-0.0491	0.0060	0.9749	0.9521	0.9982
**ebi-a-GCST90001760**	CD20 on unswitched memory B cell	acute RITs	European	Infection and immunity	16	Weighted median	-0.0514	0.0241	0.0329	-0.0987	-0.0042	0.9499	0.9060	0.9958
**ebi-a-GCST90001760**	CD20 on unswitched memory B cell	acute RITs	European	Infection and immunity	16	Weighted mode	-0.0638	0.0282	0.0387	-0.1190	-0.0086	0.9382	0.8878	0.9914
**ebi-a-GCST90001910**	CD45 on B cell	acute RITs	European	Infection and immunity	16	MR Egger	0.0392	0.0174	0.0413	0.0050	0.0733	1.0399	1.0050	1.0761
**prot-a-3080**	Cellular tumor antigen p53	acute RITs	European	Infection and immunity	19	IVW	0.0524	0.0228	0.0216	0.0077	0.0970	1.0538	1.0077	1.1019
**prot-a-432**	CMRF35-like molecule 8	acute RITs	European	Infection and immunity	25	IVW	0.0268	0.0129	0.0377	0.0015	0.0773	1.0272	1.0015	1.0535
**prot-a-303**	Complement C1q tumor necrosis factor-related protein 1	acute RITs	European	Infection and immunity	20	Weighted median	-0.0296	0.0150	0.0479	-0.0589	-0.0003	0.9708	0.9428	0.9997
**prot-a-303**	Complement C1q tumor necrosis factor-related protein 1	acute RITs	European	Infection and immunity	20	Weighted mode	-0.0341	0.0149	0.0343	-0.0634	-0.0048	0.9665	0.9386	0.9952
**prot-a-300**	Complement component 1 Q subcomponent-binding protein, mitochondrial	acute RITs	European	Infection and immunity	28	MR Egger	-0.1271	0.0613	0.0482	-0.2473	-0.0069	0.8806	0.7809	0.9931
**ebi-a-GCST90019439**	Complement factor B measurement	acute RITs	European	Infection and immunity	28	IVW	0.0332	0.0152	0.0287	0.0035	0.1315	1.0337	1.0035	1.0649
**bbj-a-14**	C-reactive protein	acute RITs	East Asian	Infection and immunity	46	IVW	0.0837	0.0288	0.0036	0.0273	0.1747	1.0873	1.0277	1.1505
**ieu-a-1015**	C-reactive protein	acute RITs	East Asian	Infection and immunity	12	IVW (fixed	0.0601	0.0300	0.0448	0.0014	0.1188	1.0620	1.0014	1.1262

						effects)								
**prot-a-670**	C-reactive protein	acute RITs	European	Infection and immunity	21	IVW	0.0353	0.0157	0.0240	0.0047	0.1921	1.0360	1.0047	1.0683
**ieu-b-35**	C-Reactive protein level	acute RITs	European	Infection and immunity	130	IVW	0.0527	0.0216	0.0148	0.0103	0.1300	1.0542	1.0104	1.0998
**ebi-a-GCST90025959**	C-reactive protein levels	acute RITs	European	Infection and immunity	353	IVW	0.0684	0.0258	0.0081	0.0178	0.1315	1.0708	1.0179	1.1263
**prot-a-579**	C-type lectin domain family 5 member A	acute RITs	European	Infection and immunity	20	IVW	0.0352	0.0156	0.0240	0.0046	0.0658	1.0358	1.0046	1.0680
**ebi-a-GCST90002012**	CX3CR1 on CD14- CD16+ monocyte	acute RITs	European	Infection and immunity	19	IVW	0.0272	0.0137	0.0466	0.0004	0.0155	1.0275	1.0004	1.0554
**ebi-a-GCST90001997**	CX3CR1 on CD14+ CD16- monocyte	acute RITs	European	Infection and immunity	20	IVW	0.0219	0.0110	0.0467	0.0003	0.0155	1.0221	1.0003	1.0444
**ebi-a-GCST90001995**	CX3CR1 on monocyte	acute RITs	European	Infection and immunity	20	IVW	0.0326	0.0143	0.0230	0.0045	0.0155	1.0331	1.0045	1.0626
**prot-a-3175**	Cytochrome b-c1 complex subunit 7	acute RITs	European	Infection and immunity	18	IVW (fixed effects)	0.0370	0.0179	0.0392	0.0018	0.0721	1.0377	1.0018	1.0748
**prot-a-813**	Desert hedgehog protein N-product	acute RITs	European	Infection and immunity	14	IVW	0.0450	0.0208	0.0301	0.0043	0.0857	1.0460	1.0043	1.0895
**prot-a-824**	Dickkopf-like protein 1	acute RITs	European	Infection and immunity	16	IVW	-0.0401	0.0203	0.0484	-0.0798	0.1115	0.9607	0.9233	0.9997
**prot-a-586**	Dual specificity protein kinase CLK2	acute RITs	European	Infection and immunity	19	IVW (fixed effects)	0.0297	0.0135	0.0273	0.0033	0.0561	1.0302	1.0033	1.0577
**prot-a-2179**	E3 ubiquitin-protein ligase parkin	acute RITs	European	Infection and immunity	20	IVW (fixed effects)	0.0408	0.0179	0.0230	0.0056	0.0759	1.0416	1.0056	1.0788
**prot-a-2528**	E3 ubiquitin-protein ligase rififylin	acute RITs	European	Infection and immunity	13	IVW	0.0663	0.0325	0.0417	0.0025	0.1446	1.0685	1.0025	1.1389
**prot-a-3278**	E3 ubiquitin-protein ligase ZNRF3	acute RITs	European	Infection and immunity	12	IVW (fixed effects)	-0.0490	0.0225	0.0294	-0.0932	-0.0049	0.9521	0.9110	0.9951
**prot-a-984**	Endoplasmic reticulum resident protein 29	acute RITs	European	Infection and immunity	15	IVW	0.0493	0.0201	0.0144	0.0098	0.2449	1.0505	1.0099	1.0928
**prot-a-902**	Ephrin-B1	acute RITs	European	Infection and immunity	15	IVW	-0.0435	0.0217	0.0445	-0.0860	0.0988	0.9574	0.9176	0.9989
**prot-a-907**	Ephrin-B3	acute RITs	European	Infection and immunity	13	IVW (fixed effects)	0.0428	0.0180	0.0175	0.0075	0.0781	1.0437	1.0075	1.0813
**prot-a-2071**	Epididymal secretory protein E1	acute RITs	European	Infection and immunity	26	IVW	-0.0229	0.0106	0.0302	-0.0436	0.0027	0.9773	0.9573	0.9978
**prot-a-1835**	Epididymis-specific alpha-mannosidase	acute RITs	European	Infection and immunity	17	IVW	0.0427	0.0164	0.0091	0.0106	0.1311	1.0436	1.0107	1.0777
**prot-a-977**	Epiregulin	acute RITs	European	Infection and immunity	20	MR Egger	0.1414	0.0640	0.0405	0.0159	0.2669	1.1519	1.0160	1.3059
**prot-a-308**	ES1 protein homolog, mitochondrial	acute RITs	European	Infection and immunity	24	Weighted median	0.0645	0.0248	0.0092	0.0159	0.1130	1.0666	1.0161	1.1196
**prot-a-2892**	Estrogen sulfotransferase	acute RITs	European	Infection and immunity	21	IVW	0.0470	0.0155	0.0025	0.0166	0.0714	1.0482	1.0167	1.0806
**prot-a-1001**	Exosome complex component CSL4	acute RITs	European	Infection and immunity	23	Weighted median	-0.0545	0.0244	0.0255	-0.1023	-0.0067	0.9470	0.9028	0.9933
**prot-a-1003**	Exostosin-like 2	acute RITs	European	Infection and immunity	17	IVW	0.0498	0.0179	0.0054	0.0147	0.1988	1.0510	1.0148	1.0885
**prot-a-1019**	Fas apoptotic inhibitory	acute RITs	European	Infection and	28	IVW	0.0404	0.0146	0.0056	0.0118	0.1692	1.0412	1.0119	1.0714

	molecule 3			immunity									
**prot-a-1102**	Fibroblast growth factor receptor 2	acute RITs	European	Infection and immunity	25	IVW	-0.0463	0.0169	0.0062	-0.0794	0.0560	0.9548	0.9237	0.9869
**prot-a-1117**	Filamin-A	acute RITs	European	Infection and immunity	16	IVW	0.0433	0.0213	0.0421	0.0015	0.1940	1.0443	1.0015	1.0889
**prot-a-567**	Galectin-10	acute RITs	European	Infection and immunity	23	MR Egger	-0.1100	0.0499	0.0389	-0.2079	-0.0122	0.8958	0.8123	0.9879
**prot-a-1295**	Glucoside xylosyltransferase 1	acute RITs	European	Infection and immunity	23	IVW	0.0253	0.0126	0.0451	0.0006	0.0965	1.0256	1.0006	1.0513
**prot-a-1285**	Glutathione S-transferase A4	acute RITs	European	Infection and immunity	26	IVW	0.0453	0.0187	0.0155	0.0086	0.2271	1.0464	1.0086	1.0855
**ebi-a-GCST90002248**	Glycated hemoglobin levels	acute RITs	Sub-Saharan African	Infection and immunity	24	IVW (fixed effects)	0.0427	0.0201	0.0336	0.0033	0.0820	1.0436	1.0033	1.0855
**prot-a-1046**	Glycosaminoglycan xylosylkinase	acute RITs	European	Infection and immunity	26	IVW	0.0361	0.0149	0.0155	0.0069	0.1375	1.0367	1.0069	1.0674
**prot-a-1277**	Granulins	acute RITs	European	Infection and immunity	26	Weighted median	-0.0241	0.0123	0.0497	-0.0482	0.0000	0.9762	0.9529	1.0000
**prot-a-1268**	GRB2-related adapter protein	acute RITs	European	Infection and immunity	11	IVW	-0.0551	0.0249	0.0272	-0.1039	0.0472	0.9464	0.9013	0.9938
**prot-a-747**	Gro-beta/gamma	acute RITs	European	Infection and immunity	16	MR Egger	0.1848	0.0730	0.0240	0.0416	0.3279	1.2030	1.0425	1.3881
**prot-b-55**	growth differentiation factor 15	acute RITs	European	Infection and immunity	15	IVW	-0.0428	0.0140	0.0022	-0.0702	-0.0214	0.9581	0.9322	0.9847
**prot-a-1272**	Growth factor receptor-bound protein 7	acute RITs	European	Infection and immunity	21	IVW	0.0412	0.0167	0.0136	0.0085	0.2203	1.0420	1.0085	1.0766
**prot-a-1196**	Growth/differentiation factor 5	acute RITs	European	Infection and immunity	20	IVW	0.0304	0.0103	0.0032	0.0102	0.0652	1.0309	1.0102	1.0520
**prot-a-1197**	Growth/differentiation factor 9	acute RITs	European	Infection and immunity	19	IVW	0.0371	0.0187	0.0469	0.0005	0.0738	1.0378	1.0005	1.0765
**prot-a-1199**	GTP-binding protein GEM	acute RITs	European	Infection and immunity	20	IVW	0.0252	0.0091	0.0054	0.0075	0.0429	1.0255	1.0075	1.0439
**prot-a-1178**	Guanylate-binding protein 6	acute RITs	European	Infection and immunity	19	IVW	0.0424	0.0207	0.0406	0.0018	0.2230	1.0434	1.0018	1.0866
**prot-a-1387**	Heat shock 70 kDa protein 1-like	acute RITs	European	Infection and immunity	12	IVW	0.0422	0.0164	0.0103	0.0100	0.1768	1.0431	1.0100	1.0773
**prot-a-1310**	Hepatitis A virus cellular receptor 1	acute RITs	European	Infection and immunity	15	IVW	-0.0333	0.0151	0.0276	-0.0629	0.0191	0.9673	0.9390	0.9963
**prot-a-1366**	Heterogeneous nuclear ribonucleoprotein K	acute RITs	European	Infection and immunity	23	IVW (fixed effects)	0.0345	0.0168	0.0404	0.0015	0.0675	1.0351	1.0015	1.0698
**prot-a-2224**	High affinity cGMP-specific 3',5'-cyclic phosphodiesterase 9A	acute RITs	European	Infection and immunity	23	IVW	0.0374	0.0166	0.0249	0.0047	0.1053	1.0381	1.0047	1.0725
**prot-a-2121**	High affinity nerve growth factor receptor	acute RITs	European	Infection and immunity	12	IVW	0.0597	0.0252	0.0178	0.0103	0.3066	1.0615	1.0104	1.1153
**prot-a-1338**	Histidine triad nucleotide-binding protein 1	acute RITs	European	Infection and immunity	16	IVW	-0.0449	0.0181	0.0133	-0.0804	0.1054	0.9561	0.9228	0.9907
**prot-a-1607**	Histone acetyltransferase KAT6A	acute RITs	European	Infection and immunity	13	IVW	0.0408	0.0208	0.0497	0.0000	0.2188	1.0416	1.0000	1.0849
**prot-a-1304**	Histone H2A.z	acute RITs	European	Infection and immunity	15	MR Egger	-0.2024	0.0899	0.0423	-0.3786	-0.0262	0.8168	0.6848	0.9742
**ebi-a-GCST90002116**	HLA DR on B cell	acute RITs	European	Infection and immunity	14	IVW (fixed effects)	0.0216	0.0105	0.0402	0.0010	0.0423	1.0219	1.0010	1.0432

**prot-a-1393**	Hyaluronidase-1	acute RITs	European	Infection and immunity	22	IVW (fixed effects)	-0.0346	0.0168	0.0401	-0.0675	-0.0016	0.9660	0.9347	0.9984
**prot-a-1071**	Immunoglobulin alpha Fc receptor	acute RITs	European	Infection and immunity	8	IVW (fixed effects)	0.0715	0.0301	0.0176	0.0125	0.1304	1.0741	1.0125	1.1393
**prot-a-1576**	Immunoglobulin superfamily containing leucine-rich repeat protein 2	acute RITs	European	Infection and immunity	17	Weighted median	-0.0516	0.0218	0.0180	-0.0943	-0.0088	0.9497	0.9100	0.9912
**prot-a-2141**	Inositol polyphosphate 5-phosphatase OCRL-1	acute RITs	European	Infection and immunity	16	IVW	0.0508	0.0166	0.0022	0.0183	0.1359	1.0521	1.0184	1.0869
**prot-a-1593**	Inositol-trisphosphate 3-kinase A	acute RITs	European	Infection and immunity	11	IVW	0.0886	0.0240	0.0002	0.0416	0.3061	1.0927	1.0425	1.1453
**prot-a-1435**	Interferon lambda-1	acute RITs	European	Infection and immunity	14	Weighted median	0.0616	0.0311	0.0475	0.0007	0.1225	1.0635	1.0007	1.1303
**prot-a-1547**	Interleukin enhancer-binding factor 3	acute RITs	European	Infection and immunity	14	MR Egger	-0.1593	0.0654	0.0314	-0.2874	-0.0311	0.8528	0.7502	0.9694
**ebi-a-GCST004444**	Interleukin-10 levels	acute RITs	European	Infection and immunity	15	IVW	0.0596	0.0212	0.0049	0.0181	0.1767	1.0614	1.0182	1.1064
**prot-a-1479**	Interleukin-16	acute RITs	European	Infection and immunity	18	IVW	-0.0330	0.0140	0.0184	-0.0604	0.0363	0.9675	0.9414	0.9944
**ebi-a-GCST004448**	Interleukin-1-beta levels	acute RITs	European	Infection and immunity	5	IVW	-0.0966	0.0425	0.0230	-0.1799	0.1158	0.9079	0.8354	0.9868
**prot-a-1517**	Interleukin-27 receptor subunit alpha	acute RITs	European	Infection and immunity	14	IVW	-0.0413	0.0192	0.0317	-0.0790	0.0244	0.9595	0.9240	0.9964
**prot-a-1524**	Interleukin-34	acute RITs	European	Infection and immunity	24	IVW (fixed effects)	-0.0336	0.0155	0.0303	-0.0639	-0.0032	0.9670	0.9381	0.9968
**prot-a-1528**	Interleukin-36 gamma	acute RITs	European	Infection and immunity	20	IVW	0.0411	0.0186	0.0273	0.0046	0.1221	1.0419	1.0046	1.0807
**prot-a-1533**	Interleukin-4 receptor subunit alpha	acute RITs	European	Infection and immunity	19	IVW	0.0530	0.0183	0.0038	0.0171	0.1245	1.0545	1.0172	1.0931
**prot-a-1535**	Interleukin-5	acute RITs	European	Infection and immunity	16	IVW	-0.0453	0.0189	0.0166	-0.0823	0.0284	0.9557	0.9210	0.9918
**prot-a-1544**	Interleukin-7 receptor subunit alpha	acute RITs	European	Infection and immunity	26	Weighted median	-0.0476	0.0211	0.0244	-0.0890	-0.0061	0.9535	0.9148	0.9939
**prot-a-1595**	Intersectin-1	acute RITs	European	Infection and immunity	21	IVW	0.0394	0.0182	0.0306	0.0037	0.2757	1.0402	1.0037	1.0781
**prot-a-81**	Intestinal-type alkaline phosphatase	acute RITs	European	Infection and immunity	16	IVW	0.0388	0.0184	0.0351	0.0027	0.1057	1.0395	1.0027	1.0777
**ebi-a-GCST90012012**	kallikrein-11 levels	acute RITs	European	Infection and immunity	26	Weighted median	0.0475	0.0223	0.0333	0.0038	0.0912	1.0486	1.0038	1.0955
**prot-a-1664**	Kallikrein-5	acute RITs	European	Infection and immunity	12	IVW	-0.0498	0.0232	0.0317	-0.0952	0.1129	0.9515	0.9092	0.9956
**prot-a-1640**	Killer cell immunoglobulin-like receptor 2DL2	acute RITs	European	Infection and immunity	17	IVW	0.0332	0.0158	0.0357	0.0022	0.1440	1.0337	1.0022	1.0663
**prot-a-1635**	Kinesin-like protein KIF16B	acute RITs	European	Infection and immunity	25	IVW (fixed effects)	0.0323	0.0156	0.0391	0.0016	0.0629	1.0328	1.0016	1.0649
**prot-b-20**	KIT ligand	acute RITs	European	Infection and immunity	12	IVW	0.0104	0.0048	0.0308	0.0010	0.0223	1.0104	1.0010	1.0200
**prot-a-2825**	Kunitz-type protease inhibitor 3	acute RITs	European	Infection and immunity	25	Weighted median	0.0304	0.0149	0.0416	0.0012	0.0597	1.0309	1.0012	1.0616
**prot-a-2825**	Kunitz-type protease inhibitor 3	acute RITs	European	Infection and immunity	25	Weighted mode	0.0330	0.0148	0.0361	0.0039	0.0620	1.0335	1.0039	1.0640

**prot-a-1714**	Lactase-like protein	acute RITs	European	Infection and immunity	13	Weighted median	-0.0709	0.0327	0.0303	-0.1351	-0.0067	0.9315	0.8736	0.9933
**prot-a-1797**	Leucine-rich repeat transmembrane neuronal protein 4	acute RITs	European	Infection and immunity	19	MR Egger	-0.1349	0.0589	0.0349	-0.2503	-0.0196	0.8738	0.7786	0.9806
**prot-a-1076**	Low affinity immunoglobulin gamma Fc region receptor III-B	acute RITs	European	Infection and immunity	24	IVW	0.0466	0.0130	0.0003	0.0211	0.0918	1.0477	1.0213	1.0747
**prot-a-2666**	L-Selectin	acute RITs	European	Infection and immunity	22	Weighted median	-0.0412	0.0170	0.0154	-0.0745	-0.0079	0.9596	0.9282	0.9922
**prot-a-2666**	L-Selectin	acute RITs	European	Infection and immunity	22	Weighted mode	-0.0401	0.0181	0.0375	-0.0755	-0.0047	0.9607	0.9273	0.9953
**prot-a-1735**	Lutropin subunit beta	acute RITs	European	Infection and immunity	20	IVW	0.0385	0.0183	0.0349	0.0027	0.1098	1.0393	1.0027	1.0771
**prot-a-454**	Lymphocyte function-associated antigen 3	acute RITs	European	Infection and immunity	11	IVW	0.0614	0.0248	0.0132	0.0128	0.2657	1.0633	1.0129	1.1162
**prot-a-3236**	Lymphotactin	acute RITs	European	Infection and immunity	15	Weighted median	-0.0460	0.0221	0.0374	-0.0892	-0.0027	0.9551	0.9146	0.9973
**prot-a-1912**	Macrophage metalloelastase	acute RITs	European	Infection and immunity	16	IVW (fixed effects)	-0.0279	0.0136	0.0400	-0.0546	-0.0013	0.9724	0.9468	0.9987
**prot-a-1931**	MAGUK p55 subfamily member 6	acute RITs	European	Infection and immunity	17	IVW	0.0655	0.0159	0.0000	0.0343	0.1891	1.0677	1.0349	1.1015
**prot-a-1854**	MAP kinase-activated protein kinase 5	acute RITs	European	Infection and immunity	23	IVW	0.0173	0.0088	0.0494	0.0000	0.0518	1.0174	1.0000	1.0351
**prot-a-1916**	Matrix metalloproteinase-16	acute RITs	European	Infection and immunity	17	IVW	-0.0448	0.0194	0.0209	-0.0829	0.1221	0.9562	0.9205	0.9932
**prot-a-1896**	Melanoma-derived growth regulatory protein	acute RITs	European	Infection and immunity	17	IVW	0.0218	0.0097	0.0250	0.0027	0.0413	1.0221	1.0027	1.0418
**prot-a-1908**	Membrane metallo-endopeptidase-like 1	acute RITs	European	Infection and immunity	28	IVW	0.0448	0.0157	0.0043	0.0140	0.0781	1.0458	1.0141	1.0785
**ebi-a-GCST90006924**	Merkel cell polyomavirus VP1 antibody levels	acute RITs	European	Infection and immunity	15	IVW	-0.0467	0.0234	0.0456	-0.0926	0.0657	0.9543	0.9116	0.9991
**prot-a-1052**	MIP18 family protein FAM96A	acute RITs	European	Infection and immunity	14	IVW	-0.0522	0.0219	0.0169	-0.0950	0.1207	0.9491	0.9093	0.9907
**prot-a-5**	Monoacylglycerol lipase ABHD12	acute RITs	European	Infection and immunity	18	IVW	-0.0413	0.0211	0.0497	-0.0826	-0.0001	0.9595	0.9207	0.9999
**prot-b-75**	mucin 16, cell surface associated	acute RITs	European	Infection and immunity	12	Weighted median	-0.0428	0.0184	0.0202	-0.0790	-0.0067	0.9581	0.9241	0.9934
**prot-b-75**	mucin 16, cell surface associated	acute RITs	European	Infection and immunity	12	Weighted mode	-0.0459	0.0202	0.0445	-0.0855	-0.0062	0.9552	0.9181	0.9938
**prot-a-1967**	Mucin-1	acute RITs	European	Infection and immunity	22	IVW	0.0511	0.0132	0.0001	0.0251	0.1209	1.0524	1.0255	1.0800
**prot-a-1995**	N-acetyl-D-glucosamine kinase	acute RITs	European	Infection and immunity	17	IVW	-0.0309	0.0136	0.0229	-0.0575	0.0231	0.9696	0.9442	0.9957
**prot-a-2057**	NACHT, LRR and PYD domains-containing protein 4	acute RITs	European	Infection and immunity	18	Weighted median	-0.0741	0.0281	0.0084	-0.1292	-0.0190	0.9286	0.8788	0.9812
**prot-a-2026**	NADH dehydrogenase [ubiquinone] flavoprotein 2, mitochondrial	acute RITs	European	Infection and immunity	12	IVW	0.0504	0.0233	0.0304	0.0048	0.2542	1.0516	1.0048	1.1007
**prot-a-2081**	Neuronal pentraxin-2	acute RITs	European	Infection and immunity	17	MR Egger	0.1499	0.0689	0.0461	0.0148	0.2850	1.1617	1.0149	1.3297
**prot-a-345**	Neuron-specific vesicular protein calcyon	acute RITs	European	Infection and immunity	18	IVW	0.0454	0.0196	0.0205	0.0070	0.1685	1.0465	1.0070	1.0874
**prot-a-2099**	Neuropilin-1	acute RITs	European	Infection and immunity	25	MR Egger	-0.1040	0.0466	0.0356	-0.1952	-0.0127	0.9013	0.8227	0.9874

**prot-a-2050**	Nidogen-2	acute RITs	European	Infection and immunity	14	IVW	-0.0351	0.0157	0.0252	-0.0659	0.0315	0.9655	0.9362	0.9956
**prot-a-1674**	NKG2-D type II integral membrane protein	acute RITs	European	Infection and immunity	20	IVW	0.0361	0.0176	0.0401	0.0016	0.1321	1.0367	1.0016	1.0731
**prot-a-1872**	Nuclear protein MDM1	acute RITs	European	Infection and immunity	17	IVW	-0.0496	0.0235	0.0350	-0.0956	0.1428	0.9517	0.9088	0.9965
**prot-a-2086**	Nuclear receptor subfamily 1 group D member 2	acute RITs	European	Infection and immunity	21	MR Egger	-0.1250	0.0556	0.0365	-0.2340	-0.0161	0.8825	0.7914	0.9840
**prot-a-2093**	Nuclear receptor-binding protein	acute RITs	European	Infection and immunity	19	IVW	0.0415	0.0194	0.0326	0.0034	0.1515	1.0423	1.0034	1.0827
**prot-a-2150**	Oligophrenin-1	acute RITs	European	Infection and immunity	20	IVW	0.0523	0.0185	0.0047	0.0160	0.2282	1.0536	1.0162	1.0925
**prot-a-2155**	Oncostatin-M	acute RITs	European	Infection and immunity	22	MR Egger	0.1234	0.0524	0.0289	0.0207	0.2262	1.1314	1.0209	1.2538
**prot-a-2272**	Paired immunoglobulin-like type 2 receptor alpha	acute RITs	European	Infection and immunity	21	IVW	0.0364	0.0175	0.0378	0.0021	0.1489	1.0371	1.0021	1.0733
**prot-a-2344**	Peptidyl-prolyl cis-trans isomerase-like 2	acute RITs	European	Infection and immunity	21	IVW (fixed effects)	0.0345	0.0170	0.0421	0.0012	0.0677	1.0351	1.0012	1.0701
**prot-a-2425**	Phosphatidylinositol 3,4,5-trisphosphate 3-phosphatase and dual-specificity protein phosphatase PTEN	acute RITs	European	Infection and immunity	22	IVW	-0.0393	0.0168	0.0196	-0.0722	0.0552	0.9615	0.9303	0.9937
**prot-a-2294**	Phospholipase B-like 1	acute RITs	European	Infection and immunity	16	IVW	-0.0419	0.0202	0.0379	-0.0815	0.0839	0.9589	0.9217	0.9977
**prot-a-2698**	Pigment epithelium-derived factor	acute RITs	European	Infection and immunity	19	IVW	0.0297	0.0145	0.0401	0.0013	0.0885	1.0301	1.0013	1.0597
**prot-a-2299**	Pleckstrin homology domain-containing family A member 1	acute RITs	European	Infection and immunity	22	IVW	0.0623	0.0195	0.0014	0.0241	0.1945	1.0642	1.0244	1.1056
**prot-a-1171**	Polypeptide N-acetylgalactosaminyltransfer ase 2	acute RITs	European	Infection and immunity	20	IVW	-0.0416	0.0200	0.0375	-0.0807	0.1106	0.9593	0.9225	0.9976
**prot-a-1613**	Potassium voltage-gated channel subfamily E regulatory beta subunit 5	acute RITs	European	Infection and immunity	16	IVW	-0.0414	0.0194	0.0329	-0.0794	0.0700	0.9594	0.9236	0.9966
**prot-a-1615**	Potassium voltage-gated channel subfamily G member 4	acute RITs	European	Infection and immunity	21	IVW	0.0589	0.0204	0.0039	0.0189	0.2358	1.0607	1.0191	1.1039
**prot-a-2209**	Procollagen C-endopeptidase enhancer 1	acute RITs	European	Infection and immunity	20	MR Egger	-0.1115	0.0486	0.0340	-0.2067	-0.0163	0.8945	0.8132	0.9839
**prot-a-2409**	Proteasome subunit alpha type-1	acute RITs	European	Infection and immunity	17	IVW (fixed effects)	-0.0419	0.0204	0.0399	-0.0819	-0.0019	0.9589	0.9214	0.9981
**prot-a-827**	Protein delta homolog 1	acute RITs	European	Infection and immunity	19	IVW	0.0298	0.0140	0.0335	0.0023	0.0458	1.0303	1.0023	1.0590
**prot-a-810**	Protein DGCR6	acute RITs	European	Infection and immunity	21	MR Egger	0.1425	0.0658	0.0431	0.0136	0.2714	1.1532	1.0137	1.3118
**prot-a-2232**	Protein disulfide-isomerase A3	acute RITs	European	Infection and immunity	23	IVW	0.0380	0.0172	0.0275	0.0042	0.1262	1.0387	1.0042	1.0743
**prot-a-2233**	Protein disulfide-isomerase A5	acute RITs	European	Infection and immunity	26	IVW (fixed effects)	0.0245	0.0120	0.0415	0.0009	0.0481	1.0249	1.0009	1.0493
**prot-a-1027**	Protein FAM163A	acute RITs	European	Infection and immunity	23	IVW (fixed	-0.0285	0.0127	0.0253	-0.0535	-0.0035	0.9719	0.9479	0.9965

						effects)								
**prot-a-1049**	Protein FAM3B	acute RITs	European	Infection and immunity	12	IVW	0.0416	0.0195	0.0325	0.0035	0.0885	1.0425	1.0035	1.0830
**prot-a-2619**	Protein S100-A5	acute RITs	European	Infection and immunity	24	IVW	0.0245	0.0122	0.0453	0.0005	0.0863	1.0248	1.0005	1.0496
**prot-a-3093**	Protein-tyrosine sulfotransferase 2	acute RITs	European	Infection and immunity	26	Weighted median	0.0426	0.0200	0.0330	0.0034	0.0817	1.0435	1.0034	1.0851
**prot-a-3093**	Protein-tyrosine sulfotransferase 2	acute RITs	European	Infection and immunity	26	Weighted mode	0.0614	0.0254	0.0233	0.0116	0.1112	1.0633	1.0117	1.1176
**prot-a-2203**	Protocadherin beta-1	acute RITs	European	Infection and immunity	18	MR Egger	0.1147	0.0523	0.0435	0.0121	0.2172	1.1215	1.0122	1.2426
**prot-a-2207**	Protocadherin gamma-C5	acute RITs	European	Infection and immunity	21	MR Egger	-0.1281	0.0581	0.0400	-0.2419	-0.0142	0.8798	0.7851	0.9859
**prot-a-2191**	Pterin-4-alpha-carbinolamine dehydratase	acute RITs	European	Infection and immunity	17	IVW	-0.0439	0.0191	0.0212	-0.0813	0.0514	0.9570	0.9219	0.9934
**prot-a-2711**	Pulmonary surfactant-associated protein C	acute RITs	European	Infection and immunity	25	IVW	-0.0425	0.0128	0.0009	-0.0675	0.0408	0.9584	0.9347	0.9827
**prot-a-2477**	Ras-related protein Rab-39B	acute RITs	European	Infection and immunity	20	IVW (fixed effects)	0.0409	0.0178	0.0218	0.0059	0.0758	1.0417	1.0060	1.0788
**prot-a-2444**	Receptor-type tyrosine-protein phosphatase H	acute RITs	European	Infection and immunity	18	IVW	-0.0621	0.0166	0.0002	-0.0947	-0.0475	0.9398	0.9097	0.9710
**prot-a-2524**	Resistin	acute RITs	European	Infection and immunity	21	IVW (fixed effects)	0.0354	0.0163	0.0294	0.0035	0.0673	1.0361	1.0035	1.0696
**prot-a-2497**	Retinoblastoma-binding protein 5	acute RITs	European	Infection and immunity	14	IVW	-0.0424	0.0200	0.0343	-0.0817	0.2032	0.9585	0.9216	0.9969
**prot-a-2602**	Ribonucleoside-diphosphate reductase subunit M2 B	acute RITs	European	Infection and immunity	15	IVW (fixed effects)	0.0428	0.0192	0.0258	0.0052	0.0805	1.0437	1.0052	1.0838
**prot-a-1945**	Ribosome-recycling factor, mitochondrial	acute RITs	European	Infection and immunity	21	IVW	0.0429	0.0179	0.0162	0.0079	0.0912	1.0439	1.0080	1.0811
**prot-a-2584**	Ribulose-phosphate 3-epimerase	acute RITs	European	Infection and immunity	12	IVW (fixed effects)	-0.0394	0.0201	0.0497	-0.0788	-0.0001	0.9613	0.9242	0.9999
**prot-a-2569**	RING finger protein 215	acute RITs	European	Infection and immunity	19	MR Egger	0.1080	0.0454	0.0292	0.0191	0.1969	1.1141	1.0193	1.2176
**prot-a-2575**	rRNA methyltransferase 3, mitochondrial	acute RITs	European	Infection and immunity	20	IVW	0.0361	0.0106	0.0007	0.0153	0.0908	1.0367	1.0154	1.0585
**prot-a-2641**	Scavenger receptor class F member 2	acute RITs	European	Infection and immunity	21	IVW	0.0403	0.0191	0.0348	0.0029	0.1063	1.0411	1.0029	1.0809
**prot-a-2642**	Scavenger receptor class F member 2	acute RITs	European	Infection and immunity	19	IVW	-0.0513	0.0175	0.0033	-0.0856	0.0491	0.9500	0.9179	0.9831
**prot-a-2709**	Secreted frizzled-related protein 1	acute RITs	European	Infection and immunity	21	IVW	0.0518	0.0176	0.0032	0.0173	0.1357	1.0531	1.0175	1.0900
**prot-a-2669**	Semaphorin-3A	acute RITs	European	Infection and immunity	25	IVW	0.0461	0.0138	0.0008	0.0191	0.1559	1.0472	1.0193	1.0760
**prot-a-2671**	Semaphorin-3C	acute RITs	European	Infection and immunity	12	IVW	-0.0553	0.0228	0.0153	-0.1000	0.0205	0.9462	0.9048	0.9894
**prot-a-2838**	Serine/arginine-rich splicing factor 6	acute RITs	European	Infection and immunity	12	IVW (fixed effects)	0.0507	0.0247	0.0403	0.0023	0.0992	1.0520	1.0023	1.1043
**prot-a-2170**	Serine/threonine-protein kinase PAK 4	acute RITs	European	Infection and immunity	13	IVW	-0.0426	0.0207	0.0396	-0.0832	0.0570	0.9583	0.9202	0.9980
**prot-a-2690**	Serpin A12	acute RITs	European	Infection and	26	IVW	-0.0383	0.0184	0.0373	-0.0744	0.0925	0.9624	0.9283	0.9978

				immunity										
**prot-a-2730**	Sialic acid-binding Ig-like lectin 8	acute RITs	European	Infection and immunity	23	IVW	0.0333	0.0146	0.0229	0.0046	0.1020	1.0338	1.0046	1.0639
**prot-a-2655**	Signal peptide, CUB and EGF-like domain-containing protein 1	acute RITs	European	Infection and immunity	15	IVW (fixed effects)	-0.0442	0.0205	0.0311	-0.0843	-0.0040	0.9568	0.9191	0.9960
**prot-a-2775**	Single-pass membrane and coiled-coil domain-containing protein 2	acute RITs	European	Infection and immunity	16	IVW	0.0564	0.0220	0.0105	0.0132	0.1755	1.0580	1.0133	1.1047
**prot-a-2714**	Small glutamine-rich tetratricopeptide repeat-containing protein alpha	acute RITs	European	Infection and immunity	20	IVW	-0.0445	0.0182	0.0145	-0.0802	0.0198	0.9565	0.9229	0.9912
**prot-a-2763**	Sodium- and chloride-dependent glycine transporter 1	acute RITs	European	Infection and immunity	13	IVW (fixed effects)	0.0483	0.0224	0.0311	0.0044	0.0923	1.0495	1.0044	1.0967
**prot-a-200**	Sodium/potassium-transporting ATPase subunit beta-2	acute RITs	European	Infection and immunity	19	IVW	-0.0341	0.0161	0.0338	-0.0656	0.0290	0.9665	0.9365	0.9974
**prot-a-352**	Soluble calcium-activated nucleotidase 1	acute RITs	European	Infection and immunity	17	IVW (fixed effects)	0.0401	0.0204	0.0498	0.0000	0.0802	1.0409	1.0000	1.0835
**prot-a-2795**	Sorting nexin-7	acute RITs	European	Infection and immunity	24	IVW	0.0413	0.0168	0.0139	0.0084	0.1712	1.0422	1.0084	1.0771
**prot-a-2804**	Sperm acrosome membrane-associated protein 3	acute RITs	European	Infection and immunity	19	IVW	-0.0325	0.0159	0.0413	-0.0637	-0.0156	0.9680	0.9382	0.9987
**prot-b-68**	spondin 1	acute RITs	European	Infection and immunity	4	IVW	0.0202	0.0101	0.0443	0.0005	0.0401	1.0204	1.0005	1.0407
**ebi-a-GCST90002074**	SSC-A on CD14+ monocyte	acute RITs	European	Infection and immunity	19	IVW	-0.0224	0.0114	0.0498	-0.0449	0.0155	0.9778	0.9561	1.0000
**ebi-a-GCST90002081**	SSC-A on CD4+ T cell	acute RITs	European	Infection and immunity	20	Weighted median	-0.0480	0.0242	0.0468	-0.0953	-0.0007	0.9531	0.9091	0.9993
**ebi-a-GCST90002079**	SSC-A on lymphocyte	acute RITs	European	Infection and immunity	17	Weighted median	-0.0553	0.0275	0.0446	-0.1092	-0.0013	0.9462	0.8965	0.9987
**ebi-a-GCST90019393**	Stromal cell-derived factor 2 measurement	acute RITs	European	Infection and immunity	15	IVW (fixed effects)	0.0542	0.0219	0.0134	0.0112	0.0972	1.0557	1.0113	1.1021
**prot-a-2468**	Sulfhydryl oxidase 1	acute RITs	European	Infection and immunity	16	IVW	0.0457	0.0194	0.0186	0.0076	0.1828	1.0468	1.0077	1.0874
**prot-a-2894**	Sulfotransferase 4A1	acute RITs	European	Infection and immunity	20	IVW	0.0447	0.0197	0.0235	0.0060	0.1386	1.0457	1.0060	1.0869
**prot-a-2901**	Sushi domain-containing protein 1	acute RITs	European	Infection and immunity	22	IVW	0.0360	0.0170	0.0349	0.0025	0.0880	1.0366	1.0025	1.0718
**prot-a-2885**	Syntaxin-1A	acute RITs	European	Infection and immunity	21	IVW (fixed effects)	-0.0290	0.0147	0.0479	-0.0577	-0.0003	0.9714	0.9439	0.9997
**prot-a-2933**	TATA-box-binding protein	acute RITs	European	Infection and immunity	19	IVW (fixed effects)	0.0412	0.0181	0.0227	0.0058	0.0767	1.0421	1.0058	1.0797
**prot-a-3068**	Tenascin-R	acute RITs	European	Infection and immunity	22	IVW	0.0481	0.0158	0.0024	0.0171	0.1250	1.0492	1.0172	1.0823
**prot-a-3084**	Thiamin pyrophosphokinase 1	acute RITs	European	Infection and immunity	17	IVW	-0.0537	0.0211	0.0111	-0.0951	-0.0122	0.9477	0.9093	0.9878
**prot-a-3102**	Thyrotropin-releasing hormone	acute RITs	European	Infection and immunity	23	IVW (fixed effects)	-0.0355	0.0172	0.0388	-0.0691	-0.0018	0.9652	0.9332	0.9982
**prot-a-3076**	Torsin-1A-interacting protein 1	acute RITs	European	Infection and	19	IVW	0.0280	0.0142	0.0483	0.0002	0.1247	1.0284	1.0002	1.0574

				immunity										
**prot-a-3115**	TPA-induced transmembrane protein	acute RITs	European	Infection and immunity	12	IVW	-0.0486	0.0247	0.0488	-0.0970	0.1896	0.9525	0.9075	0.9997
**prot-a-2963**	Transforming growth factor beta-1-induced transcript 1 protein	acute RITs	European	Infection and immunity	20	IVW (fixed effects)	0.0390	0.0177	0.0278	0.0043	0.0737	1.0398	1.0043	1.0765
**prot-a-918**	Translation initiation factor eIF-2B subunit alpha	acute RITs	European	Infection and immunity	13	IVW (fixed effects)	-0.0405	0.0193	0.0361	-0.0784	-0.0026	0.9603	0.9246	0.9974
**prot-a-2844**	Translocon-associated protein subunit alpha	acute RITs	European	Infection and immunity	14	MR Egger	0.1558	0.0688	0.0428	0.0210	0.2906	1.1686	1.0212	1.3373
**prot-a-2992**	Transmembrane and coiled-coil domain-containing protein 5A	acute RITs	European	Infection and immunity	29	IVW	0.0307	0.0150	0.0414	0.0012	0.2822	1.0312	1.0012	1.0620
**prot-a-2993**	Transmembrane emp24 domain-containing protein 10	acute RITs	European	Infection and immunity	16	IVW	-0.0299	0.0137	0.0295	-0.0568	-0.0183	0.9706	0.9448	0.9970
**prot-a-285**	Transmembrane protein C16orf54	acute RITs	European	Infection and immunity	17	IVW	0.0456	0.0209	0.0291	0.0046	0.1749	1.0467	1.0046	1.0905
**prot-a-1883**	tRNA (guanine-N(7)-)-methyltransferase	acute RITs	European	Infection and immunity	17	IVW (fixed effects)	0.0330	0.0166	0.0470	0.0004	0.0656	1.0336	1.0004	1.0678
**prot-a-3029**	Tumor necrosis factor	acute RITs	European	Infection and immunity	16	MR Egger	-0.1416	0.0654	0.0483	-0.2698	-0.0133	0.8680	0.7635	0.9868
**prot-a-3059**	Tumor necrosis factor ligand superfamily member 15	acute RITs	European	Infection and immunity	22	IVW	0.0392	0.0177	0.0262	0.0046	0.1348	1.0400	1.0047	1.0766
**prot-a-3128**	TYMS opposite strand protein	acute RITs	European	Infection and immunity	19	IVW (fixed effects)	0.0392	0.0191	0.0396	0.0019	0.0766	1.0400	1.0019	1.0796
**prot-a-3129**	Tyrosine-protein kinase receptor TYRO3	acute RITs	European	Infection and immunity	21	MR Egger	-0.1250	0.0584	0.0455	-0.2395	-0.0106	0.8825	0.7870	0.9895
**prot-a-2582**	Tyrosine-protein kinase transmembrane receptor ROR2	acute RITs	European	Infection and immunity	26	IVW	-0.0312	0.0152	0.0408	-0.0610	-0.0209	0.9693	0.9408	0.9987
**prot-a-598**	UMP-CMP kinase	acute RITs	European	Infection and immunity	18	IVW (fixed effects)	-0.0455	0.0188	0.0155	-0.0824	-0.0087	0.9555	0.9209	0.9914
**prot-a-295**	Uncharacterized protein C1orf115	acute RITs	European	Infection and immunity	25	IVW	0.0334	0.0162	0.0390	0.0017	0.1656	1.0340	1.0017	1.0673
**prot-a-311**	Uncharacterized protein C2orf66	acute RITs	European	Infection and immunity	18	IVW (fixed effects)	-0.0336	0.0155	0.0299	-0.0640	-0.0033	0.9669	0.9380	0.9967
**prot-a-3155**	UTP--glucose-1-phosphate uridylyltransferase	acute RITs	European	Infection and immunity	13	IVW	0.0522	0.0225	0.0207	0.0080	0.2432	1.0535	1.0080	1.1011
**prot-a-3197**	Vascular endothelial growth factor A, isoform 121	acute RITs	European	Infection and immunity	19	Weighted median	0.0271	0.0117	0.0202	0.0042	0.0500	1.0275	1.0042	1.0513
**prot-a-3197**	Vascular endothelial growth factor A, isoform 121	acute RITs	European	Infection and immunity	19	Weighted mode	0.0266	0.0126	0.0491	0.0019	0.0513	1.0270	1.0019	1.0527
**ebi-a-GCST004422**	Vascular endothelial growth factor levels	acute RITs	European	Infection and immunity	16	Weighted median	0.0368	0.0173	0.0340	0.0028	0.0708	1.0375	1.0028	1.0733
**ebi-a-GCST90011995**	Vascular endothelial growth factor levels	acute RITs	European	Infection and immunity	17	MR Egger	0.0551	0.0242	0.0375	0.0078	0.1024	1.0567	1.0078	1.1079
**ebi-a-GCST90011995**	Vascular endothelial growth factor levels	acute RITs	European	Infection and immunity	17	Weighted median	0.0442	0.0188	0.0190	0.0073	0.0811	1.0452	1.0073	1.0844
**ebi-a-GCST90011995**	Vascular endothelial growth factor levels	acute RITs	European	Infection and immunity	17	Weighted mode	0.0426	0.0189	0.0387	0.0055	0.0796	1.0435	1.0055	1.0829
**prot-a-3212**	Vesicle transport through interaction with t-SNAREs	acute RITs	European	Infection and immunity	20	IVW	-0.0416	0.0180	0.0207	-0.0768	0.0827	0.9593	0.9260	0.9937

	homolog 1A													
**prot-a-1179**	Vitamin D-binding protein	acute RITs	European	Infection and immunity	17	IVW (fixed effects)	0.0416	0.0204	0.0415	0.0016	0.0815	1.0424	1.0016	1.0850
**prot-a-3230**	Wnt inhibitory factor 1	acute RITs	European	Infection and immunity	17	IVW	-0.0489	0.0214	0.0221	-0.0909	0.0058	0.9522	0.9132	0.9930
**prot-a-3231**	WNT1-inducible-signaling pathway protein 1	acute RITs	European	Infection and immunity	17	IVW	-0.0244	0.0118	0.0382	-0.0475	0.0484	0.9759	0.9536	0.9987
**prot-a-3276**	Zinc finger protein 774	acute RITs	European	Infection and immunity	27	IVW	0.0334	0.0160	0.0372	0.0020	0.0642	1.0339	1.0020	1.0669
**prot-a-208**	Zinc-alpha-2-glycoprotein	acute RITs	European	Infection and immunity	16	MR Egger	0.1106	0.0486	0.0391	0.0153	0.2059	1.1170	1.0155	1.2286
**ebi-a-GCST90014002**	C reactive protein levels (UKB data field 30710)	acute RITs	European	Infection and immunity	392	IVW	0.0626	0.0246	0.0110	0.0143	0.1315	1.0646	1.0144	1.1172
**ukb-a-28**	Average weekly beer plus cider intake	acute RITs	European	Lifestyle, occupation and family background	89	MR Egger	0.8231	0.3534	0.0222	0.1303	1.5158	2.2775	1.1392	4.5532
**ukb-b-1707**	Average weekly spirits intake	acute RITs	European	Lifestyle, occupation and family background	54	IVW	-0.2919	0.1250	0.0195	-0.5369	0.9849	0.7468	0.5845	0.9542
**ukb-b-10054**	Cheese consumers	acute RITs	European	Lifestyle, occupation and family background	21	IVW	0.4135	0.1732	0.0170	0.0740	0.7902	1.5122	1.0768	2.1235
**ukb-b-1814**	Cheesecake intake	acute RITs	European	Lifestyle, occupation and family background	23	IVW (fixed effects)	-0.5563	0.2696	0.0391	-1.0847	-0.0279	0.5733	0.3380	0.9725
**ukb-b-1160**	Chocolate-covered raisin intake	acute RITs	European	Lifestyle, occupation and family background	27	MR Egger	1.4227	0.5772	0.0209	0.2914	2.5540	4.1484	1.3384	12.8582
**ukb-b-8089**	Cooked vegetable intake	acute RITs	European	Lifestyle, occupation and family background	120	IVW	-0.2135	0.1036	0.0394	-0.4165	0.9234	0.8078	0.6593	0.9896
**ukb-b-5382**	Eggs in sandwiches intake	acute RITs	European	Lifestyle, occupation and family background	13	IVW	-0.8319	0.3794	0.0283	-1.5755	-0.0883	0.4352	0.2069	0.9154
**ukb-b-14351**	Grapefruit juice intake	acute RITs	European	Lifestyle, occupation and family background	22	IVW (fixed effects)	-0.5174	0.2487	0.0375	-1.0049	-0.0298	0.5961	0.3661	0.9706
**ukb-b-18079**	Low fat hard cheese intake	acute RITs	European	Lifestyle, occupation and family background	11	MR Egger	1.7742	0.7628	0.0451	0.2791	3.2693	5.8954	1.3219	26.2921
**ukb-d-1418_1**	Milk type used: Full cream	acute RITs	European	Lifestyle, occupation and family background	38	MR Egger	5.0999	2.3503	0.0367	0.4933	9.7066	164.0112	1.6377	16425.0411
**ukb-d-1418_5**	Milk type used: Other type of milk	acute RITs	European	Lifestyle, occupation and	15	IVW (fixed	4.9530	2.1399	0.0206	0.7589	9.1471	141.6037	2.1360	9387.5627

				family background		effects)								
**ukb-e-6179_p3_CSA**	Mineral and other dietary supplements	acute RITs	South Asian	Lifestyle, occupation and family background	12	IVW	0.0416	0.0109	0.0001	0.0202	0.1107	1.0424	1.0204	1.0649
**ukb-e-1389_AFR**	Pork intake	acute RITs	African American or Afro-Caribbean	Lifestyle, occupation and family background	23	IVW	-0.0480	0.0177	0.0066	-0.0826	-0.0272	0.9531	0.9207	0.9867
**ukb-b-8006**	Poultry intake	acute RITs	European	Lifestyle, occupation and family background	93	MR Egger	0.9378	0.4339	0.0333	0.0874	1.7882	2.5544	1.0913	5.9790
**ukb-b-337**	Pure fruit/vegetable juice intake	acute RITs	European	Lifestyle, occupation and family background	17	IVW	-0.2804	0.1281	0.0287	-0.5316	0.8621	0.7555	0.5877	0.9712
**ukb-e-1478_AFR**	Salt added to food	acute RITs	African American or Afro-Caribbean	Lifestyle, occupation and family background	20	IVW	-0.0423	0.0200	0.0347	-0.0816	0.0590	0.9586	0.9216	0.9970
**ukb-b-5847**	Sausage intake	acute RITs	European	Lifestyle, occupation and family background	13	IVW	-0.3442	0.1456	0.0181	-0.6296	-0.0588	0.7088	0.5328	0.9429
**ukb-b-998**	Soya dessert intake	acute RITs	European	Lifestyle, occupation and family background	23	IVW	1.7758	0.5492	0.0012	0.6993	3.5987	5.9051	2.0123	17.3283
**ukb-b-12067**	Sponge pudding intake	acute RITs	European	Lifestyle, occupation and family background	17	IVW	1.0635	0.3825	0.0054	0.3138	2.4618	2.8964	1.3686	6.1299
**ukb-b-15984**	Thickness of butter/margarine spread on sliced bread: thin	acute RITs	European	Lifestyle, occupation and family background	16	IVW	0.5162	0.2368	0.0293	0.0520	3.5807	1.6757	1.0534	2.6657
**ukb-b-16523**	Type of meals eaten: Bought sandwiches	acute RITs	European	Lifestyle, occupation and family background	5	IVW (fixed effects)	-1.1404	0.5372	0.0338	-2.1934	-0.0874	0.3197	0.1115	0.9163
**ukb-b-6448**	Type of sliced bread eaten: mixed	acute RITs	European	Lifestyle, occupation and family background	18	IVW	-0.4512	0.2233	0.0433	-0.8889	-0.0136	0.6368	0.4111	0.9865
**ukb-b-3828**	Types of spread used on bread/crackers: Normal fat polyunsaturated margarine on bread/crackers	acute RITs	European	Lifestyle, occupation and family background	5	IVW	-1.9353	0.8543	0.0235	-3.6098	-0.2607	0.1444	0.0271	0.7705
**ukb-b-11725**	Types of spreads/sauces consumed: Tomato-based sauce	acute RITs	European	Lifestyle, occupation and family background	4	IVW	1.7359	0.8742	0.0471	0.0225	11.8375	5.6741	1.0227	31.4803
**ukb-b-3383**	Vegetarian sausages/burgers intake	acute RITs	European	Lifestyle, occupation and	22	IVW	-0.6777	0.2948	0.0215	-1.2554	-0.2232	0.5078	0.2850	0.9049

				family background										
**ukb-b-5427**	White fish intake	acute RITs	European	Lifestyle, occupation and family background	7	IVW	-0.7400	0.3263	0.0233	-1.3794	-0.1005	0.4771	0.2517	0.9044
**ukb-b-2375**	Whole-wheat cereal intake	acute RITs	European	Lifestyle, occupation and family background	17	IVW	0.2940	0.1267	0.0203	0.0457	0.5423	1.3418	1.0468	1.7200
**ukb-b-7753**	Yogurt intake	acute RITs	European	Lifestyle, occupation and family background	11	IVW (fixed effects)	-0.2525	0.1155	0.0288	-0.4788	-0.0261	0.7769	0.6195	0.9742
**ukb-e-6164_p6_CSA**	Types of physical activity in last 4 weeks	acute RITs	South Asian	Lifestyle, occupation and family background	7	IVW	0.0314	0.0144	0.0288	0.0033	0.1165	1.0319	1.0033	1.0614
**ukb-d-22612_2**	Worked with materials containing asbestos: Often	acute RITs	European	Lifestyle, occupation and family background	16	IVW	2.5025	0.8295	0.0026	0.8766	4.9645	12.2125	2.4028	62.0713
**ukb-d-22609_0**	Workplace very dusty: Rarely/never	acute RITs	European	Lifestyle, occupation and family background	19	IVW	-0.8816	0.3183	0.0056	-1.5055	0.1516	0.4141	0.2219	0.7728
**ukb-e-20107_p11_CSA**	Illnesses of father	acute RITs	South Asian	Lifestyle, occupation and family background	18	IVW (fixed effects)	-0.0112	0.0056	0.0468	-0.0221	-0.0002	0.9889	0.9781	0.9998
**ukb-a-201**	Illnesses of father: Heart disease	acute RITs	European	Lifestyle, occupation and family background	67	IVW	0.3256	0.1641	0.0472	0.0040	0.6972	1.3849	1.0040	1.9102
**ukb-a-202**	Illnesses of father: None of the above (group 1)	acute RITs	European	Lifestyle, occupation and family background	33	IVW	-0.4900	0.2444	0.0450	-0.9690	-0.0109	0.6126	0.3794	0.9892
**ukb-b-15169**	Illnesses of father: None of the above (group 1)	acute RITs	European	Lifestyle, occupation and family background	45	IVW	-0.5935	0.2913	0.0416	-1.1645	1.8084	0.5524	0.3121	0.9778
**ukb-b-10415**	Illnesses of father: None of the above (group 2)	acute RITs	European	Lifestyle, occupation and family background	36	IVW	0.6426	0.3079	0.0368	0.0392	2.0214	1.9015	1.0400	3.4766
**ukb-b-4024**	Illnesses of mother: Stroke	acute RITs	European	Lifestyle, occupation and family background	19	IVW	1.6110	0.6089	0.0082	0.4175	2.8045	5.0079	1.5182	16.5196
**ukb-b-18042**	Illnesses of siblings: Diabetes	acute RITs	European	Lifestyle, occupation and family background	80	IVW	0.5700	0.2856	0.0460	0.0102	2.2971	1.7682	1.0102	3.0948
**ukb-b-10783**	Illnesses of siblings: None of the above (group 2)	acute RITs	European	Lifestyle, occupation and	35	IVW	-0.7403	0.3594	0.0394	-1.4447	1.2650	0.4770	0.2358	0.9647

				family background										
**ukb-a-304**	Number of children fathered	acute RITs	European	Lifestyle, occupation and family background	33	IVW	-0.2582	0.1136	0.0231	-0.4809	0.3153	0.7724	0.6182	0.9652
**ebi-a-GCST006702**	Parental longevity (combined parental age at death)	acute RITs	European	Lifestyle, occupation and family background	60	IVW	-0.1838	0.0716	0.0102	-0.3241	0.2398	0.8321	0.7232	0.9574
**ebi-a-GCST006699**	Parental longevity (mother's age at death)	acute RITs	European	Lifestyle, occupation and family background	36	IVW	0.2275	0.1106	0.0397	0.0108	1.1203	1.2555	1.0108	1.5593
**ebi-a-GCST006696**	Parental longevity (mother's attained age)	acute RITs	European	Lifestyle, occupation and family background	55	IVW	-0.1951	0.0974	0.0452	-0.3859	-0.1457	0.8228	0.6798	0.9958
**ukb-a-406**	Current employment status: Looking after home and/or family	acute RITs	European	Lifestyle, occupation and family background	17	IVW	1.8398	0.9381	0.0499	0.0011	4.4174	6.2956	1.0011	39.5910
**ukb-d-22601_41123205**	Job coding: civil service administrative officer or assistant or clerk, tax collector, prison service clerk, law courts clerk	acute RITs	European	Lifestyle, occupation and family background	26	IVW	-0.8542	0.4024	0.0338	-1.6429	2.9368	0.4256	0.1934	0.9366
**ukb-d-22601_35393271**	Job coding: management information officer, conference/events co-ordinator/organiser, exhibition officer, work study engineer/officer/analyst, contract adviser/agent, election agent, business system analyst	acute RITs	European	Lifestyle, occupation and family background	22	IVW	1.4220	0.6368	0.0255	0.1739	5.2473	4.1454	1.1899	14.4419
**ukb-d-22601_23193405**	Job coding: other teaching professional including private tutor, tefl (teaching english as a foreign language) teacher; tutor at adult education centre, etc.	acute RITs	European	Lifestyle, occupation and family background	20	IVW	2.8991	0.8189	0.0004	1.2940	6.6754	18.1570	3.6473	90.3888
**ukb-d-22601_23213026**	Job coding: scientific researcher, scientific officer, medical research associate, experimental officer	acute RITs	European	Lifestyle, occupation and family background	23	IVW	-1.6119	0.8022	0.0445	-3.1843	5.8148	0.1995	0.0414	0.9612
**ukb-d-22601_23163403**	Job coding: special needs teaching professional (including head teacher)	acute RITs	European	Lifestyle, occupation and family background	19	IVW	-2.4505	1.0982	0.0257	-4.6030	5.4522	0.0862	0.0100	0.7423
**ukb-e-826_AFR**	Job involves shift work	acute RITs	African American or Afro-Caribbean	Lifestyle, occupation and family background	15	IVW	-0.0277	0.0125	0.0269	-0.0522	0.0252	0.9727	0.9491	0.9968
**ukb-d-22617_1151**	Job SOC coding: Financial institution managers	acute RITs	European	Lifestyle, occupation and family background	21	IVW	-2.5693	0.9299	0.0057	-4.3920	5.9452	0.0766	0.0124	0.4740

**ukb-d-22617_2321**	Job SOC coding: Scientific researchers	acute RITs	European	Lifestyle, occupation and family background	26	IVW	-1.7650	0.8652	0.0413	-3.4608	5.4610	0.1712	0.0314	0.9331
**ukb-d-22617_3231**	Job SOC coding: Youth and community workers	acute RITs	European	Lifestyle, occupation and family background	24	IVW (fixed effects)	1.7189	0.8373	0.0401	0.0778	3.3599	5.5781	1.0809	28.7875
**ukb-b-18099**	Qualifications: O levels/GCSEs or equivalent	acute RITs	European	Lifestyle, occupation and family background	134	IVW	0.4338	0.1597	0.0066	0.1208	1.9383	1.5431	1.1284	2.1102
**ukb-a-368**	Work/job satisfaction	acute RITs	European	Lifestyle, occupation and family background	24	IVW	0.2203	0.0902	0.0146	0.0436	0.3851	1.2465	1.0445	1.4875
**ukb-b-5779**	Alcohol intake frequency.	acute RITs	European	Lifestyle, occupation and family background	295	Weighted mode	-0.1909	0.0854	0.0262	-0.3583	-0.0235	0.8262	0.6988	0.9768
**ukb-a-32**	Alcohol intake versus 10 years previously	acute RITs	European	Lifestyle, occupation and family background	68	MR Egger	-1.0401	0.4681	0.0297	-1.9576	-0.1227	0.3534	0.1412	0.8845
**ukb-e-1628_CSA**	Alcohol intake versus 10 years previously	acute RITs	South Asian	Lifestyle, occupation and family background	21	IVW	-0.0612	0.0278	0.0274	-0.1156	0.0427	0.9406	0.8908	0.9932
**ieu-b-4825**	Cigarettes smoked per day	acute RITs	European	Lifestyle, occupation and family background	16	Weighted median	0.0121	0.0056	0.0314	0.0011	0.0232	1.0122	1.0011	1.0235
**ukb-b-1572**	Difficulty not smoking for 1 day	acute RITs	European	Lifestyle, occupation and family background	22	IVW	0.1332	0.0523	0.0109	0.0307	0.5096	1.1425	1.0312	1.2658
**ukb-a-342**	Number of cigarettes currently smoked daily (current cigarette smokers)	acute RITs	European	Lifestyle, occupation and family background	24	IVW	0.1197	0.0531	0.0241	0.0157	0.4898	1.1271	1.0158	1.2507
**ukb-a-238**	Pack years adult smoking as proportion of life span exposed to smoking PREVIEW ONLY	acute RITs	European	Lifestyle, occupation and family background	63	MR Egger	0.2548	0.1227	0.0421	0.0143	0.4953	1.2902	1.0144	1.6409
**ukb-a-237**	Pack years of smoking PREVIEW ONLY	acute RITs	European	Lifestyle, occupation and family background	62	IVW	0.0985	0.0476	0.0383	0.0053	0.1918	1.1036	1.0053	1.2114
**ebi-a-GCST008027**	Smoking behavior (cigarettes smoked per day)	acute RITs	Hispanic or Latin American	Lifestyle, occupation and family background	30	IVW	0.0874	0.0432	0.0429	0.0028	0.0472	1.0914	1.0028	1.1878
**ieu-a-1010**	Years of schooling	acute RITs	European	Lifestyle, occupation and family background	96	IVW	-0.1079	0.0523	0.0391	-0.2105	0.5593	0.8977	0.8102	0.9946

**ukb-e-Z88_AFR**	Z88 Personal history of allergy to drugs, medicaments and biologocal substances	acute RITs	African American or Afro-Caribbean	Lifestyle, occupation and family background	19	IVW	0.0111	0.0056	0.0463	0.0002	0.0557	1.0111	1.0002	1.0222
**ukb-e-1677_CSA**	Breastfed as a baby	acute RITs	South Asian	Lifestyle, occupation and family background	8	IVW	0.0111	0.0054	0.0412	0.0004	0.0250	1.0112	1.0004	1.0220
**ukb-e-1468_p1_CSA**	Cereal type	acute RITs	South Asian	Lifestyle, occupation and family background	23	IVW	0.0239	0.0072	0.0009	0.0098	0.0422	1.0242	1.0098	1.0388
**ukb-e-24014_AFR**	Close to major road	acute RITs	African American or Afro-Caribbean	Lifestyle, occupation and family background	16	IVW	-0.0175	0.0081	0.0312	-0.0334	0.0134	0.9827	0.9672	0.9984
**ukb-e-894_AFR**	Duration of moderate activity	acute RITs	African American or Afro-Caribbean	Lifestyle, occupation and family background	18	IVW (fixed effects)	-0.0400	0.0202	0.0474	-0.0796	-0.0005	0.9608	0.9235	0.9995
**ukb-e-874_p1_AFR**	Duration of walks	acute RITs	African American or Afro-Caribbean	Lifestyle, occupation and family background	24	IVW	-0.0286	0.0124	0.0207	-0.0529	0.0116	0.9718	0.9485	0.9956
**ukb-e-404_CSA**	Duration to first press of snap-button in each round	acute RITs	South Asian	Lifestyle, occupation and family background	20	IVW	0.0478	0.0236	0.0425	0.0016	0.1360	1.0490	1.0016	1.0986
**ukb-d-20411_0**	Ever been injured or injured someone else through drinking alcohol: No	acute RITs	European	Lifestyle, occupation and family background	18	IVW	-1.5032	0.5759	0.0090	-2.6319	0.8239	0.2224	0.0719	0.6876
**ieu-b-4860**	Physical activity	acute RITs	European	Lifestyle, occupation and family background	20	IVW (fixed effects)	0.3953	0.1916	0.0391	0.0198	0.7708	1.4848	1.0200	2.1615
**ebi-a-GCST90061410**	Physical activity (Total log acceleration 2am-4am)	acute RITs	European	Lifestyle, occupation and family background	27	IVW	0.1756	0.0802	0.0287	0.0183	0.1315	1.1919	1.0185	1.3949
**ukb-a-421**	Reason for glasses/contact lenses: For just reading/near work as you are getting older (called 'presbyopia')	acute RITs	European	Lifestyle, occupation and family background	43	IVW	1.1337	0.4626	0.0142	0.2271	4.1067	3.1072	1.2550	7.6932
**ukb-a-420**	Reason for glasses/contact lenses: For long-sightedness i.e. for distance and near but particularly for near tasks like reading (called 'hypermetropia')	acute RITs	European	Lifestyle, occupation and family background	39	IVW	1.4000	0.6316	0.0266	0.1621	9.6602	4.0552	1.1760	13.9843
**ukb-d-2664_5**	Reason for reducing amount of alcohol drunk: Other reason	acute RITs	European	Lifestyle, occupation and family background	28	IVW	-0.3661	0.1855	0.0484	-0.7297	-0.1109	0.6934	0.4821	0.9975
**ukb-a-343**	Time from waking to first cigarette	acute RITs	European	Lifestyle, occupation and family	28	Weighted median	-0.1320	0.0477	0.0057	-0.2255	-0.0384	0.8764	0.7981	0.9624

				background										
**ukb-b-2732**	Time from waking to first cigarette	acute RITs	European	Lifestyle, occupation and family background	24	Weighted median	-0.1407	0.0589	0.0170	-0.2563	-0.0252	0.8687	0.7739	0.9751
**ukb-a-7**	Time spent driving	acute RITs	European	Lifestyle, occupation and family background	47	MR Egger	-0.9471	0.4635	0.0469	-1.8556	-0.0385	0.3879	0.1564	0.9622
**ukb-b-3709**	Wants to stop smoking	acute RITs	European	Lifestyle, occupation and family background	19	IVW	-0.2099	0.0729	0.0040	-0.3528	0.7417	0.8107	0.7027	0.9353
**ebi-a-GCST90013919**	Cholesterol lowering medication use (UKB data field 6177_1) (Firth correction)	acute RITs	European	Medication use and treatment	122	IVW	0.0393	0.0158	0.0131	0.0082	0.3863	1.0401	1.0083	1.0728
**ebi-a-GCST90013969**	Cholesterol lowering medication use (UKB data field 6177_1) (SPA correction)	acute RITs	European	Medication use and treatment	121	IVW	0.0402	0.0158	0.0110	0.0092	0.1424	1.0410	1.0092	1.0737
**ukb-a-488**	Medication for cholesterol blood pressure or diabetes: Cholesterol lowering medication	acute RITs	European	Medication use and treatment	94	IVW (fixed effects)	0.1837	0.0931	0.0485	0.0012	0.3662	1.2016	1.0012	1.4422
**ukb-b-11740**	Medication for cholesterol, blood pressure or diabetes: Cholesterol lowering medication	acute RITs	European	Medication use and treatment	129	IVW	0.2404	0.0962	0.0124	0.0519	0.6031	1.2717	1.0532	1.5356
**ukb-e-6153_p2_AFR**	Medication for cholesterol, blood pressure, diabetes, or take exogenous hormones	acute RITs	African American or Afro-Caribbean	Medication use and treatment	21	IVW (fixed effects)	0.0127	0.0063	0.0442	0.0003	0.0250	1.0127	1.0003	1.0253
**ebi-a-GCST90018984**	Medication use (antihypertensives)	acute RITs	European	Medication use and treatment	53	IVW	0.0389	0.0190	0.0413	0.0015	0.1315	1.0396	1.0015	1.0792
**ebi-a-GCST90018767**	Medication use (calcium channel blockers)	acute RITs	East Asian	Medication use and treatment	68	IVW (fixed effects)	0.0505	0.0229	0.0277	0.0056	0.0954	1.0518	1.0056	1.1001
**ebi-a-GCST90018985**	Medication use (diuretics)	acute RITs	European	Medication use and treatment	207	IVW	0.0304	0.0150	0.0425	0.0010	0.1315	1.0309	1.0010	1.0617
**ebi-a-GCST90018993**	Medication use (drugs affecting bone structure and mineralization)	acute RITs	European	Medication use and treatment	59	IVW	0.0346	0.0173	0.0454	0.0007	0.1315	1.0352	1.0007	1.0709
**ukb-b-12753**	Recent medication for hayfever or allergic rhinitis	acute RITs	European	Medication use and treatment	17	IVW	-0.2684	0.1237	0.0300	-0.5107	0.5313	0.7646	0.6001	0.9743
**ukb-b-15918**	Treatment speciality of consultant (recoded): Dermatology	acute RITs	European	Medication use and treatment	30	Weighted median	-3.6432	1.7622	0.0387	-7.0971	-0.1892	0.0262	0.0008	0.8276
**ukb-b-3255**	Treatment speciality of consultant (recoded): Upper gastrointestinal surgery	acute RITs	European	Medication use and treatment	7	IVW	-16.5261	6.1088	0.0068	-28.4993	-4.5529	0.0000	0.0000	0.0105
**ukb-e-20003_p118_AFR**	Treatment/medication code	acute RITs	African American or Afro-Caribbean	Medication use and treatment	20	IVW (fixed effects)	-0.0154	0.0074	0.0372	-0.0298	-0.0009	0.9847	0.9706	0.9991
**ukb-b-9207**	Treatment/medication code: amlodipine	acute RITs	European	Medication use and treatment	89	IVW	0.8756	0.4229	0.0384	0.0467	1.7044	2.4002	1.0478	5.4983
**ukb-b-11862**	Treatment/medication code: beconase 50micrograms nasal spray	acute RITs	European	Medication use and treatment	10	IVW	8.3394	3.5026	0.0173	1.4742	56.4303	4185.4250	4.3675	4010986.2957

**ukb-b-7385**	Treatment/medication code: codeine	acute RITs	European	Medication use and treatment	6	IVW (fixed effects)	10.7788	5.4105	0.0464	0.1741	21.3834	47991.3786	1.1902	1935116309.0000
**ukb-b-8668**	Treatment/medication code: doxazosin	acute RITs	European	Medication use and treatment	36	IVW	2.9459	1.4017	0.0356	0.1986	25.9528	19.0276	1.2197	296.8410
**ukb-a-162**	Treatment/medication code: perindopril	acute RITs	European	Medication use and treatment	23	Weighted median	4.6098	2.0604	0.0253	0.5714	8.6482	100.4627	1.7707	5699.8738
**ukb-a-141**	Treatment/medication code: prednisolone	acute RITs	European	Medication use and treatment	15	MR Egger	19.2935	8.3631	0.0382	2.9018	35.6852	239362625.6000	18.2066	3150000000000000.0000
**ukb-b-11895**	Treatment/medication code: ramipril	acute RITs	European	Medication use and treatment	65	IVW	1.0952	0.4980	0.0279	0.1190	4.8634	2.9897	1.1264	7.9351
**ukb-a-130**	Treatment/medication code: senna	acute RITs	European	Medication use and treatment	27	IVW	-8.1696	3.6370	0.0247	-15.2981	14.0181	0.0003	0.0000	0.3531
**ukb-b-16956**	Treatment/medication code: thyroxine sodium	acute RITs	European	Medication use and treatment	11	IVW	13.8864	4.6817	0.0030	4.7102	62.8267	1073421.5753	111.0693	10374006079.1460
**ukb-a-120**	Treatment/medication code: tranexamic acid	acute RITs	European	Medication use and treatment	23	Weighted median	-14.1893	7.1441	0.0470	-28.1917	-0.1869	0.0000	0.0000	0.8295
**ukb-b-17616**	Treatment/medication code: xalatan 0.005% eye drops	acute RITs	European	Medication use and treatment	11	IVW	-10.4543	4.5727	0.0222	-19.4169	63.7345	0.0000	0.0000	0.2250
**ukb-e-6155_p5_AFR**	Vitamin and mineral supplements	acute RITs	African American or Afro-Caribbean	Medication use and treatment	12	Weighted median	-0.0192	0.0096	0.0466	-0.0380	-0.0003	0.9810	0.9627	0.9997
**ukb-e-6155_p8_AFR**	Vitamin and mineral supplements	acute RITs	African American or Afro-Caribbean	Medication use and treatment	16	Weighted median	-0.0303	0.0138	0.0281	-0.0574	-0.0033	0.9701	0.9442	0.9967
**ukb-a-464**	Vitamin and mineral supplements: Multivitamins +/- minerals	acute RITs	European	Medication use and treatment	47	IVW	0.6782	0.2904	0.0195	0.1089	2.9932	1.9703	1.1151	3.4813
**ukb-b-19550**	Vitamin and/or mineral supplement use: Calcium	acute RITs	European	Medication use and treatment	6	IVW	1.3549	0.6146	0.0275	0.1503	2.5595	3.8764	1.1622	12.9297
**ukb-b-18593**	Vitamin D	acute RITs	European	Medication use and treatment	22	Weighted median	0.2210	0.1065	0.0380	0.0123	0.4297	1.2473	1.0123	1.5368
**ukb-e-Z51_AFR**	Z51 Other medical care	acute RITs	African American or Afro-Caribbean	Medication use and treatment	15	IVW	0.0126	0.0063	0.0446	0.0003	0.0416	1.0126	1.0003	1.0251
**ukb-b-17102**	External causes: Y83.6 Removal of other organ (partial) (total)	acute RITs	European	Medication use and treatment	9	IVW	-9.0358	3.9885	0.0235	-16.8533	33.0263	0.0001	0.0000	0.2957
**ukb-b-11247**	Main speciality of consultant (recoded): Cardiothoracic surgery	acute RITs	European	Medication use and treatment	22	IVW (fixed effects)	3.3575	1.7124	0.0499	0.0012	6.7137	28.7161	1.0012	823.6148
**ukb-b-17277**	Operation code: anal surgery	acute RITs	European	Medication use and treatment	10	IVW	9.3425	4.2876	0.0293	0.9388	70.1816	11413.2680	2.5570	50943504.0211
**ukb-b-6863**	Operation code: caesarean section / caesarian section	acute RITs	European	Medication use and treatment	44	IVW	-1.2227	0.6194	0.0484	-2.4367	0.0062	0.2944	0.0874	0.9913
**ukb-b-6235**	Operation code: cholecystectomy/gall bladder removal	acute RITs	European	Medication use and treatment	99	Weighted median	-1.0905	0.4225	0.0098	-1.9185	-0.2625	0.3360	0.1468	0.7691
**ukb-b-6235**	Operation code: cholecystectomy/gall bladder removal	acute RITs	European	Medication use and treatment	99	Weighted mode	-0.8731	0.4242	0.0422	-1.7045	-0.0417	0.4177	0.1819	0.9592
**ukb-b-9263**	Operation code:	acute RITs	European	Medication use	75	IVW	1.2443	0.4918	0.0114	0.2804	3.3527	3.4706	1.3237	9.0995

	inguinal/femoral hernia repair			and treatment										
**ukb-b-847**	Operation code: reduction or fixation of bone fracture	acute RITs	European	Medication use and treatment	31	IVW	-1.9114	0.8764	0.0292	-3.6291	3.0605	0.1479	0.0265	0.8240
**ukb-b-13003**	Operation code: sinus surgery	acute RITs	European	Medication use and treatment	6	IVW	-19.2229	7.4735	0.0101	-33.8710	290.6570	0.0000	0.0000	0.0103
**ukb-b-15592**	Operation code: varicose vein surgery	acute RITs	European	Medication use and treatment	206	MR Egger	-1.3607	0.6402	0.0347	-2.6154	-0.1060	0.2565	0.0731	0.8994
**ukb-b-7609**	Operative procedures - main OPCS: B28.2 Partial excision of breast NEC	acute RITs	European	Medication use and treatment	24	IVW	4.8776	1.7279	0.0048	1.4910	11.7569	131.3207	4.4416	3882.6157
**ukb-b-4944**	Operative procedures - main OPCS: W85.2 Endoscopic irrigation of knee joint	acute RITs	European	Medication use and treatment	4	IVW	-17.4403	8.5926	0.0424	-34.2818	-0.5988	0.0000	0.0000	0.5495
**ukb-e-41200_p138_AFR**	Operative procedures - main OPCS4	acute RITs	African American or Afro-Caribbean	Medication use and treatment	25	IVW	0.0100	0.0039	0.0104	0.0024	0.0467	1.0101	1.0024	1.0178
**ukb-e-41200_p26_CSA**	Operative procedures - main OPCS4	acute RITs	South Asian	Medication use and treatment	19	IVW	-0.0069	0.0035	0.0473	-0.0138	0.0187	0.9931	0.9863	0.9999
**ukb-e-41200_p356_AFR**	Operative procedures - main OPCS4	acute RITs	African American or Afro-Caribbean	Medication use and treatment	12	IVW	0.0116	0.0047	0.0128	0.0025	0.0321	1.0116	1.0025	1.0209
**ukb-e-41200_p834_AFR**	Operative procedures - main OPCS4	acute RITs	African American or Afro-Caribbean	Medication use and treatment	15	IVW	-0.0095	0.0047	0.0461	-0.0188	0.0175	0.9906	0.9814	0.9998
**ukb-e-41200_p991_CSA**	Operative procedures - main OPCS4	acute RITs	South Asian	Medication use and treatment	9	Weighted median	-0.0172	0.0083	0.0375	-0.0334	-0.0010	0.9829	0.9671	0.9990
**ukb-b-4820**	Operative procedures - secondary OPCS: E13.6 Puncture of maxillary antrum	acute RITs	European	Medication use and treatment	5	IVW	23.9016	8.5527	0.0052	7.1383	349.2162	24007019831.6372	1259.3000	457664588623786000.0000
**ukb-b-4373**	Operative procedures - secondary OPCS: T85.2 Block dissection of axillary lymph nodes	acute RITs	European	Medication use and treatment	17	IVW	6.3867	2.3066	0.0056	1.8657	10.9076	593.8685	6.4607	54588.7024
**ukb-b-7815**	Operative procedures - secondary OPCS: U05.1 Computed tomography of head	acute RITs	European	Medication use and treatment	6	IVW	12.7099	6.4642	0.0493	0.0401	25.3796	331001.5553	1.0409	105254400904.5330
**ukb-b-13551**	Operative procedures - secondary OPCS: X72.1 Delivery of complex chemotherapy for neoplasm including prolonged infusional treatment at first attendance	acute RITs	European	Medication use and treatment	6	IVW (fixed effects)	11.8655	6.0520	0.0499	0.0035	23.7274	142265.8249	1.0035	20169020745.0000
**ukb-b-13683**	Operative procedures - secondary OPCS: Y58.8 Other specified harvest of skin for graft	acute RITs	European	Medication use and treatment	3	IVW	24.6172	11.6528	0.0346	1.7777	1470.0069	49105378845.9730	5.9165	407561435025151000000.0000
**ukb-b-9816**	Operative procedures - secondary OPCS: Y80.4 Intravenous anaesthetic NEC	acute RITs	European	Medication use and treatment	9	IVW	11.2117	4.9396	0.0232	1.5301	175.3342	73992.1128	4.6186	1185390592.3667
**ukb-b-13121**	Operative procedures - secondary OPCS: Z27.1 Oesophagus	acute RITs	European	Medication use and treatment	10	IVW	-6.1811	2.7861	0.0265	-11.6419	56.9258	0.0021	0.0000	0.4866
**ukb-b-5222**	Operative procedures - secondary OPCS: Z94.1	acute RITs	European	Medication use and treatment	18	MR Egger	7.9016	3.6592	0.0463	0.7296	15.0736	2701.6368	2.0742	3518827.8190

	Bilateral operation													
**ukb-e-Y83_CSA**	Y83 Surgical operation and other surgical procedures as the cause of abnormal reaction of the patient, or of later complication, without mention of misadventure at the time of the procedure	acute RITs	South Asian	Medication use and treatment	16	IVW (fixed effects)	-0.2525	0.1155	0.0288	-0.4788	-0.0261	0.7769	0.6195	0.9742
**prot-a-2235**	[Pyruvate dehydrogenase (acetyl-transferring)] kinase isozyme 1, mitochondrial	acute RITs	European	Metabolism	12	IVW	0.0422	0.0167	0.0116	0.0094	0.1350	1.0431	1.0095	1.0779
**prot-a-3251**	14-3-3 protein zeta/delta	acute RITs	European	Metabolism	20	IVW	0.0411	0.0196	0.0361	0.0027	0.1149	1.0420	1.0027	1.0829
**prot-a-1370**	15-hydroxyprostaglandin dehydrogenase [NAD(+)]	acute RITs	European	Metabolism	23	IVW	0.0386	0.0161	0.0164	0.0071	0.0738	1.0394	1.0071	1.0727
**prot-a-11**	Acetyl-CoA carboxylase 2	acute RITs	European	Metabolism	19	IVW	0.0389	0.0182	0.0322	0.0033	0.0745	1.0397	1.0033	1.0773
**prot-a-105**	Acidic leucine-rich nuclear phosphoprotein 32 family member B	acute RITs	European	Metabolism	24	IVW	0.0333	0.0161	0.0385	0.0018	0.0649	1.0339	1.0018	1.0670
**prot-a-43**	Adhesion G-protein coupled receptor F1	acute RITs	European	Metabolism	19	IVW	0.0304	0.0154	0.0487	0.0002	0.0606	1.0309	1.0002	1.0625
**prot-a-47**	Alcohol dehydrogenase 4	acute RITs	European	Metabolism	22	IVW	-0.0460	0.0172	0.0076	-0.0797	0.0761	0.9551	0.9234	0.9879
**prot-a-1155**	Alpha-(1,3)-fucosyltransferase 9	acute RITs	European	Metabolism	16	IVW	-0.0407	0.0119	0.0006	-0.0640	-0.0297	0.9601	0.9380	0.9827
**prot-a-2290**	Calcium-dependent phospholipase A2	acute RITs	European	Metabolism	23	IVW (fixed effects)	0.0337	0.0165	0.0411	0.0014	0.0661	1.0343	1.0014	1.0683
**prot-a-557**	Carbohydrate sulfotransferase 5	acute RITs	European	Metabolism	16	IVW	-0.0427	0.0214	0.0459	-0.0847	-0.0008	0.9582	0.9188	0.9992
**prot-a-332**	Carbonic anhydrase 5A, mitochondrial	acute RITs	European	Metabolism	14	IVW	-0.0521	0.0216	0.0160	-0.0945	0.1574	0.9492	0.9099	0.9903
**prot-a-327**	Carbonic anhydrase-related protein 10	acute RITs	European	Metabolism	22	MR Egger	0.1267	0.0572	0.0384	0.0147	0.2388	1.1351	1.0148	1.2697
**prot-a-649**	Carboxypeptidase Z	acute RITs	European	Metabolism	21	IVW	0.0469	0.0225	0.0371	0.0028	0.1550	1.0480	1.0028	1.0953
**prot-a-1086**	D-dimer	acute RITs	European	Metabolism	16	IVW	0.0637	0.0226	0.0048	0.0194	0.2087	1.0658	1.0196	1.1141
**met-c-846**	3-hydroxybutyrate	acute RITs	European	Metabolism	17	IVW	-0.0955	0.0463	0.0392	-0.1862	0.1102	0.9090	0.8301	0.9953
**met-d-bOHbutyrate**	3-Hydroxybutyrate	acute RITs	European	Metabolism	44	IVW	-0.1187	0.0593	0.0452	-0.2348	0.1123	0.8881	0.7907	0.9974
**met-a-311**	3-hydroxybutyrate (BHBA)	acute RITs	European	Metabolism	9	IVW	0.1307	0.0588	0.0262	0.0155	0.3469	1.1397	1.0156	1.2789
**met-a-365**	3-methoxytyrosine	acute RITs	European	Metabolism	16	IVW	0.4006	0.1834	0.0290	0.0411	0.6012	1.4927	1.0419	2.1384
**ieu-a-1**	Adiponectin	acute RITs	Mixed	Metabolism	32	IVW	0.0879	0.0404	0.0293	0.0089	0.1670	1.0919	1.0089	1.1818
**met-a-537**	Adrenate (22:4n6)	acute RITs	European	Metabolism	11	IVW (fixed effects)	0.2715	0.1317	0.0393	0.0134	0.5296	1.3119	1.0134	1.6982
**bbj-a-9**	Albumin	acute RITs	East Asian	Metabolism	48	Weighted median	0.1586	0.0690	0.0215	0.0234	0.2938	1.1718	1.0236	1.3415
**met-a-616**	Alpha-hydroxyisovalerate	acute RITs	European	Metabolism	14	IVW	0.2090	0.1026	0.0417	0.0078	0.4951	1.2324	1.0079	1.5070
**met-d-ApoA1**	Apolipoprotein A1	acute RITs	European	Metabolism	141	IVW	0.0548	0.0243	0.0242	0.0071	0.1416	1.0564	1.0072	1.1080
**ieu-b-107**	apolipoprotein A-I	acute RITs	European	Metabolism	469	IVW	0.0442	0.0217	0.0418	0.0016	0.1650	1.0452	1.0016	1.0906
**met-c-842**	Apolipoprotein A-I	acute RITs	European	Metabolism	30	IVW	0.0534	0.0246	0.0302	0.0051	0.1250	1.0549	1.0051	1.1071
**ieu-b-108**	apolipoprotein B	acute RITs	European	Metabolism	302	MR Egger	0.0808	0.0370	0.0299	0.0082	0.1534	1.0841	1.0082	1.1657
**ieu-b-108**	apolipoprotein B	acute RITs	European	Metabolism	302	Weighted mode	0.0812	0.0382	0.0342	0.0064	0.1559	1.0846	1.0064	1.1688
**met-d-ApoB**	Apolipoprotein B	acute RITs	European	Metabolism	108	IVW	0.0581	0.0248	0.0190	0.0095	0.0870	1.0598	1.0096	1.1126
**ebi-a-GCST90092809**	Apolipoprotein B levels	acute RITs	European	Metabolism	109	IVW	0.0612	0.0249	0.0141	0.0124	0.1315	1.0631	1.0124	1.1163
**prot-a-134**	Apolipoprotein L1	acute RITs	European	Metabolism	21	MR Egger	-0.0543	0.0233	0.0307	-0.1000	-0.0087	0.9471	0.9049	0.9913

**met-a-638**	Asparagine	acute RITs	European	Metabolism	39	MR Egger	-0.5814	0.2803	0.0451	-1.1308	-0.0319	0.5591	0.3228	0.9686
**ieu-b-4869**	Bioavailable Testosterone	acute RITs	European	Metabolism	231	Weighted median	-0.1012	0.0430	0.0187	-0.1856	-0.0169	0.9037	0.8306	0.9833
**ebi-a-GCST90012102**	Bioavailable testosterone levels	acute RITs	European	Metabolism	266	Weighted median	-0.1419	0.0542	0.0089	-0.2482	-0.0356	0.8677	0.7802	0.9651
**ebi-a-GCST90012104**	Bioavailable testosterone levels	acute RITs	European	Metabolism	224	Weighted median	-0.1813	0.0757	0.0165	-0.3296	-0.0330	0.8342	0.7192	0.9675
**ebi-a-GCST90012104**	Bioavailable testosterone levels	acute RITs	European	Metabolism	224	Weighted mode	-0.2387	0.1198	0.0476	-0.4735	-0.0038	0.7877	0.6228	0.9962
**bbj-a-10**	Blood sugar	acute RITs	East Asian	Metabolism	44	Weighted median	-0.1606	0.0719	0.0256	-0.3015	-0.0196	0.8517	0.7397	0.9806
**ebi-a-GCST90018948**	Blood urea nitrogen levels	acute RITs	European	Metabolism	357	MR Egger	0.1385	0.0701	0.0491	0.0010	0.2760	1.1486	1.0010	1.3179
**met-a-481**	Caprylate (8:0)	acute RITs	European	Metabolism	42	IVW	0.3180	0.1438	0.0270	0.0362	1.1848	1.3744	1.0368	1.8219
**met-c-887**	Cholesterol esters in large VLDL	acute RITs	European	Metabolism	31	Weighted median	-0.0767	0.0369	0.0376	-0.1490	-0.0044	0.9262	0.8616	0.9956
**met-c-899**	Cholesterol esters in medium HDL	acute RITs	European	Metabolism	21	Weighted median	0.0891	0.0449	0.0475	0.0010	0.1771	1.0932	1.0010	1.1938
**met-c-899**	Cholesterol esters in medium HDL	acute RITs	European	Metabolism	21	Weighted mode	0.0985	0.0492	0.0589	0.0021	0.1950	1.1036	1.0021	1.2153
**met-d-M_HDL_C**	Cholesterol in medium HDL	acute RITs	European	Metabolism	152	IVW	0.0619	0.0247	0.0121	0.0136	0.1537	1.0639	1.0137	1.1166
**met-d-M_VLDL_C**	Cholesterol in medium VLDL	acute RITs	European	Metabolism	114	IVW (fixed effects)	0.0496	0.0248	0.0455	0.0010	0.0981	1.0508	1.0010	1.1031
**met-d-S_LDL_C**	Cholesterol in small LDL	acute RITs	European	Metabolism	101	IVW	0.0543	0.0263	0.0394	0.0026	0.1315	1.0558	1.0027	1.1118
**ebi-a-GCST90014000**	Cholesterol levels (UKB data field 30690)	acute RITs	European	Metabolism	279	IVW	0.0594	0.0271	0.0284	0.0063	0.1315	1.0613	1.0063	1.1192
**ebi-a-GCST90092956**	Cholesterol levels in small LDL	acute RITs	European	Metabolism	97	IVW	0.0533	0.0238	0.0249	0.0067	0.1315	1.0548	1.0068	1.1051
**met-d-M_HDL_C_pct**	Cholesterol to total lipids ratio in medium HDL	acute RITs	European	Metabolism	168	IVW	0.0450	0.0213	0.0347	0.0032	0.1481	1.0460	1.0032	1.0906
**ebi-a-GCST90092918**	Cholesteryl ester levels in medium VLDL	acute RITs	European	Metabolism	96	IVW	0.0585	0.0264	0.0269	0.0067	0.1315	1.0602	1.0067	1.1166
**ebi-a-GCST90060634**	Cholesteryl ester(18:3)_[M+NH4]1+ levels	acute RITs	South Asian	Metabolism	20	IVW	0.1248	0.0470	0.0079	0.0327	0.1315	1.1329	1.0332	1.2422
**met-d-HDL_CE**	Cholesteryl esters in HDL	acute RITs	European	Metabolism	182	IVW	0.0429	0.0217	0.0477	0.0004	0.0854	1.0438	1.0004	1.0891
**met-d-L_LDL_CE**	Cholesteryl esters in large LDL	acute RITs	European	Metabolism	102	IVW	0.0541	0.0241	0.0247	0.0069	0.1185	1.0556	1.0069	1.1067
**met-d-LDL_CE**	Cholesteryl esters in LDL	acute RITs	European	Metabolism	98	IVW	0.0572	0.0266	0.0314	0.0051	0.1093	1.0589	1.0051	1.1155
**met-d-M_HDL_CE**	Cholesteryl esters in medium HDL	acute RITs	European	Metabolism	154	IVW	0.0605	0.0247	0.0144	0.0120	0.1090	1.0624	1.0121	1.1151
**met-d-M_VLDL_CE**	Cholesteryl esters in medium VLDL	acute RITs	European	Metabolism	101	IVW	0.0552	0.0264	0.0368	0.0034	0.1070	1.0567	1.0034	1.1129
**met-d-S_HDL_CE**	Cholesteryl esters in small HDL	acute RITs	European	Metabolism	104	IVW	0.0510	0.0257	0.0469	0.0007	0.1014	1.0524	1.0007	1.1067
**met-d-IDL_CE_pct**	Cholesteryl esters to total lipids ratio in IDL	acute RITs	European	Metabolism	130	MR Egger	0.0606	0.0287	0.0367	0.0043	0.1169	1.0625	1.0043	1.1241
**met-d-L_HDL_CE_pct**	Cholesteryl esters to total lipids ratio in large HDL	acute RITs	European	Metabolism	176	Weighted median	0.0753	0.0371	0.0423	0.0026	0.1480	1.0782	1.0026	1.1595
**met-d-L_HDL_CE_pct**	Cholesteryl esters to total lipids ratio in large HDL	acute RITs	European	Metabolism	176	Weighted mode	0.0712	0.0331	0.0326	0.0064	0.1360	1.0738	1.0064	1.1457
**met-d-M_HDL_CE_pct**	Cholesteryl esters to total lipids ratio in medium HDL	acute RITs	European	Metabolism	163	IVW	0.0475	0.0213	0.0256	0.0058	0.0892	1.0486	1.0058	1.0933
**ebi-a-GCST90007307**	circulating leptin levels	acute RITs	Mixed	Metabolism	15	IVW	-0.1557	0.0589	0.0082	-0.2712	0.0657	0.8558	0.7625	0.9606
**ebi-a-GCST90007309**	circulating leptin levels	acute RITs	Mixed	Metabolism	7	IVW	-0.1793	0.0701	0.0105	-0.3166	0.0657	0.8359	0.7286	0.9589
**ebi-a-GCST90007310**	circulating leptin levels	acute RITs	European	Metabolism	9	IVW	-0.1662	0.0715	0.0201	-0.3063	0.0657	0.8469	0.7362	0.9743

**ebi-a-GCST90007312**	circulating leptin levels	acute RITs	European	Metabolism	7	IVW	-0.1617	0.0664	0.0149	-0.2919	0.0657	0.8507	0.7468	0.9689
**ebi-a-GCST90007313**	circulating leptin levels	acute RITs	Mixed	Metabolism	6	IVW	-0.1408	0.0559	0.0118	-0.2504	0.0657	0.8687	0.7785	0.9693
**ebi-a-GCST90007316**	circulating leptin levels	acute RITs	European	Metabolism	6	IVW	-0.1387	0.0570	0.0149	-0.2504	0.0657	0.8705	0.7785	0.9733
**ebi-a-GCST90007319**	circulating leptin levels adjusted for BMI	acute RITs	Mixed	Metabolism	10	IVW	-0.1734	0.0651	0.0077	-0.3010	0.0657	0.8408	0.7401	0.9552
**ebi-a-GCST90007320**	circulating leptin levels adjusted for BMI	acute RITs	Mixed	Metabolism	6	IVW	-0.1564	0.0736	0.0336	-0.3006	0.0657	0.8552	0.7403	0.9880
**ebi-a-GCST90007321**	circulating leptin levels adjusted for BMI	acute RITs	Mixed	Metabolism	9	Weighted median	-0.1596	0.0735	0.0299	-0.3036	-0.0155	0.8525	0.7381	0.9846
**ebi-a-GCST90007322**	circulating leptin levels adjusted for BMI	acute RITs	European	Metabolism	8	IVW	-0.1798	0.0687	0.0088	-0.3145	0.0657	0.8354	0.7302	0.9558
**ebi-a-GCST90007324**	circulating leptin levels adjusted for BMI	acute RITs	European	Metabolism	8	IVW	-0.1180	0.0546	0.0306	-0.2250	0.0657	0.8887	0.7985	0.9890
**ebi-a-GCST90007327**	circulating leptin levels adjusted for BMI	acute RITs	Mixed	Metabolism	6	IVW	-0.0976	0.0439	0.0262	-0.1836	0.0657	0.9070	0.8322	0.9885
**met-d-Clinical_LDL_C**	Clinical LDL cholesterol	acute RITs	European	Metabolism	100	IVW (fixed effects)	0.0471	0.0236	0.0463	0.0008	0.0934	1.0482	1.0008	1.0979
**met-d-HDL_P**	Concentration of HDL particles	acute RITs	European	Metabolism	142	IVW	0.0660	0.0252	0.0087	0.0167	0.1488	1.0682	1.0169	1.1222
**ebi-a-GCST90092887**	Concentration of LDL particles	acute RITs	European	Metabolism	104	IVW	0.0665	0.0251	0.0080	0.0174	0.1315	1.0688	1.0175	1.1226
**met-d-LDL_P**	Concentration of LDL particles	acute RITs	European	Metabolism	110	IVW	0.0607	0.0248	0.0144	0.0121	0.1094	1.0626	1.0121	1.1156
**met-d-M_HDL_P**	Concentration of medium HDL particles	acute RITs	European	Metabolism	142	IVW	0.0546	0.0245	0.0259	0.0066	0.1373	1.0562	1.0066	1.1082
**ebi-a-GCST90092963**	Concentration of small LDL particles	acute RITs	European	Metabolism	113	IVW	0.0550	0.0243	0.0234	0.0075	0.1315	1.0566	1.0075	1.1080
**met-d-S_LDL_P**	Concentration of small LDL particles	acute RITs	European	Metabolism	114	IVW	0.0535	0.0242	0.0272	0.0060	0.0814	1.0549	1.0060	1.1062
**met-d-XL_VLDL_P**	Concentration of very large VLDL particles	acute RITs	European	Metabolism	146	Weighted median	-0.0766	0.0377	0.0419	-0.1504	-0.0028	0.9262	0.8603	0.9972
**met-a-744**	Cyclo(leu-pro)	acute RITs	European	Metabolism	14	IVW (fixed effects)	0.1681	0.0797	0.0348	0.0120	0.3242	1.1830	1.0120	1.3830
**ebi-a-GCST90060606**	Diacylglycerol(34:2)_[M+H-H2O]1+ levels	acute RITs	South Asian	Metabolism	17	MR Egger	-0.4009	0.1541	0.0200	-0.7029	-0.0988	0.6697	0.4951	0.9059
**ebi-a-GCST90060606**	Diacylglycerol(34:2)_[M+H-H2O]1+ levels	acute RITs	South Asian	Metabolism	17	Weighted median	-0.2051	0.0895	0.0220	-0.3805	-0.0296	0.8146	0.6835	0.9708
**ebi-a-GCST90060625**	Diacylglycerol(36:2)_[M+NH4]1+ levels	acute RITs	South Asian	Metabolism	18	IVW	-0.1341	0.0467	0.0041	-0.2256	0.1315	0.8745	0.7980	0.9583
**ebi-a-GCST90025954**	Direct low density lipoprotein cholesterol levels	acute RITs	European	Metabolism	271	IVW	0.0522	0.0258	0.0426	0.0017	0.1315	1.0536	1.0017	1.1082
**met-a-586**	Eicosenoate (20:1n9 or 11)	acute RITs	European	Metabolism	12	IVW (fixed effects)	-0.2753	0.1285	0.0322	-0.5273	-0.0234	0.7593	0.5902	0.9769
**ebi-a-GCST90019407**	Electron transfer flavoprotein subunit alpha, mitochondrial measurement	acute RITs	European	Metabolism	24	IVW	0.0418	0.0177	0.0179	0.0072	0.1315	1.0427	1.0072	1.0795
**ebi-a-GCST90012105**	Estradiol levels	acute RITs	European	Metabolism	42	IVW (fixed effects)	0.5830	0.2667	0.0288	0.0602	1.1058	1.7914	1.0621	3.0216
**ebi-a-GCST000571**	Fasting blood insulin	acute RITs	European	Metabolism	12	IVW	0.2592	0.1247	0.0377	0.0148	1.6523	1.2959	1.0149	1.6548
**ebi-a-GCST90060570**	Fatty acid(15:0)_[M-H]1- levels	acute RITs	South Asian	Metabolism	12	Weighted median	-0.2378	0.1038	0.0220	-0.4413	-0.0344	0.7883	0.6432	0.9662
**ebi-a-GCST90060571**	Fatty acid(16:1)_[M-H]1- levels	acute RITs	South Asian	Metabolism	12	IVW	-0.1035	0.0347	0.0028	-0.1715	0.1315	0.9017	0.8424	0.9651
**met-d-HDL_FC**	Free cholesterol in HDL	acute RITs	European	Metabolism	169	IVW	0.0520	0.0216	0.0163	0.0096	0.1261	1.0533	1.0096	1.0989

**met-d-IDL_FC**	Free cholesterol in IDL	acute RITs	European	Metabolism	112	IVW	0.0488	0.0231	0.0344	0.0036	0.1245	1.0500	1.0036	1.0985
**met-c-900**	Free cholesterol in medium HDL	acute RITs	European	Metabolism	24	MR Egger	0.1781	0.0740	0.0250	0.0330	0.3232	1.1950	1.0335	1.3816
**met-d-M_HDL_FC**	Free cholesterol in medium HDL	acute RITs	European	Metabolism	152	IVW	0.0597	0.0257	0.0203	0.0093	0.1512	1.0615	1.0093	1.1164
**met-d-M_LDL_FC**	Free cholesterol in medium LDL	acute RITs	European	Metabolism	97	IVW	0.0491	0.0245	0.0451	0.0011	0.1027	1.0503	1.0011	1.1020
**met-d-M_HDL_FC_pct**	Free cholesterol to total lipids ratio in medium HDL	acute RITs	European	Metabolism	175	MR Egger	0.0632	0.0313	0.0451	0.0018	0.1245	1.0652	1.0018	1.1326
**met-d-S_HDL_FC_pct**	Free cholesterol to total lipids ratio in small HDL	acute RITs	European	Metabolism	125	IVW	0.0438	0.0213	0.0398	0.0020	0.1089	1.0448	1.0021	1.0893
**ebi-a-GCST90025966**	Gamma glutamyl transferase levels	acute RITs	European	Metabolism	486	Weighted median	0.0707	0.0353	0.0449	0.0016	0.1398	1.0733	1.0016	1.1501
**met-a-564**	Gamma-glutamylthreonine*	acute RITs	European	Metabolism	9	IVW	-0.4710	0.1699	0.0056	-0.8039	0.6493	0.6244	0.4476	0.8711
**ebi-a-GCST90018735**	Glucose levels	acute RITs	East Asian	Metabolism	47	IVW	-0.1215	0.0554	0.0284	-0.2302	0.1315	0.8856	0.7944	0.9872
**ebi-a-GCST005058**	HDL cholesterol	acute RITs	European	Metabolism	21	Weighted median	0.0694	0.0316	0.0282	0.0074	0.1313	1.0718	1.0074	1.1403
**ebi-a-GCST90018736**	HDL cholesterol	acute RITs	East Asian	Metabolism	104	Weighted median	0.0811	0.0340	0.0172	0.0144	0.1479	1.0845	1.0145	1.1594
**ebi-a-GCST90018956**	HDL cholesterol	acute RITs	European	Metabolism	466	MR Egger	0.0680	0.0323	0.0360	0.0046	0.1314	1.0704	1.0046	1.1404
**ieu-b-109**	HDL cholesterol	acute RITs	European	Metabolism	526	MR Egger	0.0712	0.0316	0.0247	0.0092	0.1331	1.0738	1.0093	1.1424
**ieu-b-4843**	HDL cholesterol	acute RITs	European	Metabolism	67	IVW	0.0396	0.0195	0.0422	0.0014	0.1220	1.0404	1.0014	1.0809
**ieu-b-4844**	HDL cholesterol	acute RITs	European	Metabolism	153	IVW	0.0516	0.0198	0.0090	0.0129	0.1405	1.0529	1.0129	1.0945
**met-d-HDL_C**	HDL cholesterol	acute RITs	European	Metabolism	178	IVW	0.0511	0.0218	0.0192	0.0083	0.1558	1.0524	1.0084	1.0984
**ukb-e-30760_AFR**	HDL cholesterol	acute RITs	African American or Afro-Caribbean	Metabolism	27	MR Egger	0.1038	0.0459	0.0328	0.0138	0.1937	1.1093	1.0139	1.2138
**ebi-a-GCST90025956**	HDL cholesterol levels	acute RITs	European	Metabolism	452	MR Egger	0.0544	0.0266	0.0413	0.0023	0.1064	1.0559	1.0023	1.1123
**ebi-a-GCST90002309**	Hemoglobin concentration	acute RITs	Mixed	Metabolism	40	IVW	0.0413	0.0190	0.0302	0.0040	0.2007	1.0422	1.0040	1.0818
**ebi-a-GCST008035**	High density lipoprotein cholesterol levels	acute RITs	Hispanic or Latin American	Metabolism	73	Weighted median	0.0044	0.0021	0.0408	0.0002	0.0086	1.0044	1.0002	1.0086
**ebi-a-GCST90014007**	High density lipoprotein cholesterol levels (UKB data field 30760)	acute RITs	European	Metabolism	458	IVW	0.0551	0.0221	0.0126	0.0118	0.1315	1.0567	1.0119	1.1034
**bbj-a-24**	High-density-lipoprotein cholesterol	acute RITs	East Asian	Metabolism	85	IVW	0.0395	0.0193	0.0405	0.0017	0.1117	1.0403	1.0017	1.0803
**ebi-a-GCST90014008**	IGF 1 (UKB data field 30770)	acute RITs	European	Metabolism	532	IVW	-0.0629	0.0215	0.0034	-0.1050	0.1315	0.9391	0.9003	0.9795
**ukb-e-30770_CSA**	IGF-1	acute RITs	South Asian	Metabolism	30	IVW (fixed effects)	-0.0425	0.0203	0.0364	-0.0822	-0.0027	0.9584	0.9211	0.9973
**ukb-e-recode1_CSA**	Indirect bilirubin	acute RITs	South Asian	Metabolism	19	IVW	0.0547	0.0189	0.0038	0.0176	0.1312	1.0562	1.0178	1.0961
**met-a-323**	Isoleucine	acute RITs	European	Metabolism	18	MR Egger	1.8856	0.7447	0.0222	0.4259	3.3453	6.5902	1.5310	28.3683
**met-a-652**	Isovalerylcarnitine	acute RITs	European	Metabolism	19	IVW	-0.2331	0.0993	0.0189	-0.4276	-0.1661	0.7921	0.6520	0.9622
**ieu-b-110**	LDL cholesterol	acute RITs	European	Metabolism	281	IVW	0.0656	0.0238	0.0059	0.0189	0.1162	1.0678	1.0191	1.1189
**ieu-b-5089**	LDL cholesterol	acute RITs	European	Metabolism	152	IVW	0.0612	0.0259	0.0179	0.0105	0.1415	1.0631	1.0106	1.1184
**met-d-LDL_C**	LDL cholesterol	acute RITs	European	Metabolism	95	IVW	0.0478	0.0242	0.0483	0.0004	0.0953	1.0490	1.0004	1.1000
**ebi-a-GCST90025993**	Lipoprotein (a) levels	acute RITs	European	Metabolism	70	IVW (fixed effects)	0.0814	0.0410	0.0473	0.0010	0.1618	1.0848	1.0010	1.1756
**ebi-a-GCST90002412**	Low density lipoprotein cholesterol levels	acute RITs	European	Metabolism	481	IVW	0.0567	0.0196	0.0039	0.0182	0.0657	1.0583	1.0183	1.0998

**ebi-a-GCST90019485**	MAP/microtubule affinity-regulating kinase 3 measurement	acute RITs	European	Metabolism	25	IVW	0.0622	0.0297	0.0360	0.0041	0.1315	1.0642	1.0041	1.1279
**ebi-a-GCST90019404**	Medium-chain specific acyl-CoA dehydrogenase, mitochondrial measurement	acute RITs	European	Metabolism	21	IVW	-0.0422	0.0168	0.0121	-0.0752	0.1315	0.9586	0.9275	0.9908
**ebi-a-GCST90012047**	Myoglobin levels	acute RITs	European	Metabolism	38	IVW	-0.0637	0.0305	0.0368	-0.1236	0.0922	0.9383	0.8838	0.9961
**met-a-477**	Myristoleate (14:1n5)	acute RITs	European	Metabolism	13	IVW	-0.2354	0.1181	0.0463	-0.4670	0.5183	0.7903	0.6269	0.9962
**met-a-576**	Palmitoleate (16:1n7)	acute RITs	European	Metabolism	8	Weighted median	-0.4191	0.1978	0.0342	-0.8068	-0.0313	0.6577	0.4463	0.9692
**met-c-919**	Phenylalanine	acute RITs	European	Metabolism	22	IVW	-0.0758	0.0375	0.0429	-0.1492	0.1553	0.9270	0.8614	0.9976
**met-a-430**	Phenyllactate (PLA)	acute RITs	European	Metabolism	18	IVW	-0.2876	0.1393	0.0389	-0.5606	0.0528	0.7500	0.5708	0.9855
**ebi-a-GCST90014010**	Phosphate levels (UKB data field 30810)	acute RITs	European	Metabolism	286	Weighted median	-0.1116	0.0528	0.0345	-0.2151	-0.0081	0.8944	0.8064	0.9919
**ebi-a-GCST90060687**	Phosphatidate(34:0)_[M+OAc]1- levels	acute RITs	South Asian	Metabolism	10	IVW	0.1037	0.0425	0.0147	0.0204	0.1315	1.1093	1.0206	1.2056
**ebi-a-GCST90060914**	Phosphatidylcholine(38:7)_[M+OAc]1-/Phosphatidylserine(42:6)_[M-H]1- levels	acute RITs	South Asian	Metabolism	16	MR Egger	0.2687	0.1241	0.0480	0.0256	0.5119	1.3083	1.0259	1.6685
**ebi-a-GCST90060671**	Phosphatidylcholine-O(32:0)_[M+H]1+/Phosphatidylethanolamine-O(35:0)_[M+H]1+ levels	acute RITs	South Asian	Metabolism	18	IVW	0.1410	0.0588	0.0166	0.0256	0.1315	1.1514	1.0260	1.2921
**ebi-a-GCST90060801**	Phosphatidylcholine-O(38:4)_[M+H]1+/Phosphatidylcholine-P(38:3)_[M+H]1+ levels	acute RITs	South Asian	Metabolism	17	MR Egger	0.1260	0.0549	0.0364	0.0185	0.2336	1.1343	1.0187	1.2631
**ebi-a-GCST90060961**	Phosphatidylserine(40:5)_[M+OAc]1- levels	acute RITs	South Asian	Metabolism	26	Weighted median	0.0776	0.0387	0.0453	0.0016	0.1535	1.0806	1.0016	1.1659
**met-d-XL_VLDL_PL**	Phospholipids in very large VLDL	acute RITs	European	Metabolism	140	Weighted median	-0.0786	0.0376	0.0365	-0.1522	-0.0049	0.9245	0.8588	0.9951
**met-d-L_VLDL_PL_pct**	Phospholipids to total lipids ratio in large VLDL	acute RITs	European	Metabolism	145	MR Egger	-0.0770	0.0336	0.0232	-0.1428	-0.0112	0.9259	0.8670	0.9888
**met-d-M_HDL_PL_pct**	Phospholipids to total lipids ratio in medium HDL	acute RITs	European	Metabolism	182	IVW	-0.0626	0.0229	0.0063	-0.1075	-0.0177	0.9393	0.8980	0.9825
**ebi-a-GCST90092977**	Phospholipids to total lipids ratio in small VLDL	acute RITs	European	Metabolism	137	IVW	0.0460	0.0224	0.0404	0.0020	0.1315	1.0471	1.0020	1.0942
**ieu-a-1012**	Plasma cortisol	acute RITs	European	Metabolism	8	IVW	-0.0596	0.0296	0.0441	-0.1176	0.0660	0.9422	0.8890	0.9984
**met-a-678**	Pro-hydroxy-pro	acute RITs	European	Metabolism	18	Weighted median	0.4603	0.2244	0.0403	0.0204	0.9002	1.5845	1.0206	2.4601
**met-a-574**	Pseudouridine	acute RITs	European	Metabolism	26	IVW	-0.7032	0.2571	0.0062	-1.2070	1.3996	0.4950	0.2991	0.8193
**met-a-501**	Pyroglutamine*	acute RITs	European	Metabolism	18	IVW	-0.2137	0.0961	0.0262	-0.4020	0.6600	0.8076	0.6690	0.9750
**met-d-SFA_pct**	Ratio of saturated fatty acids to total fatty acids	acute RITs	European	Metabolism	77	MR Egger	0.2233	0.1006	0.0295	0.0261	0.4206	1.2502	1.0264	1.5229
**met-d-TG_by_PG**	Ratio of triglycerides to phosphoglycerides	acute RITs	European	Metabolism	173	Weighted median	-0.0743	0.0356	0.0369	-0.1441	-0.0045	0.9284	0.8658	0.9955
**met-d-Remnant_C**	Remnant cholesterol (non-HDL, non-LDL -cholesterol)	acute RITs	European	Metabolism	114	IVW	0.0548	0.0250	0.0286	0.0057	0.1067	1.0564	1.0058	1.1095
**ebi-a-GCST005059**	Serum albumin level	acute RITs	European	Metabolism	18	IVW	0.0248	0.0120	0.0387	0.0013	0.0713	1.0251	1.0013	1.0495
**ebi-a-GCST90018722**	Serum alkaline phosphatase levels	acute RITs	East Asian	Metabolism	104	IVW	0.0530	0.0235	0.0242	0.0069	0.1315	1.0544	1.0069	1.1041
**ebi-a-GCST90025991**	Serum urea levels	acute RITs	European	Metabolism	321	IVW	0.0762	0.0317	0.0161	0.0141	0.1315	1.0792	1.0142	1.1483
**ebi-a-GCST90019384**	SPARC-related modular calcium-binding protein 1 measurement	acute RITs	European	Metabolism	30	MR Egger	0.0838	0.0382	0.0367	0.0089	0.1586	1.0874	1.0090	1.1719
**ebi-a-GCST90060683**	Sphingomyelin(32:1)_[M+OAc	acute RITs	South Asian	Metabolism	20	IVW	-0.1063	0.0531	0.0452	-0.2103	-0.0023	0.8992	0.8104	0.9977

	]1- levels					(fixed effects)								
**ebi-a-GCST90060633**	Sphingomyelin(32:1)_[M-CH3]1- levels	acute RITs	South Asian	Metabolism	16	IVW (fixed effects)	-0.1403	0.0706	0.0470	-0.2787	-0.0018	0.8691	0.7568	0.9982
**ebi-a-GCST90060643**	Sphingomyelin(34:2)_[M-CH3]1- levels	acute RITs	South Asian	Metabolism	11	IVW	0.3068	0.1209	0.0112	0.0699	0.1315	1.3591	1.0723	1.7225
**ebi-a-GCST90060776**	Sphingomyelin(40:1)_[M+H]1+ levels	acute RITs	South Asian	Metabolism	17	MR Egger	0.1344	0.0607	0.0426	0.0155	0.2533	1.1439	1.0156	1.2883
**ebi-a-GCST90060742**	Sphingomyelin(40:2)_[M-CH3]1- levels	acute RITs	South Asian	Metabolism	18	IVW	0.1836	0.0590	0.0018	0.0681	0.1315	1.2015	1.0704	1.3487
**ebi-a-GCST90060814**	Sphingomyelin(41:0)_[M+H]1-levels	acute RITs	South Asian	Metabolism	10	MR Egger	-0.3815	0.1422	0.0278	-0.6603	-0.1028	0.6828	0.5167	0.9023
**ebi-a-GCST90060835**	Sphingomyelin(42:2)_[M+H]1+ levels	acute RITs	South Asian	Metabolism	15	Weighted median	0.1876	0.0937	0.0453	0.0040	0.3712	1.2064	1.0040	1.4495
**ebi-a-GCST90060812**	Sphingomyelin(42:8)_[M+H]1+ levels	acute RITs	South Asian	Metabolism	10	IVW	0.0226	0.0108	0.0356	0.0015	0.1315	1.0229	1.0015	1.0447
**met-d-Total_C**	Total cholesterol	acute RITs	European	Metabolism	113	IVW	0.0612	0.0259	0.0179	0.0105	0.1576	1.0631	1.0106	1.1184
**met-c-898**	Total cholesterol in medium HDL	acute RITs	European	Metabolism	27	IVW	0.0658	0.0298	0.0271	0.0075	0.2297	1.0680	1.0075	1.1321
**ebi-a-GCST90018974**	Total cholesterol levels	acute RITs	European	Metabolism	294	IVW	0.0515	0.0245	0.0356	0.0035	0.1315	1.0528	1.0035	1.1046
**ebi-a-GCST90025953**	Total cholesterol levels	acute RITs	European	Metabolism	281	IVW	0.0551	0.0232	0.0178	0.0095	0.1315	1.0566	1.0096	1.1058
**met-d-non_HDL_C**	Total cholesterol minus HDL-C	acute RITs	European	Metabolism	101	IVW	0.0561	0.0266	0.0353	0.0039	0.1100	1.0577	1.0039	1.1143
**met-d-Total_P**	Total concentration of lipoprotein particles	acute RITs	European	Metabolism	126	IVW	0.0573	0.0273	0.0360	0.0038	0.1694	1.0590	1.0038	1.1172
**met-d-Total_CE**	Total esterified cholesterol	acute RITs	European	Metabolism	113	IVW	0.0631	0.0260	0.0153	0.0121	0.1142	1.0652	1.0122	1.1209
**met-d-Total_FC**	Total free cholesterol	acute RITs	European	Metabolism	113	IVW	0.0532	0.0256	0.0378	0.0030	0.1399	1.0546	1.0030	1.1088
**met-c-957**	Total lipids in chylomicrons and largest VLDL particles	acute RITs	European	Metabolism	25	IVW	-0.0636	0.0286	0.0262	-0.1197	0.0118	0.9384	0.8872	0.9925
**met-d-HDL_L**	Total lipids in HDL	acute RITs	European	Metabolism	164	IVW	0.0496	0.0242	0.0407	0.0021	0.1409	1.0508	1.0021	1.1019
**met-d-L_LDL_L**	Total lipids in large LDL	acute RITs	European	Metabolism	104	IVW	0.0499	0.0238	0.0356	0.0034	0.1038	1.0512	1.0034	1.1013
**met-d-M_HDL_L**	Total lipids in medium HDL	acute RITs	European	Metabolism	139	IVW	0.0563	0.0252	0.0255	0.0069	0.1305	1.0579	1.0069	1.1114
**met-d-S_LDL_L**	Total lipids in small LDL	acute RITs	European	Metabolism	116	IVW	0.0517	0.0227	0.0226	0.0072	0.0911	1.0530	1.0073	1.1008
**met-d-XL_VLDL_L**	Total lipids in very large VLDL	acute RITs	European	Metabolism	145	Weighted median	-0.0798	0.0381	0.0364	-0.1546	-0.0051	0.9233	0.8568	0.9950
**ukb-e-30860_CSA**	Total protein	acute RITs	South Asian	Metabolism	23	MR Egger	-0.1607	0.0759	0.0464	-0.3096	-0.0119	0.8515	0.7338	0.9882
**ebi-a-GCST90060736**	Triacylglycerol(44:1)_[M+NH4]1+ levels	acute RITs	South Asian	Metabolism	19	IVW	-0.0198	0.0100	0.0487	-0.0395	0.1315	0.9804	0.9613	0.9999
**ebi-a-GCST90060999**	Triacylglycerol(56:3)_[M+NH4]1+ levels	acute RITs	South Asian	Metabolism	11	IVW	-0.1254	0.0393	0.0014	-0.2023	0.1315	0.8822	0.8168	0.9528
**ukb-e-30870_CSA**	Triglycerides	acute RITs	South Asian	Metabolism	28	IVW	0.0364	0.0178	0.0410	0.0015	0.1290	1.0370	1.0015	1.0739
**met-d-L_HDL_TG_pct**	Triglycerides to total lipids ratio in large HDL	acute RITs	European	Metabolism	157	Weighted median	-0.0727	0.0330	0.0277	-0.1374	-0.0080	0.9299	0.8716	0.9921
**met-d-L_HDL_TG_pct**	Triglycerides to total lipids ratio in large HDL	acute RITs	European	Metabolism	157	Weighted mode	-0.0618	0.0294	0.0370	-0.1194	-0.0042	0.9401	0.8874	0.9958
**met-d-M_HDL_TG_pct**	Triglycerides to total lipids ratio in medium HDL	acute RITs	European	Metabolism	156	Weighted median	-0.0731	0.0334	0.0286	-0.1386	-0.0076	0.9295	0.8706	0.9924
**met-d-M_HDL_TG_pct**	Triglycerides to total lipids ratio in medium HDL	acute RITs	European	Metabolism	156	Weighted mode	-0.0687	0.0275	0.0134	-0.1226	-0.0149	0.9336	0.8846	0.9852
**met-d-M_LDL_TG_pct**	Triglycerides to total lipids ratio in medium LDL	acute RITs	European	Metabolism	120	MR Egger	-0.0649	0.0278	0.0211	-0.1193	-0.0105	0.9371	0.8875	0.9896
**met-d-M_LDL_TG_pct**	Triglycerides to total lipids ratio in medium LDL	acute RITs	European	Metabolism	120	Weighted mode	-0.0556	0.0260	0.0346	-0.1066	-0.0046	0.9459	0.8989	0.9954
**met-d-S_HDL_TG_pc**	Triglycerides to total lipids	acute RITs	European	Metabolism	172	Weighte	-0.0717	0.0330	0.0300	-0.1365	-0.0069	0.9308	0.8724	0.9931

**t**	ratio in small HDL					d median							
**met-d-S_HDL_TG_pct**	Triglycerides to total lipids ratio in small HDL	acute RITs	European	Metabolism	172	Weighted mode	-0.0709	0.0306	0.0217	-0.1309	-0.0109	0.9315	0.8773	0.9891
**ieu-a-786**	Urate	acute RITs	European	Metabolism	5	IVW	0.6573	0.3199	0.0399	0.0303	2.5204	1.9297	1.0308	3.6123
**met-c-940**	Valine	acute RITs	European	Metabolism	26	IVW	0.0712	0.0325	0.0281	0.0076	0.2519	1.0738	1.0077	1.1444
**ebi-a-GCST90019468**	von Willebrand factor A domain-containing protein 2 measurement	acute RITs	European	Metabolism	18	IVW	-0.0577	0.0201	0.0041	-0.0971	0.1315	0.9439	0.9074	0.9819
**met-a-499**	X-11334	acute RITs	European	Metabolism	25	IVW	0.2904	0.1284	0.0237	0.0387	1.3335	1.3369	1.0395	1.7195
**met-a-520**	X-11470	acute RITs	European	Metabolism	10	IVW (fixed effects)	0.3268	0.1428	0.0221	0.0469	0.6067	1.3865	1.0481	1.8343
**met-a-548**	X-11820	acute RITs	European	Metabolism	12	MR Egger	0.4976	0.1960	0.0294	0.1135	0.8817	1.6447	1.1202	2.4149
**met-a-610**	X-12442--5,8-tetradecadienoate	acute RITs	European	Metabolism	12	IVW	0.2138	0.1001	0.0327	0.0176	0.5942	1.2383	1.0178	1.5067
**met-a-701**	X-13671	acute RITs	European	Metabolism	13	MR Egger	-2.0201	0.9153	0.0495	-3.8141	-0.2262	0.1326	0.0221	0.7975
**met-a-715**	X-14056	acute RITs	European	Metabolism	9	IVW	0.4372	0.1607	0.0065	0.1223	1.0522	1.5483	1.1301	2.1214
**ukb-b-13350**	PCT responsible for patient data: NOTTINGHAM CITY PCT	acute RITs	European	Other	16	IVW	6.5186	3.2047	0.0419	0.2374	94.5872	677.6336	1.2680	362137.8920
**ukb-b-6873**	PCT responsible for patient data: WESTERN CHESHIRE PCT	acute RITs	European	Other	6	IVW	-20.3480	9.7145	0.0362	-39.3885	717.0602	0.0000	0.0000	0.2705
**ukb-b-1539**	PCT where patients GP was registered: BROXTOWE AND HUCKNALL PCT	acute RITs	European	Other	19	IVW	8.3038	3.1284	0.0079	2.1721	104.3861	4039.1087	8.7768	1858809.7596
**ukb-b-6547**	PCT where patients GP was registered: BURY PCT	acute RITs	European	Other	35	IVW	-5.3846	1.7986	0.0028	-8.9099	21.0125	0.0046	0.0001	0.1558
**ukb-b-8205**	PCT where patients GP was registered: CAMDEN PCT	acute RITs	European	Other	13	IVW	10.5995	5.2334	0.0428	0.3420	443.5913	40115.8217	1.4078	1143105409.4280
**ukb-b-2838**	PCT where patients GP was registered: DURHAM AND CHESTER-LE-STREET PCT	acute RITs	European	Other	19	IVW	-7.0895	3.5821	0.0478	-14.1103	102.0995	0.0008	0.0000	0.9337
**ukb-b-19050**	PCT where patients GP was registered: EAST LANCASHIRE TEACHING PCT	acute RITs	European	Other	5	IVW	25.7584	11.7340	0.0281	2.7598	48.7570	153720437216.9070	15.7969	1495859142857010000000.0000
**ukb-b-14654**	PCT where patients GP was registered: ROCHDALE PCT	acute RITs	European	Other	10	IVW	11.5378	5.6762	0.0421	0.4125	247.5472	102520.4772	1.5106	6957575704.7156
**ukb-b-8842**	PCT where patients GP was registered: SOLIHULL PCT	acute RITs	European	Other	6	IVW	-12.9698	6.5599	0.0480	-25.8272	-0.1125	0.0000	0.0000	0.8936
**ukb-b-7288**	3mm asymmetry angle (left)	acute RITs	European	Other	45	Weighted median	0.2344	0.0878	0.0076	0.0623	0.4065	1.2641	1.0642	1.5016
**ukb-e-5108_CSA**	3mm asymmetry angle (right)	acute RITs	South Asian	Other	14	IVW	0.0419	0.0180	0.0202	0.0065	0.1073	1.0428	1.0066	1.0803
**ukb-e-5156_AFR**	3mm asymmetry index (left)	acute RITs	African American or Afro-Caribbean	Other	25	IVW	0.0229	0.0109	0.0353	0.0016	0.0694	1.0232	1.0016	1.0453
**ukb-e-5107_AFR**	3mm strong meridian angle (right)	acute RITs	African American or Afro-Caribbean	Other	13	IVW (fixed effects)	0.0321	0.0160	0.0448	0.0007	0.0635	1.0327	1.0007	1.0656
**ukb-b-4874**	6mm asymmetry angle (right)	acute RITs	European	Other	31	IVW	0.1212	0.0602	0.0440	0.0032	0.6538	1.1289	1.0032	1.2702

**ukb-e-5306_CSA**	6mm index of best keratometry results (left)	acute RITs	South Asian	Other	22	IVW	0.0605	0.0197	0.0021	0.0219	0.1481	1.0624	1.0221	1.1042
**ieu-a-1095**	Age at menarche	acute RITs	European	Other	154	IVW	0.0469	0.0207	0.0234	0.0063	0.1367	1.0480	1.0064	1.0913
**ukb-d-4700_irnt**	Age cataract diagnosed	acute RITs	European	Other	18	IVW	0.0775	0.0328	0.0183	0.0131	0.4136	1.0805	1.0132	1.1524
**ukb-b-1061**	Age high blood pressure diagnosed	acute RITs	European	Other	62	IVW	-0.1132	0.0566	0.0454	-0.2241	0.1773	0.8930	0.7993	0.9977
**ukb-e-2217_AFR**	Age started wearing glasses or contact lenses	acute RITs	African American or Afro-Caribbean	Other	22	Weighted mode	0.0606	0.0283	0.0442	0.0051	0.1161	1.0625	1.0051	1.1231
**ukb-b-5090**	Astigmatism angle (left)	acute RITs	European	Other	26	IVW	-0.1748	0.0839	0.0373	-0.3393	0.5408	0.8396	0.7123	0.9898
**ukb-e-B96_CSA**	B96 Other bacterial agents as the cause of diseases classified to other chapters	acute RITs	South Asian	Other	16	IVW (fixed effects)	-0.0119	0.0053	0.0240	-0.0222	-0.0016	0.9882	0.9780	0.9984
**ukb-e-5086_AFR**	Cylindrical power (left)	acute RITs	African American or Afro-Caribbean	Other	15	IVW (fixed effects)	0.0241	0.0121	0.0468	0.0003	0.0478	1.0243	1.0003	1.0489
**ukb-b-7196**	Delivery methods: Elective caesarean section	acute RITs	European	Other	4	IVW	-0.4206	0.2144	0.0498	-0.8407	-0.0004	0.6567	0.4314	0.9996
**ukb-e-2296_CSA**	Falls in the last year	acute RITs	South Asian	Other	19	IVW	-0.1090	0.0485	0.0245	-0.2040	0.1602	0.8967	0.8154	0.9861
**ukb-e-4935_CSA**	FI1 : numeric addition test	acute RITs	South Asian	Other	16	IVW	-0.0132	0.0064	0.0391	-0.0258	0.0132	0.9869	0.9745	0.9993
**ukb-d-4979**	FI5 : family relationship calculation	acute RITs	European	Other	27	IVW	-0.4732	0.1864	0.0111	-0.8386	0.7872	0.6230	0.4323	0.8978
**ukb-b-17738**	Fractured bone site(s): Other bones	acute RITs	European	Other	30	IVW	1.4558	0.7190	0.0429	0.0467	7.0283	4.2881	1.0478	17.5497
**ukb-a-439**	Fractured bone site(s): Wrist	acute RITs	European	Other	23	IVW	2.3540	1.0368	0.0232	0.3220	11.7940	10.5281	1.3798	80.3288
**ukb-b-9571**	Fractured bone site(s): Wrist	acute RITs	European	Other	26	IVW	2.4754	1.0790	0.0218	0.3606	11.2980	11.8869	1.4342	98.5207
**ebi-a-GCST90013913**	Fractured bones in last 5 years (UKB data field 2463) (Firth correction)	acute RITs	European	Other	67	MR Egger	0.1965	0.0968	0.0464	0.0068	0.3863	1.2172	1.0068	1.4715
**ukb-a-2**	Frequency of light DIY in last 4 weeks	acute RITs	European	Other	25	Weighted median	0.2842	0.1257	0.0238	0.0378	0.5307	1.3288	1.0386	1.7001
**ebi-a-GCST90019399**	Gelsolin measurement	acute RITs	European	Other	20	IVW	-0.0730	0.0323	0.0237	-0.1363	0.1315	0.9296	0.8726	0.9903
**ukb-a-340**	Hearing aid user	acute RITs	European	Other	27	IVW	1.1511	0.5195	0.0267	0.1329	1.2526	3.1617	1.1421	8.7529
**ukb-b-19060**	Hearing aid user	acute RITs	European	Other	40	IVW	1.2710	0.4827	0.0085	0.3250	2.2171	3.5645	1.3840	9.1806
**ukb-e-4849_p2_AFR**	Hearing test done	acute RITs	African American or Afro-Caribbean	Other	25	MR Egger	-0.0062	0.0029	0.0423	-0.0118	-0.0005	0.9938	0.9883	0.9995
**ukb-e-6141_p2_AFR**	How are people in household related to participant	acute RITs	African American or Afro-Caribbean	Other	20	IVW	-0.0194	0.0078	0.0134	-0.0347	0.0138	0.9808	0.9659	0.9960
**ukb-e-6145_p2_AFR**	Illness, injury, bereavement, stress in last 2 years	acute RITs	African American or Afro-Caribbean	Other	31	IVW	0.0127	0.0063	0.0448	0.0003	0.0387	1.0128	1.0003	1.0254
**ukb-e-6145_p4_CSA**	Illness, injury, bereavement, stress in last 2 years	acute RITs	South Asian	Other	13	IVW	-0.0306	0.0145	0.0345	-0.0589	0.0729	0.9699	0.9428	0.9978
**ukb-b-14449**	Index of best refractometry result (left)	acute RITs	European	Other	16	IVW	0.2687	0.1340	0.0449	0.0061	0.7105	1.3083	1.0062	1.7012
**ukb-e-41244_p2_CSA**	Intended management of patient (recoded)	acute RITs	South Asian	Other	20	Weighted median	-0.0310	0.0155	0.0450	-0.0613	-0.0007	0.9695	0.9406	0.9993
**ukb-b-16311**	Interval between previous point and current one in alphanumeric path (trail #2)	acute RITs	European	Other	73	IVW	0.1028	0.0489	0.0353	0.0071	0.5844	1.1083	1.0071	1.2197
**ebi-a-GCST90013453**	Lack of behavioral control	acute RITs	European	Other	7	IVW	-0.0698	0.0302	0.0208	-0.1289	0.0715	0.9326	0.8790	0.9895

**ukb-e-4728_CSA**	Leg pain on walking	acute RITs	South Asian	Other	23	IVW	-0.0262	0.0088	0.0030	-0.0434	0.0231	0.9742	0.9575	0.9911
**ukb-e-6160_p5_AFR**	Leisure/social activities	acute RITs	African American or Afro-Caribbean	Other	16	IVW	-0.0136	0.0061	0.0264	-0.0257	0.0149	0.9865	0.9747	0.9984
**ukb-e-41245_p20_CSA**	Main speciality of consultant (recoded)	acute RITs	South Asian	Other	11	Weighted median	-0.0522	0.0225	0.0203	-0.0964	-0.0081	0.9491	0.9081	0.9919
**ukb-e-41245_p6_AFR**	Main speciality of consultant (recoded)	acute RITs	African American or Afro-Caribbean	Other	18	IVW	0.0125	0.0050	0.0131	0.0026	0.0272	1.0126	1.0026	1.0226
**ukb-a-392**	Maximum workload during fitness test	acute RITs	European	Other	17	IVW	-0.2268	0.0910	0.0127	-0.4051	0.2119	0.7971	0.6669	0.9527
**ebi-a-GCST90002397**	Mean spheric corpuscular volume	acute RITs	European	Other	637	IVW	0.0320	0.0146	0.0288	0.0033	0.0657	1.0325	1.0033	1.0625
**ukb-e-41249_p4_AFR**	Methods of admission to hospital (recoded)	acute RITs	African American or Afro-Caribbean	Other	22	IVW	-0.0139	0.0068	0.0400	-0.0272	0.0040	0.9862	0.9731	0.9994
**ukb-b-5477**	Methods of admission to hospital (recoded): Elective admission	acute RITs	European	Other	237	IVW	-0.7048	0.3307	0.0331	-1.3530	-0.0566	0.4942	0.2585	0.9449
**ukb-b-10655**	Methods of admission to hospital (recoded): Emergency admission	acute RITs	European	Other	61	Weighted median	1.5397	0.7268	0.0341	0.1151	2.9642	4.6630	1.1220	19.3795
**ukb-b-4540**	Methods of admission to hospital (recoded): Emergency admission: Non-injury	acute RITs	European	Other	134	Weighted median	-1.7754	0.8289	0.0322	-3.4000	-0.1509	0.1694	0.0334	0.8600
**ukb-b-19790**	Methods of admission to hospital (recoded): Maternity admission: Post-partum	acute RITs	European	Other	18	IVW	-2.6868	1.3409	0.0451	-5.3150	-0.0585	0.0681	0.0049	0.9432
**ukb-e-41250_p1_AFR**	Methods of discharge from hospital (recoded)	acute RITs	African American or Afro-Caribbean	Other	22	MR Egger	0.0215	0.0101	0.0462	0.0017	0.0413	1.0217	1.0017	1.0422
**ukb-e-52_p9_CSA**	Month of birth	acute RITs	South Asian	Other	5	IVW (fixed effects)	0.0286	0.0141	0.0430	0.0009	0.0563	1.0290	1.0009	1.0579
**ukb-a-12**	Nap during day	acute RITs	European	Other	192	IVW	0.1846	0.0821	0.0246	0.0236	0.3456	1.2027	1.0239	1.4128
**ukb-d-2654_8**	Non-butter spread type details: Other low or reduced fat spread	acute RITs	European	Other	18	Weighted median	-1.3004	0.6333	0.0400	-2.5416	-0.0591	0.2724	0.0787	0.9426
**ukb-d-2654_9**	Non-butter spread type details: Other type of spread/margarine	acute RITs	European	Other	9	IVW	-2.1637	1.0670	0.0426	-4.2550	15.0764	0.1149	0.0142	0.9302
**ukb-d-2654_4**	Non-butter spread type details: Soft (tub) margarine	acute RITs	European	Other	34	IVW	-0.7823	0.3189	0.0142	-1.4073	1.1200	0.4573	0.2448	0.8544
**ukb-e-4291_CSA**	Number of attempts	acute RITs	South Asian	Other	10	IVW (fixed effects)	-0.0259	0.0129	0.0453	-0.0512	-0.0005	0.9745	0.9501	0.9995
**ieu-b-4827**	Number of children ever born measurement	acute RITs	European	Other	15	IVW	0.1082	0.0430	0.0120	0.0238	0.3911	1.1142	1.0241	1.2123
**ukb-b-2988**	Number of fluid intelligence questions attempted within time limit	acute RITs	European	Other	106	IVW	-0.0510	0.0233	0.0285	-0.0966	0.2286	0.9503	0.9079	0.9947
**ukb-e-399_CSA**	Number of incorrect matches in round	acute RITs	South Asian	Other	11	MR Egger	0.1304	0.0559	0.0446	0.0208	0.2400	1.1393	1.0210	1.2712
**ukb-b-1209**	Number of live births	acute RITs	European	Other	78	IVW (fixed effects)	0.1223	0.0612	0.0456	0.0024	0.2423	1.1301	1.0024	1.2742
**ukb-b-6891**	Number of triplets attempted	acute RITs	European	Other	15	IVW	-0.1806	0.0652	0.0056	-0.3084	-0.0529	0.8348	0.7347	0.9485

	(left)													
**ukb-b-4909**	Number of triplets attempted (right)	acute RITs	European	Other	25	IVW	0.1380	0.0559	0.0135	0.0285	0.1971	1.1480	1.0289	1.2809
**ukb-e-4276_AFR**	Number of triplets attempted (right)	acute RITs	African American or Afro-Caribbean	Other	5	IVW	-0.0732	0.0335	0.0291	-0.1389	0.2040	0.9294	0.8703	0.9926
**ebi-a-GCST90012790**	Percentage of invited food questionnaires completed	acute RITs	European	Other	118	Weighted median	-0.0056	0.0027	0.0386	-0.0109	-0.0003	0.9944	0.9891	0.9997
**ukb-e-20018_AFR**	Prospective memory result	acute RITs	African American or Afro-Caribbean	Other	11	IVW	-0.0885	0.0356	0.0128	-0.1582	0.0768	0.9153	0.8536	0.9814
**ukb-e-20019_AFR**	Speech-reception-threshold (SRT) estimate (left)	acute RITs	African American or Afro-Caribbean	Other	16	IVW	-0.0324	0.0150	0.0311	-0.0619	0.0025	0.9681	0.9400	0.9971
**ukb-d-20531**	Victim of sexual assault	acute RITs	European	Other	25	IVW	0.7709	0.3546	0.0297	0.0759	2.5118	2.1616	1.0789	4.3310
**ukb-e-3659_CSA**	Year immigrated to UK (United Kingdom)	acute RITs	South Asian	Other	21	IVW	0.0963	0.0459	0.0359	0.0063	0.2799	1.1011	1.0063	1.2048
**ukb-e-Z53_AFR**	Z53 Persons encountering health services for specifie procedures, not carried out	acute RITs	African American or Afro-Caribbean	Other	15	MR Egger	-0.0286	0.0118	0.0307	-0.0517	-0.0055	0.9718	0.9496	0.9945

Table S2 Results of phenome-wide Mendelian randomization analysis of 181 traits in the Robust category for acute radiation-induced toxicities (RITs)

Exposure ID	Outcome	Trait name	Population	Category	SNPn	method	β	Se	P value	Lo_ci	Up_ci	OR	OR_lci95	OR_uci95
**ieu-a-61**	acute RITs	Waist circumference	European	Anthropometrics	105	IVW	0.0969	0.0409	0.0179	0.0167	0.3838	1.1017	1.0168	1.1937
**ebi-a-GCST90013422**	acute RITs	Ultradistal forearm bone mineral density	European	Anthropometrics	45	IVW	-0.0673	0.0228	0.0031	-0.1119	0.0715	0.9349	0.8941	0.9776
**ukb-e-23127_AFR**	acute RITs	Trunk fat percentage	African American or Afro-Caribbean	Anthropometrics	33	IVW	-0.0469	0.0164	0.0043	-0.0791	0.0540	0.9542	0.9239	0.9854
**ukb-b-17848**	acute RITs	Heel quantitative ultrasound index (QUI), manual entry	European	Anthropometrics	114	IVW	-0.0583	0.0183	0.0014	-0.0941	0.0528	0.9434	0.9102	0.9777
**ukb-b-11364**	acute RITs	Heel bone mineral density (BMD), manual entry	European	Anthropometrics	118	IVW	-0.0547	0.0182	0.0027	-0.0904	0.0399	0.9468	0.9135	0.9812
**ukb-b-8909**	acute RITs	Body fat percentage	European	Anthropometrics	644	IVW	0.0711	0.0339	0.0360	0.0046	0.2587	1.0737	1.0047	1.1476
**ebi-a-GCST90018875**	acute RITs	Lung cancer	European	Disease	25	IVW	0.0498	0.0217	0.0215	0.0073	0.1315	1.0511	1.0074	1.0967
**ebi-a-GCST012876**	acute RITs	Colorectal cancer	European	Disease	36	IVW	-0.0535	0.0188	0.0044	-0.0903	0.0635	0.9479	0.9136	0.9834
**ukb-b-13545**	acute RITs	Cancer code, self-reported: uterine/endometrial cancer	European	Disease	5	IVW	21.5551	7.9916	0.0070	5.8915	259.8108	2297414605.8295	361.9402	14582834247476100.0000
**ukb-a-246**	acute RITs	Seen doctor (GP) for nerves anxiety tension or depression	European	Disease	131	IVW	0.3240	0.1376	0.0185	0.0544	0.5936	1.3826	1.0559	1.8105
**ukb-b-14027**	acute RITs	Non-cancer illness code, self-reported: chickenpox	European	Disease	6	IVW	18.4089	5.9791	0.0021	6.6898	283.0909	98831975.3985	804.1785	12146257123423.7000
**ukb-b-19354**	acute RITs	Diagnoses - main ICD10:	European	Disease	9	IVW	10.2213	3.0951	0.0010	4.1550	16.287	27483.	63.753	118480

		K20 Oesophagitis									7	6981	6	11.6535
**ukb-b-2592**	acute RITs	Non-cancer illness code, self-reported: irritable bowel syndrome	European	Disease	18	IVW	4.8102	1.6789	0.0042	1.5195	22.1987	122.7576	4.5699	3297.5570
**ebi-a-GCST90038626**	acute RITs	Irritable bowel syndrome	NA	Disease	22	IVW	3.5530	1.3483	0.0084	0.9102	0.1315	34.9171	2.4849	490.6377
**ukb-b-18596**	acute RITs	Pain type(s) experienced in last month: Neck or shoulder pain	European	Disease	90	IVW	0.5368	0.2377	0.0239	0.0709	1.0027	1.7106	1.0735	2.7257
**ebi-a-GCST006940**	acute RITs	Neurociticism	European	Disease	286	IVW	0.0929	0.0420	0.0268	0.0107	0.2398	1.0974	1.0108	1.1915
**ebi-a-GCST90018683**	acute RITs	Pollinosis	East Asian	Disease	25	IVW	0.0907	0.0372	0.0149	0.0177	0.1315	1.0949	1.0178	1.1778
**bbj-a-129**	acute RITs	Ischemic stroke	East Asian	Disease	35	IVW	0.0621	0.0213	0.0035	0.0204	0.1563	1.0640	1.0206	1.1093
**ebi-a-GCST90018897**	acute RITs	Periodontal disease	European	Disease	16	IVW	0.0594	0.0254	0.0192	0.0097	0.1315	1.0612	1.0097	1.1153
**ebi-a-GCST008026**	acute RITs	Chronic kidney disease	Hispanic or Latin American	Disease	31	IVW	0.0347	0.0137	0.0113	0.0079	0.0472	1.0353	1.0079	1.0636
**bbj-a-71**	acute RITs	Atrial Fibrillation	East Asian	Disease	67	IVW	0.0199	0.0084	0.0184	0.0033	0.0488	1.0201	1.0034	1.0370
**ukb-e-6152_p1_AFR**	acute RITs	Blood clot, DVT, bronchitis, emphysema, asthma, rhinitis, eczema, allergy diagnosed by doctor	African American or Afro-Caribbean	Disease	16	IVW	0.0176	0.0073	0.0159	0.0033	0.0341	1.0178	1.0033	1.0324
**ukb-e-596_CSA**	acute RITs	Other disorders of bladder	South Asian	Disease	12	IVW	0.0136	0.0049	0.0060	0.0039	0.0344	1.0137	1.0039	1.0235
**ieu-a-30**	acute RITs	Crohn's disease	European	Disease	115	IVW	-0.0142	0.0057	0.0136	-0.0254	0.0043	0.9859	0.9749	0.9971
**ebi-a-GCST90011866**	acute RITs	Systemic lupus erythematosus	East Asian	Disease	78	IVW	-0.0155	0.0055	0.0044	-0.0262	-0.0093	0.9846	0.9741	0.9952
**ebi-a-GCST003045**	acute RITs	Ulcerative colitis	European	Disease	143	IVW	-0.0198	0.0083	0.0172	-0.0360	-0.0044	0.9804	0.9646	0.9965
**ebi-a-GCST003043**	acute RITs	Inflammatory bowel disease	European	Disease	199	IVW	-0.0199	0.0080	0.0133	-0.0356	0.0097	0.9803	0.9650	0.9959
**ieu-a-292**	acute RITs	Inflammatory bowel disease	European	Disease	176	IVW	-0.0202	0.0078	0.0094	-0.0355	0.0196	0.9800	0.9651	0.9951
**ieu-a-970**	acute RITs	Ulcerative colitis	European	Disease	145	IVW	-0.0221	0.0082	0.0067	-0.0381	-0.0045	0.9781	0.9626	0.9939
**ebi-a-GCST90018706**	acute RITs	Type 2 diabetes	East Asian	Disease	174	IVW	-0.0246	0.0098	0.0119	-0.0437	0.1315	0.9757	0.9572	0.9946
**bbj-a-153**	acute RITs	Type 2 diabetes	East Asian	Disease	203	IVW	-0.0285	0.0104	0.0061	-0.0490	0.0049	0.9719	0.9522	0.9919
**ukb-e-K21_CSA**	acute RITs	K21 Gastro-oesophageal reflux disease	South Asian	Disease	10	IVW	-0.0351	0.0109	0.0013	-0.0565	0.0566	0.9656	0.9451	0.9865
**ebi-a-GCST90000026**	acute RITs	Appendicular lean mass	European	Disease	570	IVW	-0.0422	0.0186	0.0233	-0.0786	0.0635	0.9587	0.9244	0.9943
**ieu-a-1169**	acute RITs	Hip osteoarthritis	European	Disease	13	IVW	-0.0470	0.0183	0.0101	-0.0829	-0.0112	0.9540	0.9205	0.9888
**ebi-a-GCST90018625**	acute RITs	Food allergy	East Asian	Disease	10	IVW	-0.0738	0.0289	0.0107	-0.1305	0.1315	0.9289	0.8777	0.9831
**ukb-a-94**	acute RITs	Non-cancer illness code self-reported: hayfever/allergic rhinitis	European	Disease	72	IVW	-0.7422	0.3294	0.0243	-1.3878	2.4490	0.4761	0.2496	0.9080
**ukb-a-521**	acute RITs	Diagnoses - main ICD10: D12 Benign neoplasm of colon rectum anus and anal canal	European	Disease	24	IVW	-3.7761	1.2442	0.0024	-6.2148	-3.6729	0.0229	0.0020	0.2625
**ukb-b-10911**	acute RITs	Diagnoses - secondary ICD10: Z92.2 Personal history of long-term (current) use of other medicaments	European	Disease	19	IVW	-4.9026	1.6296	0.0026	-8.0967	-3.0799	0.0074	0.0003	0.1811
**ukb-a-86**	acute RITs	Non-cancer illness code	European	Disease	25	IVW	-6.7112	2.4351	0.0059	-11.483	3.9309	0.0012	0.0000	0.1439

		self-reported: joint disorder								9				
**ukb-d-K81**	acute RITs	Diagnoses - main ICD10: K81 Cholecystitis	European	Disease	21	IVW	-7.3253	2.3565	0.0019	-11.9441	1.3929	0.0007	0.0000	0.0668
**ukb-b-3145**	acute RITs	Diagnoses - main ICD10: C44.5 Skin of trunk	European	Disease	7	IVW	-15.1271	6.0226	0.0120	-26.9314	-3.3228	0.0000	0.0000	0.0361
**ebi-a-GCST90032488**	acute RITs	Negativibacillus massiliensis abundance in stool	European	Gut microbiota	15	IVW	-0.1690	0.0638	0.0081	-0.2941	0.1315	0.8445	0.7452	0.9571
**ebi-a-GCST90032569**	acute RITs	Succiniclasticum abundance in stool	European	Gut microbiota	30	IVW	-0.1233	0.0504	0.0145	-0.2221	0.1315	0.8840	0.8008	0.9759
**ebi-a-GCST90027824**	acute RITs	Gut microbiota abundance (k_Bacteria.p_Bacteroidetes.c_Bacteroidia.o_Bacteroidales.f_Bacteroidaceae.g_Bacteroides.s_Bacteroides_dorei)	European	Gut microbiota	15	IVW	-0.1066	0.0309	0.0006	-0.1671	0.1315	0.8989	0.8461	0.9550
**ebi-a-GCST90017040**	acute RITs	Gut microbiota abundance (genus Paraprevotella id.962)	European	Gut microbiota	13	IVW	-0.0802	0.0337	0.0174	-0.1463	0.1315	0.9229	0.8639	0.9860
**ebi-a-GCST90027765**	acute RITs	Gut microbiota abundance (k_Bacteria.p_Bacteroidetes.c_Bacteroidia.o_Bacteroidales.f_Porphyromonadaceae.g_Odoribacter.s_Odoribacter_splanchnicus)	European	Gut microbiota	14	IVW	-0.0723	0.0277	0.0090	-0.1266	0.1315	0.9302	0.8811	0.9821
**ebi-a-GCST90027487**	acute RITs	Gut bacterial pathway abundance (KETOGLUCONMET.PWY..ketogluconate.metabolism)	European	Gut microbiota	11	IVW	-0.0508	0.0205	0.0132	-0.0910	0.1315	0.9505	0.9130	0.9894
**ebi-a-GCST90011556**	acute RITs	OTU97_100 (Ruminococcaceae) prevalence	European	Gut microbiota	8	IVW	-0.0417	0.0166	0.0120	-0.0743	0.2470	0.9591	0.9284	0.9909
**ebi-a-GCST90011636**	acute RITs	OTU99_155 (Butyrivibrio) prevalence	European	Gut microbiota	8	IVW	0.0307	0.0117	0.0088	0.0077	0.2470	1.0312	1.0078	1.0551
**ebi-a-GCST90017082**	acute RITs	Gut microbiota abundance (unknown genus id.2001)	European	Gut microbiota	10	IVW	0.1053	0.0418	0.0117	0.0234	0.1315	1.1111	1.0237	1.2059
**ebi-a-GCST90016952**	acute RITs	Gut microbiota abundance (family Streptococcaceae id.1850)	European	Gut microbiota	14	IVW	0.1190	0.0442	0.0071	0.0323	0.1315	1.1264	1.0329	1.2284
**ebi-a-GCST90017101**	acute RITs	Gut microbiota abundance (order Lactobacillales id.1800)	European	Gut microbiota	15	IVW	0.1347	0.0439	0.0022	0.0486	0.1315	1.1442	1.0498	1.2470
**ukb-e-Z09_CSA**	acute RITs	Z09 Follow-up examination after treatment for conditions other than malignant neoplasms	South Asian	Health and hematologic traits	10	IVW	0.0156	0.0046	0.0007	0.0066	0.0346	1.0158	1.0066	1.0250
**ukb-a-251**	acute RITs	Overall health rating	European	Health and hematologic traits	262	IVW	0.1497	0.0621	0.0159	0.0280	0.2715	1.1615	1.0284	1.3119
**ebi-a-GCST90020194**	acute RITs	Inferior Posterior lobe of cerebellar volume (including Crus II to IX hemispheric lobules)	European	Health and hematologic traits	73	IVW	-0.1103	0.0435	0.0112	-0.1955	0.1315	0.8955	0.8224	0.9752
**ebi-a-GCST008029**	acute RITs	Diastolic blood pressure	Hispanic or Latin American	Health and hematologic traits	53	IVW	0.0064	0.0028	0.0198	0.0010	0.0301	1.0064	1.0010	1.0119
**ukb-d-30040_irnt**	acute RITs	Mean corpuscular volume	European	Health and hematologic traits	477	IVW	0.0445	0.0157	0.0047	0.0137	0.1025	1.0455	1.0138	1.0782

**ebi-a-GCST004602**	acute RITs	Mean corpuscular volume	European	Health and hematologic traits	314	IVW	0.0366	0.0145	0.0117	0.0081	0.0873	1.0373	1.0082	1.0673
**ebi-a-GCST90025962**	acute RITs	Mean corpuscular hemoglobin concentration	European	Health and hematologic traits	498	IVW	0.0429	0.0165	0.0092	0.0106	0.1315	1.0439	1.0107	1.0781
**ebi-a-GCST004630**	acute RITs	Mean corpuscular hemoglobin	European	Health and hematologic traits	296	IVW	0.0441	0.0149	0.0030	0.0150	0.0995	1.0451	1.0151	1.0759
**ebi-a-GCST90013925**	acute RITs	Irritability (UKB data field 1940) (SPA correction)	European	Health and hematologic traits	148	IVW	0.0634	0.0280	0.0235	0.0085	0.2973	1.0655	1.0086	1.1256
**ebi-a-GCST90013875**	acute RITs	Irritability (UKB data field 1940) (Firth correction)	European	Health and hematologic traits	148	IVW	0.0634	0.0280	0.0237	0.0085	0.2958	1.0654	1.0085	1.1255
**ebi-a-GCST006944**	acute RITs	Experiencing mood swings	European	Health and hematologic traits	156	IVW	0.1296	0.0585	0.0267	0.0150	0.2398	1.1384	1.0151	1.2767
**ebi-a-GCST90025968**	acute RITs	Systolic blood pressure	European	Health and hematologic traits	498	IVW	0.0763	0.0273	0.0052	0.0228	0.1315	1.0793	1.0230	1.1387
**ebi-a-GCST90001577**	acute RITs	Transitional B cell Absolute Count	European	Health and hematologic traits	22	IVW	0.0423	0.0181	0.0191	0.0069	0.0635	1.0433	1.0070	1.0808
**ebi-a-GCST004601**	acute RITs	Red blood cell count	European	Health and hematologic traits	313	IVW	-0.0497	0.0205	0.0153	-0.0898	-0.0166	0.9516	0.9141	0.9905
**ebi-a-GCST90002356**	acute RITs	Platelet count	Mixed	Health and hematologic traits	48	IVW	0.0534	0.0172	0.0019	0.0197	0.1247	1.0549	1.0199	1.0911
**ebi-a-GCST90001396**	acute RITs	IgD+ CD38- B cell Absolute Count	European	Health and hematologic traits	12	IVW	-0.0493	0.0195	0.0114	-0.0874	0.0635	0.9519	0.9163	0.9890
**ebi-a-GCST90001431**	acute RITs	IgD+ CD38- B cell %lymphocyte	European	Health and hematologic traits	11	IVW	-0.0607	0.0228	0.0076	-0.1053	0.0635	0.9411	0.9000	0.9840
**ebi-a-GCST90001491**	acute RITs	CD39+ activated CD4 regulatory T cell %CD4 regulatory T cell	European	Health and hematologic traits	20	IVW	-0.0234	0.0071	0.0010	-0.0374	0.0635	0.9769	0.9633	0.9906
**ebi-a-GCST90001641**	acute RITs	CD3- lymphocyte %leukocyte	European	Health and hematologic traits	16	IVW	-0.0437	0.0170	0.0102	-0.0771	0.0635	0.9572	0.9258	0.9897
**ebi-a-GCST90001654**	acute RITs	CD28- CD4-CD8- T cell Absolute Count	European	Health and hematologic traits	21	IVW	-0.0308	0.0130	0.0180	-0.0564	0.0635	0.9696	0.9452	0.9947
**ukb-e-4570_AFR**	acute RITs	Friendships satisfaction	African American or Afro-Caribbean	Health and hematologic traits	22	IVW	-0.0382	0.0154	0.0133	-0.0683	0.1014	0.9626	0.9339	0.9921
**prot-a-3084**	acute RITs	Thiamin pyrophosphokinase 1	European	Infection and immunity	17	IVW	-0.0537	0.0211	0.0111	-0.0951	-0.0122	0.9477	0.9093	0.9878
**prot-a-3068**	acute RITs	Tenascin-R	European	Infection and immunity	22	IVW	0.0481	0.0158	0.0024	0.0171	0.1250	1.0492	1.0172	1.0823
**prot-a-2795**	acute RITs	Sorting nexin-7	European	Infection and immunity	24	IVW	0.0413	0.0168	0.0139	0.0084	0.1712	1.0422	1.0084	1.0771
**prot-a-2775**	acute RITs	Single-pass membrane and coiled-coil domain-containing	European	Infection and immunity	16	IVW	0.0564	0.0220	0.0105	0.0132	0.1755	1.0580	1.0133	1.1047

		protein 2												
**prot-a-2709**	acute RITs	Secreted frizzled-related protein 1	European	Infection and immunity	21	IVW	0.0518	0.0176	0.0032	0.0173	0.1357	1.0531	1.0175	1.0900
**prot-a-2642**	acute RITs	Scavenger receptor class F member 2	European	Infection and immunity	19	IVW	-0.0513	0.0175	0.0033	-0.0856	0.0491	0.9500	0.9179	0.9831
**prot-a-1945**	acute RITs	Ribosome-recycling factor, mitochondrial	European	Infection and immunity	21	IVW	0.0429	0.0179	0.0162	0.0079	0.0912	1.0439	1.0080	1.0811
**prot-a-2444**	acute RITs	Receptor-type tyrosine-protein phosphatase H	European	Infection and immunity	18	IVW	-0.0621	0.0166	0.0002	-0.0947	-0.0475	0.9398	0.9097	0.9710
**prot-a-2711**	acute RITs	Pulmonary surfactant-associated protein C	European	Infection and immunity	25	IVW	-0.0425	0.0128	0.0009	-0.0675	0.0408	0.9584	0.9347	0.9827
**prot-a-1615**	acute RITs	Potassium voltage-gated channel subfamily G member 4	European	Infection and immunity	21	IVW	0.0589	0.0204	0.0039	0.0189	0.2358	1.0607	1.0191	1.1039
**prot-a-2299**	acute RITs	Pleckstrin homology domain-containing family A member 1	European	Infection and immunity	22	IVW	0.0623	0.0195	0.0014	0.0241	0.1945	1.0642	1.0244	1.1056
**prot-a-2150**	acute RITs	Oligophrenin-1	European	Infection and immunity	20	IVW	0.0523	0.0185	0.0047	0.0160	0.2282	1.0536	1.0162	1.0925
**prot-a-1908**	acute RITs	Membrane metallo-endopeptidase-like 1	European	Infection and immunity	28	IVW	0.0448	0.0157	0.0043	0.0140	0.0781	1.0458	1.0141	1.0785
**prot-a-1931**	acute RITs	MAGUK p55 subfamily member 6	European	Infection and immunity	17	IVW	0.0655	0.0159	0.0000	0.0343	0.1891	1.0677	1.0349	1.1015
**prot-a-1076**	acute RITs	Low affinity immunoglobulin gamma Fc region receptor III-B	European	Infection and immunity	24	IVW	0.0466	0.0130	0.0003	0.0211	0.0918	1.0477	1.0213	1.0747
**prot-a-1593**	acute RITs	Inositol-trisphosphate 3-kinase A	European	Infection and immunity	11	IVW	0.0886	0.0240	0.0002	0.0416	0.3061	1.0927	1.0425	1.1453
**prot-b-55**	acute RITs	growth differentiation factor 15	European	Infection and immunity	15	IVW	-0.0428	0.0140	0.0022	-0.0702	-0.0214	0.9581	0.9322	0.9847
**prot-a-1046**	acute RITs	Glycosaminoglycan xylosylkinase	European	Infection and immunity	26	IVW	0.0361	0.0149	0.0155	0.0069	0.1375	1.0367	1.0069	1.0674
**prot-a-1102**	acute RITs	Fibroblast growth factor receptor 2	European	Infection and immunity	25	IVW	-0.0463	0.0169	0.0062	-0.0794	0.0560	0.9548	0.9237	0.9869
**prot-a-1019**	acute RITs	Fas apoptotic inhibitory molecule 3	European	Infection and immunity	28	IVW	0.0404	0.0146	0.0056	0.0118	0.1692	1.0412	1.0119	1.0714
**prot-a-1003**	acute RITs	Exostosin-like 2	European	Infection and immunity	17	IVW	0.0498	0.0179	0.0054	0.0147	0.1988	1.0510	1.0148	1.0885
**prot-a-2892**	acute RITs	Estrogen sulfotransferase	European	Infection and immunity	21	IVW	0.0470	0.0155	0.0025	0.0166	0.0714	1.0482	1.0167	1.0806
**prot-a-697**	acute RITs	Casein kinase II 2-alpha:2-beta heterotetramer	European	Infection and immunity	19	IVW	0.0435	0.0182	0.0168	0.0079	0.1557	1.0444	1.0079	1.0823
**prot-a-347**	acute RITs	Calcium/calmodulin-dependent protein kinase type 1D	European	Infection and immunity	21	IVW	0.0526	0.0149	0.0004	0.0233	0.1888	1.0540	1.0236	1.0853
**prot-a-2391**	acute RITs	Brain-specific serine protease 4	European	Infection and immunity	16	IVW	-0.0633	0.0173	0.0003	-0.0972	0.0739	0.9386	0.9074	0.9710
**ebi-a-GCST011084**	acute RITs	COVID-19 (hospitalized vs population) RELEASE 5	European	Infection and immunity	45	IVW	-0.0356	0.0152	0.0193	-0.0655	0.0635	0.9650	0.9366	0.9942
**ebi-a-GCST011082**	acute RITs	COVID-19 (hospitalized vs population) RELEASE 5	European	Infection and immunity	42	IVW	-0.0322	0.0137	0.0191	-0.0591	0.0635	0.9683	0.9426	0.9948
**ebi-a-GCST010779**	acute RITs	COVID-19 (hospitalized vs population) RELEASE 4	European	Infection and immunity	42	IVW	-0.0276	0.0118	0.0189	-0.0507	0.0635	0.9727	0.9505	0.9955
**ebi-a-GCST900**	acute RITs	C-reactive protein levels	European	Infection and	353	IVW	0.0684	0.0258	0.0081	0.0178	0.1315	1.0708	1.0179	1.1263

**25959**				immunity										
**bbj-a-14**	acute RITs	C-reactive protein	East Asian	Infection and immunity	46	IVW	0.0837	0.0288	0.0036	0.0273	0.1747	1.0873	1.0277	1.1505
**prot-a-1533**	acute RITs	Interleukin-4 receptor subunit alpha	European	Infection and immunity	19	IVW	0.0530	0.0183	0.0038	0.0171	0.1245	1.0545	1.0172	1.0931
**prot-a-1479**	acute RITs	Interleukin-16	European	Infection and immunity	18	IVW	-0.0330	0.0140	0.0184	-0.0604	0.0363	0.9675	0.9414	0.9944
**ebi-a-GCST90014002**	acute RITs	C reactive protein levels (UKB data field 30710)	European	Infection and immunity	392	IVW	0.0626	0.0246	0.0110	0.0143	0.1315	1.0646	1.0144	1.1172
**ukb-d-22609_0**	acute RITs	Workplace very dusty: Rarely/never	European	Lifestyle, occupation and family background	19	IVW	-0.8816	0.3183	0.0056	-1.5055	0.1516	0.4141	0.2219	0.7728
**ukb-d-22612_2**	acute RITs	Worked with materials containing asbestos: Often	European	Lifestyle, occupation and family background	16	IVW	2.5025	0.8295	0.0026	0.8766	4.9645	12.2125	2.4028	62.0713
**ukb-b-12067**	acute RITs	Sponge pudding intake	European	Lifestyle, occupation and family background	17	IVW	1.0635	0.3825	0.0054	0.3138	2.4618	2.8964	1.3686	6.1299
**ukb-b-998**	acute RITs	Soya dessert intake	European	Lifestyle, occupation and family background	23	IVW	1.7758	0.5492	0.0012	0.6993	3.5987	5.9051	2.0123	17.3283
**ukb-e-1389_AFR**	acute RITs	Pork intake	African American or Afro-Caribbean	Lifestyle, occupation and family background	23	IVW	-0.0480	0.0177	0.0066	-0.0826	-0.0272	0.9531	0.9207	0.9867
**ukb-e-6179_p3_CSA**	acute RITs	Mineral and other dietary supplements	South Asian	Lifestyle, occupation and family background	12	IVW	0.0416	0.0109	0.0001	0.0202	0.1107	1.0424	1.0204	1.0649
**ukb-b-10054**	acute RITs	Cheese consumers	European	Lifestyle, occupation and family background	21	IVW	0.4135	0.1732	0.0170	0.0740	0.7902	1.5122	1.0768	2.1235
**ukb-b-1707**	acute RITs	Average weekly spirits intake	European	Lifestyle, occupation and family background	54	IVW	-0.2919	0.1250	0.0195	-0.5369	0.9849	0.7468	0.5845	0.9542
**ebi-a-GCST006702**	acute RITs	Parental longevity (combined parental age at death)	European	Lifestyle, occupation and family background	60	IVW	-0.1838	0.0716	0.0102	-0.3241	0.2398	0.8321	0.7232	0.9574
**ukb-b-4024**	acute RITs	Illnesses of mother: Stroke	European	Lifestyle, occupation and family background	19	IVW	1.6110	0.6089	0.0082	0.4175	2.8045	5.0079	1.5182	16.5196
**ukb-a-368**	acute RITs	Work/job satisfaction	European	Lifestyle, occupation and family background	24	IVW	0.2203	0.0902	0.0146	0.0436	0.3851	1.2465	1.0445	1.4875
**ukb-b-18099**	acute RITs	Qualifications: O levels/GCSEs or equivalent	European	Lifestyle, occupation and family	134	IVW	0.4338	0.1597	0.0066	0.1208	1.9383	1.5431	1.1284	2.1102

				background										
**ukb-d-22617_1151**	acute RITs	Job SOC coding: Financial institution managers	European	Lifestyle, occupation and family background	21	IVW	-2.5693	0.9299	0.0057	-4.3920	5.9452	0.0766	0.0124	0.4740
**ukb-d-22601_23193405**	acute RITs	Job coding: other teaching professional including private tutor, tefl (teaching english as a foreign language) teacher; tutor at adult education centre, etc.	European	Lifestyle, occupation and family background	20	IVW	2.8991	0.8189	0.0004	1.2940	6.6754	18.1570	3.6473	90.3888
**ukb-b-3709**	acute RITs	Wants to stop smoking	European	Lifestyle, occupation and family background	19	IVW	-0.2099	0.0729	0.0040	-0.3528	0.7417	0.8107	0.7027	0.9353
**ukb-a-421**	acute RITs	Reason for glasses/contact lenses: For just reading/near work as you are getting older (called 'presbyopia')	European	Lifestyle, occupation and family background	43	IVW	1.1337	0.4626	0.0142	0.2271	4.1067	3.1072	1.2550	7.6932
**ukb-e-1468_p1_CSA**	acute RITs	Cereal type	South Asian	Lifestyle, occupation and family background	23	IVW	0.0239	0.0072	0.0009	0.0098	0.0422	1.0242	1.0098	1.0388
**ukb-a-464**	acute RITs	Vitamin and mineral supplements: Multivitamins +/- minerals	European	Medication use and treatment	47	IVW	0.6782	0.2904	0.0195	0.1089	2.9932	1.9703	1.1151	3.4813
**ukb-b-16956**	acute RITs	Treatment/medication code: thyroxine sodium	European	Medication use and treatment	11	IVW	13.8864	4.6817	0.0030	4.7102	62.8267	1073421.5753	111.0693	10374006079.1460
**ukb-b-11862**	acute RITs	Treatment/medication code: beconase 50micrograms nasal spray	European	Medication use and treatment	10	IVW	8.3394	3.5026	0.0173	1.4742	56.4303	4185.4250	4.3675	4010986.2957
**ukb-b-3255**	acute RITs	Treatment speciality of consultant (recoded): Upper gastrointestinal surgery	European	Medication use and treatment	7	IVW	-16.5261	6.1088	0.0068	-28.4993	-4.5529	0.0000	0.0000	0.0105
**ukb-b-11740**	acute RITs	Medication for cholesterol, blood pressure or diabetes: Cholesterol lowering medication	European	Medication use and treatment	129	IVW	0.2404	0.0962	0.0124	0.0519	0.6031	1.2717	1.0532	1.5356
**ebi-a-GCST90013969**	acute RITs	Cholesterol lowering medication use (UKB data field 6177_1) (SPA correction)	European	Medication use and treatment	121	IVW	0.0402	0.0158	0.0110	0.0092	0.1424	1.0410	1.0092	1.0737
**ebi-a-GCST90013919**	acute RITs	Cholesterol lowering medication use (UKB data field 6177_1) (Firth correction)	European	Medication use and treatment	122	IVW	0.0393	0.0158	0.0131	0.0082	0.3863	1.0401	1.0083	1.0728
**ukb-b-4373**	acute RITs	Operative procedures - secondary OPCS: T85.2 Block dissection of axillary lymph nodes	European	Medication use and treatment	17	IVW	6.3867	2.3066	0.0056	1.8657	10.9076	593.8685	6.4607	54588.7024
**ukb-b-4820**	acute RITs	Operative procedures - secondary OPCS: E13.6 Puncture of maxillary antrum	European	Medication use and treatment	5	IVW	23.9016	8.5527	0.0052	7.1383	349.2162	24007019831.6372	1259.3000	457664588623786000.0000
**ukb-e-41200_p3**	acute RITs	Operative procedures - main	African	Medication use	12	IVW	0.0116	0.0047	0.0128	0.0025	0.0321	1.0116	1.0025	1.0209

**56_AFR**		OPCS4	American or Afro-Caribbean	and treatment									
**ukb-b-7609**	acute RITs	Operative procedures - main OPCS: B28.2 Partial excision of breast NEC	European	Medication use and treatment	24	IVW	4.8776	1.7279	0.0048	1.4910	11.7569	131.3207	4.4416	3882.6157
**ukb-b-13003**	acute RITs	Operation code: sinus surgery	European	Medication use and treatment	6	IVW	-19.2229	7.4735	0.0101	-33.8710	290.6570	0.0000	0.0000	0.0103
**prot-a-47**	acute RITs	Alcohol dehydrogenase 4	European	Metabolism	22	IVW	-0.0460	0.0172	0.0076	-0.0797	0.0761	0.9551	0.9234	0.9879
**prot-a-1370**	acute RITs	15-hydroxyprostaglandin dehydrogenase [NAD(+)]	European	Metabolism	23	IVW	0.0386	0.0161	0.0164	0.0071	0.0738	1.0394	1.0071	1.0727
**prot-a-1086**	acute RITs	D-dimer	European	Metabolism	16	IVW	0.0637	0.0226	0.0048	0.0194	0.2087	1.0658	1.0196	1.1141
**met-a-715**	acute RITs	X-14056	European	Metabolism	9	IVW	0.4372	0.1607	0.0065	0.1223	1.0522	1.5483	1.1301	2.1214
**ebi-a-GCST90060742**	acute RITs	Sphingomyelin(40:2)_[M-CH3]1- levels	South Asian	Metabolism	18	IVW	0.1836	0.0590	0.0018	0.0681	0.1315	1.2015	1.0704	1.3487
**met-a-574**	acute RITs	Pseudouridine	European	Metabolism	26	IVW	-0.7032	0.2571	0.0062	-1.2070	1.3996	0.4950	0.2991	0.8193
**ukb-e-recode1_CSA**	acute RITs	Indirect bilirubin	South Asian	Metabolism	19	IVW	0.0547	0.0189	0.0038	0.0176	0.1312	1.0562	1.0178	1.0961
**ebi-a-GCST90014008**	acute RITs	IGF 1 (UKB data field 30770)	European	Metabolism	532	IVW	-0.0629	0.0215	0.0034	-0.1050	0.1315	0.9391	0.9003	0.9795
**met-a-564**	acute RITs	Gamma-glutamylthreonine*	European	Metabolism	9	IVW	-0.4710	0.1699	0.0056	-0.8039	0.6493	0.6244	0.4476	0.8711
**ebi-a-GCST90025991**	acute RITs	Serum urea levels	European	Metabolism	321	IVW	0.0762	0.0317	0.0161	0.0141	0.1315	1.0792	1.0142	1.1483
**ebi-a-GCST90002412**	acute RITs	Low density lipoprotein cholesterol levels	European	Metabolism	481	IVW	0.0567	0.0196	0.0039	0.0182	0.0657	1.0583	1.0183	1.0998
**ebi-a-GCST90014007**	acute RITs	High density lipoprotein cholesterol levels (UKB data field 30760)	European	Metabolism	458	IVW	0.0551	0.0221	0.0126	0.0118	0.1315	1.0567	1.0119	1.1034
**ebi-a-GCST90025953**	acute RITs	Total cholesterol levels	European	Metabolism	281	IVW	0.0551	0.0232	0.0178	0.0095	0.1315	1.0566	1.0096	1.1058
**ebi-a-GCST90092809**	acute RITs	Apolipoprotein B levels	European	Metabolism	109	IVW	0.0612	0.0249	0.0141	0.0124	0.1315	1.0631	1.0124	1.1163
**ebi-a-GCST90092887**	acute RITs	Concentration of LDL particles	European	Metabolism	104	IVW	0.0665	0.0251	0.0080	0.0174	0.1315	1.0688	1.0175	1.1226
**ieu-b-110**	acute RITs	LDL cholesterol	European	Metabolism	281	IVW	0.0656	0.0238	0.0059	0.0189	0.1162	1.0678	1.0191	1.1189
**ieu-b-4844**	acute RITs	HDL cholesterol	European	Metabolism	153	IVW	0.0516	0.0198	0.0090	0.0129	0.1405	1.0529	1.0129	1.0945
**met-d-HDL_C**	acute RITs	HDL cholesterol	European	Metabolism	178	IVW	0.0511	0.0218	0.0192	0.0083	0.1558	1.0524	1.0084	1.0984
**met-d-HDL_P**	acute RITs	Concentration of HDL particles	European	Metabolism	142	IVW	0.0660	0.0252	0.0087	0.0167	0.1488	1.0682	1.0169	1.1222
**met-d-LDL_P**	acute RITs	Concentration of LDL particles	European	Metabolism	110	IVW	0.0607	0.0248	0.0144	0.0121	0.1094	1.0626	1.0121	1.1156
**met-d-M_HDL_C**	acute RITs	Cholesterol in medium HDL	European	Metabolism	152	IVW	0.0619	0.0247	0.0121	0.0136	0.1537	1.0639	1.0137	1.1166
**met-d-M_HDL_P**	acute RITs	Concentration of medium HDL particles	European	Metabolism	142	IVW	0.0546	0.0245	0.0259	0.0066	0.1373	1.0562	1.0066	1.1082
**met-d-M_HDL_PL_pct**	acute RITs	Phospholipids to total lipids ratio in medium HDL	European	Metabolism	182	IVW	-0.0626	0.0229	0.0063	-0.1075	-0.0177	0.9393	0.8980	0.9825
**ebi-a-GCST90060571**	acute RITs	Fatty acid(16:1)_[M-H]1- levels	South Asian	Metabolism	12	IVW	-0.1035	0.0347	0.0028	-0.1715	0.1315	0.9017	0.8424	0.9651
**ebi-a-GCST90060625**	acute RITs	Diacylglycerol(36:2)_[M+NH4]1+ levels	South Asian	Metabolism	18	IVW	-0.1341	0.0467	0.0041	-0.2256	0.1315	0.8745	0.7980	0.9583
**ebi-a-GCST90060634**	acute RITs	Cholesteryl ester(18:3)_[M+NH4]1+ levels	South Asian	Metabolism	20	IVW	0.1248	0.0470	0.0079	0.0327	0.1315	1.1329	1.0332	1.2422
**ebi-a-GCST90060671**	acute RITs	Phosphatidylcholine-O(32:0)_[M+H]1+/Phosphatidylethano	South Asian	Metabolism	18	IVW	0.1410	0.0588	0.0166	0.0256	0.1315	1.1514	1.0260	1.2921

		lamine-O(35:0)_[M+H]1+ levels												
**ebi-a-GCST90060999**	acute RITs	Triacylglycerol(56:3)_[M+NH4]1+ levels	South Asian	Metabolism	11	IVW	-0.1254	0.0393	0.0014	-0.2023	0.1315	0.8822	0.8168	0.9528
**ebi-a-GCST90007322**	acute RITs	circulating leptin levels adjusted for BMI	European	Metabolism	8	IVW	-0.1798	0.0687	0.0088	-0.3145	0.0657	0.8354	0.7302	0.9558
**ebi-a-GCST90007319**	acute RITs	circulating leptin levels adjusted for BMI	Mixed	Metabolism	10	IVW	-0.1734	0.0651	0.0077	-0.3010	0.0657	0.8408	0.7401	0.9552
**ebi-a-GCST90007309**	acute RITs	circulating leptin levels	Mixed	Metabolism	7	IVW	-0.1793	0.0701	0.0105	-0.3166	0.0657	0.8359	0.7286	0.9589
**ebi-a-GCST90007313**	acute RITs	circulating leptin levels	Mixed	Metabolism	6	IVW	-0.1408	0.0559	0.0118	-0.2504	0.0657	0.8687	0.7785	0.9693
**ebi-a-GCST90007307**	acute RITs	circulating leptin levels	Mixed	Metabolism	15	IVW	-0.1557	0.0589	0.0082	-0.2712	0.0657	0.8558	0.7625	0.9606
**ukb-b-6547**	acute RITs	PCT where patients GP was registered: BURY PCT	European	Other	35	IVW	-5.3846	1.7986	0.0028	-8.9099	21.0125	0.0046	0.0001	0.1558
**ukb-b-1539**	acute RITs	PCT where patients GP was registered: BROXTOWE AND HUCKNALL PCT	European	Other	19	IVW	8.3038	3.1284	0.0079	2.1721	104.3861	4039.1087	8.7768	1858809.7596
**ukb-e-20018_AFR**	acute RITs	Prospective memory result	African American or Afro-Caribbean	Other	11	IVW	-0.0885	0.0356	0.0128	-0.1582	0.0768	0.9153	0.8536	0.9814
**ukb-b-6891**	acute RITs	Number of triplets attempted (left)	European	Other	15	IVW	-0.1806	0.0652	0.0056	-0.3084	-0.0529	0.8348	0.7347	0.9485
**ukb-b-2988**	acute RITs	Number of fluid intelligence questions attempted within time limit	European	Other	106	IVW	-0.0510	0.0233	0.0285	-0.0966	0.2286	0.9503	0.9079	0.9947
**ieu-b-4827**	acute RITs	Number of children ever born measurement	European	Other	15	IVW	0.1082	0.0430	0.0120	0.0238	0.3911	1.1142	1.0241	1.2123
**ukb-a-12**	acute RITs	Nap during day	European	Other	192	IVW	0.1846	0.0821	0.0246	0.0236	0.3456	1.2027	1.0239	1.4128
**ukb-b-5477**	acute RITs	Methods of admission to hospital (recoded): Elective admission	European	Other	237	IVW	-0.7048	0.3307	0.0331	-1.3530	-0.0566	0.4942	0.2585	0.9449
**ukb-a-392**	acute RITs	Maximum workload during fitness test	European	Other	17	IVW	-0.2268	0.0910	0.0127	-0.4051	0.2119	0.7971	0.6669	0.9527
**ukb-e-4728_CSA**	acute RITs	Leg pain on walking	South Asian	Other	23	IVW	-0.0262	0.0088	0.0030	-0.0434	0.0231	0.9742	0.9575	0.9911
**ukb-b-19060**	acute RITs	Hearing aid user	European	Other	40	IVW	1.2710	0.4827	0.0085	0.3250	2.2171	3.5645	1.3840	9.1806
**ukb-d-4979**	acute RITs	FI5 : family relationship calculation	European	Other	27	IVW	-0.4732	0.1864	0.0111	-0.8386	0.7872	0.6230	0.4323	0.8978
**ukb-e-5306_CSA**	acute RITs	6mm index of best keratometry results (left)	South Asian	Other	22	IVW	0.0605	0.0197	0.0021	0.0219	0.1481	1.0624	1.0221	1.1042

Table S3 Heterogeneity test results for univariate Mendelian randomization between 990 traits and acute radiation-induced toxicities (RITs)

**Exposure ID**	**Outcome**	**Trait name**	**Method**	**Q**	**Q_df**	**Q_pval**
**ebi-a-GCST90011301**	acute RITs	C_Actinobacteria abundance	MR Egger	17.6854	15	0.2796
**ebi-a-GCST90011305**	acute RITs	C_Clostridia abundance	MR Egger	21.5147	20	0.3674
**ebi-a-GCST90011316**	acute RITs	F_Rikenellaceae abundance	MR Egger	12.0099	12	0.4449

**ebi-a-GCST90011330**	acute RITs	G_Faecalibacterium abundance	MR Egger	8.1083	10	0.6183
**ebi-a-GCST90011333**	acute RITs	G_Lachnospiraceae abundance	MR Egger	7.8611	10	0.6424
**ebi-a-GCST90027467**	acute RITs	Gut bacterial pathway abundance (FUC.RHAMCAT.PWY..superpathway.of.fucose.and.rhamnose.degradation)	MR Egger	4.4793	5	0.4827
**ebi-a-GCST90027487**	acute RITs	Gut bacterial pathway abundance (KETOGLUCONMET.PWY..ketogluconate.metabolism)	MR Egger	4.9434	9	0.8392
**ebi-a-GCST90027501**	acute RITs	Gut bacterial pathway abundance (P42.PWY..incomplete.reductive.TCA.cycle)	MR Egger	12.3292	12	0.4196
**ebi-a-GCST90027548**	acute RITs	Gut bacterial pathway abundance (PWY.5345..superpathway.of.L.methionine.biosynthesis..by.sulfhydrylation.)	MR Egger	5.1444	8	0.7420
**ebi-a-GCST90027577**	acute RITs	Gut bacterial pathway abundance (PWY.6285..superpathway.of.fatty.acids.biosynthesis..E..coli.)	MR Egger	15.3368	10	0.1203
**ebi-a-GCST90027623**	acute RITs	Gut bacterial pathway abundance (PWY.7371..1.4.dihydroxy.6.naphthoate.biosynthesis.II)	MR Egger	1.6028	7	0.9785
**ebi-a-GCST90027627**	acute RITs	Gut bacterial pathway abundance (PWY.7456..mannan.degradation)	MR Egger	1.9555	3	0.5817
**ebi-a-GCST90027635**	acute RITs	Gut bacterial pathway abundance (PWY_REDCITCYC..TCA.cycle.VIII..helicobacter.)	MR Egger	9.2148	9	0.4177
**ebi-a-GCST90027520**	acute RITs	Gut bacterial pathway abundance (PWY0.1338..polymyxin.resistance)	MR Egger	6.9549	8	0.5415
**ebi-a-GCST90027524**	acute RITs	Gut bacterial pathway abundance (PWY0.1586..peptidoglycan.maturation..meso.diaminopimelate.containing.)	MR Egger	6.1088	7	0.5271
**ebi-a-GCST90027640**	acute RITs	Gut bacterial pathway abundance (RHAMCAT.PWY..L.rhamnose.degradation.I)	MR Egger	15.4745	10	0.1157
**ebi-a-GCST90016910**	acute RITs	Gut microbiota abundance (class Bacilli id.1673)	MR Egger	23.0270	16	0.1130
**ebi-a-GCST90016927**	acute RITs	Gut microbiota abundance (family Bacteroidaceae id.917)	MR Egger	4.2980	7	0.7449
**ebi-a-GCST90016928**	acute RITs	Gut microbiota abundance (family Bacteroidales S24 7group id.11173)	MR Egger	4.9124	7	0.6707
**ebi-a-GCST90016952**	acute RITs	Gut microbiota abundance (family Streptococcaceae id.1850)	MR Egger	8.4665	12	0.7477
**ebi-a-GCST90016968**	acute RITs	Gut microbiota abundance (genus Bacteroides id.918)	MR Egger	4.2980	7	0.7449
**ebi-a-GCST90017008**	acute RITs	Gut microbiota abundance (genus Family XIII AD3011 group id.11293)	MR Egger	9.5327	11	0.5728
**ebi-a-GCST90017009**	acute RITs	Gut microbiota abundance (genus Family XIII UCG001 id.11294)	MR Egger	3.5130	6	0.7422
**ebi-a-GCST90017028**	acute RITs	Gut microbiota abundance (genus Lachnospiraceae UCG010 id.11330)	MR Egger	7.7622	8	0.4570
**ebi-a-GCST90017040**	acute RITs	Gut microbiota abundance (genus Paraprevotella id.962)	MR Egger	13.7245	11	0.2486
**ebi-a-GCST90017046**	acute RITs	Gut microbiota abundance (genus Rikenellaceae RC9 gut group id.11191)	MR Egger	6.5738	9	0.6814
**ebi-a-GCST90027748**	acute RITs	Gut microbiota abundance (k_Bacteria.p_Actinobacteria)	MR Egger	6.9583	8	0.5411
**ebi-a-GCST90027651**	acute RITs	Gut microbiota abundance (k_Bacteria.p_Actinobacteria.c_Actinobacteria)	MR Egger	6.9583	8	0.5411
**ebi-a-GCST90027824**	acute RITs	Gut microbiota abundance (k_Bacteria.p_Bacteroidetes.c_Bacteroidia.o_Bacteroidales.f_Bacteroidaceae.g_Bacteroides.s_Bacteroides_dorei)	MR Egger	16.1723	13	0.2399
**ebi-a-GCST90027765**	acute RITs	Gut microbiota abundance (k_Bacteria.p_Bacteroidetes.c_Bacteroidia.o_Bacteroidales.f_Porphyromonadaceae.g_Odoribacter.s_Odoribacter_splanchnicus)	MR Egger	11.5117	12	0.4856
**ebi-a-GCST90027803**	acute RITs	Gut microbiota abundance (k_Bacteria.p_Firmicutes.c_Erysipelotrichia.o_Erysipelotrichales.f_Erysipelotrichaceae.g_Erysipelotrichaceae_noname.s_Eubacterium_biforme)	MR Egger	3.0333	6	0.8047
**ebi-a-GCST90027805**	acute RITs	Gut microbiota abundance (k_Bacteria.p_Firmicutes.c_Erysipelotrichia.o_Erysipelotrichales.f_Erysipelotrichaceae.g_Holdemania.s_Holdemania_unclassified)	MR Egger	12.8756	7	0.0752
**ebi-a-GCST90017101**	acute RITs	Gut microbiota abundance (order Lactobacillales id.1800)	MR Egger	13.6538	13	0.3987
**ebi-a-GCST90017112**	acute RITs	Gut microbiota abundance (phylum Cyanobacteria id.1500)	MR Egger	9.8191	6	0.1325

**ebi-a-GCST90017079**	acute RITs	Gut microbiota abundance (unknown genus id.1000005479)	MR Egger	4.9124	7	0.6707
**ebi-a-GCST90017082**	acute RITs	Gut microbiota abundance (unknown genus id.2001)	MR Egger	4.9505	8	0.7629
**ebi-a-GCST90032456**	acute RITs	Lactobacillus B abundance in stool	MR Egger	12.3219	14	0.5805
**ebi-a-GCST90032488**	acute RITs	Negativibacillus massiliensis abundance in stool	MR Egger	11.3344	13	0.5828
**ebi-a-GCST90011556**	acute RITs	OTU97_100 (Ruminococcaceae) prevalence	MR Egger	2.2873	6	0.8915
**ebi-a-GCST90011359**	acute RITs	OTU97_106 (Ruminococcaceae) abundance	MR Egger	6.3411	6	0.3861
**ebi-a-GCST90011570**	acute RITs	OTU97_130 (Butyrivibrio) prevalence	MR Egger	3.6121	5	0.6065
**ebi-a-GCST90011572**	acute RITs	OTU97_138 (Oscillibacter) prevalence	MR Egger	9.4575	10	0.4893
**ebi-a-GCST90011372**	acute RITs	OTU97_140 (Bacteroides) abundance	MR Egger	10.4444	13	0.6572
**ebi-a-GCST90011385**	acute RITs	OTU97_24 (Prevotella) abundance	MR Egger	7.7968	8	0.4536
**ebi-a-GCST90011604**	acute RITs	OTU97_53 (Bacteroides) prevalence	MR Egger	6.4694	13	0.9274
**ebi-a-GCST90011605**	acute RITs	OTU97_56 (Ruminococcaceae) prevalence	MR Egger	5.9301	8	0.6551
**ebi-a-GCST90011636**	acute RITs	OTU99_155 (Butyrivibrio) prevalence	MR Egger	3.5873	6	0.7323
**ebi-a-GCST90011440**	acute RITs	OTU99_171 (Bacteroides) abundance	MR Egger	16.7331	13	0.2118
**ebi-a-GCST90011655**	acute RITs	OTU99_34 (Holdemanella) prevalence	MR Egger	11.8261	12	0.4597
**ebi-a-GCST90011465**	acute RITs	OTU99_558 (Bacteroidales) abundance	MR Egger	16.0911	17	0.5174
**ebi-a-GCST90011675**	acute RITs	OTU99_8 (Ruminococcaceae) prevalence	MR Egger	3.9454	7	0.7860
**ebi-a-GCST90011481**	acute RITs	P_Actinobacteria abundance	MR Egger	17.6854	15	0.2796
**ebi-a-GCST90011482**	acute RITs	P_Bacteroidetes abundance	MR Egger	13.1927	8	0.1054
**ebi-a-GCST90032500**	acute RITs	Parabacteroides sp000436495 abundance in stool	MR Egger	13.0371	10	0.2216
**ebi-a-GCST90032502**	acute RITs	Parachlamydiales abundance in stool	MR Egger	6.3590	8	0.6071
**ebi-a-GCST90032540**	acute RITs	Roseibacillus abundance in stool	MR Egger	6.0417	3	0.1096
**ebi-a-GCST90032569**	acute RITs	Succiniclasticum abundance in stool	MR Egger	26.5269	28	0.5441
**ebi-a-GCST90032578**	acute RITs	Terrisporobacter abundance in stool	MR Egger	14.9984	13	0.3075
**ebi-a-GCST90011687**	acute RITs	TestASV_14 (Lachnospiraceae) prevalence	MR Egger	8.5561	5	0.1281
**ebi-a-GCST90032590**	acute RITs	UBA1033 sp001695555 abundance in stool	MR Egger	13.0985	10	0.2182
**ebi-a-GCST90032601**	acute RITs	UBA1409 abundance in stool	MR Egger	2.7012	10	0.9876
**ebi-a-GCST90032608**	acute RITs	UBA1777 sp900316255 abundance in stool	MR Egger	16.1894	12	0.1827
**ebi-a-GCST90032616**	acute RITs	UBA3855 sp900316885 abundance in stool	MR Egger	11.7143	13	0.5512
**ebi-a-GCST90032618**	acute RITs	UBA6382 abundance in stool	MR Egger	12.3434	11	0.3384
**ebi-a-GCST90032628**	acute RITs	UBA737 sp002451855 abundance in stool	MR Egger	23.0402	15	0.0833
**ieu-b-4810**	acute RITs	Breast cancer	MR Egger	76.1646	76	0.4731
**ukb-a-57**	acute RITs	Cancer code self-reported: prostate cancer	MR Egger	44.5359	40	0.2867
**ukb-b-13545**	acute RITs	Cancer code, self-reported: uterine/endometrial cancer	MR Egger	1.6778	3	0.6419
**ebi-a-GCST90018817**	acute RITs	Cervical cancer	MR Egger	16.8136	19	0.6025
**ebi-a-GCST012876**	acute RITs	Colorectal cancer	MR Egger	46.8361	34	0.0703

**ebi-a-GCST012877**	acute RITs	Colorectal cancer	MR Egger	33.6966	36	0.5786
**ebi-a-GCST90018588**	acute RITs	Colorectal cancer	MR Egger	34.6371	39	0.6691
**ebi-a-GCST90018838**	acute RITs	Endometrial cancer	MR Egger	16.2466	13	0.2361
**ieu-a-1163**	acute RITs	ER- Breast cancer (Survival)	MR Egger	16.5787	20	0.6801
**ebi-a-GCST90018875**	acute RITs	Lung cancer	MR Egger	27.6017	23	0.2312
**ieu-a-985**	acute RITs	Lung cancer	MR Egger	38.5723	39	0.4892
**ebi-a-GCST004747**	acute RITs	Lung cancer in never smokers	MR Egger	17.3038	21	0.6925
**ukb-b-14956**	acute RITs	Type of cancer: ICD10: C54.1 Endometrium	MR Egger	2.5993	2	0.2726
**ebi-a-GCST90001501**	acute RITs	Activated & secreting CD4 regulatory T cell Absolute Count	MR Egger	29.1643	13	0.0062
**ebi-a-GCST90001694**	acute RITs	CD28- CD4-CD8- T cell %T cell	MR Egger	19.3102	18	0.3730
**ebi-a-GCST90001654**	acute RITs	CD28- CD4-CD8- T cell Absolute Count	MR Egger	18.6128	19	0.4819
**ebi-a-GCST90001641**	acute RITs	CD3- lymphocyte %leukocyte	MR Egger	13.1614	14	0.5139
**ebi-a-GCST90001491**	acute RITs	CD39+ activated CD4 regulatory T cell %CD4 regulatory T cell	MR Egger	4.1991	18	0.9997
**ebi-a-GCST90001489**	acute RITs	CD39+ activated CD4 regulatory T cell Absolute Count	MR Egger	9.7684	23	0.9926
**ebi-a-GCST90001495**	acute RITs	CD39+ secreting CD4 regulatory T cell Absolute Count	MR Egger	13.9525	19	0.7865
**ebi-a-GCST90001548**	acute RITs	Central Memory CD8+ T cell Absolute Count	MR Egger	20.7846	19	0.3488
**ebi-a-GCST90001556**	acute RITs	Effector Memory CD8+ T cell %T cell	MR Egger	13.5494	10	0.1946
**ebi-a-GCST90001554**	acute RITs	Effector Memory CD8+ T cell Absolute Count	MR Egger	27.9528	20	0.1105
**ukb-e-30210_CSA**	acute RITs	Eosinophill percentage	MR Egger	17.6217	24	0.8210
**ukb-e-30300_AFR**	acute RITs	High light scatter reticulocyte count	MR Egger	28.9945	26	0.3113
**ebi-a-GCST90001441**	acute RITs	IgD+ CD24- B cell %lymphocyte	MR Egger	11.2433	15	0.7352
**ebi-a-GCST90001431**	acute RITs	IgD+ CD38- B cell %lymphocyte	MR Egger	2.5032	9	0.9808
**ebi-a-GCST90001396**	acute RITs	IgD+ CD38- B cell Absolute Count	MR Egger	4.7723	10	0.9059
**ebi-a-GCST90018962**	acute RITs	Lymphocyte count	MR Egger	505.1868	497	0.3900
**ukb-d-30120_irnt**	acute RITs	Lymphocyte count	MR Egger	515.1055	482	0.1436
**ebi-a-GCST004627**	acute RITs	Lymphocyte counts	MR Egger	288.3798	283	0.4003
**bbj-a-44**	acute RITs	Neutrophil count	MR Egger	62.6477	70	0.7216
**ebi-a-GCST90002356**	acute RITs	Platelet count	MR Egger	27.8965	46	0.9839
**ieu-a-1008**	acute RITs	Platelet count	MR Egger	68.5523	70	0.5266
**ebi-a-GCST004601**	acute RITs	Red blood cell count	MR Egger	388.1035	311	0.0019
**ebi-a-GCST90025964**	acute RITs	Red blood cell count	MR Egger	623.6210	566	0.0467
**ebi-a-GCST90001578**	acute RITs	Transitional B cell %lymphocyte	MR Egger	14.3550	20	0.8121
**ebi-a-GCST90001577**	acute RITs	Transitional B cell Absolute Count	MR Egger	11.0098	20	0.9460
**ukb-d-30000_irnt**	acute RITs	White blood cell (leukocyte) count	MR Egger	529.1755	481	0.0636
**ebi-a-GCST90002407**	acute RITs	White blood cell count	MR Egger	630.8128	581	0.0748
**ebi-a-GCST90018978**	acute RITs	White blood cell count	MR Egger	543.5234	518	0.2116

**ebi-a-GCST90025985**	acute RITs	White blood cell count	MR Egger	476.7254	498	0.7463
**ebi-a-GCST010779**	acute RITs	COVID-19 (hospitalized vs population) RELEASE 4	MR Egger	38.3547	40	0.5445
**ebi-a-GCST011082**	acute RITs	COVID-19 (hospitalized vs population) RELEASE 5	MR Egger	42.1309	40	0.3789
**ebi-a-GCST011084**	acute RITs	COVID-19 (hospitalized vs population) RELEASE 5	MR Egger	29.9096	43	0.9349
**ukb-a-28**	acute RITs	Average weekly beer plus cider intake	MR Egger	91.9986	87	0.3364
**ukb-b-1707**	acute RITs	Average weekly spirits intake	MR Egger	64.2180	52	0.1191
**ukb-b-10054**	acute RITs	Cheese consumers	MR Egger	21.0592	19	0.3335
**ukb-b-1814**	acute RITs	Cheesecake intake	MR Egger	30.9119	21	0.0751
**ukb-b-1160**	acute RITs	Chocolate-covered raisin intake	MR Egger	22.3212	25	0.6171
**ukb-b-8089**	acute RITs	Cooked vegetable intake	MR Egger	99.5416	118	0.8900
**ukb-b-5382**	acute RITs	Eggs in sandwiches intake	MR Egger	15.6105	11	0.1562
**ukb-b-14351**	acute RITs	Grapefruit juice intake	MR Egger	28.7772	20	0.0922
**ukb-b-18079**	acute RITs	Low fat hard cheese intake	MR Egger	2.5654	9	0.9791
**ukb-d-1418_1**	acute RITs	Milk type used: Full cream	MR Egger	43.2107	36	0.1904
**ukb-d-1418_5**	acute RITs	Milk type used: Other type of milk	MR Egger	21.8021	13	0.0585
**ukb-e-6179_p3_CSA**	acute RITs	Mineral and other dietary supplements	MR Egger	8.0455	10	0.6244
**ukb-e-1389_AFR**	acute RITs	Pork intake	MR Egger	5.5797	21	0.9997
**ukb-b-8006**	acute RITs	Poultry intake	MR Egger	95.3162	91	0.3578
**ukb-b-337**	acute RITs	Pure fruit/vegetable juice intake	MR Egger	15.8382	15	0.3929
**ukb-e-1478_AFR**	acute RITs	Salt added to food	MR Egger	21.0756	18	0.2756
**ukb-b-5847**	acute RITs	Sausage intake	MR Egger	10.3350	11	0.5005
**ukb-b-998**	acute RITs	Soya dessert intake	MR Egger	21.7391	21	0.4147
**ukb-b-12067**	acute RITs	Sponge pudding intake	MR Egger	20.5327	15	0.1524
**ukb-b-15984**	acute RITs	Thickness of butter/margarine spread on sliced bread: thin	MR Egger	9.8633	14	0.7721
**ukb-b-16523**	acute RITs	Type of meals eaten: Bought sandwiches	MR Egger	5.5814	3	0.1338
**ukb-b-6448**	acute RITs	Type of sliced bread eaten: mixed	MR Egger	20.0968	16	0.2159
**ukb-b-3828**	acute RITs	Types of spread used on bread/crackers: Normal fat polyunsaturated margarine on bread/crackers	MR Egger	2.0276	3	0.5667
**ukb-b-11725**	acute RITs	Types of spreads/sauces consumed: Tomato-based sauce	MR Egger	0.6010	2	0.7404
**ukb-b-3383**	acute RITs	Vegetarian sausages/burgers intake	MR Egger	17.2816	20	0.6346
**ukb-b-5427**	acute RITs	White fish intake	MR Egger	4.5159	5	0.4778
**ukb-b-2375**	acute RITs	Whole-wheat cereal intake	MR Egger	11.6072	15	0.7085
**ukb-b-7753**	acute RITs	Yogurt intake	MR Egger	11.4210	9	0.2480
**ukb-d-M13_ADHCAPSULITIS**	acute RITs	Adhesive capsulitis of shoulder	MR Egger	33.9093	23	0.0665
**ebi-a-GCST90086041**	acute RITs	Allergic rhinitis	MR Egger	10.1862	18	0.9257
**ebi-a-GCST005529**	acute RITs	Ankylosing spondylitis	MR Egger	34.1500	39	0.6905
**ieu-a-45**	acute RITs	Anorexia nervosa	MR Egger	1.6137	7	0.9781

**ukb-d-KRA_PSY_ANYMENTAL**	acute RITs	Any mental disorder	MR Egger	28.2161	17	0.0425
**ebi-a-GCST90000026**	acute RITs	Appendicular lean mass	MR Egger	570.7923	568	0.4592
**bbj-a-86**	acute RITs	Arrhythmia	MR Egger	41.4856	51	0.8266
**bbj-a-71**	acute RITs	Atrial Fibrillation	MR Egger	68.3937	65	0.3628
**ukb-b-8463**	acute RITs	Back pain for 3+ months	MR Egger	38.3312	23	0.0235
**ukb-e-41_CSA**	acute RITs	Bacterial infection NOS	MR Egger	8.9329	10	0.5385
**ukb-a-444**	acute RITs	Blood clot DVT bronchitis emphysema asthma rhinitis eczema allergy diagnosed by doctor: Emphysema/chronic bronchitis	MR Egger	29.5313	19	0.0581
**ukb-e-6152_p1_AFR**	acute RITs	Blood clot, DVT, bronchitis, emphysema, asthma, rhinitis, eczema, allergy diagnosed by doctor	MR Egger	10.1900	14	0.7482
**ebi-a-GCST90038652**	acute RITs	Bone disorder	MR Egger	69.4882	63	0.2682
**ebi-a-GCST90018591**	acute RITs	Cardiac valvular disease	MR Egger	23.0425	17	0.1479
**ebi-a-GCST90018812**	acute RITs	Cardiomegaly	MR Egger	22.3535	19	0.2670
**ukb-e-366_CSA**	acute RITs	Cataract	MR Egger	7.7884	11	0.7321
**ieu-a-1058**	acute RITs	Celiac disease	MR Egger	22.7714	27	0.6972
**ebi-a-GCST90018818**	acute RITs	Cholecystitis	MR Egger	55.1272	46	0.1676
**ieu-b-4971**	acute RITs	Cholecystitits	MR Egger	17.5963	19	0.5495
**ebi-a-GCST90013939**	acute RITs	Cholelithiasis gall stones (SPA correction)	MR Egger	78.6309	73	0.3052
**ebi-a-GCST90013939**	acute RITs	Cholelithiasis gall stones (SPA correction)	MR Egger	78.6309	73	0.3052
**ebi-a-GCST008026**	acute RITs	Chronic kidney disease	MR Egger	28.6930	29	0.4811
**ebi-a-GCST90018809**	acute RITs	Chronic suppurative otitis media	MR Egger	12.9287	15	0.6078
**ukb-e-851_CSA**	acute RITs	Complications of transplants and reattached limbs	MR Egger	18.2640	14	0.1950
**ukb-e-5264_AFR**	acute RITs	Corneal hysteresis (left)	MR Egger	22.8129	28	0.7423
**ebi-a-GCST004132**	acute RITs	Crohn's disease	MR Egger	157.3567	181	0.8972
**ieu-a-30**	acute RITs	Crohn's disease	MR Egger	116.5096	113	0.3915
**ukb-e-D64_CSA**	acute RITs	D64 Other anaemias	MR Egger	11.1785	10	0.3438
**ukb-b-3145**	acute RITs	Diagnoses - main ICD10: C44.5 Skin of trunk	MR Egger	3.4211	5	0.6354
**ukb-d-C67**	acute RITs	Diagnoses - main ICD10: C67 Malignant neoplasm of bladder	MR Egger	18.4797	22	0.6772
**ukb-a-521**	acute RITs	Diagnoses - main ICD10: D12 Benign neoplasm of colon rectum anus and anal canal	MR Egger	16.1111	22	0.8103
**ukb-d-D23**	acute RITs	Diagnoses - main ICD10: D23 Other benign neoplasms of skin	MR Egger	12.4159	15	0.6473
**ukb-d-H33**	acute RITs	Diagnoses - main ICD10: H33 Retinal detachments and breaks	MR Egger	33.3548	33	0.4500
**ukb-b-11771**	acute RITs	Diagnoses - main ICD10: K08.3 Retained dental root	MR Egger	1.5203	1	0.2176
**ukb-b-19354**	acute RITs	Diagnoses - main ICD10: K20 Oesophagitis	MR Egger	1.0703	7	0.9936
**ukb-b-11369**	acute RITs	Diagnoses - main ICD10: K40.2 Bilateral inguinal hernia, without obstruction or gangrene	MR Egger	5.8191	5	0.3242
**ukb-b-19805**	acute RITs	Diagnoses - main ICD10: K62.1 Rectal polyp	MR Egger	8.3441	8	0.4006
**ukb-b-8988**	acute RITs	Diagnoses - main ICD10: K80.1 Calculus of gallbladder with other cholecystitis	MR Egger	24.1206	18	0.1511
**ukb-b-8988**	acute RITs	Diagnoses - main ICD10: K80.1 Calculus of gallbladder with other cholecystitis	MR Egger	24.1206	18	0.1511
**ukb-d-K81**	acute RITs	Diagnoses - main ICD10: K81 Cholecystitis	MR Egger	14.5925	19	0.7481

**ukb-d-K92**	acute RITs	Diagnoses - main ICD10: K92 Other diseases of digestive system	MR Egger	9.4227	13	0.7403
**ukb-d-L57**	acute RITs	Diagnoses - main ICD10: L57 Skin changes due to chronic exposure to nonionising radiation	MR Egger	27.5510	32	0.6914
**ukb-b-15003**	acute RITs	Diagnoses - main ICD10: L72.0 Epidermal cyst	MR Egger	4.9658	6	0.5482
**ukb-b-7700**	acute RITs	Diagnoses - main ICD10: M17.1 Other primary gonarthrosis	MR Egger	9.2539	16	0.9026
**ukb-b-9694**	acute RITs	Diagnoses - main ICD10: M23.22 Derangement of meniscus due to old tear or injury (Posterior cruciate ligament or Posterior horn of medial meniscus)	MR Egger	8.0155	4	0.0910
**ukb-d-M70**	acute RITs	Diagnoses - main ICD10: M70 Soft tissue disorders related to use, overuse and pressure	MR Egger	13.5559	13	0.4058
**ukb-b-18372**	acute RITs	Diagnoses - main ICD10: N20.0 Calculus of kidney	MR Egger	13.4222	14	0.4936
**ukb-b-18629**	acute RITs	Diagnoses - main ICD10: N20.1 Calculus of ureter	MR Egger	11.6735	11	0.3887
**ukb-d-O80**	acute RITs	Diagnoses - main ICD10: O80 Single spontaneous delivery	MR Egger	10.2145	15	0.8060
**ukb-b-6608**	acute RITs	Diagnoses - main ICD10: R10.1 Pain localised to upper abdomen	MR Egger	4.5641	7	0.7130
**ukb-b-17456**	acute RITs	Diagnoses - main ICD10: R10.3 Pain localised to other parts of lower abdomen	MR Egger	12.2184	10	0.2707
**ukb-a-584**	acute RITs	Diagnoses - main ICD10: R14 Flatulence and related conditions	MR Egger	27.9030	22	0.1789
**ukb-d-S61**	acute RITs	Diagnoses - main ICD10: S61 Open wound of wrist and hand	MR Egger	11.8499	18	0.8549
**ukb-d-Z45**	acute RITs	Diagnoses - main ICD10: Z45 Adjustment and management of implanted device	MR Egger	18.0963	16	0.3183
**ukb-b-16056**	acute RITs	Diagnoses - secondary ICD10: F10.1 Harmful use	MR Egger	2.2063	5	0.8199
**ukb-b-19651**	acute RITs	Diagnoses - secondary ICD10: F10.2 Dependence syndrome	MR Egger	9.7676	10	0.4611
**ukb-b-16751**	acute RITs	Diagnoses - secondary ICD10: J44.9 Chronic obstructive pulmonary disease, unspecified	MR Egger	33.4565	26	0.1493
**ukb-b-11675**	acute RITs	Diagnoses - secondary ICD10: K66.0 Peritoneal adhesions	MR Egger	1.8200	3	0.6106
**ukb-b-20233**	acute RITs	Diagnoses - secondary ICD10: M19.99 Arthrosis, unspecified (Site unspecified)	MR Egger	10.5155	7	0.1612
**ukb-b-6413**	acute RITs	Diagnoses - secondary ICD10: N73.6 Female pelvic peritoneal adhesions	MR Egger	5.8483	4	0.2108
**ukb-b-10490**	acute RITs	Diagnoses - secondary ICD10: Z03.8 Observation for other suspected diseases and conditions	MR Egger	0.9287	9	0.9996
**ukb-b-2895**	acute RITs	Diagnoses - secondary ICD10: Z60.2 Living alone	MR Egger	0.4973	2	0.7799
**ukb-b-10911**	acute RITs	Diagnoses - secondary ICD10: Z92.2 Personal history of long-term (current) use of other medicaments	MR Egger	12.7922	17	0.7500
**ukb-d-IX_CIRCULATORY**	acute RITs	Diseases of the circulatory system	MR Egger	70.3051	59	0.1489
**ukb-a-254**	acute RITs	Doctor diagnosed hayfever or allergic rhinitis	MR Egger	65.0377	65	0.4754
**ukb-d-20429**	acute RITs	Easily tired during worst period of anxiety	MR Egger	16.9967	17	0.4546
**ukb-e-F17_CSA**	acute RITs	F17 Mental and behavioral disorders due to use of tobacco	MR Egger	23.8477	15	0.0677
**ebi-a-GCST90018625**	acute RITs	Food allergy	MR Egger	9.1147	8	0.3327
**ebi-a-GCST90000514**	acute RITs	Gastroesophageal reflux disease	MR Egger	208.3521	224	0.7659
**ebi-a-GCST90018635**	acute RITs	Hashimoto thyroiditis	MR Egger	12.3742	9	0.1930
**ukb-e-2247_p1_AFR**	acute RITs	Hearing difficulty/problems	MR Egger	8.6500	12	0.7325
**ebi-a-GCST90038627**	acute RITs	Hepatitis	MR Egger	25.3133	21	0.2338
**ukb-d-K11_HERNIA**	acute RITs	Hernia	MR Egger	50.2775	58	0.7546
**ieu-a-1169**	acute RITs	Hip osteoarthritis	MR Egger	8.5723	11	0.6613
**ebi-a-GCST90018860**	acute RITs	Hyperthyroidism	MR Egger	54.6208	49	0.2695

**ebi-a-GCST90018861**	acute RITs	Hypertrophic cardiomyopathy	MR Egger	14.2952	14	0.4280
**ukb-e-I25_AFR**	acute RITs	I25 Chronic ischaemic heart disease	MR Egger	5.9839	16	0.9883
**ebi-a-GCST003043**	acute RITs	Inflammatory bowel disease	MR Egger	227.0096	197	0.0702
**ieu-a-292**	acute RITs	Inflammatory bowel disease	MR Egger	195.9376	174	0.1220
**ieu-a-294**	acute RITs	Inflammatory bowel disease	MR Egger	225.0009	198	0.0913
**ebi-a-GCST90038626**	acute RITs	Irritable bowel syndrome	MR Egger	18.8719	20	0.5302
**bbj-a-129**	acute RITs	Ischemic stroke	MR Egger	31.9558	33	0.5189
**ukb-e-K21_CSA**	acute RITs	K21 Gastro-oesophageal reflux disease	MR Egger	5.7787	8	0.6720
**ukb-e-M17_CSA**	acute RITs	M17 Gonarthrosis [arthrosis of knee]	MR Egger	8.1512	13	0.8336
**ukb-b-6458**	acute RITs	Mouth/teeth dental problems: Mouth ulcers	MR Egger	126.1222	120	0.3330
**ukb-a-429**	acute RITs	Mouth/teeth dental problems: Painful gums	MR Egger	8.9468	11	0.6268
**ieu-b-18**	acute RITs	multiple sclerosis	MR Egger	133.7718	134	0.4893
**ukb-e-N17_CSA**	acute RITs	N17 Acute renal failure	MR Egger	10.6986	10	0.3815
**ukb-e-N18_AFR**	acute RITs	N18 Chronic renal failure	MR Egger	10.7101	5	0.0574
**ebi-a-GCST006940**	acute RITs	Neurociticism	MR Egger	247.6022	284	0.9417
**ukb-a-230**	acute RITs	Neuroticism score	MR Egger	167.4983	223	0.9978
**ukb-a-81**	acute RITs	Non-cancer illness code self-reported: depression	MR Egger	39.4198	35	0.2788
**ukb-a-94**	acute RITs	Non-cancer illness code self-reported: hayfever/allergic rhinitis	MR Egger	52.6346	70	0.9397
**ukb-a-86**	acute RITs	Non-cancer illness code self-reported: joint disorder	MR Egger	23.1568	23	0.4516
**ukb-e-20002_p95_CSA**	acute RITs	Non-cancer illness code, self-reported	MR Egger	8.5200	13	0.8082
**ukb-b-14027**	acute RITs	Non-cancer illness code, self-reported: chickenpox	MR Egger	1.8543	4	0.7625
**ukb-b-3044**	acute RITs	Non-cancer illness code, self-reported: colitis/not crohns or ulcerative colitis	MR Egger	2.6911	4	0.6108
**ukb-b-14452**	acute RITs	Non-cancer illness code, self-reported: inguinal hernia	MR Egger	5.2654	5	0.3844
**ukb-b-2592**	acute RITs	Non-cancer illness code, self-reported: irritable bowel syndrome	MR Egger	21.3084	16	0.1670
**ukb-b-17670**	acute RITs	Non-cancer illness code, self-reported: multiple sclerosis	MR Egger	8.5976	12	0.7369
**ukb-b-6633**	acute RITs	Non-cancer illness code, self-reported: other renal/kidney problem	MR Egger	3.1770	7	0.8682
**ukb-b-14210**	acute RITs	Non-cancer illness code, self-reported: rectal or colon adenoma/polyps	MR Egger	2.0330	1	0.1539
**ukb-b-15749**	acute RITs	Non-cancer illness code, self-reported: transient ischaemic attack (tia)	MR Egger	0.7250	3	0.8673
**ebi-a-GCST007091**	acute RITs	Osteoarthritis (hip)	MR Egger	74.0959	69	0.3156
**ukb-d-I9_ARTOTH**	acute RITs	Other diseases of arteries and capillaries	MR Egger	23.8581	18	0.1597
**ukb-e-596_CSA**	acute RITs	Other disorders of bladder	MR Egger	7.5203	10	0.6756
**ukb-e-569_AFR**	acute RITs	Other disorders of intestine	MR Egger	12.5801	12	0.4003
**ukb-e-306_CSA**	acute RITs	Other mental disorder	MR Egger	10.8257	9	0.2878
**ukb-a-469**	acute RITs	Pain type(s) experienced in last month: Headache	MR Egger	99.2384	109	0.7378
**ukb-b-18596**	acute RITs	Pain type(s) experienced in last month: Neck or shoulder pain	MR Egger	104.3310	88	0.1128
**ukb-b-9130**	acute RITs	Pain type(s) experienced in last month: None of the above	MR Egger	173.1336	175	0.5257

**ukb-d-M13_DUPUTR YEN**	acute RITs	Palmar fascial fibromatosis [Dupuytren]	MR Egger	74.9046	76	0.5140
**ebi-a-GCST90018897**	acute RITs	Periodontal disease	MR Egger	8.5571	14	0.8583
**ebi-a-GCST90018679**	acute RITs	Pleurisy	MR Egger	6.9747	9	0.6398
**ebi-a-GCST90018683**	acute RITs	Pollinosis	MR Egger	38.8110	23	0.0208
**ebi-a-GCST005581**	acute RITs	Primary biliary cirrhosis	MR Egger	43.3829	41	0.3701
**ebi-a-GCST90013954**	acute RITs	Retinitis pigmentosa (SPA correction)	MR Egger	17.7986	13	0.1653
**ebi-a-GCST005538**	acute RITs	Sarcoidosis	MR Egger	17.6463	11	0.0902
**ukb-a-246**	acute RITs	Seen doctor (GP) for nerves anxiety tension or depression	MR Egger	127.1674	129	0.5291
**ebi-a-GCST90000255**	acute RITs	Severe COVID-19 infection with respiratory failure (analysis I)	MR Egger	13.2410	13	0.4294
**ebi-a-GCST90026414**	acute RITs	Severe insulin-resistant type 2 diabetes	MR Egger	5.3018	10	0.8701
**ukb-d-L12_NONION RADISKIN**	acute RITs	Skin changes due to chronic exposure to nonionizing radiation	MR Egger	27.5510	32	0.6914
**ebi-a-GCST003156**	acute RITs	Systemic lupus erythematosus	MR Egger	90.5280	84	0.2938
**ebi-a-GCST90011866**	acute RITs	Systemic lupus erythematosus	MR Egger	58.2862	76	0.9346
**ieu-a-815**	acute RITs	Systemic lupus erythematosus	MR Egger	4.5097	8	0.8085
**bbj-a-153**	acute RITs	Type 2 diabetes	MR Egger	185.5136	201	0.7763
**ebi-a-GCST90018706**	acute RITs	Type 2 diabetes	MR Egger	153.2660	172	0.8445
**ebi-a-GCST003045**	acute RITs	Ulcerative colitis	MR Egger	160.7781	141	0.1218
**ieu-a-968**	acute RITs	Ulcerative colitis	MR Egger	144.5327	116	0.0374
**ieu-a-970**	acute RITs	Ulcerative colitis	MR Egger	155.3815	143	0.2263
**ieu-b-5063**	acute RITs	URTI	MR Egger	14.3568	13	0.3492
**ebi-a-GCST90018718**	acute RITs	Uveitis	MR Egger	2.7072	3	0.4390
**ukb-e-20107_p11_CS A**	acute RITs	Illnesses of father	MR Egger	22.7980	16	0.1192
**ukb-a-201**	acute RITs	Illnesses of father: Heart disease	MR Egger	67.9014	65	0.3786
**ukb-a-202**	acute RITs	Illnesses of father: None of the above (group 1)	MR Egger	30.3928	31	0.4971
**ukb-b-15169**	acute RITs	Illnesses of father: None of the above (group 1)	MR Egger	66.2234	43	0.0130
**ukb-b-10415**	acute RITs	Illnesses of father: None of the above (group 2)	MR Egger	27.0057	34	0.7973
**ukb-b-4024**	acute RITs	Illnesses of mother: Stroke	MR Egger	12.3584	17	0.7780
**ukb-b-18042**	acute RITs	Illnesses of siblings: Diabetes	MR Egger	85.0003	78	0.2751
**ukb-b-10783**	acute RITs	Illnesses of siblings: None of the above (group 2)	MR Egger	20.7161	33	0.9526
**ukb-a-304**	acute RITs	Number of children fathered	MR Egger	26.0320	31	0.7198
**ebi-a-GCST006702**	acute RITs	Parental longevity (combined parental age at death)	MR Egger	43.5928	58	0.9199
**ebi-a-GCST006699**	acute RITs	Parental longevity (mother's age at death)	MR Egger	33.9812	34	0.4686
**ebi-a-GCST006696**	acute RITs	Parental longevity (mother's attained age)	MR Egger	53.6722	53	0.4484
**prot-a-2235**	acute RITs	[Pyruvate dehydrogenase (acetyl-transferring)] kinase isozyme 1, mitochondrial	MR Egger	12.1016	10	0.2783
**prot-a-3251**	acute RITs	14-3-3 protein zeta/delta	MR Egger	23.2777	18	0.1801

**prot-a-1370**	acute RITs	15-hydroxyprostaglandin dehydrogenase [NAD(+)]	MR Egger	25.8032	21	0.2141
**prot-a-11**	acute RITs	Acetyl-CoA carboxylase 2	MR Egger	6.0483	17	0.9929
**prot-a-105**	acute RITs	Acidic leucine-rich nuclear phosphoprotein 32 family member B	MR Egger	20.9154	22	0.5260
**prot-a-43**	acute RITs	Adhesion G-protein coupled receptor F1	MR Egger	21.9598	17	0.1863
**prot-a-47**	acute RITs	Alcohol dehydrogenase 4	MR Egger	21.4664	20	0.3701
**prot-a-1155**	acute RITs	Alpha-(1,3)-fucosyltransferase 9	MR Egger	10.7067	14	0.7089
**ebi-a-GCST90006919**	acute RITs	Anti-herpes simplex virus 2 IgG seropositivity	MR Egger	13.8563	16	0.6094
**ebi-a-GCST90006921**	acute RITs	Anti-polyomavirus 2 IgG seropositivity	MR Egger	17.8640	16	0.3319
**prot-a-180**	acute RITs	Arf-GAP with SH3 domain, ANK repeat and PH domain-containing protein 2	MR Egger	30.5199	20	0.0619
**prot-a-204**	acute RITs	Aurora kinase A	MR Egger	13.8336	16	0.6111
**ebi-a-GCST90001829**	acute RITs	BAFF-R on B cell	MR Egger	11.0973	12	0.5206
**ebi-a-GCST90001830**	acute RITs	BAFF-R on CD20- B cell	MR Egger	5.8698	4	0.2091
**ebi-a-GCST90001702**	acute RITs	BAFF-R on CD24+ CD27+ B cell	MR Egger	8.0292	11	0.7107
**ebi-a-GCST90001710**	acute RITs	BAFF-R on IgD- CD24- B cell	MR Egger	14.1574	12	0.2908
**ebi-a-GCST90001711**	acute RITs	BAFF-R on IgD- CD27- B cell	MR Egger	12.4196	10	0.2580
**ebi-a-GCST90001712**	acute RITs	BAFF-R on IgD- CD38- B cell	MR Egger	10.8918	11	0.4524
**ebi-a-GCST90001713**	acute RITs	BAFF-R on IgD- CD38+ B cell	MR Egger	9.4910	11	0.5767
**ebi-a-GCST90001719**	acute RITs	BAFF-R on IgD+ B cell	MR Egger	13.0503	15	0.5984
**ebi-a-GCST90001704**	acute RITs	BAFF-R on IgD+ CD24- B cell	MR Egger	13.0409	13	0.4447
**ebi-a-GCST90001703**	acute RITs	BAFF-R on IgD+ CD24+ B cell	MR Egger	2.7161	11	0.9940
**ebi-a-GCST90001705**	acute RITs	BAFF-R on IgD+ CD38- B cell	MR Egger	11.4332	14	0.6517
**ebi-a-GCST90001708**	acute RITs	BAFF-R on IgD+ CD38+ B cell	MR Egger	15.5020	16	0.4882
**ebi-a-GCST90001709**	acute RITs	BAFF-R on IgD+ CD38dim B cell	MR Egger	17.5971	13	0.1734
**ebi-a-GCST90001715**	acute RITs	BAFF-R on memory B cell	MR Egger	6.8319	8	0.5549
**ebi-a-GCST90001716**	acute RITs	BAFF-R on naive-mature B cell	MR Egger	12.4309	14	0.5717
**ebi-a-GCST90001718**	acute RITs	BAFF-R on switched memory B cell	MR Egger	9.9031	10	0.4490
**ebi-a-GCST90001720**	acute RITs	BAFF-R on transitional B cell	MR Egger	22.1946	16	0.1370
**ebi-a-GCST90001717**	acute RITs	BAFF-R on unswitched memory B cell	MR Egger	9.8788	14	0.7710
**prot-a-231**	acute RITs	Basal Cell Adhesion Molecule	MR Egger	15.1884	23	0.8878
**prot-a-218**	acute RITs	Beta-1,4-galactosyltransferase 3	MR Egger	13.9221	8	0.0838
**prot-a-800**	acute RITs	Beta-defensin 128	MR Egger	23.6720	20	0.2570
**prot-a-801**	acute RITs	Beta-defensin 134	MR Egger	12.8374	19	0.8468
**prot-a-1396**	acute RITs	Bone sialoprotein 2	MR Egger	16.0886	16	0.4468
**prot-a-2391**	acute RITs	Brain-specific serine protease 4	MR Egger	10.5752	14	0.7190
**prot-a-232**	acute RITs	Brevican core protein	MR Egger	20.3529	15	0.1588
**prot-a-2947**	acute RITs	Calcineurin B homologous protein 3	MR Egger	18.8905	13	0.1265

**prot-a-2509**	acute RITs	Calcipressin-1	MR Egger	28.3624	17	0.0409
**prot-a-347**	acute RITs	Calcium/calmodulin-dependent protein kinase type 1D	MR Egger	14.8937	19	0.7293
**prot-a-2290**	acute RITs	Calcium-dependent phospholipase A2	MR Egger	26.1186	21	0.2020
**prot-a-557**	acute RITs	Carbohydrate sulfotransferase 5	MR Egger	13.2204	14	0.5092
**prot-a-332**	acute RITs	Carbonic anhydrase 5A, mitochondrial	MR Egger	12.6295	12	0.3965
**prot-a-327**	acute RITs	Carbonic anhydrase-related protein 10	MR Egger	10.0730	20	0.9668
**prot-a-649**	acute RITs	Carboxypeptidase Z	MR Egger	33.6375	19	0.0203
**prot-a-697**	acute RITs	Casein kinase II 2-alpha:2-beta heterotetramer	MR Egger	12.2942	17	0.7820
**prot-a-721**	acute RITs	Cathepsin F	MR Egger	17.2336	16	0.3706
**prot-a-723**	acute RITs	Cathepsin G	MR Egger	18.2980	17	0.3703
**prot-a-724**	acute RITs	Cathepsin H	MR Egger	17.4365	26	0.8953
**prot-a-728**	acute RITs	Cathepsin L2	MR Egger	17.7541	17	0.4045
**prot-a-368**	acute RITs	Caveolin-2	MR Egger	18.9659	14	0.1663
**prot-a-398**	acute RITs	C-C motif chemokine 22	MR Egger	14.3590	20	0.8118
**prot-a-409**	acute RITs	C-C motif chemokine 5	MR Egger	23.6220	24	0.4834
**ebi-a-GCST90001449**	acute RITs	CD11c+ monocyte %monocyte	MR Egger	13.0289	13	0.4456
**ebi-a-GCST90001748**	acute RITs	CD20 on IgD+ CD38- B cell	MR Egger	15.6426	19	0.6810
**ebi-a-GCST90001751**	acute RITs	CD20 on IgD+ CD38+ B cell	MR Egger	10.3453	19	0.9439
**ebi-a-GCST90001760**	acute RITs	CD20 on unswitched memory B cell	MR Egger	21.4455	14	0.0907
**ebi-a-GCST90001760**	acute RITs	CD20 on unswitched memory B cell	MR Egger	21.4455	14	0.0907
**ebi-a-GCST90001910**	acute RITs	CD45 on B cell	MR Egger	8.6191	14	0.8546
**prot-a-3080**	acute RITs	Cellular tumor antigen p53	MR Egger	29.4795	17	0.0304
**prot-a-432**	acute RITs	CMRF35-like molecule 8	MR Egger	14.3205	23	0.9173
**prot-a-303**	acute RITs	Complement C1q tumor necrosis factor-related protein 1	MR Egger	13.8001	18	0.7420
**prot-a-303**	acute RITs	Complement C1q tumor necrosis factor-related protein 1	MR Egger	13.8001	18	0.7420
**prot-a-300**	acute RITs	Complement component 1 Q subcomponent-binding protein, mitochondrial	MR Egger	27.3953	26	0.3888
**ebi-a-GCST90019439**	acute RITs	Complement factor B measurement	MR Egger	20.8139	26	0.7515
**bbj-a-14**	acute RITs	C-reactive protein	MR Egger	40.6548	44	0.6158
**ieu-a-1015**	acute RITs	C-reactive protein	MR Egger	17.4109	10	0.0658
**prot-a-670**	acute RITs	C-reactive protein	MR Egger	16.5111	19	0.6230
**ieu-b-35**	acute RITs	C-Reactive protein level	MR Egger	127.4712	128	0.4966
**ebi-a-GCST90025959**	acute RITs	C-reactive protein levels	MR Egger	369.3230	351	0.2404
**prot-a-579**	acute RITs	C-type lectin domain family 5 member A	MR Egger	14.7115	18	0.6817
**ebi-a-GCST90002012**	acute RITs	CX3CR1 on CD14- CD16+ monocyte	MR Egger	20.2197	17	0.2631
**ebi-a-GCST90001997**	acute RITs	CX3CR1 on CD14+ CD16- monocyte	MR Egger	19.4771	18	0.3630
**ebi-a-GCST90001995**	acute RITs	CX3CR1 on monocyte	MR Egger	24.6545	18	0.1347

**prot-a-3175**	acute RITs	Cytochrome b-c1 complex subunit 7	MR Egger	20.5149	16	0.1979
**prot-a-1086**	acute RITs	D-dimer	MR Egger	18.9999	14	0.1650
**prot-a-813**	acute RITs	Desert hedgehog protein N-product	MR Egger	6.8027	12	0.8704
**prot-a-824**	acute RITs	Dickkopf-like protein 1	MR Egger	17.0942	14	0.2512
**prot-a-586**	acute RITs	Dual specificity protein kinase CLK2	MR Egger	20.2132	17	0.2635
**prot-a-2179**	acute RITs	E3 ubiquitin-protein ligase parkin	MR Egger	26.9373	18	0.0802
**prot-a-2528**	acute RITs	E3 ubiquitin-protein ligase rififylin	MR Egger	20.3184	11	0.0412
**prot-a-3278**	acute RITs	E3 ubiquitin-protein ligase ZNRF3	MR Egger	12.8557	10	0.2319
**prot-a-984**	acute RITs	Endoplasmic reticulum resident protein 29	MR Egger	11.5365	13	0.5660
**prot-a-902**	acute RITs	Ephrin-B1	MR Egger	12.9819	13	0.4492
**prot-a-907**	acute RITs	Ephrin-B3	MR Egger	15.4364	11	0.1634
**prot-a-2071**	acute RITs	Epididymal secretory protein E1	MR Egger	35.4736	24	0.0617
**prot-a-1835**	acute RITs	Epididymis-specific alpha-mannosidase	MR Egger	10.2115	15	0.8062
**prot-a-977**	acute RITs	Epiregulin	MR Egger	19.8661	18	0.3404
**prot-a-308**	acute RITs	ES1 protein homolog, mitochondrial	MR Egger	22.4212	22	0.4350
**prot-a-2892**	acute RITs	Estrogen sulfotransferase	MR Egger	23.4029	19	0.2201
**prot-a-1001**	acute RITs	Exosome complex component CSL4	MR Egger	22.3648	21	0.3788
**prot-a-1003**	acute RITs	Exostosin-like 2	MR Egger	10.7529	15	0.7699
**prot-a-1019**	acute RITs	Fas apoptotic inhibitory molecule 3	MR Egger	25.5470	26	0.4882
**prot-a-1102**	acute RITs	Fibroblast growth factor receptor 2	MR Egger	21.3769	23	0.5581
**prot-a-1117**	acute RITs	Filamin-A	MR Egger	19.4266	14	0.1493
**prot-a-567**	acute RITs	Galectin-10	MR Egger	9.8317	21	0.9810
**prot-a-1295**	acute RITs	Glucoside xylosyltransferase 1	MR Egger	16.5622	21	0.7373
**prot-a-1285**	acute RITs	Glutathione S-transferase A4	MR Egger	32.0086	24	0.1268
**ebi-a-GCST90002248**	acute RITs	Glycated hemoglobin levels	MR Egger	30.0777	22	0.1166
**prot-a-1046**	acute RITs	Glycosaminoglycan xylosylkinase	MR Egger	22.4670	24	0.5514
**prot-a-1277**	acute RITs	Granulins	MR Egger	23.4833	24	0.4914
**prot-a-1268**	acute RITs	GRB2-related adapter protein	MR Egger	4.8974	9	0.8432
**prot-a-747**	acute RITs	Gro-beta/gamma	MR Egger	5.8370	14	0.9704
**prot-b-55**	acute RITs	growth differentiation factor 15	MR Egger	8.0856	13	0.8380
**prot-a-1272**	acute RITs	Growth factor receptor-bound protein 7	MR Egger	13.1289	19	0.8319
**prot-a-1196**	acute RITs	Growth/differentiation factor 5	MR Egger	25.9864	18	0.1001
**prot-a-1197**	acute RITs	Growth/differentiation factor 9	MR Egger	15.1331	17	0.5859
**prot-a-1199**	acute RITs	GTP-binding protein GEM	MR Egger	12.9299	18	0.7957
**prot-a-1178**	acute RITs	Guanylate-binding protein 6	MR Egger	28.6298	17	0.0381
**prot-a-1387**	acute RITs	Heat shock 70 kDa protein 1-like	MR Egger	5.9420	10	0.8201

**prot-a-1310**	acute RITs	Hepatitis A virus cellular receptor 1	MR Egger	13.8579	13	0.3839
**prot-a-1366**	acute RITs	Heterogeneous nuclear ribonucleoprotein K	MR Egger	25.9969	21	0.2066
**prot-a-2224**	acute RITs	High affinity cGMP-specific 3',5'-cyclic phosphodiesterase 9A	MR Egger	12.4163	21	0.9277
**prot-a-2121**	acute RITs	High affinity nerve growth factor receptor	MR Egger	11.6470	10	0.3094
**prot-a-1338**	acute RITs	Histidine triad nucleotide-binding protein 1	MR Egger	14.6382	14	0.4033
**prot-a-1607**	acute RITs	Histone acetyltransferase KAT6A	MR Egger	8.5634	11	0.6621
**prot-a-1304**	acute RITs	Histone H2A.z	MR Egger	12.9408	13	0.4524
**ebi-a-GCST90002116**	acute RITs	HLA DR on B cell	MR Egger	16.4945	12	0.1696
**prot-a-1393**	acute RITs	Hyaluronidase-1	MR Egger	30.1789	20	0.0670
**prot-a-1071**	acute RITs	Immunoglobulin alpha Fc receptor	MR Egger	6.2777	6	0.3928
**prot-a-1576**	acute RITs	Immunoglobulin superfamily containing leucine-rich repeat protein 2	MR Egger	12.5864	15	0.6342
**prot-a-2141**	acute RITs	Inositol polyphosphate 5-phosphatase OCRL-1	MR Egger	17.7098	14	0.2203
**prot-a-1593**	acute RITs	Inositol-trisphosphate 3-kinase A	MR Egger	4.0298	9	0.9094
**prot-a-1435**	acute RITs	Interferon lambda-1	MR Egger	20.7357	12	0.0544
**prot-a-1547**	acute RITs	Interleukin enhancer-binding factor 3	MR Egger	12.7634	12	0.3865
**ebi-a-GCST004444**	acute RITs	Interleukin-10 levels	MR Egger	13.3341	13	0.4223
**prot-a-1479**	acute RITs	Interleukin-16	MR Egger	20.7648	16	0.1877
**ebi-a-GCST004448**	acute RITs	Interleukin-1-beta levels	MR Egger	5.0298	3	0.1696
**prot-a-1517**	acute RITs	Interleukin-27 receptor subunit alpha	MR Egger	11.1417	12	0.5168
**prot-a-1524**	acute RITs	Interleukin-34	MR Egger	33.6091	22	0.0538
**prot-a-1528**	acute RITs	Interleukin-36 gamma	MR Egger	12.3299	18	0.8297
**prot-a-1533**	acute RITs	Interleukin-4 receptor subunit alpha	MR Egger	16.7544	17	0.4711
**prot-a-1535**	acute RITs	Interleukin-5	MR Egger	16.9921	14	0.2566
**prot-a-1544**	acute RITs	Interleukin-7 receptor subunit alpha	MR Egger	26.4890	24	0.3288
**prot-a-1595**	acute RITs	Intersectin-1	MR Egger	23.3447	19	0.2225
**prot-a-81**	acute RITs	Intestinal-type alkaline phosphatase	MR Egger	14.5913	14	0.4067
**ebi-a-GCST90012012**	acute RITs	kallikrein-11 levels	MR Egger	23.9840	24	0.4625
**prot-a-1664**	acute RITs	Kallikrein-5	MR Egger	9.1997	10	0.5133
**prot-a-1640**	acute RITs	Killer cell immunoglobulin-like receptor 2DL2	MR Egger	15.0282	15	0.4494
**prot-a-1635**	acute RITs	Kinesin-like protein KIF16B	MR Egger	26.5063	23	0.2776
**prot-b-20**	acute RITs	KIT ligand	MR Egger	10.8861	10	0.3665
**prot-a-2825**	acute RITs	Kunitz-type protease inhibitor 3	MR Egger	34.9208	23	0.0530
**prot-a-2825**	acute RITs	Kunitz-type protease inhibitor 3	MR Egger	34.9208	23	0.0530
**prot-a-1714**	acute RITs	Lactase-like protein	MR Egger	17.0916	11	0.1052
**prot-a-1797**	acute RITs	Leucine-rich repeat transmembrane neuronal protein 4	MR Egger	13.2826	17	0.7171
**prot-a-1076**	acute RITs	Low affinity immunoglobulin gamma Fc region receptor III-B	MR Egger	22.2120	22	0.4473

**prot-a-2666**	acute RITs	L-Selectin	MR Egger	12.1245	20	0.9117
**prot-a-2666**	acute RITs	L-Selectin	MR Egger	12.1245	20	0.9117
**prot-a-1735**	acute RITs	Lutropin subunit beta	MR Egger	21.6425	18	0.2482
**prot-a-454**	acute RITs	Lymphocyte function-associated antigen 3	MR Egger	11.7311	9	0.2289
**prot-a-3236**	acute RITs	Lymphotactin	MR Egger	9.9917	13	0.6946
**prot-a-1912**	acute RITs	Macrophage metalloelastase	MR Egger	20.2028	14	0.1239
**prot-a-1931**	acute RITs	MAGUK p55 subfamily member 6	MR Egger	11.6626	15	0.7044
**prot-a-1854**	acute RITs	MAP kinase-activated protein kinase 5	MR Egger	27.8767	21	0.1437
**prot-a-1916**	acute RITs	Matrix metalloproteinase-16	MR Egger	10.6347	15	0.7780
**prot-a-1896**	acute RITs	Melanoma-derived growth regulatory protein	MR Egger	11.3601	15	0.7267
**prot-a-1908**	acute RITs	Membrane metallo-endopeptidase-like 1	MR Egger	19.6668	26	0.8071
**ebi-a-GCST90006924**	acute RITs	Merkel cell polyomavirus VP1 antibody levels	MR Egger	13.6464	13	0.3992
**prot-a-1052**	acute RITs	MIP18 family protein FAM96A	MR Egger	12.9799	12	0.3705
**prot-a-5**	acute RITs	Monoacylglycerol lipase ABHD12	MR Egger	20.7421	16	0.1886
**prot-b-75**	acute RITs	mucin 16, cell surface associated	MR Egger	13.9397	10	0.1758
**prot-b-75**	acute RITs	mucin 16, cell surface associated	MR Egger	13.9397	10	0.1758
**prot-a-1967**	acute RITs	Mucin-1	MR Egger	18.3683	20	0.5632
**prot-a-1995**	acute RITs	N-acetyl-D-glucosamine kinase	MR Egger	18.0411	15	0.2605
**prot-a-2057**	acute RITs	NACHT, LRR and PYD domains-containing protein 4	MR Egger	23.0133	16	0.1134
**prot-a-2026**	acute RITs	NADH dehydrogenase [ubiquinone] flavoprotein 2, mitochondrial	MR Egger	4.0299	10	0.9460
**prot-a-2081**	acute RITs	Neuronal pentraxin-2	MR Egger	15.8523	15	0.3919
**prot-a-345**	acute RITs	Neuron-specific vesicular protein calcyon	MR Egger	16.8495	16	0.3954
**prot-a-2099**	acute RITs	Neuropilin-1	MR Egger	17.4618	23	0.7860
**prot-a-2050**	acute RITs	Nidogen-2	MR Egger	9.3410	12	0.6736
**prot-a-1674**	acute RITs	NKG2-D type II integral membrane protein	MR Egger	17.3318	18	0.5004
**prot-a-1872**	acute RITs	Nuclear protein MDM1	MR Egger	22.8331	15	0.0877
**prot-a-2086**	acute RITs	Nuclear receptor subfamily 1 group D member 2	MR Egger	19.4588	19	0.4278
**prot-a-2093**	acute RITs	Nuclear receptor-binding protein	MR Egger	21.5889	17	0.2010
**prot-a-2150**	acute RITs	Oligophrenin-1	MR Egger	14.2786	18	0.7108
**prot-a-2155**	acute RITs	Oncostatin-M	MR Egger	11.2612	20	0.9392
**prot-a-2272**	acute RITs	Paired immunoglobulin-like type 2 receptor alpha	MR Egger	13.1362	19	0.8315
**prot-a-2344**	acute RITs	Peptidyl-prolyl cis-trans isomerase-like 2	MR Egger	28.3108	19	0.0776
**prot-a-2425**	acute RITs	Phosphatidylinositol 3,4,5-trisphosphate 3-phosphatase and dual-specificity protein phosphatase PTEN	MR Egger	21.1516	20	0.3883
**prot-a-2294**	acute RITs	Phospholipase B-like 1	MR Egger	8.4344	14	0.8655
**prot-a-2698**	acute RITs	Pigment epithelium-derived factor	MR Egger	11.8713	17	0.8079
**prot-a-2299**	acute RITs	Pleckstrin homology domain-containing family A member 1	MR Egger	24.3720	20	0.2265

**prot-a-1171**	acute RITs	Polypeptide N-acetylgalactosaminyltransferase 2	MR Egger	24.4302	18	0.1414
**prot-a-1613**	acute RITs	Potassium voltage-gated channel subfamily E regulatory beta subunit 5	MR Egger	11.1618	14	0.6733
**prot-a-1615**	acute RITs	Potassium voltage-gated channel subfamily G member 4	MR Egger	22.6642	19	0.2525
**prot-a-2209**	acute RITs	Procollagen C-endopeptidase enhancer 1	MR Egger	15.7999	18	0.6065
**prot-a-2409**	acute RITs	Proteasome subunit alpha type-1	MR Egger	19.8851	15	0.1764
**prot-a-827**	acute RITs	Protein delta homolog 1	MR Egger	14.9014	17	0.6026
**prot-a-810**	acute RITs	Protein DGCR6	MR Egger	17.3577	19	0.5656
**prot-a-2232**	acute RITs	Protein disulfide-isomerase A3	MR Egger	17.8411	21	0.6590
**prot-a-2233**	acute RITs	Protein disulfide-isomerase A5	MR Egger	36.3343	24	0.0509
**prot-a-1027**	acute RITs	Protein FAM163A	MR Egger	29.1901	21	0.1095
**prot-a-1049**	acute RITs	Protein FAM3B	MR Egger	15.2434	10	0.1234
**prot-a-2619**	acute RITs	Protein S100-A5	MR Egger	24.9758	22	0.2982
**prot-a-3093**	acute RITs	Protein-tyrosine sulfotransferase 2	MR Egger	31.5890	24	0.1375
**prot-a-3093**	acute RITs	Protein-tyrosine sulfotransferase 2	MR Egger	31.5890	24	0.1375
**prot-a-2203**	acute RITs	Protocadherin beta-1	MR Egger	14.0309	16	0.5964
**prot-a-2207**	acute RITs	Protocadherin gamma-C5	MR Egger	19.5632	19	0.4213
**prot-a-2191**	acute RITs	Pterin-4-alpha-carbinolamine dehydratase	MR Egger	12.3551	15	0.6520
**prot-a-2711**	acute RITs	Pulmonary surfactant-associated protein C	MR Egger	25.4214	23	0.3289
**prot-a-2477**	acute RITs	Ras-related protein Rab-39B	MR Egger	28.0787	18	0.0609
**prot-a-2444**	acute RITs	Receptor-type tyrosine-protein phosphatase H	MR Egger	9.1460	16	0.9073
**prot-a-2524**	acute RITs	Resistin	MR Egger	24.9242	19	0.1631
**prot-a-2497**	acute RITs	Retinoblastoma-binding protein 5	MR Egger	10.7267	12	0.5525
**prot-a-2602**	acute RITs	Ribonucleoside-diphosphate reductase subunit M2 B	MR Egger	17.9169	13	0.1607
**prot-a-1945**	acute RITs	Ribosome-recycling factor, mitochondrial	MR Egger	17.4362	19	0.5603
**prot-a-2584**	acute RITs	Ribulose-phosphate 3-epimerase	MR Egger	12.5120	10	0.2523
**prot-a-2569**	acute RITs	RING finger protein 215	MR Egger	17.5015	17	0.4209
**prot-a-2575**	acute RITs	rRNA methyltransferase 3, mitochondrial	MR Egger	17.7704	18	0.4709
**prot-a-2641**	acute RITs	Scavenger receptor class F member 2	MR Egger	20.5740	19	0.3608
**prot-a-2642**	acute RITs	Scavenger receptor class F member 2	MR Egger	14.0834	17	0.6612
**prot-a-2709**	acute RITs	Secreted frizzled-related protein 1	MR Egger	13.8600	19	0.7918
**prot-a-2669**	acute RITs	Semaphorin-3A	MR Egger	16.9595	23	0.8112
**prot-a-2671**	acute RITs	Semaphorin-3C	MR Egger	8.9164	10	0.5401
**prot-a-2838**	acute RITs	Serine/arginine-rich splicing factor 6	MR Egger	10.7555	10	0.3769
**prot-a-2170**	acute RITs	Serine/threonine-protein kinase PAK 4	MR Egger	10.4493	11	0.4905
**prot-a-2690**	acute RITs	Serpin A12	MR Egger	45.1903	24	0.0055
**prot-a-2730**	acute RITs	Sialic acid-binding Ig-like lectin 8	MR Egger	23.1612	21	0.3354

**prot-a-2655**	acute RITs	Signal peptide, CUB and EGF-like domain-containing protein 1	MR Egger	21.5462	13	0.0628
**prot-a-2775**	acute RITs	Single-pass membrane and coiled-coil domain-containing protein 2	MR Egger	16.4598	14	0.2861
**prot-a-2714**	acute RITs	Small glutamine-rich tetratricopeptide repeat-containing protein alpha	MR Egger	14.2228	18	0.7145
**prot-a-2763**	acute RITs	Sodium- and chloride-dependent glycine transporter 1	MR Egger	12.0151	11	0.3625
**prot-a-200**	acute RITs	Sodium/potassium-transporting ATPase subunit beta-2	MR Egger	15.7664	17	0.5404
**prot-a-352**	acute RITs	Soluble calcium-activated nucleotidase 1	MR Egger	19.0121	15	0.2132
**prot-a-2795**	acute RITs	Sorting nexin-7	MR Egger	19.4470	22	0.6176
**prot-a-2804**	acute RITs	Sperm acrosome membrane-associated protein 3	MR Egger	20.3281	17	0.2578
**prot-b-68**	acute RITs	spondin 1	MR Egger	2.3981	2	0.3015
**ebi-a-GCST90002074**	acute RITs	SSC-A on CD14+ monocyte	MR Egger	14.9470	17	0.5993
**ebi-a-GCST90002081**	acute RITs	SSC-A on CD4+ T cell	MR Egger	22.7171	18	0.2017
**ebi-a-GCST90002079**	acute RITs	SSC-A on lymphocyte	MR Egger	12.3689	15	0.6509
**ebi-a-GCST90019393**	acute RITs	Stromal cell-derived factor 2 measurement	MR Egger	16.9295	13	0.2025
**prot-a-2468**	acute RITs	Sulfhydryl oxidase 1	MR Egger	12.0768	14	0.6001
**prot-a-2894**	acute RITs	Sulfotransferase 4A1	MR Egger	21.7505	18	0.2433
**prot-a-2901**	acute RITs	Sushi domain-containing protein 1	MR Egger	15.0327	20	0.7745
**prot-a-2885**	acute RITs	Syntaxin-1A	MR Egger	19.2578	19	0.4404
**prot-a-2933**	acute RITs	TATA-box-binding protein	MR Egger	25.3594	17	0.0869
**prot-a-3068**	acute RITs	Tenascin-R	MR Egger	13.6423	20	0.8482
**prot-a-3084**	acute RITs	Thiamin pyrophosphokinase 1	MR Egger	15.0966	15	0.4445
**prot-a-3102**	acute RITs	Thyrotropin-releasing hormone	MR Egger	27.3671	21	0.1590
**prot-a-3076**	acute RITs	Torsin-1A-interacting protein 1	MR Egger	20.7310	17	0.2386
**prot-a-3115**	acute RITs	TPA-induced transmembrane protein	MR Egger	9.9254	10	0.4471
**prot-a-2963**	acute RITs	Transforming growth factor beta-1-induced transcript 1 protein	MR Egger	25.1920	18	0.1197
**prot-a-918**	acute RITs	Translation initiation factor eIF-2B subunit alpha	MR Egger	14.0787	11	0.2287
**prot-a-2844**	acute RITs	Translocon-associated protein subunit alpha	MR Egger	13.5616	12	0.3296
**prot-a-2992**	acute RITs	Transmembrane and coiled-coil domain-containing protein 5A	MR Egger	16.1171	27	0.9507
**prot-a-2993**	acute RITs	Transmembrane emp24 domain-containing protein 10	MR Egger	7.8867	14	0.8951
**prot-a-285**	acute RITs	Transmembrane protein C16orf54	MR Egger	18.5662	15	0.2341
**prot-a-1883**	acute RITs	tRNA (guanine-N(7)-)-methyltransferase	MR Egger	22.7817	15	0.0889
**prot-a-3029**	acute RITs	Tumor necrosis factor	MR Egger	11.2181	14	0.6688
**prot-a-3059**	acute RITs	Tumor necrosis factor ligand superfamily member 15	MR Egger	19.0716	20	0.5172
**prot-a-3128**	acute RITs	TYMS opposite strand protein	MR Egger	25.5311	17	0.0834
**prot-a-3129**	acute RITs	Tyrosine-protein kinase receptor TYRO3	MR Egger	15.7539	19	0.6736
**prot-a-2582**	acute RITs	Tyrosine-protein kinase transmembrane receptor ROR2	MR Egger	21.8201	24	0.5900
**prot-a-598**	acute RITs	UMP-CMP kinase	MR Egger	23.7727	16	0.0946

**prot-a-295**	acute RITs	Uncharacterized protein C1orf115	MR Egger	22.2732	23	0.5038
**prot-a-311**	acute RITs	Uncharacterized protein C2orf66	MR Egger	16.9315	16	0.3900
**prot-a-3155**	acute RITs	UTP--glucose-1-phosphate uridylyltransferase	MR Egger	12.4798	11	0.3287
**prot-a-3197**	acute RITs	Vascular endothelial growth factor A, isoform 121	MR Egger	7.3596	17	0.9784
**prot-a-3197**	acute RITs	Vascular endothelial growth factor A, isoform 121	MR Egger	7.3596	17	0.9784
**ebi-a-GCST004422**	acute RITs	Vascular endothelial growth factor levels	MR Egger	14.4331	14	0.4180
**ebi-a-GCST90011995**	acute RITs	Vascular endothelial growth factor levels	MR Egger	10.8427	15	0.7637
**ebi-a-GCST90011995**	acute RITs	Vascular endothelial growth factor levels	MR Egger	10.8427	15	0.7637
**ebi-a-GCST90011995**	acute RITs	Vascular endothelial growth factor levels	MR Egger	10.8427	15	0.7637
**prot-a-3212**	acute RITs	Vesicle transport through interaction with t-SNAREs homolog 1A	MR Egger	15.3234	18	0.6397
**prot-a-1179**	acute RITs	Vitamin D-binding protein	MR Egger	19.1904	15	0.2052
**prot-a-3230**	acute RITs	Wnt inhibitory factor 1	MR Egger	16.2734	15	0.3641
**prot-a-3231**	acute RITs	WNT1-inducible-signaling pathway protein 1	MR Egger	12.5816	15	0.6346
**prot-a-3276**	acute RITs	Zinc finger protein 774	MR Egger	21.3909	25	0.6706
**prot-a-208**	acute RITs	Zinc-alpha-2-glycoprotein	MR Egger	12.5341	14	0.5635
**ukb-a-406**	acute RITs	Current employment status: Looking after home and/or family	MR Egger	7.1092	15	0.9545
**ukb-d-22601_41123205**	acute RITs	Job coding: civil service administrative officer or assistant or clerk, tax collector, prison service clerk, law courts clerk	MR Egger	21.5350	24	0.6070
**ukb-d-22601_35393271**	acute RITs	Job coding: management information officer, conference/events co-ordinator/organiser, exhibition officer, work study engineer/officer/analyst, contract adviser/agent, election agent, business system analyst	MR Egger	14.3838	20	0.8105
**ukb-d-22601_23193405**	acute RITs	Job coding: other teaching professional including private tutor, tefl (teaching english as a foreign language) teacher; tutor at adult education centre, etc.	MR Egger	17.2236	18	0.5078
**ukb-d-22601_23213026**	acute RITs	Job coding: scientific researcher, scientific officer, medical research associate, experimental officer	MR Egger	20.3373	21	0.5000
**ukb-d-22601_23163403**	acute RITs	Job coding: special needs teaching professional (including head teacher)	MR Egger	27.0209	17	0.0578
**ukb-e-826_AFR**	acute RITs	Job involves shift work	MR Egger	10.8408	13	0.6241
**ukb-d-22617_1151**	acute RITs	Job SOC coding: Financial institution managers	MR Egger	25.9140	19	0.1326
**ukb-d-22617_2321**	acute RITs	Job SOC coding: Scientific researchers	MR Egger	30.9385	24	0.1556
**ukb-d-22617_3231**	acute RITs	Job SOC coding: Youth and community workers	MR Egger	27.1554	22	0.2054
**ukb-b-18099**	acute RITs	Qualifications: O levels/GCSEs or equivalent	MR Egger	151.2988	132	0.1200
**ukb-a-368**	acute RITs	Work/job satisfaction	MR Egger	17.1804	22	0.7533
**ebi-a-GCST90013919**	acute RITs	Cholesterol lowering medication use (UKB data field 6177_1) (Firth correction)	MR Egger	124.2182	120	0.3775
**ebi-a-GCST90013969**	acute RITs	Cholesterol lowering medication use (UKB data field 6177_1) (SPA correction)	MR Egger	120.2129	119	0.4516
**ukb-a-488**	acute RITs	Medication for cholesterol blood pressure or diabetes: Cholesterol lowering medication	MR Egger	115.1620	92	0.0515
**ukb-b-11740**	acute RITs	Medication for cholesterol, blood pressure or diabetes: Cholesterol lowering medication	MR Egger	142.8262	127	0.1596
**ukb-e-6153_p2_AFR**	acute RITs	Medication for cholesterol, blood pressure, diabetes, or take exogenous hormones	MR Egger	25.4026	19	0.1477
**ebi-a-GCST90018984**	acute RITs	Medication use (antihypertensives)	MR Egger	65.5009	51	0.0833
**ebi-a-GCST90018767**	acute RITs	Medication use (calcium channel blockers)	MR Egger	85.5253	66	0.0534

**ebi-a-GCST90018985**	acute RITs	Medication use (diuretics)	MR Egger	200.8694	205	0.5684
**ebi-a-GCST90018993**	acute RITs	Medication use (drugs affecting bone structure and mineralization)	MR Egger	61.8000	57	0.3087
**ukb-b-12753**	acute RITs	Recent medication for hayfever or allergic rhinitis	MR Egger	20.0930	15	0.1684
**ukb-b-15918**	acute RITs	Treatment speciality of consultant (recoded): Dermatology	MR Egger	33.3506	28	0.2231
**ukb-b-3255**	acute RITs	Treatment speciality of consultant (recoded): Upper gastrointestinal surgery	MR Egger	4.4632	5	0.4848
**ukb-e-20003_p118_AFR**	acute RITs	Treatment/medication code	MR Egger	26.5559	18	0.0877
**ukb-b-9207**	acute RITs	Treatment/medication code: amlodipine	MR Egger	89.4062	87	0.4086
**ukb-b-11862**	acute RITs	Treatment/medication code: beconase 50micrograms nasal spray	MR Egger	4.9543	8	0.7624
**ukb-b-7385**	acute RITs	Treatment/medication code: codeine	MR Egger	5.3450	4	0.2537
**ukb-b-8668**	acute RITs	Treatment/medication code: doxazosin	MR Egger	37.3080	34	0.3195
**ukb-a-162**	acute RITs	Treatment/medication code: perindopril	MR Egger	25.2689	21	0.2357
**ukb-a-141**	acute RITs	Treatment/medication code: prednisolone	MR Egger	9.0058	13	0.7725
**ukb-b-11895**	acute RITs	Treatment/medication code: ramipril	MR Egger	45.5781	63	0.9518
**ukb-a-130**	acute RITs	Treatment/medication code: senna	MR Egger	19.0385	25	0.7953
**ukb-b-16956**	acute RITs	Treatment/medication code: thyroxine sodium	MR Egger	6.7921	9	0.6588
**ukb-a-120**	acute RITs	Treatment/medication code: tranexamic acid	MR Egger	17.9197	21	0.6541
**ukb-b-17616**	acute RITs	Treatment/medication code: xalatan 0.005% eye drops	MR Egger	5.2207	9	0.8147
**ukb-e-6155_p5_AFR**	acute RITs	Vitamin and mineral supplements	MR Egger	13.8846	10	0.1783
**ukb-e-6155_p8_AFR**	acute RITs	Vitamin and mineral supplements	MR Egger	12.3538	14	0.5779
**ukb-a-464**	acute RITs	Vitamin and mineral supplements: Multivitamins +/- minerals	MR Egger	48.4845	45	0.3343
**ukb-b-19550**	acute RITs	Vitamin and/or mineral supplement use: Calcium	MR Egger	3.3034	4	0.5084
**ukb-b-18593**	acute RITs	Vitamin D	MR Egger	18.0256	20	0.5857
**ukb-e-Z51_AFR**	acute RITs	Z51 Other medical care	MR Egger	10.5295	13	0.6502
**met-c-846**	acute RITs	3-hydroxybutyrate	MR Egger	19.3353	15	0.1989
**met-d-bOHbutyrate**	acute RITs	3-Hydroxybutyrate	MR Egger	52.0645	42	0.1373
**met-a-311**	acute RITs	3-hydroxybutyrate (BHBA)	MR Egger	10.2107	7	0.1769
**met-a-365**	acute RITs	3-methoxytyrosine	MR Egger	18.3732	14	0.1903
**ieu-a-1**	acute RITs	Adiponectin	MR Egger	22.0373	30	0.8527
**met-a-537**	acute RITs	Adrenate (22:4n6)	MR Egger	13.2748	9	0.1506
**bbj-a-9**	acute RITs	Albumin	MR Egger	29.1847	46	0.9748
**met-a-616**	acute RITs	Alpha-hydroxyisovalerate	MR Egger	9.9355	12	0.6216
**met-d-ApoA1**	acute RITs	Apolipoprotein A1	MR Egger	140.8373	139	0.4405
**ieu-b-107**	acute RITs	apolipoprotein A-I	MR Egger	449.1411	467	0.7157
**met-c-842**	acute RITs	Apolipoprotein A-I	MR Egger	31.2163	28	0.3076
**ieu-b-108**	acute RITs	apolipoprotein B	MR Egger	297.6148	300	0.5281
**ieu-b-108**	acute RITs	apolipoprotein B	MR Egger	297.6148	300	0.5281

**met-d-ApoB**	acute RITs	Apolipoprotein B	MR Egger	101.8768	106	0.5952
**ebi-a-GCST90092809**	acute RITs	Apolipoprotein B levels	MR Egger	102.0968	107	0.6159
**prot-a-134**	acute RITs	Apolipoprotein L1	MR Egger	20.6306	19	0.3576
**met-a-638**	acute RITs	Asparagine	MR Egger	38.7805	37	0.3893
**ieu-b-4869**	acute RITs	Bioavailable Testosterone	MR Egger	223.3301	229	0.5933
**ebi-a-GCST90012102**	acute RITs	Bioavailable testosterone levels	MR Egger	266.0651	264	0.4528
**ebi-a-GCST90012104**	acute RITs	Bioavailable testosterone levels	MR Egger	230.6376	222	0.3313
**ebi-a-GCST90012104**	acute RITs	Bioavailable testosterone levels	MR Egger	230.6376	222	0.3313
**bbj-a-10**	acute RITs	Blood sugar	MR Egger	38.2642	42	0.6357
**ebi-a-GCST90018948**	acute RITs	Blood urea nitrogen levels	MR Egger	371.0350	355	0.2683
**ebi-a-GCST90014002**	acute RITs	C reactive protein levels (UKB data field 30710)	MR Egger	389.9967	390	0.4905
**met-a-481**	acute RITs	Caprylate (8:0)	MR Egger	29.6037	40	0.8860
**met-c-887**	acute RITs	Cholesterol esters in large VLDL	MR Egger	28.1059	29	0.5123
**met-c-899**	acute RITs	Cholesterol esters in medium HDL	MR Egger	10.2574	19	0.9463
**met-c-899**	acute RITs	Cholesterol esters in medium HDL	MR Egger	10.2574	19	0.9463
**met-d-M_HDL_C**	acute RITs	Cholesterol in medium HDL	MR Egger	165.1999	150	0.1873
**met-d-M_VLDL_C**	acute RITs	Cholesterol in medium VLDL	MR Egger	118.3159	112	0.3232
**met-d-S_LDL_C**	acute RITs	Cholesterol in small LDL	MR Egger	103.2440	99	0.3652
**ebi-a-GCST90014000**	acute RITs	Cholesterol levels (UKB data field 30690)	MR Egger	240.4932	277	0.9448
**ebi-a-GCST90092956**	acute RITs	Cholesterol levels in small LDL	MR Egger	83.7880	95	0.7878
**met-d-M_HDL_C_pct**	acute RITs	Cholesterol to total lipids ratio in medium HDL	MR Egger	143.3572	166	0.8974
**ebi-a-GCST90092918**	acute RITs	Cholesteryl ester levels in medium VLDL	MR Egger	90.9270	94	0.5706
**ebi-a-GCST90060634**	acute RITs	Cholesteryl ester(18:3)_[M+NH4]1+ levels	MR Egger	19.2451	18	0.3769
**met-d-HDL_CE**	acute RITs	Cholesteryl esters in HDL	MR Egger	169.5808	180	0.7001
**met-d-L_LDL_CE**	acute RITs	Cholesteryl esters in large LDL	MR Egger	86.9036	100	0.8218
**met-d-LDL_CE**	acute RITs	Cholesteryl esters in LDL	MR Egger	85.5298	96	0.7693
**met-d-M_HDL_CE**	acute RITs	Cholesteryl esters in medium HDL	MR Egger	167.2439	152	0.1882
**met-d-M_VLDL_CE**	acute RITs	Cholesteryl esters in medium VLDL	MR Egger	100.8855	99	0.4284
**met-d-S_HDL_CE**	acute RITs	Cholesteryl esters in small HDL	MR Egger	68.7391	102	0.9953
**met-d-IDL_CE_pct**	acute RITs	Cholesteryl esters to total lipids ratio in IDL	MR Egger	127.5938	128	0.4935
**met-d-L_HDL_CE_pct**	acute RITs	Cholesteryl esters to total lipids ratio in large HDL	MR Egger	157.0964	174	0.8163
**met-d-L_HDL_CE_pct**	acute RITs	Cholesteryl esters to total lipids ratio in large HDL	MR Egger	157.0964	174	0.8163
**met-d-M_HDL_CE_pct**	acute RITs	Cholesteryl esters to total lipids ratio in medium HDL	MR Egger	115.7557	161	0.9972
**ebi-a-GCST90007307**	acute RITs	circulating leptin levels	MR Egger	8.6353	13	0.7999
**ebi-a-GCST90007309**	acute RITs	circulating leptin levels	MR Egger	3.2076	5	0.6680

**ebi-a-GCST90007310**	acute RITs	circulating leptin levels	MR Egger	4.8173	7	0.6822
**ebi-a-GCST90007312**	acute RITs	circulating leptin levels	MR Egger	4.4116	5	0.4918
**ebi-a-GCST90007313**	acute RITs	circulating leptin levels	MR Egger	3.5250	4	0.4741
**ebi-a-GCST90007316**	acute RITs	circulating leptin levels	MR Egger	3.7730	4	0.4376
**ebi-a-GCST90007319**	acute RITs	circulating leptin levels adjusted for BMI	MR Egger	5.8073	8	0.6688
**ebi-a-GCST90007320**	acute RITs	circulating leptin levels adjusted for BMI	MR Egger	2.1501	4	0.7082
**ebi-a-GCST90007321**	acute RITs	circulating leptin levels adjusted for BMI	MR Egger	12.9351	7	0.0737
**ebi-a-GCST90007322**	acute RITs	circulating leptin levels adjusted for BMI	MR Egger	5.8361	6	0.4418
**ebi-a-GCST90007324**	acute RITs	circulating leptin levels adjusted for BMI	MR Egger	4.1705	6	0.6536
**ebi-a-GCST90007327**	acute RITs	circulating leptin levels adjusted for BMI	MR Egger	3.6569	4	0.4544
**met-d-Clinical_LDL_C**	acute RITs	Clinical LDL cholesterol	MR Egger	109.4176	98	0.2024
**met-d-HDL_P**	acute RITs	Concentration of HDL particles	MR Egger	138.2954	140	0.5249
**ebi-a-GCST90092887**	acute RITs	Concentration of LDL particles	MR Egger	94.9546	102	0.6766
**met-d-LDL_P**	acute RITs	Concentration of LDL particles	MR Egger	103.3334	108	0.6090
**met-d-M_HDL_P**	acute RITs	Concentration of medium HDL particles	MR Egger	154.2069	140	0.1945
**ebi-a-GCST90092963**	acute RITs	Concentration of small LDL particles	MR Egger	108.3613	111	0.5532
**met-d-S_LDL_P**	acute RITs	Concentration of small LDL particles	MR Egger	108.6623	112	0.5717
**met-d-XL_VLDL_P**	acute RITs	Concentration of very large VLDL particles	MR Egger	127.3143	144	0.8375
**met-a-744**	acute RITs	Cyclo(leu-pro)	MR Egger	12.5352	12	0.4037
**ebi-a-GCST90060606**	acute RITs	Diacylglycerol(34:2)_[M+H-H2O]1+ levels	MR Egger	15.3824	15	0.4242
**ebi-a-GCST90060606**	acute RITs	Diacylglycerol(34:2)_[M+H-H2O]1+ levels	MR Egger	15.3824	15	0.4242
**ebi-a-GCST90060625**	acute RITs	Diacylglycerol(36:2)_[M+NH4]1+ levels	MR Egger	15.0447	16	0.5214
**ebi-a-GCST90025954**	acute RITs	Direct low density lipoprotein cholesterol levels	MR Egger	284.3176	269	0.2492
**met-a-586**	acute RITs	Eicosenoate (20:1n9 or 11)	MR Egger	15.2259	10	0.1240
**ebi-a-GCST90019407**	acute RITs	Electron transfer flavoprotein subunit alpha, mitochondrial measurement	MR Egger	26.8327	22	0.2176
**ebi-a-GCST90012105**	acute RITs	Estradiol levels	MR Egger	51.3561	40	0.1076
**ebi-a-GCST000571**	acute RITs	Fasting blood insulin	MR Egger	1.8057	10	0.9976
**ebi-a-GCST90060570**	acute RITs	Fatty acid(15:0)_[M-H]1- levels	MR Egger	8.6010	10	0.5703
**ebi-a-GCST90060571**	acute RITs	Fatty acid(16:1)_[M-H]1- levels	MR Egger	8.1194	10	0.6172
**met-d-HDL_FC**	acute RITs	Free cholesterol in HDL	MR Egger	166.7121	167	0.4917
**met-d-IDL_FC**	acute RITs	Free cholesterol in IDL	MR Egger	102.6771	110	0.6772
**met-c-900**	acute RITs	Free cholesterol in medium HDL	MR Egger	16.0935	22	0.8112
**met-d-M_HDL_FC**	acute RITs	Free cholesterol in medium HDL	MR Egger	182.1389	150	0.0379
**met-d-M_LDL_FC**	acute RITs	Free cholesterol in medium LDL	MR Egger	107.0537	95	0.1873
**met-d-M_HDL_FC_pct**	acute RITs	Free cholesterol to total lipids ratio in medium HDL	MR Egger	153.8961	173	0.8488
**met-d-S_HDL_FC_pc**	acute RITs	Free cholesterol to total lipids ratio in small HDL	MR Egger	137.6310	123	0.1735

**t**						
**ebi-a-GCST90025966**	acute RITs	Gamma glutamyl transferase levels	MR Egger	517.6442	484	0.1404
**met-a-564**	acute RITs	Gamma-glutamylthreonine*	MR Egger	3.9774	7	0.7824
**ebi-a-GCST90018735**	acute RITs	Glucose levels	MR Egger	39.1558	45	0.7169
**ebi-a-GCST005058**	acute RITs	HDL cholesterol	MR Egger	14.5753	19	0.7492
**ebi-a-GCST90018736**	acute RITs	HDL cholesterol	MR Egger	102.0345	102	0.4804
**ebi-a-GCST90018956**	acute RITs	HDL cholesterol	MR Egger	480.7110	464	0.2864
**ieu-b-109**	acute RITs	HDL cholesterol	MR Egger	511.0267	524	0.6493
**ieu-b-4843**	acute RITs	HDL cholesterol	MR Egger	60.4851	65	0.6356
**ieu-b-4844**	acute RITs	HDL cholesterol	MR Egger	136.1812	151	0.8006
**met-d-HDL_C**	acute RITs	HDL cholesterol	MR Egger	170.1835	176	0.6094
**ukb-e-30760_AFR**	acute RITs	HDL cholesterol	MR Egger	27.0213	25	0.3548
**ebi-a-GCST90025956**	acute RITs	HDL cholesterol levels	MR Egger	468.0316	450	0.2692
**ebi-a-GCST90002309**	acute RITs	Hemoglobin concentration	MR Egger	35.9554	38	0.5643
**ebi-a-GCST008035**	acute RITs	High density lipoprotein cholesterol levels	MR Egger	80.2131	71	0.2126
**ebi-a-GCST90014007**	acute RITs	High density lipoprotein cholesterol levels (UKB data field 30760)	MR Egger	451.5486	456	0.5501
**bbj-a-24**	acute RITs	High-density-lipoprotein cholesterol	MR Egger	73.3051	83	0.7678
**ebi-a-GCST90014008**	acute RITs	IGF 1 (UKB data field 30770)	MR Egger	525.1976	530	0.5507
**ukb-e-30770_CSA**	acute RITs	IGF-1	MR Egger	36.4113	28	0.1324
**ukb-e-recode1_CSA**	acute RITs	Indirect bilirubin	MR Egger	8.3839	17	0.9576
**met-a-323**	acute RITs	Isoleucine	MR Egger	10.2913	16	0.8510
**met-a-652**	acute RITs	Isovalerylcarnitine	MR Egger	11.0474	17	0.8541
**ieu-b-110**	acute RITs	LDL cholesterol	MR Egger	268.4832	279	0.6637
**ieu-b-5089**	acute RITs	LDL cholesterol	MR Egger	166.5359	150	0.1686
**met-d-LDL_C**	acute RITs	LDL cholesterol	MR Egger	92.2208	93	0.5033
**ebi-a-GCST90025993**	acute RITs	Lipoprotein (a) levels	MR Egger	76.7932	68	0.2176
**ebi-a-GCST90002412**	acute RITs	Low density lipoprotein cholesterol levels	MR Egger	502.5563	479	0.2205
**ebi-a-GCST90019485**	acute RITs	MAP/microtubule affinity-regulating kinase 3 measurement	MR Egger	16.1328	23	0.8495
**ebi-a-GCST90019404**	acute RITs	Medium-chain specific acyl-CoA dehydrogenase, mitochondrial measurement	MR Egger	19.8617	19	0.4029
**ebi-a-GCST90012047**	acute RITs	Myoglobin levels	MR Egger	32.6653	36	0.6280
**met-a-477**	acute RITs	Myristoleate (14:1n5)	MR Egger	7.6087	11	0.7479
**met-a-576**	acute RITs	Palmitoleate (16:1n7)	MR Egger	5.3903	6	0.4948
**met-c-919**	acute RITs	Phenylalanine	MR Egger	14.4116	20	0.8090
**met-a-430**	acute RITs	Phenyllactate (PLA)	MR Egger	10.9062	16	0.8152
**ebi-a-GCST90014010**	acute RITs	Phosphate levels (UKB data field 30810)	MR Egger	299.2806	284	0.2553
**ebi-a-GCST90060687**	acute RITs	Phosphatidate(34:0)_[M+OAc]1- levels	MR Egger	6.3441	8	0.6087

**ebi-a-GCST90060914**	acute RITs	Phosphatidylcholine(38:7)_[M+OAc]1-/Phosphatidylserine(42:6)_[M-H]1- levels	MR Egger	11.8492	14	0.6184
**ebi-a-GCST90060671**	acute RITs	Phosphatidylcholine-O(32:0)_[M+H]1+/Phosphatidylethanolamine-O(35:0)_[M+H]1+ levels	MR Egger	17.3928	16	0.3606
**ebi-a-GCST90060801**	acute RITs	Phosphatidylcholine-O(38:4)_[M+H]1+/Phosphatidylcholine-P(38:3)_[M+H]1+ levels	MR Egger	12.3867	15	0.6496
**ebi-a-GCST90060961**	acute RITs	Phosphatidylserine(40:5)_[M+OAc]1- levels	MR Egger	19.0209	24	0.7509
**met-d-XL_VLDL_PL**	acute RITs	Phospholipids in very large VLDL	MR Egger	124.7331	138	0.7838
**met-d-L_VLDL_PL_pct**	acute RITs	Phospholipids to total lipids ratio in large VLDL	MR Egger	110.0952	143	0.9812
**met-d-M_HDL_PL_pct**	acute RITs	Phospholipids to total lipids ratio in medium HDL	MR Egger	171.3816	180	0.6651
**ebi-a-GCST90092977**	acute RITs	Phospholipids to total lipids ratio in small VLDL	MR Egger	151.1657	135	0.1618
**ieu-a-1012**	acute RITs	Plasma cortisol	MR Egger	4.3618	6	0.6278
**met-a-678**	acute RITs	Pro-hydroxy-pro	MR Egger	16.2216	16	0.4376
**met-a-574**	acute RITs	Pseudouridine	MR Egger	20.4857	24	0.6688
**met-a-501**	acute RITs	Pyroglutamine*	MR Egger	19.9377	16	0.2230
**met-d-SFA_pct**	acute RITs	Ratio of saturated fatty acids to total fatty acids	MR Egger	65.8806	75	0.7649
**met-d-TG_by_PG**	acute RITs	Ratio of triglycerides to phosphoglycerides	MR Egger	152.7670	171	0.8382
**met-d-Remnant_C**	acute RITs	Remnant cholesterol (non-HDL, non-LDL -cholesterol)	MR Egger	115.0468	112	0.4028
**ebi-a-GCST005059**	acute RITs	Serum albumin level	MR Egger	13.6606	16	0.6240
**ebi-a-GCST90018722**	acute RITs	Serum alkaline phosphatase levels	MR Egger	83.5949	102	0.9079
**ebi-a-GCST90025991**	acute RITs	Serum urea levels	MR Egger	351.9225	319	0.0990
**ebi-a-GCST90019384**	acute RITs	SPARC-related modular calcium-binding protein 1 measurement	MR Egger	36.6747	28	0.1262
**ebi-a-GCST90060683**	acute RITs	Sphingomyelin(32:1)_[M+OAc]1- levels	MR Egger	18.7727	18	0.4059
**ebi-a-GCST90060633**	acute RITs	Sphingomyelin(32:1)_[M-CH3]1- levels	MR Egger	15.3809	14	0.3526
**ebi-a-GCST90060643**	acute RITs	Sphingomyelin(34:2)_[M-CH3]1- levels	MR Egger	15.9377	9	0.0682
**ebi-a-GCST90060776**	acute RITs	Sphingomyelin(40:1)_[M+H]1+ levels	MR Egger	10.3673	15	0.7960
**ebi-a-GCST90060742**	acute RITs	Sphingomyelin(40:2)_[M-CH3]1- levels	MR Egger	13.9199	16	0.6047
**ebi-a-GCST90060814**	acute RITs	Sphingomyelin(41:0)_[M+H]1- levels	MR Egger	1.9764	8	0.9817
**ebi-a-GCST90060835**	acute RITs	Sphingomyelin(42:2)_[M+H]1+ levels	MR Egger	20.4627	13	0.0843
**ebi-a-GCST90060812**	acute RITs	Sphingomyelin(42:8)_[M+H]1+ levels	MR Egger	2.9891	8	0.9350
**met-d-Total_C**	acute RITs	Total cholesterol	MR Egger	97.2174	111	0.8215
**met-c-898**	acute RITs	Total cholesterol in medium HDL	MR Egger	18.5922	25	0.8162
**ebi-a-GCST90018974**	acute RITs	Total cholesterol levels	MR Egger	271.8808	292	0.7952
**ebi-a-GCST90025953**	acute RITs	Total cholesterol levels	MR Egger	265.2532	279	0.7134
**met-d-non_HDL_C**	acute RITs	Total cholesterol minus HDL-C	MR Egger	102.6963	99	0.3795
**met-d-Total_P**	acute RITs	Total concentration of lipoprotein particles	MR Egger	131.2802	124	0.3101
**met-d-Total_CE**	acute RITs	Total esterified cholesterol	MR Egger	107.7988	111	0.5683
**met-d-Total_FC**	acute RITs	Total free cholesterol	MR Egger	101.6232	111	0.7267
**met-c-957**	acute RITs	Total lipids in chylomicrons and largest VLDL particles	MR Egger	16.4206	23	0.8367

**met-d-HDL_L**	acute RITs	Total lipids in HDL	MR Egger	186.7429	162	0.0890
**met-d-L_LDL_L**	acute RITs	Total lipids in large LDL	MR Egger	95.3385	102	0.6664
**met-d-M_HDL_L**	acute RITs	Total lipids in medium HDL	MR Egger	154.8659	137	0.1411
**met-d-S_LDL_L**	acute RITs	Total lipids in small LDL	MR Egger	97.4380	114	0.8666
**met-d-XL_VLDL_L**	acute RITs	Total lipids in very large VLDL	MR Egger	129.5784	143	0.7824
**ukb-e-30860_CSA**	acute RITs	Total protein	MR Egger	17.0492	21	0.7081
**ebi-a-GCST90060736**	acute RITs	Triacylglycerol(44:1)_[M+NH4]1+ levels	MR Egger	18.3260	17	0.3686
**ebi-a-GCST90060999**	acute RITs	Triacylglycerol(56:3)_[M+NH4]1+ levels	MR Egger	6.6659	9	0.6719
**ukb-e-30870_CSA**	acute RITs	Triglycerides	MR Egger	20.3550	26	0.7744
**met-d-L_HDL_TG_pct**	acute RITs	Triglycerides to total lipids ratio in large HDL	MR Egger	124.0275	155	0.9681
**met-d-L_HDL_TG_pct**	acute RITs	Triglycerides to total lipids ratio in large HDL	MR Egger	124.0275	155	0.9681
**met-d-M_HDL_TG_pct**	acute RITs	Triglycerides to total lipids ratio in medium HDL	MR Egger	124.0843	154	0.9633
**met-d-M_HDL_TG_pct**	acute RITs	Triglycerides to total lipids ratio in medium HDL	MR Egger	124.0843	154	0.9633
**met-d-M_LDL_TG_pct**	acute RITs	Triglycerides to total lipids ratio in medium LDL	MR Egger	102.8794	118	0.8379
**met-d-M_LDL_TG_pct**	acute RITs	Triglycerides to total lipids ratio in medium LDL	MR Egger	102.8794	118	0.8379
**met-d-S_HDL_TG_pct**	acute RITs	Triglycerides to total lipids ratio in small HDL	MR Egger	136.0784	170	0.9739
**met-d-S_HDL_TG_pct**	acute RITs	Triglycerides to total lipids ratio in small HDL	MR Egger	136.0784	170	0.9739
**ieu-a-786**	acute RITs	Urate	MR Egger	3.2230	3	0.3585
**met-c-940**	acute RITs	Valine	MR Egger	19.6295	24	0.7176
**ebi-a-GCST90019468**	acute RITs	von Willebrand factor A domain-containing protein 2 measurement	MR Egger	20.3197	16	0.2062
**met-a-499**	acute RITs	X-11334	MR Egger	23.9050	23	0.4091
**met-a-520**	acute RITs	X-11470	MR Egger	11.9403	8	0.1539
**met-a-548**	acute RITs	X-11820	MR Egger	5.2969	10	0.8705
**met-a-610**	acute RITs	X-12442--5,8-tetradecadienoate	MR Egger	2.9554	10	0.9825
**met-a-701**	acute RITs	X-13671	MR Egger	10.2667	11	0.5066
**met-a-715**	acute RITs	X-14056	MR Egger	8.6462	7	0.2791
**ukb-b-17102**	acute RITs	External causes: Y83.6 Removal of other organ (partial) (total)	MR Egger	6.9199	7	0.4373
**ukb-b-11247**	acute RITs	Main speciality of consultant (recoded): Cardiothoracic surgery	MR Egger	27.2345	20	0.1288
**ukb-b-17277**	acute RITs	Operation code: anal surgery	MR Egger	11.5541	8	0.1722
**ukb-b-6863**	acute RITs	Operation code: caesarean section / caesarian section	MR Egger	35.4099	42	0.7539
**ukb-b-6235**	acute RITs	Operation code: cholecystectomy/gall bladder removal	MR Egger	117.8441	97	0.0738
**ukb-b-6235**	acute RITs	Operation code: cholecystectomy/gall bladder removal	MR Egger	117.8441	97	0.0738
**ukb-b-9263**	acute RITs	Operation code: inguinal/femoral hernia repair	MR Egger	62.1102	73	0.8145
**ukb-b-847**	acute RITs	Operation code: reduction or fixation of bone fracture	MR Egger	33.5870	29	0.2547

**ukb-b-13003**	acute RITs	Operation code: sinus surgery	MR Egger	4.4162	4	0.3526
**ukb-b-15592**	acute RITs	Operation code: varicose vein surgery	MR Egger	184.1349	204	0.8375
**ukb-b-7609**	acute RITs	Operative procedures - main OPCS: B28.2 Partial excision of breast NEC	MR Egger	19.2564	22	0.6294
**ukb-b-4944**	acute RITs	Operative procedures - main OPCS: W85.2 Endoscopic irrigation of knee joint	MR Egger	1.7341	2	0.4202
**ukb-e-41200_p138_AFR**	acute RITs	Operative procedures - main OPCS4	MR Egger	14.8869	23	0.8986
**ukb-e-41200_p26_CSA**	acute RITs	Operative procedures - main OPCS4	MR Egger	14.1987	17	0.6530
**ukb-e-41200_p356_AFR**	acute RITs	Operative procedures - main OPCS4	MR Egger	6.9520	10	0.7300
**ukb-e-41200_p834_AFR**	acute RITs	Operative procedures - main OPCS4	MR Egger	12.0076	13	0.5270
**ukb-e-41200_p991_CSA**	acute RITs	Operative procedures - main OPCS4	MR Egger	3.5719	7	0.8276
**ukb-b-4820**	acute RITs	Operative procedures - secondary OPCS: E13.6 Puncture of maxillary antrum	MR Egger	0.3721	3	0.9459
**ukb-b-4373**	acute RITs	Operative procedures - secondary OPCS: T85.2 Block dissection of axillary lymph nodes	MR Egger	13.1827	15	0.5882
**ukb-b-7815**	acute RITs	Operative procedures - secondary OPCS: U05.1 Computed tomography of head	MR Egger	2.0822	4	0.7206
**ukb-b-13551**	acute RITs	Operative procedures - secondary OPCS: X72.1 Delivery of complex chemotherapy for neoplasm including prolonged infusional treatment at first attendance	MR Egger	3.1400	4	0.5347
**ukb-b-13683**	acute RITs	Operative procedures - secondary OPCS: Y58.8 Other specified harvest of skin for graft	MR Egger	1.7117	1	0.1908
**ukb-b-9816**	acute RITs	Operative procedures - secondary OPCS: Y80.4 Intravenous anaesthetic NEC	MR Egger	5.5015	7	0.5990
**ukb-b-13121**	acute RITs	Operative procedures - secondary OPCS: Z27.1 Oesophagus	MR Egger	4.6374	8	0.7955
**ukb-b-5222**	acute RITs	Operative procedures - secondary OPCS: Z94.1 Bilateral operation	MR Egger	20.0048	16	0.2200
**ukb-e-Y83_CSA**	acute RITs	Y83 Surgical operation and other surgical procedures as the cause of abnormal reaction of the patient, or of later complication, without mention of misadventure at the time of the procedure	MR Egger	16.4235	14	0.2882
**ukb-b-13350**	acute RITs	PCT responsible for patient data: NOTTINGHAM CITY PCT	MR Egger	8.4598	14	0.8640
**ukb-b-6873**	acute RITs	PCT responsible for patient data: WESTERN CHESHIRE PCT	MR Egger	2.4522	4	0.6532
**ukb-b-1539**	acute RITs	PCT where patients GP was registered: BROXTOWE AND HUCKNALL PCT	MR Egger	8.7518	17	0.9477
**ukb-b-6547**	acute RITs	PCT where patients GP was registered: BURY PCT	MR Egger	36.2496	33	0.3195
**ukb-b-8205**	acute RITs	PCT where patients GP was registered: CAMDEN PCT	MR Egger	5.8216	11	0.8850
**ukb-b-2838**	acute RITs	PCT where patients GP was registered: DURHAM AND CHESTER-LE-STREET PCT	MR Egger	17.3936	17	0.4280
**ukb-b-19050**	acute RITs	PCT where patients GP was registered: EAST LANCASHIRE TEACHING PCT	MR Egger	5.5049	3	0.1383
**ukb-b-14654**	acute RITs	PCT where patients GP was registered: ROCHDALE PCT	MR Egger	4.9036	8	0.7678
**ukb-b-8842**	acute RITs	PCT where patients GP was registered: SOLIHULL PCT	MR Egger	0.3805	4	0.9840
**ukb-b-7288**	acute RITs	3mm asymmetry angle (left)	MR Egger	37.0080	43	0.7277
**ukb-e-5108_CSA**	acute RITs	3mm asymmetry angle (right)	MR Egger	10.0370	12	0.6127
**ukb-e-5156_AFR**	acute RITs	3mm asymmetry index (left)	MR Egger	20.3164	23	0.6228
**ukb-e-5107_AFR**	acute RITs	3mm strong meridian angle (right)	MR Egger	13.1425	11	0.2841
**ukb-b-4874**	acute RITs	6mm asymmetry angle (right)	MR Egger	32.2535	29	0.3089
**ukb-e-5306_CSA**	acute RITs	6mm index of best keratometry results (left)	MR Egger	20.4248	20	0.4317
**ieu-a-1095**	acute RITs	Age at menarche	MR Egger	146.0139	152	0.6216

**ukb-d-4700_irnt**	acute RITs	Age cataract diagnosed	MR Egger	11.3915	16	0.7847
**ukb-b-1061**	acute RITs	Age high blood pressure diagnosed	MR Egger	68.7719	60	0.2047
**ukb-e-2217_AFR**	acute RITs	Age started wearing glasses or contact lenses	MR Egger	25.4190	20	0.1859
**ukb-b-5090**	acute RITs	Astigmatism angle (left)	MR Egger	18.0268	24	0.8017
**ukb-e-B96_CSA**	acute RITs	B96 Other bacterial agents as the cause of diseases classified to other chapters	MR Egger	22.3433	14	0.0718
**ukb-e-1677_CSA**	acute RITs	Breastfed as a baby	MR Egger	2.9398	6	0.8164
**ukb-e-1468_p1_CSA**	acute RITs	Cereal type	MR Egger	17.1180	21	0.7039
**ukb-e-24014_AFR**	acute RITs	Close to major road	MR Egger	13.1240	14	0.5168
**ukb-e-5086_AFR**	acute RITs	Cylindrical power (left)	MR Egger	20.5199	13	0.0830
**ukb-b-7196**	acute RITs	Delivery methods: Elective caesarean section	MR Egger	1.0145	2	0.6021
**ukb-e-894_AFR**	acute RITs	Duration of moderate activity	MR Egger	18.2296	16	0.3106
**ukb-e-874_p1_AFR**	acute RITs	Duration of walks	MR Egger	24.2710	22	0.3332
**ukb-e-404_CSA**	acute RITs	Duration to first press of snap-button in each round	MR Egger	13.1669	18	0.7816
**ukb-d-20411_0**	acute RITs	Ever been injured or injured someone else through drinking alcohol: No	MR Egger	15.2842	16	0.5039
**ukb-e-2296_CSA**	acute RITs	Falls in the last year	MR Egger	13.5090	17	0.7015
**ukb-e-4935_CSA**	acute RITs	FI1 : numeric addition test	MR Egger	13.0964	14	0.5189
**ukb-d-4979**	acute RITs	FI5 : family relationship calculation	MR Egger	21.9432	25	0.6390
**ukb-b-17738**	acute RITs	Fractured bone site(s): Other bones	MR Egger	22.7185	28	0.7470
**ukb-a-439**	acute RITs	Fractured bone site(s): Wrist	MR Egger	21.5621	21	0.4251
**ukb-b-9571**	acute RITs	Fractured bone site(s): Wrist	MR Egger	24.1459	24	0.4533
**ebi-a-GCST90013913**	acute RITs	Fractured bones in last 5 years (UKB data field 2463) (Firth correction)	MR Egger	65.1044	65	0.4730
**ukb-a-2**	acute RITs	Frequency of light DIY in last 4 weeks	MR Egger	28.1083	23	0.2117
**ukb-e-4570_AFR**	acute RITs	Friendships satisfaction	MR Egger	9.4209	20	0.9775
**ebi-a-GCST90019399**	acute RITs	Gelsolin measurement	MR Egger	20.1901	18	0.3222
**ukb-a-340**	acute RITs	Hearing aid user	MR Egger	17.2335	25	0.8731
**ukb-b-19060**	acute RITs	Hearing aid user	MR Egger	30.5920	38	0.7980
**ukb-e-4849_p2_AFR**	acute RITs	Hearing test done	MR Egger	25.0532	23	0.3475
**ukb-e-6141_p2_AFR**	acute RITs	How are people in household related to participant	MR Egger	16.9368	18	0.5275
**ukb-e-6145_p2_AFR**	acute RITs	Illness, injury, bereavement, stress in last 2 years	MR Egger	28.2651	29	0.5038
**ukb-e-6145_p4_CSA**	acute RITs	Illness, injury, bereavement, stress in last 2 years	MR Egger	6.4347	11	0.8428
**ukb-b-14449**	acute RITs	Index of best refractometry result (left)	MR Egger	13.9184	14	0.4558
**ukb-e-41244_p2_CSA**	acute RITs	Intended management of patient (recoded)	MR Egger	20.0412	18	0.3305
**ukb-b-16311**	acute RITs	Interval between previous point and current one in alphanumeric path (trail #2)	MR Egger	58.3546	71	0.8588
**ebi-a-GCST90013453**	acute RITs	Lack of behavioral control	MR Egger	3.7368	5	0.5879
**ukb-e-4728_CSA**	acute RITs	Leg pain on walking	MR Egger	23.6794	21	0.3089
**ukb-e-6160_p5_AFR**	acute RITs	Leisure/social activities	MR Egger	10.2235	14	0.7457

**ukb-e-41245_p20_CSA**	acute RITs	Main speciality of consultant (recoded)	MR Egger	9.8404	9	0.3636
**ukb-e-41245_p6_AFR**	acute RITs	Main speciality of consultant (recoded)	MR Egger	6.0494	16	0.9876
**ukb-a-392**	acute RITs	Maximum workload during fitness test	MR Egger	10.1610	15	0.8095
**ebi-a-GCST90002397**	acute RITs	Mean spheric corpuscular volume	MR Egger	568.9903	635	0.9714
**ukb-e-41249_p4_AFR**	acute RITs	Methods of admission to hospital (recoded)	MR Egger	16.3387	20	0.6954
**ukb-b-5477**	acute RITs	Methods of admission to hospital (recoded): Elective admission	MR Egger	218.1418	235	0.7782
**ukb-b-10655**	acute RITs	Methods of admission to hospital (recoded): Emergency admission	MR Egger	68.1550	59	0.1940
**ukb-b-4540**	acute RITs	Methods of admission to hospital (recoded): Emergency admission: Non-injury	MR Egger	143.6598	132	0.2302
**ukb-b-19790**	acute RITs	Methods of admission to hospital (recoded): Maternity admission: Post-partum	MR Egger	11.7766	16	0.7592
**ukb-e-41250_p1_AFR**	acute RITs	Methods of discharge from hospital (recoded)	MR Egger	14.9280	20	0.7805
**ukb-e-52_p9_CSA**	acute RITs	Month of birth	MR Egger	4.9429	3	0.1760
**ukb-a-12**	acute RITs	Nap during day	MR Egger	195.0925	190	0.3847
**ukb-d-2654_8**	acute RITs	Non-butter spread type details: Other low or reduced fat spread	MR Egger	10.3347	16	0.8486
**ukb-d-2654_9**	acute RITs	Non-butter spread type details: Other type of spread/margarine	MR Egger	2.9608	7	0.8886
**ukb-d-2654_4**	acute RITs	Non-butter spread type details: Soft (tub) margarine	MR Egger	32.6722	32	0.4338
**ukb-e-4291_CSA**	acute RITs	Number of attempts	MR Egger	11.4618	8	0.1769
**ieu-b-4827**	acute RITs	Number of children ever born measurement	MR Egger	7.3527	13	0.8831
**ukb-b-2988**	acute RITs	Number of fluid intelligence questions attempted within time limit	MR Egger	106.7568	104	0.4069
**ukb-e-399_CSA**	acute RITs	Number of incorrect matches in round	MR Egger	7.7805	9	0.5564
**ukb-b-1209**	acute RITs	Number of live births	MR Egger	85.6500	76	0.2103
**ukb-b-6891**	acute RITs	Number of triplets attempted (left)	MR Egger	11.6528	13	0.5563
**ukb-b-4909**	acute RITs	Number of triplets attempted (right)	MR Egger	20.9917	23	0.5816
**ukb-e-4276_AFR**	acute RITs	Number of triplets attempted (right)	MR Egger	1.3440	3	0.7187
**ebi-a-GCST90012790**	acute RITs	Percentage of invited food questionnaires completed	MR Egger	126.9848	116	0.2287
**ieu-b-4860**	acute RITs	Physical activity	MR Egger	26.5411	18	0.0880
**ebi-a-GCST90061410**	acute RITs	Physical activity (Total log acceleration 2am-4am)	MR Egger	33.2074	25	0.1260
**ukb-e-20018_AFR**	acute RITs	Prospective memory result	MR Egger	5.5098	9	0.7878
**ukb-a-421**	acute RITs	Reason for glasses/contact lenses: For just reading/near work as you are getting older (called 'presbyopia')	MR Egger	32.2285	41	0.8347
**ukb-a-420**	acute RITs	Reason for glasses/contact lenses: For long-sightedness i.e. for distance and near but particularly for near tasks like reading (called 'hypermetropia')	MR Egger	24.9044	37	0.9354
**ukb-d-2664_5**	acute RITs	Reason for reducing amount of alcohol drunk: Other reason	MR Egger	28.2510	26	0.3462
**ukb-e-20019_AFR**	acute RITs	Speech-reception-threshold (SRT) estimate (left)	MR Egger	14.3326	14	0.4252
**ukb-a-343**	acute RITs	Time from waking to first cigarette	MR Egger	32.8217	26	0.1673
**ukb-b-2732**	acute RITs	Time from waking to first cigarette	MR Egger	28.0102	22	0.1753
**ukb-a-7**	acute RITs	Time spent driving	MR Egger	33.0348	45	0.9070
**ukb-d-20531**	acute RITs	Victim of sexual assault	MR Egger	36.5476	23	0.0362

**ukb-b-3709**	acute RITs	Wants to stop smoking	MR Egger	17.6508	17	0.4112
**ukb-e-3659_CSA**	acute RITs	Year immigrated to UK (United Kingdom)	MR Egger	26.6268	19	0.1137
**ukb-e-Z53_AFR**	acute RITs	Z53 Persons encountering health services for specifie procedures, not carried out	MR Egger	8.3967	13	0.8168
**ukb-e-23119_CSA**	acute RITs	Arm fat percentage (right)	MR Egger	15.0116	19	0.7219
**ukb-e-23121_AFR**	acute RITs	Arm fat-free mass (right)	MR Egger	42.2560	30	0.0680
**ukb-b-8909**	acute RITs	Body fat percentage	MR Egger	657.7912	642	0.3243
**ukb-e-23099_AFR**	acute RITs	Body fat percentage	MR Egger	22.9978	20	0.2889
**ukb-e-23104_AFR**	acute RITs	Body mass index (BMI)	MR Egger	25.6392	23	0.3182
**ebi-a-GCST90092504**	acute RITs	Carotid Intima-media thickness (mean of the maximum cIMT)	MR Egger	21.1615	20	0.3877
**ebi-a-GCST008029**	acute RITs	Diastolic blood pressure	MR Egger	57.3612	51	0.2512
**ieu-b-106**	acute RITs	FEV1/FVC < 0.7	MR Egger	239.1013	243	0.5586
**ieu-b-4853**	acute RITs	Forced expiratory volume in 1-second	MR Egger	2.4983	6	0.8687
**ukb-e-46_AFR**	acute RITs	Hand grip strength (left)	MR Egger	16.7689	15	0.3329
**ukb-b-8875**	acute RITs	Heel bone mineral density (BMD)	MR Egger	558.4835	543	0.3136
**ukb-a-500**	acute RITs	Heel bone mineral density (BMD) T-score automated	MR Egger	438.4338	398	0.0792
**ukb-a-500**	acute RITs	Heel bone mineral density (BMD) T-score automated	MR Egger	438.4338	398	0.0792
**ukb-a-361**	acute RITs	Heel bone mineral density (BMD) T-score automated (left)	MR Egger	261.8694	255	0.3703
**ukb-a-361**	acute RITs	Heel bone mineral density (BMD) T-score automated (left)	MR Egger	261.8694	255	0.3703
**ukb-a-362**	acute RITs	Heel bone mineral density (BMD) T-score automated (right)	MR Egger	254.3059	265	0.6707
**ukb-a-362**	acute RITs	Heel bone mineral density (BMD) T-score automated (right)	MR Egger	254.3059	265	0.6707
**ukb-b-20124**	acute RITs	Heel bone mineral density (BMD) T-score, automated	MR Egger	573.2515	539	0.1487
**ukb-b-11364**	acute RITs	Heel bone mineral density (BMD), manual entry	MR Egger	117.6572	116	0.4396
**ukb-b-17612**	acute RITs	Heel bone ultrasound T-score, manual entry	MR Egger	84.4377	81	0.3750
**ukb-b-17612**	acute RITs	Heel bone ultrasound T-score, manual entry	MR Egger	84.4377	81	0.3750
**ukb-b-17952**	acute RITs	Heel Broadband ultrasound attenuation (BUA), manual entry	MR Egger	97.6496	90	0.2729
**ukb-b-17952**	acute RITs	Heel Broadband ultrasound attenuation (BUA), manual entry	MR Egger	97.6496	90	0.2729
**ukb-b-5447**	acute RITs	Heel broadband ultrasound attenuation (left)	MR Egger	300.2056	302	0.5184
**ukb-b-5447**	acute RITs	Heel broadband ultrasound attenuation (left)	MR Egger	300.2056	302	0.5184
**ukb-b-6027**	acute RITs	Heel broadband ultrasound attenuation (right)	MR Egger	297.2692	309	0.6740
**ukb-b-6027**	acute RITs	Heel broadband ultrasound attenuation (right)	MR Egger	297.2692	309	0.6740
**ukb-b-6027**	acute RITs	Heel broadband ultrasound attenuation (right)	MR Egger	297.2692	309	0.6740
**ukb-b-15851**	acute RITs	Heel Broadband ultrasound attenuation, direct entry	MR Egger	504.8827	478	0.1908
**ukb-b-19234**	acute RITs	Heel quantitative ultrasound index (QUI), direct entry	MR Egger	573.1370	539	0.1495
**ukb-b-19234**	acute RITs	Heel quantitative ultrasound index (QUI), direct entry	MR Egger	573.1370	539	0.1495
**ukb-b-17848**	acute RITs	Heel quantitative ultrasound index (QUI), manual entry	MR Egger	105.8760	112	0.6451
**ukb-e-49_AFR**	acute RITs	Hip circumference	MR Egger	18.9693	14	0.1661

**ukb-e-23110_AFR**	acute RITs	Impedance of arm (left)	MR Egger	22.6800	20	0.3048
**ebi-a-GCST90020194**	acute RITs	Inferior Posterior lobe of cerebellar volume (including Crus II to IX hemispheric lobules)	MR Egger	78.8848	71	0.2438
**ukb-b-19277**	acute RITs	Intra-ocular pressure, corneal-compensated (right)	MR Egger	163.8266	141	0.0915
**bbj-a-37**	acute RITs	Mean arterial pressure	MR Egger	86.5667	81	0.3157
**ebi-a-GCST90018743**	acute RITs	Mean arterial pressure	MR Egger	104.4344	97	0.2849
**ukb-e-MAP_p2_AFR**	acute RITs	Mean arterial pressure, automated reading, adjusted by medication	MR Egger	29.1179	22	0.1415
**ukb-e-MAP_p4_AFR**	acute RITs	Mean arterial pressure, combined automated + manual reading, adjusted by medication	MR Egger	33.6551	23	0.0703
**ukb-d-30050_irnt**	acute RITs	Mean corpuscular haemoglobin	MR Egger	429.4260	455	0.8001
**ebi-a-GCST004630**	acute RITs	Mean corpuscular hemoglobin	MR Egger	304.7894	294	0.3202
**ebi-a-GCST90002390**	acute RITs	Mean corpuscular hemoglobin	MR Egger	557.7483	545	0.3434
**ebi-a-GCST90002391**	acute RITs	Mean corpuscular hemoglobin concentration	MR Egger	331.7647	324	0.3712
**ebi-a-GCST90002391**	acute RITs	Mean corpuscular hemoglobin concentration	MR Egger	331.7647	324	0.3712
**ebi-a-GCST90025962**	acute RITs	Mean corpuscular hemoglobin concentration	MR Egger	473.0245	496	0.7642
**ebi-a-GCST004602**	acute RITs	Mean corpuscular volume	MR Egger	317.2899	312	0.4062
**ebi-a-GCST90002392**	acute RITs	Mean corpuscular volume	MR Egger	632.4465	610	0.2566
**ebi-a-GCST90025963**	acute RITs	Mean corpuscular volume	MR Egger	514.6082	528	0.6536
**ukb-d-30040_irnt**	acute RITs	Mean corpuscular volume	MR Egger	464.2624	475	0.6291
**ebi-a-GCST90013979**	acute RITs	Mean corpuscular volume (UKB data field 30040)	MR Egger	498.0661	489	0.3785
**ukb-a-251**	acute RITs	Overall health rating	MR Egger	275.3932	260	0.2447
**ieu-a-274**	acute RITs	Packed cell volume	MR Egger	35.6354	44	0.8114
**ebi-a-GCST90000065**	acute RITs	Pulse pressure	MR Egger	309.7947	284	0.1403
**ukb-e-PP_p1_CSA**	acute RITs	Pulse pressure, automated reading	MR Egger	11.0344	15	0.7502
**ukb-e-R19_CSA**	acute RITs	R19 Other symptoms and signs involving the digestive system and abdomen	MR Egger	14.7213	10	0.1426
**ebi-a-GCST90020192**	acute RITs	Superior Posterior lobe of cerebellar volume (including VI to Crus I hemispheric lobules)	MR Egger	71.8201	83	0.8045
**ebi-a-GCST005349**	acute RITs	Total body bone mineral density (age over 60)	MR Egger	70.3462	71	0.4996
**ebi-a-GCST90020190**	acute RITs	Total cerebellar volume (excluding Crus I vermis)	MR Egger	73.9464	58	0.0772
**ukb-e-23128_AFR**	acute RITs	Trunk fat mass	MR Egger	24.0201	21	0.2921
**ukb-e-23127_AFR**	acute RITs	Trunk fat percentage	MR Egger	35.3352	31	0.2707
**ebi-a-GCST90002228**	acute RITs	Two-hour glucose	MR Egger	12.8293	14	0.5400
**ebi-a-GCST90013422**	acute RITs	Ultradistal forearm bone mineral density	MR Egger	44.1418	43	0.4231
**ieu-a-61**	acute RITs	Waist circumference	MR Egger	92.6402	103	0.7582
**ieu-a-72**	acute RITs	Waist-to-hip ratio	MR Egger	87.8626	81	0.2821
**ebi-a-GCST90095035**	acute RITs	Waist-to-hip ratio adjusted for BMI	MR Egger	25.2449	29	0.6655
**ukb-d-5610_3**	acute RITs	Which eye(s) affected by presbyopia: Both eyes	MR Egger	9.0877	15	0.8729
**ukb-e-Z09_CSA**	acute RITs	Z09 Follow-up examination after treatment for conditions other than malignant neoplasms	MR Egger	7.1514	8	0.5204
**ukb-e-Z12_CSA**	acute RITs	Z12 Special screening examination for neoplasms	MR Egger	17.5234	10	0.0636

**ukb-b-5779**	acute RITs	Alcohol intake frequency.	MR Egger	295.3668	293	0.4502
**ukb-a-32**	acute RITs	Alcohol intake versus 10 years previously	MR Egger	78.5193	66	0.1390
**ukb-e-1628_CSA**	acute RITs	Alcohol intake versus 10 years previously	MR Egger	22.6753	19	0.2519
**ieu-b-4825**	acute RITs	Cigarettes smoked per day	MR Egger	8.1857	14	0.8794
**ukb-b-1572**	acute RITs	Difficulty not smoking for 1 day	MR Egger	12.5113	20	0.8974
**ukb-d-20541**	acute RITs	Difficulty stopping worrying during worst period of anxiety	MR Egger	10.7982	15	0.7668
**ukb-d-20425**	acute RITs	Ever worried more than most people would in similar situation	MR Egger	39.4367	33	0.2042
**ebi-a-GCST006944**	acute RITs	Experiencing mood swings	MR Egger	143.3515	154	0.7201
**ieu-b-117**	acute RITs	HOMA-B	MR Egger	18.2841	13	0.1470
**ukb-a-47**	acute RITs	Irritability	MR Egger	148.9571	130	0.1222
**ebi-a-GCST90013875**	acute RITs	Irritability (UKB data field 1940) (Firth correction)	MR Egger	149.4422	146	0.4056
**ebi-a-GCST90013925**	acute RITs	Irritability (UKB data field 1940) (SPA correction)	MR Egger	149.4271	146	0.4060
**ebi-a-GCST90013873**	acute RITs	Mood swings (UKB data field 1920) (Firth correction)	MR Egger	154.9898	160	0.5970
**ebi-a-GCST90013923**	acute RITs	Mood swings (UKB data field 1920) (SPA correction)	MR Egger	154.9934	160	0.5970
**ukb-a-342**	acute RITs	Number of cigarettes currently smoked daily (current cigarette smokers)	MR Egger	15.1543	22	0.8555
**ukb-a-238**	acute RITs	Pack years adult smoking as proportion of life span exposed to smoking PREVIEW ONLY	MR Egger	68.2635	61	0.2442
**ukb-a-237**	acute RITs	Pack years of smoking PREVIEW ONLY	MR Egger	60.4163	60	0.4607
**ieu-a-1046**	acute RITs	Pallidum volume	MR Egger	7.1814	10	0.7082
**ebi-a-GCST008027**	acute RITs	Smoking behavior (cigarettes smoked per day)	MR Egger	34.3637	28	0.1892
**ukb-e-2010_AFR**	acute RITs	Suffer from 'nerves'	MR Egger	12.2483	14	0.5864
**ebi-a-GCST90025968**	acute RITs	Systolic blood pressure	MR Egger	522.7550	496	0.1961
**ieu-b-38**	acute RITs	systolic blood pressure	MR Egger	568.0064	640	0.9810
**ebi-a-GCST90014018**	acute RITs	Systolic blood pressure automated reading (UKB data field 4080)	MR Egger	427.8090	398	0.1458
**ukb-e-SBP_p1_AFR**	acute RITs	Systolic blood pressure, automated reading, adjusted by medication	MR Egger	30.1102	27	0.3092
**ukb-e-SBP_p2_AFR**	acute RITs	Systolic blood pressure, combined automated + manual reading	MR Egger	16.6060	17	0.4814
**ukb-e-SBP_p3_AFR**	acute RITs	Systolic blood pressure, combined automated + manual reading, adjusted by medication	MR Egger	22.5843	19	0.2561
**ukb-e-6164_p6_CSA**	acute RITs	Types of physical activity in last 4 weeks	MR Egger	0.8198	5	0.9758
**ukb-d-22612_2**	acute RITs	Worked with materials containing asbestos: Often	MR Egger	11.8093	14	0.6216
**ukb-d-22609_0**	acute RITs	Workplace very dusty: Rarely/never	MR Egger	10.3547	17	0.8881
**ieu-a-1010**	acute RITs	Years of schooling	MR Egger	109.3657	94	0.1329
**ukb-e-Z88_AFR**	acute RITs	Z88 Personal history of allergy to drugs, medicaments and biologocal substances	MR Egger	21.4374	17	0.2073

Table S4 Pleiotropy test results for univariate Mendelian randomization between 990 traits and acute radiation-induced toxicities (RITs)

Exposure ID	Outcome	Trait name	Egger intercept	Se	*P* value
**ebi-a-GCST90011301**	acute RITs	C_Actinobacteria abundance	0.0027	0.0080	0.7395
**ebi-a-GCST90011305**	acute RITs	C_Clostridia abundance	0.0105	0.0137	0.4550
**ebi-a-GCST90011316**	acute RITs	F_Rikenellaceae abundance	0.0079	0.0141	0.5842
**ebi-a-GCST90011330**	acute RITs	G_Faecalibacterium abundance	-0.0028	0.0051	0.5986
**ebi-a-GCST90011333**	acute RITs	G_Lachnospiraceae abundance	-0.0011	0.0106	0.9206
**ebi-a-GCST90027467**	acute RITs	Gut bacterial pathway abundance (FUC.RHAMCAT.PWY..superpathway.of.fucose.and.rhamnose.degradation)	0.0450	0.0177	0.0516
**ebi-a-GCST90027487**	acute RITs	Gut bacterial pathway abundance (KETOGLUCONMET.PWY..ketogluconate.metabolism)	-0.0246	0.0144	0.1227
**ebi-a-GCST90027501**	acute RITs	Gut bacterial pathway abundance (P42.PWY..incomplete.reductive.TCA.cycle)	-0.0294	0.0142	0.0597
**ebi-a-GCST90027548**	acute RITs	Gut bacterial pathway abundance (PWY.5345..superpathway.of.L.methionine.biosynthesis..by.sulfhydrylation.)	0.0093	0.0144	0.5349
**ebi-a-GCST90027577**	acute RITs	Gut bacterial pathway abundance (PWY.6285..superpathway.of.fatty.acids.biosynthesis..E..coli.)	-0.0163	0.0185	0.3995
**ebi-a-GCST90027623**	acute RITs	Gut bacterial pathway abundance (PWY.7371..1.4.dihydroxy.6.naphthoate.biosynthesis.II)	-0.0359	0.0167	0.0683
**ebi-a-GCST90027627**	acute RITs	Gut bacterial pathway abundance (PWY.7456..mannan.degradation)	-0.0226	0.0430	0.6357
**ebi-a-GCST90027635**	acute RITs	Gut bacterial pathway abundance (PWY_REDCITCYC..TCA.cycle.VIII..helicobacter.)	-0.0181	0.0145	0.2455
**ebi-a-GCST90027520**	acute RITs	Gut bacterial pathway abundance (PWY0.1338..polymyxin.resistance)	-0.0021	0.0189	0.9128
**ebi-a-GCST90027524**	acute RITs	Gut bacterial pathway abundance (PWY0.1586..peptidoglycan.maturation..meso.diaminopimelate.containing.)	-0.0001	0.0142	0.9947
**ebi-a-GCST90027640**	acute RITs	Gut bacterial pathway abundance (RHAMCAT.PWY..L.rhamnose.degradation.I)	-0.0109	0.0309	0.7313
**ebi-a-GCST90016910**	acute RITs	Gut microbiota abundance (class Bacilli id.1673)	-0.0011	0.0099	0.9164
**ebi-a-GCST90016927**	acute RITs	Gut microbiota abundance (family Bacteroidaceae id.917)	-0.0012	0.0198	0.9521
**ebi-a-GCST90016928**	acute RITs	Gut microbiota abundance (family Bacteroidales S24 7group id.11173)	0.0042	0.0171	0.8127
**ebi-a-GCST90016952**	acute RITs	Gut microbiota abundance (family Streptococcaceae id.1850)	0.0229	0.0134	0.1124
**ebi-a-GCST90016968**	acute RITs	Gut microbiota abundance (genus Bacteroides id.918)	-0.0012	0.0198	0.9521
**ebi-a-GCST90017008**	acute RITs	Gut microbiota abundance (genus Family XIII AD3011 group id.11293)	0.0201	0.0178	0.2842
**ebi-a-GCST90017009**	acute RITs	Gut microbiota abundance (genus Family XIII UCG001 id.11294)	-0.0129	0.0117	0.3146
**ebi-a-GCST90017028**	acute RITs	Gut microbiota abundance (genus Lachnospiraceae UCG010 id.11330)	0.0020	0.0093	0.8386
**ebi-a-GCST90017040**	acute RITs	Gut microbiota abundance (genus Paraprevotella id.962)	-0.0145	0.0126	0.2746
**ebi-a-GCST90017046**	acute RITs	Gut microbiota abundance (genus Rikenellaceae RC9 gut group id.11191)	0.0021	0.0207	0.9230
**ebi-a-GCST90027748**	acute RITs	Gut microbiota abundance (k_Bacteria.p_Actinobacteria)	-0.0193	0.0170	0.2891
**ebi-a-GCST90027651**	acute RITs	Gut microbiota abundance (k_Bacteria.p_Actinobacteria.c_Actinobacteria)	-0.0193	0.0170	0.2890
**ebi-a-GCST90027824**	acute RITs	Gut microbiota abundance (k_Bacteria.p_Bacteroidetes.c_Bacteroidia.o_Bacteroidales.f_Bacteroidaceae.g_Bacteroides.s_Bacteroides_dorei)	-0.0047	0.0173	0.7922
**ebi-a-GCST90027765**	acute RITs	Gut microbiota abundance (k_Bacteria.p_Bacteroidetes.c_Bacteroidia.o_Bacteroidales.f_Porphyromonadaceae.g_Odoribacter.s_Odoribacter_splanchnicus)	-0.0123	0.0111	0.2916
**ebi-a-GCST90027803**	acute RITs	Gut microbiota abundance (k_Bacteria.p_Firmicutes.c_Erysipelotrichia.o_Erysipelotrichales.f_Erysipelotrichaceae.g_Erysipelotrichaceae_noname.s_Eubacterium_biforme)	0.0205	0.0166	0.2638

**ebi-a-GCST90027805**	acute RITs	Gut microbiota abundance (k_Bacteria.p_Firmicutes.c_Erysipelotrichia.o_Erysipelotrichales.f_Erysipelotrichaceae.g_Holdemania.s_Holdemania_unclassified)	-0.0154	0.0299	0.6237
**ebi-a-GCST90017101**	acute RITs	Gut microbiota abundance (order Lactobacillales id.1800)	0.0067	0.0087	0.4522
**ebi-a-GCST90017112**	acute RITs	Gut microbiota abundance (phylum Cyanobacteria id.1500)	0.0062	0.0209	0.7752
**ebi-a-GCST90017079**	acute RITs	Gut microbiota abundance (unknown genus id.1000005479)	0.0042	0.0171	0.8127
**ebi-a-GCST90017082**	acute RITs	Gut microbiota abundance (unknown genus id.2001)	-0.0041	0.0132	0.7613
**ebi-a-GCST90032456**	acute RITs	Lactobacillus B abundance in stool	-0.0114	0.0061	0.0833
**ebi-a-GCST90032488**	acute RITs	Negativibacillus massiliensis abundance in stool	-0.0089	0.0090	0.3375
**ebi-a-GCST90011556**	acute RITs	OTU97_100 (Ruminococcaceae) prevalence	0.0012	0.0185	0.9506
**ebi-a-GCST90011359**	acute RITs	OTU97_106 (Ruminococcaceae) abundance	0.0187	0.0261	0.4994
**ebi-a-GCST90011570**	acute RITs	OTU97_130 (Butyrivibrio) prevalence	-0.0075	0.0168	0.6735
**ebi-a-GCST90011572**	acute RITs	OTU97_138 (Oscillibacter) prevalence	-0.0087	0.0152	0.5779
**ebi-a-GCST90011372**	acute RITs	OTU97_140 (Bacteroides) abundance	0.0103	0.0074	0.1844
**ebi-a-GCST90011385**	acute RITs	OTU97_24 (Prevotella) abundance	-0.0215	0.0189	0.2874
**ebi-a-GCST90011604**	acute RITs	OTU97_53 (Bacteroides) prevalence	0.0056	0.0125	0.6622
**ebi-a-GCST90011605**	acute RITs	OTU97_56 (Ruminococcaceae) prevalence	-0.0002	0.0093	0.9800
**ebi-a-GCST90011636**	acute RITs	OTU99_155 (Butyrivibrio) prevalence	-0.0080	0.0163	0.6404
**ebi-a-GCST90011440**	acute RITs	OTU99_171 (Bacteroides) abundance	0.0167	0.0079	0.0527
**ebi-a-GCST90011655**	acute RITs	OTU99_34 (Holdemanella) prevalence	-0.0059	0.0146	0.6927
**ebi-a-GCST90011465**	acute RITs	OTU99_558 (Bacteroidales) abundance	-0.0062	0.0081	0.4569
**ebi-a-GCST90011675**	acute RITs	OTU99_8 (Ruminococcaceae) prevalence	-0.0166	0.0214	0.4620
**ebi-a-GCST90011481**	acute RITs	P_Actinobacteria abundance	0.0027	0.0080	0.7395
**ebi-a-GCST90011482**	acute RITs	P_Bacteroidetes abundance	-0.0064	0.0081	0.4506
**ebi-a-GCST90032500**	acute RITs	Parabacteroides sp000436495 abundance in stool	-0.0162	0.0097	0.1272
**ebi-a-GCST90032502**	acute RITs	Parachlamydiales abundance in stool	-0.0144	0.0118	0.2566
**ebi-a-GCST90032540**	acute RITs	Roseibacillus abundance in stool	-0.0046	0.0172	0.8062
**ebi-a-GCST90032569**	acute RITs	Succiniclasticum abundance in stool	-0.0016	0.0057	0.7821
**ebi-a-GCST90032578**	acute RITs	Terrisporobacter abundance in stool	-0.0212	0.0112	0.0818
**ebi-a-GCST90011687**	acute RITs	TestASV_14 (Lachnospiraceae) prevalence	-0.0083	0.0269	0.7694
**ebi-a-GCST90032590**	acute RITs	UBA1033 sp001695555 abundance in stool	-0.0113	0.0107	0.3141
**ebi-a-GCST90032601**	acute RITs	UBA1409 abundance in stool	-0.0132	0.0099	0.2151
**ebi-a-GCST90032608**	acute RITs	UBA1777 sp900316255 abundance in stool	-0.0093	0.0106	0.3985
**ebi-a-GCST90032616**	acute RITs	UBA3855 sp900316885 abundance in stool	0.0174	0.0118	0.1654
**ebi-a-GCST90032618**	acute RITs	UBA6382 abundance in stool	-0.0089	0.0081	0.2955
**ebi-a-GCST90032628**	acute RITs	UBA737 sp002451855 abundance in stool	-0.0061	0.0103	0.5609
**ieu-b-4810**	acute RITs	Breast cancer	-0.0018	0.0028	0.5287

**ukb-a-57**	acute RITs	Cancer code self-reported: prostate cancer	-0.0011	0.0051	0.8377
**ukb-b-13545**	acute RITs	Cancer code, self-reported: uterine/endometrial cancer	-0.0097	0.0583	0.8788
**ebi-a-GCST90018817**	acute RITs	Cervical cancer	0.0063	0.0067	0.3604
**ebi-a-GCST012876**	acute RITs	Colorectal cancer	0.0042	0.0069	0.5489
**ebi-a-GCST012877**	acute RITs	Colorectal cancer	0.0128	0.0065	0.0547
**ebi-a-GCST90018588**	acute RITs	Colorectal cancer	-0.0013	0.0045	0.7669
**ebi-a-GCST90018838**	acute RITs	Endometrial cancer	0.0129	0.0101	0.2234
**ieu-a-1163**	acute RITs	ER- Breast cancer (Survival)	-0.0139	0.0084	0.1140
**ebi-a-GCST90018875**	acute RITs	Lung cancer	0.0054	0.0073	0.4714
**ieu-a-985**	acute RITs	Lung cancer	-0.0086	0.0046	0.0702
**ebi-a-GCST004747**	acute RITs	Lung cancer in never smokers	-0.0133	0.0075	0.0901
**ukb-b-14956**	acute RITs	Type of cancer: ICD10: C54.1 Endometrium	0.0261	0.0771	0.7673
**ebi-a-GCST90001501**	acute RITs	Activated & secreting CD4 regulatory T cell Absolute Count	-0.0050	0.0079	0.5372
**ebi-a-GCST90001694**	acute RITs	CD28- CD4-CD8- T cell %T cell	-0.0032	0.0052	0.5388
**ebi-a-GCST90001654**	acute RITs	CD28- CD4-CD8- T cell Absolute Count	-0.0019	0.0045	0.6782
**ebi-a-GCST90001641**	acute RITs	CD3- lymphocyte %leukocyte	0.0013	0.0060	0.8376
**ebi-a-GCST90001491**	acute RITs	CD39+ activated CD4 regulatory T cell %CD4 regulatory T cell	-0.0006	0.0043	0.8957
**ebi-a-GCST90001489**	acute RITs	CD39+ activated CD4 regulatory T cell Absolute Count	0.0022	0.0038	0.5629
**ebi-a-GCST90001495**	acute RITs	CD39+ secreting CD4 regulatory T cell Absolute Count	-0.0010	0.0047	0.8402
**ebi-a-GCST90001548**	acute RITs	Central Memory CD8+ T cell Absolute Count	-0.0017	0.0074	0.8250
**ebi-a-GCST90001556**	acute RITs	Effector Memory CD8+ T cell %T cell	0.0097	0.0059	0.1308
**ebi-a-GCST90001554**	acute RITs	Effector Memory CD8+ T cell Absolute Count	0.0051	0.0051	0.3316
**ukb-e-30210_CSA**	acute RITs	Eosinophill percentage	0.0096	0.0060	0.1231
**ukb-e-30300_AFR**	acute RITs	High light scatter reticulocyte count	-0.0135	0.0066	0.0513
**ebi-a-GCST90001441**	acute RITs	IgD+ CD24- B cell %lymphocyte	0.0009	0.0081	0.9154
**ebi-a-GCST90001431**	acute RITs	IgD+ CD38- B cell %lymphocyte	-0.0076	0.0115	0.5241
**ebi-a-GCST90001396**	acute RITs	IgD+ CD38- B cell Absolute Count	0.0041	0.0099	0.6850
**ebi-a-GCST90018962**	acute RITs	Lymphocyte count	0.0005	0.0010	0.6001
**ukb-d-30120_irnt**	acute RITs	Lymphocyte count	-0.0001	0.0011	0.9423
**ebi-a-GCST004627**	acute RITs	Lymphocyte counts	-0.0003	0.0015	0.8436
**bbj-a-44**	acute RITs	Neutrophil count	-0.0024	0.0037	0.5140
**ebi-a-GCST90002356**	acute RITs	Platelet count	-0.0008	0.0031	0.7925
**ieu-a-1008**	acute RITs	Platelet count	-0.0053	0.0029	0.0666
**ebi-a-GCST004601**	acute RITs	Red blood cell count	0.0018	0.0013	0.1838
**ebi-a-GCST90025964**	acute RITs	Red blood cell count	0.0001	0.0008	0.8768
**ebi-a-GCST90001578**	acute RITs	Transitional B cell %lymphocyte	-0.0034	0.0064	0.6002

**ebi-a-GCST90001577**	acute RITs	Transitional B cell Absolute Count	0.0028	0.0086	0.7451
**ukb-d-30000_irnt**	acute RITs	White blood cell (leukocyte) count	-0.0007	0.0011	0.5281
**ebi-a-GCST90002407**	acute RITs	White blood cell count	-0.0016	0.0010	0.0996
**ebi-a-GCST90018978**	acute RITs	White blood cell count	-0.0019	0.0010	0.0578
**ebi-a-GCST90025985**	acute RITs	White blood cell count	-0.0017	0.0010	0.0799
**ebi-a-GCST010779**	acute RITs	COVID-19 (hospitalized vs population) RELEASE 4	-0.0053	0.0050	0.2981
**ebi-a-GCST011082**	acute RITs	COVID-19 (hospitalized vs population) RELEASE 5	-0.0036	0.0047	0.4435
**ebi-a-GCST011084**	acute RITs	COVID-19 (hospitalized vs population) RELEASE 5	0.0021	0.0044	0.6409
**ukb-a-28**	acute RITs	Average weekly beer plus cider intake	-0.0080	0.0042	0.0621
**ukb-b-1707**	acute RITs	Average weekly spirits intake	-0.0005	0.0079	0.9464
**ukb-b-10054**	acute RITs	Cheese consumers	0.0108	0.0087	0.2304
**ukb-b-1814**	acute RITs	Cheesecake intake	-0.0044	0.0086	0.6141
**ukb-b-1160**	acute RITs	Chocolate-covered raisin intake	-0.0148	0.0078	0.0705
**ukb-b-8089**	acute RITs	Cooked vegetable intake	-0.0018	0.0045	0.6855
**ukb-b-5382**	acute RITs	Eggs in sandwiches intake	0.0018	0.0123	0.8872
**ukb-b-14351**	acute RITs	Grapefruit juice intake	-0.0048	0.0085	0.5770
**ukb-b-18079**	acute RITs	Low fat hard cheese intake	-0.0217	0.0112	0.0855
**ukb-d-1418_1**	acute RITs	Milk type used: Full cream	-0.0163	0.0081	0.0503
**ukb-d-1418_5**	acute RITs	Milk type used: Other type of milk	-0.0003	0.0128	0.9829
**ukb-e-6179_p3_CSA**	acute RITs	Mineral and other dietary supplements	-0.0091	0.0077	0.2671
**ukb-e-1389_AFR**	acute RITs	Pork intake	0.0076	0.0048	0.1293
**ukb-b-8006**	acute RITs	Poultry intake	-0.0087	0.0047	0.0642
**ukb-b-337**	acute RITs	Pure fruit/vegetable juice intake	-0.0094	0.0125	0.4641
**ukb-e-1478_AFR**	acute RITs	Salt added to food	-0.0030	0.0058	0.6134
**ukb-b-5847**	acute RITs	Sausage intake	-0.0236	0.0109	0.0540
**ukb-b-998**	acute RITs	Soya dessert intake	0.0045	0.0084	0.5997
**ukb-b-12067**	acute RITs	Sponge pudding intake	0.0077	0.0115	0.5110
**ukb-b-15984**	acute RITs	Thickness of butter/margarine spread on sliced bread: thin	-0.0104	0.0134	0.4512
**ukb-b-16523**	acute RITs	Type of meals eaten: Bought sandwiches	-0.0222	0.0380	0.5995
**ukb-b-6448**	acute RITs	Type of sliced bread eaten: mixed	-0.0062	0.0116	0.5988
**ukb-b-3828**	acute RITs	Types of spread used on bread/crackers: Normal fat polyunsaturated margarine on bread/crackers	-0.0116	0.0454	0.8144
**ukb-b-11725**	acute RITs	Types of spreads/sauces consumed: Tomato-based sauce	-0.0135	0.0293	0.6912
**ukb-b-3383**	acute RITs	Vegetarian sausages/burgers intake	0.0117	0.0081	0.1635
**ukb-b-5427**	acute RITs	White fish intake	0.0120	0.0134	0.4119
**ukb-b-2375**	acute RITs	Whole-wheat cereal intake	0.0082	0.0094	0.3949
**ukb-b-7753**	acute RITs	Yogurt intake	-0.0130	0.0127	0.3330

**ukb-d-M13_ADHCAPSULITIS**	acute RITs	Adhesive capsulitis of shoulder	-0.0088	0.0111	0.4355
**ebi-a-GCST90086041**	acute RITs	Allergic rhinitis	-0.0027	0.0035	0.4474
**ebi-a-GCST005529**	acute RITs	Ankylosing spondylitis	0.0034	0.0026	0.1911
**ieu-a-45**	acute RITs	Anorexia nervosa	0.0058	0.0037	0.1630
**ukb-d-KRA_PSY_ANYMENTAL**	acute RITs	Any mental disorder	-0.0016	0.0128	0.9037
**ebi-a-GCST90000026**	acute RITs	Appendicular lean mass	-0.0017	0.0011	0.1241
**bbj-a-86**	acute RITs	Arrhythmia	-0.0025	0.0024	0.2947
**bbj-a-71**	acute RITs	Atrial Fibrillation	0.0019	0.0032	0.5471
**ukb-b-8463**	acute RITs	Back pain for 3+ months	-0.0027	0.0105	0.7986
**ukb-e-41_CSA**	acute RITs	Bacterial infection NOS	-0.0103	0.0084	0.2497
**ukb-a-444**	acute RITs	Blood clot DVT bronchitis emphysema asthma rhinitis eczema allergy diagnosed by doctor: Emphysema/chronic bronchitis	0.0047	0.0084	0.5827
**ukb-e-6152_p1_AFR**	acute RITs	Blood clot, DVT, bronchitis, emphysema, asthma, rhinitis, eczema, allergy diagnosed by doctor	0.0048	0.0056	0.4025
**ebi-a-GCST90038652**	acute RITs	Bone disorder	-0.0098	0.0060	0.1073
**ebi-a-GCST90018591**	acute RITs	Cardiac valvular disease	0.0031	0.0040	0.4538
**ebi-a-GCST90018812**	acute RITs	Cardiomegaly	0.0132	0.0088	0.1477
**ukb-e-366_CSA**	acute RITs	Cataract	0.0055	0.0091	0.5575
**ieu-a-1058**	acute RITs	Celiac disease	0.0056	0.0027	0.0503
**ebi-a-GCST90018818**	acute RITs	Cholecystitis	0.0029	0.0030	0.3343
**ieu-b-4971**	acute RITs	Cholecystitits	0.0113	0.0062	0.0852
**ebi-a-GCST90013939**	acute RITs	Cholelithiasis gall stones (SPA correction)	0.0008	0.0021	0.7009
**ebi-a-GCST90013939**	acute RITs	Cholelithiasis gall stones (SPA correction)	0.0008	0.0021	0.7009
**ebi-a-GCST008026**	acute RITs	Chronic kidney disease	0.0088	0.0051	0.0947
**ebi-a-GCST90018809**	acute RITs	Chronic suppurative otitis media	0.0003	0.0069	0.9641
**ukb-e-851_CSA**	acute RITs	Complications of transplants and reattached limbs	-0.0152	0.0085	0.0970
**ukb-e-5264_AFR**	acute RITs	Corneal hysteresis (left)	-0.0097	0.0057	0.0983
**ebi-a-GCST004132**	acute RITs	Crohn's disease	0.0012	0.0019	0.5484
**ieu-a-30**	acute RITs	Crohn's disease	0.0017	0.0024	0.4908
**ukb-e-D64_CSA**	acute RITs	D64 Other anaemias	0.0095	0.0077	0.2481
**ukb-b-3145**	acute RITs	Diagnoses - main ICD10: C44.5 Skin of trunk	-0.0102	0.0385	0.8016
**ukb-d-C67**	acute RITs	Diagnoses - main ICD10: C67 Malignant neoplasm of bladder	-0.0027	0.0092	0.7672
**ukb-a-521**	acute RITs	Diagnoses - main ICD10: D12 Benign neoplasm of colon rectum anus and anal canal	0.0151	0.0074	0.0527
**ukb-d-D23**	acute RITs	Diagnoses - main ICD10: D23 Other benign neoplasms of skin	0.0012	0.0104	0.9103
**ukb-d-H33**	acute RITs	Diagnoses - main ICD10: H33 Retinal detachments and breaks	0.0006	0.0066	0.9311
**ukb-b-11771**	acute RITs	Diagnoses - main ICD10: K08.3 Retained dental root	-0.0777	0.2362	0.7976
**ukb-b-19354**	acute RITs	Diagnoses - main ICD10: K20 Oesophagitis	-0.0054	0.0269	0.8461

**ukb-b-11369**	acute RITs	Diagnoses - main ICD10: K40.2 Bilateral inguinal hernia, without obstruction or gangrene	0.0471	0.0295	0.1710
**ukb-b-19805**	acute RITs	Diagnoses - main ICD10: K62.1 Rectal polyp	0.0325	0.0153	0.0661
**ukb-b-8988**	acute RITs	Diagnoses - main ICD10: K80.1 Calculus of gallbladder with other cholecystitis	0.0116	0.0069	0.1075
**ukb-b-8988**	acute RITs	Diagnoses - main ICD10: K80.1 Calculus of gallbladder with other cholecystitis	0.0116	0.0069	0.1075
**ukb-d-K81**	acute RITs	Diagnoses - main ICD10: K81 Cholecystitis	0.0017	0.0071	0.8147
**ukb-d-K92**	acute RITs	Diagnoses - main ICD10: K92 Other diseases of digestive system	-0.0131	0.0098	0.2025
**ukb-d-L57**	acute RITs	Diagnoses - main ICD10: L57 Skin changes due to chronic exposure to nonionising radiation	-0.0121	0.0063	0.0644
**ukb-b-15003**	acute RITs	Diagnoses - main ICD10: L72.0 Epidermal cyst	0.0073	0.0319	0.8254
**ukb-b-7700**	acute RITs	Diagnoses - main ICD10: M17.1 Other primary gonarthrosis	-0.0224	0.0166	0.1980
**ukb-b-9694**	acute RITs	Diagnoses - main ICD10: M23.22 Derangement of meniscus due to old tear or injury (Posterior cruciate ligament or Posterior horn of medial meniscus)	-0.0023	0.0672	0.9746
**ukb-d-M70**	acute RITs	Diagnoses - main ICD10: M70 Soft tissue disorders related to use, overuse and pressure	-0.0038	0.0100	0.7096
**ukb-b-18372**	acute RITs	Diagnoses - main ICD10: N20.0 Calculus of kidney	0.0087	0.0119	0.4793
**ukb-b-18629**	acute RITs	Diagnoses - main ICD10: N20.1 Calculus of ureter	0.0293	0.0225	0.2194
**ukb-d-O80**	acute RITs	Diagnoses - main ICD10: O80 Single spontaneous delivery	-0.0170	0.0090	0.0786
**ukb-b-6608**	acute RITs	Diagnoses - main ICD10: R10.1 Pain localised to upper abdomen	0.0120	0.0127	0.3745
**ukb-b-17456**	acute RITs	Diagnoses - main ICD10: R10.3 Pain localised to other parts of lower abdomen	-0.0042	0.0202	0.8410
**ukb-a-584**	acute RITs	Diagnoses - main ICD10: R14 Flatulence and related conditions	0.0026	0.0090	0.7785
**ukb-d-S61**	acute RITs	Diagnoses - main ICD10: S61 Open wound of wrist and hand	-0.0043	0.0082	0.6028
**ukb-d-Z45**	acute RITs	Diagnoses - main ICD10: Z45 Adjustment and management of implanted device	0.0058	0.0097	0.5598
**ukb-b-16056**	acute RITs	Diagnoses - secondary ICD10: F10.1 Harmful use	-0.0405	0.0438	0.3972
**ukb-b-19651**	acute RITs	Diagnoses - secondary ICD10: F10.2 Dependence syndrome	0.0049	0.0523	0.9278
**ukb-b-16751**	acute RITs	Diagnoses - secondary ICD10: J44.9 Chronic obstructive pulmonary disease, unspecified	-0.0042	0.0157	0.7895
**ukb-b-11675**	acute RITs	Diagnoses - secondary ICD10: K66.0 Peritoneal adhesions	0.0101	0.2401	0.9692
**ukb-b-20233**	acute RITs	Diagnoses - secondary ICD10: M19.99 Arthrosis, unspecified (Site unspecified)	-0.0016	0.0339	0.9628
**ukb-b-6413**	acute RITs	Diagnoses - secondary ICD10: N73.6 Female pelvic peritoneal adhesions	0.0917	0.0763	0.2957
**ukb-b-10490**	acute RITs	Diagnoses - secondary ICD10: Z03.8 Observation for other suspected diseases and conditions	-0.0893	0.0441	0.0733
**ukb-b-2895**	acute RITs	Diagnoses - secondary ICD10: Z60.2 Living alone	0.0304	0.0572	0.6484
**ukb-b-10911**	acute RITs	Diagnoses - secondary ICD10: Z92.2 Personal history of long-term (current) use of other medicaments	0.0192	0.0111	0.1010
**ukb-d-IX_CIRCULATORY**	acute RITs	Diseases of the circulatory system	0.0026	0.0050	0.6036
**ukb-a-254**	acute RITs	Doctor diagnosed hayfever or allergic rhinitis	0.0080	0.0043	0.0673
**ukb-d-20429**	acute RITs	Easily tired during worst period of anxiety	0.0086	0.0092	0.3620
**ukb-e-F17_CSA**	acute RITs	F17 Mental and behavioral disorders due to use of tobacco	-0.0083	0.0080	0.3163
**ebi-a-GCST90018625**	acute RITs	Food allergy	-0.0158	0.0098	0.1449
**ebi-a-GCST90000514**	acute RITs	Gastroesophageal reflux disease	0.0016	0.0033	0.6266
**ebi-a-GCST90018635**	acute RITs	Hashimoto thyroiditis	0.0002	0.0058	0.9752
**ukb-e-2247_p1_AFR**	acute RITs	Hearing difficulty/problems	-0.0125	0.0059	0.0546

**ebi-a-GCST90038627**	acute RITs	Hepatitis	0.0132	0.0090	0.1573
**ukb-d-K11_HERNIA**	acute RITs	Hernia	-0.0056	0.0050	0.2716
**ieu-a-1169**	acute RITs	Hip osteoarthritis	0.0002	0.0168	0.9918
**ebi-a-GCST90018860**	acute RITs	Hyperthyroidism	0.0010	0.0043	0.8234
**ebi-a-GCST90018861**	acute RITs	Hypertrophic cardiomyopathy	0.0053	0.0069	0.4590
**ukb-e-I25_AFR**	acute RITs	I25 Chronic ischaemic heart disease	0.0040	0.0050	0.4265
**ebi-a-GCST003043**	acute RITs	Inflammatory bowel disease	0.0006	0.0017	0.7188
**ieu-a-292**	acute RITs	Inflammatory bowel disease	-0.0007	0.0018	0.7064
**ieu-a-294**	acute RITs	Inflammatory bowel disease	0.0008	0.0017	0.6522
**ebi-a-GCST90038626**	acute RITs	Irritable bowel syndrome	-0.0024	0.0072	0.7385
**bbj-a-129**	acute RITs	Ischemic stroke	-0.0013	0.0037	0.7263
**ukb-e-K21_CSA**	acute RITs	K21 Gastro-oesophageal reflux disease	-0.0072	0.0137	0.6135
**ukb-e-M17_CSA**	acute RITs	M17 Gonarthrosis [arthrosis of knee]	0.0023	0.0075	0.7679
**ukb-b-6458**	acute RITs	Mouth/teeth dental problems: Mouth ulcers	0.0006	0.0020	0.7448
**ukb-a-429**	acute RITs	Mouth/teeth dental problems: Painful gums	-0.0008	0.0125	0.9485
**ieu-b-18**	acute RITs	multiple sclerosis	0.0004	0.0015	0.7779
**ukb-e-N17_CSA**	acute RITs	N17 Acute renal failure	0.0041	0.0081	0.6212
**ukb-e-N18_AFR**	acute RITs	N18 Chronic renal failure	0.0024	0.0117	0.8484
**ebi-a-GCST006940**	acute RITs	Neurociticism	0.0021	0.0027	0.4383
**ukb-a-230**	acute RITs	Neuroticism score	0.0034	0.0033	0.3009
**ukb-a-81**	acute RITs	Non-cancer illness code self-reported: depression	-0.0120	0.0066	0.0760
**ukb-a-94**	acute RITs	Non-cancer illness code self-reported: hayfever/allergic rhinitis	-0.0041	0.0042	0.3412
**ukb-a-86**	acute RITs	Non-cancer illness code self-reported: joint disorder	0.0030	0.0080	0.7064
**ukb-e-20002_p95_CSA**	acute RITs	Non-cancer illness code, self-reported	-0.0031	0.0070	0.6672
**ukb-b-14027**	acute RITs	Non-cancer illness code, self-reported: chickenpox	-0.0533	0.0581	0.4107
**ukb-b-3044**	acute RITs	Non-cancer illness code, self-reported: colitis/not crohns or ulcerative colitis	0.0314	0.0493	0.5584
**ukb-b-14452**	acute RITs	Non-cancer illness code, self-reported: inguinal hernia	0.0164	0.0414	0.7089
**ukb-b-2592**	acute RITs	Non-cancer illness code, self-reported: irritable bowel syndrome	-0.0106	0.0107	0.3379
**ukb-b-17670**	acute RITs	Non-cancer illness code, self-reported: multiple sclerosis	-0.0015	0.0044	0.7480
**ukb-b-6633**	acute RITs	Non-cancer illness code, self-reported: other renal/kidney problem	0.0207	0.0335	0.5567
**ukb-b-14210**	acute RITs	Non-cancer illness code, self-reported: rectal or colon adenoma/polyps	-0.0647	0.2990	0.8644
**ukb-b-15749**	acute RITs	Non-cancer illness code, self-reported: transient ischaemic attack (tia)	-0.1114	0.0807	0.2611
**ebi-a-GCST007091**	acute RITs	Osteoarthritis (hip)	0.0017	0.0047	0.7138
**ukb-d-I9_ARTOTH**	acute RITs	Other diseases of arteries and capillaries	-0.0164	0.0084	0.0680
**ukb-e-596_CSA**	acute RITs	Other disorders of bladder	-0.0001	0.0127	0.9946
**ukb-e-569_AFR**	acute RITs	Other disorders of intestine	-0.0014	0.0063	0.8329

**ukb-e-306_CSA**	acute RITs	Other mental disorder	0.0001	0.0072	0.9867
**ukb-a-469**	acute RITs	Pain type(s) experienced in last month: Headache	-0.0054	0.0032	0.0968
**ukb-b-18596**	acute RITs	Pain type(s) experienced in last month: Neck or shoulder pain	0.0001	0.0051	0.9904
**ukb-b-9130**	acute RITs	Pain type(s) experienced in last month: None of the above	0.0046	0.0035	0.1910
**ukb-d-M13_DUPUTRYEN**	acute RITs	Palmar fascial fibromatosis [Dupuytren]	0.0038	0.0021	0.0771
**ebi-a-GCST90018897**	acute RITs	Periodontal disease	0.0013	0.0054	0.8137
**ebi-a-GCST90018679**	acute RITs	Pleurisy	-0.0018	0.0041	0.6705
**ebi-a-GCST90018683**	acute RITs	Pollinosis	0.0005	0.0089	0.9592
**ebi-a-GCST005581**	acute RITs	Primary biliary cirrhosis	0.0078	0.0039	0.0547
**ebi-a-GCST90013954**	acute RITs	Retinitis pigmentosa (SPA correction)	-0.0046	0.0134	0.7383
**ebi-a-GCST005538**	acute RITs	Sarcoidosis	-0.0050	0.0097	0.6147
**ukb-a-246**	acute RITs	Seen doctor (GP) for nerves anxiety tension or depression	-0.0001	0.0037	0.9861
**ebi-a-GCST90000255**	acute RITs	Severe COVID-19 infection with respiratory failure (analysis I)	-0.0019	0.0106	0.8612
**ebi-a-GCST90026414**	acute RITs	Severe insulin-resistant type 2 diabetes	0.0013	0.0156	0.9362
**ukb-d-L12_NONIONRADISKIN**	acute RITs	Skin changes due to chronic exposure to nonionizing radiation	-0.0121	0.0063	0.0644
**ebi-a-GCST003156**	acute RITs	Systemic lupus erythematosus	0.0035	0.0024	0.1515
**ebi-a-GCST90011866**	acute RITs	Systemic lupus erythematosus	0.0052	0.0032	0.1045
**ieu-a-815**	acute RITs	Systemic lupus erythematosus	-0.0161	0.0102	0.1532
**bbj-a-153**	acute RITs	Type 2 diabetes	0.0007	0.0016	0.6669
**ebi-a-GCST90018706**	acute RITs	Type 2 diabetes	-0.0003	0.0017	0.8392
**ebi-a-GCST003045**	acute RITs	Ulcerative colitis	0.0027	0.0021	0.1968
**ieu-a-968**	acute RITs	Ulcerative colitis	0.0008	0.0022	0.7257
**ieu-a-970**	acute RITs	Ulcerative colitis	0.0023	0.0020	0.2542
**ieu-b-5063**	acute RITs	URTI	0.0087	0.0094	0.3694
**ebi-a-GCST90018718**	acute RITs	Uveitis	0.0106	0.0057	0.1602
**ukb-e-20107_p11_CSA**	acute RITs	Illnesses of father	-0.0107	0.0085	0.2244
**ukb-a-201**	acute RITs	Illnesses of father: Heart disease	0.0043	0.0037	0.2486
**ukb-a-202**	acute RITs	Illnesses of father: None of the above (group 1)	0.0049	0.0067	0.4726
**ukb-b-15169**	acute RITs	Illnesses of father: None of the above (group 1)	-0.0051	0.0066	0.4494
**ukb-b-10415**	acute RITs	Illnesses of father: None of the above (group 2)	0.0063	0.0073	0.3923
**ukb-b-4024**	acute RITs	Illnesses of mother: Stroke	0.0062	0.0097	0.5316
**ukb-b-18042**	acute RITs	Illnesses of siblings: Diabetes	-0.0006	0.0036	0.8771
**ukb-b-10783**	acute RITs	Illnesses of siblings: None of the above (group 2)	0.0015	0.0064	0.8192
**ukb-a-304**	acute RITs	Number of children fathered	0.0044	0.0073	0.5547
**ebi-a-GCST006702**	acute RITs	Parental longevity (combined parental age at death)	0.0006	0.0048	0.9076
**ebi-a-GCST006699**	acute RITs	Parental longevity (mother's age at death)	-0.0044	0.0057	0.4427

**ebi-a-GCST006696**	acute RITs	Parental longevity (mother's attained age)	0.0061	0.0031	0.0543
**prot-a-2235**	acute RITs	[Pyruvate dehydrogenase (acetyl-transferring)] kinase isozyme 1, mitochondrial	-0.0093	0.0066	0.1884
**prot-a-3251**	acute RITs	14-3-3 protein zeta/delta	0.0089	0.0110	0.4294
**prot-a-1370**	acute RITs	15-hydroxyprostaglandin dehydrogenase [NAD(+)]	0.0060	0.0059	0.3211
**prot-a-11**	acute RITs	Acetyl-CoA carboxylase 2	-0.0140	0.0090	0.1391
**prot-a-105**	acute RITs	Acidic leucine-rich nuclear phosphoprotein 32 family member B	-0.0052	0.0092	0.5770
**prot-a-43**	acute RITs	Adhesion G-protein coupled receptor F1	-0.0157	0.0086	0.0848
**prot-a-47**	acute RITs	Alcohol dehydrogenase 4	-0.0011	0.0088	0.9016
**prot-a-1155**	acute RITs	Alpha-(1,3)-fucosyltransferase 9	0.0113	0.0065	0.1055
**ebi-a-GCST90006919**	acute RITs	Anti-herpes simplex virus 2 IgG seropositivity	0.0064	0.0092	0.4959
**ebi-a-GCST90006921**	acute RITs	Anti-polyomavirus 2 IgG seropositivity	-0.0067	0.0065	0.3191
**prot-a-180**	acute RITs	Arf-GAP with SH3 domain, ANK repeat and PH domain-containing protein 2	-0.0044	0.0052	0.4051
**prot-a-204**	acute RITs	Aurora kinase A	0.0008	0.0077	0.9169
**ebi-a-GCST90001829**	acute RITs	BAFF-R on B cell	0.0043	0.0046	0.3695
**ebi-a-GCST90001830**	acute RITs	BAFF-R on CD20- B cell	0.0192	0.0147	0.2618
**ebi-a-GCST90001702**	acute RITs	BAFF-R on CD24+ CD27+ B cell	0.0024	0.0051	0.6444
**ebi-a-GCST90001710**	acute RITs	BAFF-R on IgD- CD24- B cell	0.0013	0.0046	0.7868
**ebi-a-GCST90001711**	acute RITs	BAFF-R on IgD- CD27- B cell	0.0049	0.0055	0.3982
**ebi-a-GCST90001712**	acute RITs	BAFF-R on IgD- CD38- B cell	0.0045	0.0057	0.4482
**ebi-a-GCST90001713**	acute RITs	BAFF-R on IgD- CD38+ B cell	0.0069	0.0070	0.3488
**ebi-a-GCST90001719**	acute RITs	BAFF-R on IgD+ B cell	0.0006	0.0041	0.8883
**ebi-a-GCST90001704**	acute RITs	BAFF-R on IgD+ CD24- B cell	-0.0006	0.0042	0.8904
**ebi-a-GCST90001703**	acute RITs	BAFF-R on IgD+ CD24+ B cell	0.0068	0.0052	0.2179
**ebi-a-GCST90001705**	acute RITs	BAFF-R on IgD+ CD38- B cell	0.0002	0.0040	0.9596
**ebi-a-GCST90001708**	acute RITs	BAFF-R on IgD+ CD38+ B cell	-0.0028	0.0045	0.5328
**ebi-a-GCST90001709**	acute RITs	BAFF-R on IgD+ CD38dim B cell	0.0017	0.0050	0.7474
**ebi-a-GCST90001715**	acute RITs	BAFF-R on memory B cell	0.0041	0.0055	0.4811
**ebi-a-GCST90001716**	acute RITs	BAFF-R on naive-mature B cell	0.0039	0.0046	0.4117
**ebi-a-GCST90001718**	acute RITs	BAFF-R on switched memory B cell	0.0050	0.0059	0.4111
**ebi-a-GCST90001720**	acute RITs	BAFF-R on transitional B cell	-0.0013	0.0050	0.8016
**ebi-a-GCST90001717**	acute RITs	BAFF-R on unswitched memory B cell	0.0048	0.0045	0.3004
**prot-a-231**	acute RITs	Basal Cell Adhesion Molecule	-0.0083	0.0089	0.3587
**prot-a-218**	acute RITs	Beta-1,4-galactosyltransferase 3	-0.0075	0.0162	0.6555
**prot-a-800**	acute RITs	Beta-defensin 128	0.0007	0.0105	0.9453
**prot-a-801**	acute RITs	Beta-defensin 134	-0.0139	0.0086	0.1222
**prot-a-1396**	acute RITs	Bone sialoprotein 2	0.0062	0.0093	0.5141

**prot-a-2391**	acute RITs	Brain-specific serine protease 4	-0.0093	0.0079	0.2598
**prot-a-232**	acute RITs	Brevican core protein	-0.0082	0.0134	0.5475
**prot-a-2947**	acute RITs	Calcineurin B homologous protein 3	0.0205	0.0127	0.1310
**prot-a-2509**	acute RITs	Calcipressin-1	-0.0034	0.0109	0.7612
**prot-a-347**	acute RITs	Calcium/calmodulin-dependent protein kinase type 1D	-0.0122	0.0086	0.1726
**prot-a-2290**	acute RITs	Calcium-dependent phospholipase A2	-0.0085	0.0105	0.4274
**prot-a-557**	acute RITs	Carbohydrate sulfotransferase 5	-0.0048	0.0094	0.6160
**prot-a-332**	acute RITs	Carbonic anhydrase 5A, mitochondrial	-0.0151	0.0109	0.1904
**prot-a-327**	acute RITs	Carbonic anhydrase-related protein 10	-0.0164	0.0081	0.0578
**prot-a-649**	acute RITs	Carboxypeptidase Z	0.0084	0.0122	0.5010
**prot-a-697**	acute RITs	Casein kinase II 2-alpha:2-beta heterotetramer	0.0011	0.0092	0.9085
**prot-a-721**	acute RITs	Cathepsin F	-0.0004	0.0105	0.9677
**prot-a-723**	acute RITs	Cathepsin G	-0.0113	0.0086	0.2066
**prot-a-724**	acute RITs	Cathepsin H	-0.0031	0.0076	0.6844
**prot-a-728**	acute RITs	Cathepsin L2	0.0124	0.0089	0.1790
**prot-a-368**	acute RITs	Caveolin-2	-0.0013	0.0130	0.9201
**prot-a-398**	acute RITs	C-C motif chemokine 22	0.0146	0.0088	0.1137
**prot-a-409**	acute RITs	C-C motif chemokine 5	0.0009	0.0081	0.9093
**ebi-a-GCST90001449**	acute RITs	CD11c+ monocyte %monocyte	0.0144	0.0069	0.0564
**ebi-a-GCST90001748**	acute RITs	CD20 on IgD+ CD38- B cell	0.0149	0.0083	0.0864
**ebi-a-GCST90001751**	acute RITs	CD20 on IgD+ CD38+ B cell	0.0023	0.0045	0.6173
**ebi-a-GCST90001760**	acute RITs	CD20 on unswitched memory B cell	0.0108	0.0091	0.2522
**ebi-a-GCST90001760**	acute RITs	CD20 on unswitched memory B cell	0.0108	0.0091	0.2522
**ebi-a-GCST90001910**	acute RITs	CD45 on B cell	-0.0095	0.0046	0.0590
**prot-a-3080**	acute RITs	Cellular tumor antigen p53	-0.0132	0.0137	0.3504
**prot-a-432**	acute RITs	CMRF35-like molecule 8	0.0012	0.0052	0.8229
**prot-a-303**	acute RITs	Complement C1q tumor necrosis factor-related protein 1	0.0083	0.0056	0.1558
**prot-a-303**	acute RITs	Complement C1q tumor necrosis factor-related protein 1	0.0083	0.0056	0.1558
**prot-a-300**	acute RITs	Complement component 1 Q subcomponent-binding protein, mitochondrial	0.0169	0.0093	0.0821
**ebi-a-GCST90019439**	acute RITs	Complement factor B measurement	0.0004	0.0038	0.9091
**bbj-a-14**	acute RITs	C-reactive protein	-0.0010	0.0024	0.6846
**ieu-a-1015**	acute RITs	C-reactive protein	-0.0009	0.0116	0.9400
**prot-a-670**	acute RITs	C-reactive protein	-0.0047	0.0117	0.6919
**ieu-b-35**	acute RITs	C-Reactive protein level	-0.0009	0.0013	0.5106
**ebi-a-GCST90025959**	acute RITs	C-reactive protein levels	0.0001	0.0010	0.9362
**prot-a-579**	acute RITs	C-type lectin domain family 5 member A	0.0046	0.0055	0.4145

**ebi-a-GCST90002012**	acute RITs	CX3CR1 on CD14- CD16+ monocyte	0.0085	0.0045	0.0757
**ebi-a-GCST90001997**	acute RITs	CX3CR1 on CD14+ CD16- monocyte	0.0035	0.0055	0.5340
**ebi-a-GCST90001995**	acute RITs	CX3CR1 on monocyte	0.0067	0.0079	0.4040
**prot-a-3175**	acute RITs	Cytochrome b-c1 complex subunit 7	-0.0026	0.0107	0.8085
**prot-a-1086**	acute RITs	D-dimer	0.0037	0.0127	0.7765
**prot-a-813**	acute RITs	Desert hedgehog protein N-product	0.0044	0.0122	0.7229
**prot-a-824**	acute RITs	Dickkopf-like protein 1	-0.0015	0.0121	0.9050
**prot-a-586**	acute RITs	Dual specificity protein kinase CLK2	-0.0131	0.0074	0.0933
**prot-a-2179**	acute RITs	E3 ubiquitin-protein ligase parkin	0.0107	0.0104	0.3176
**prot-a-2528**	acute RITs	E3 ubiquitin-protein ligase rififylin	0.0216	0.0170	0.2297
**prot-a-3278**	acute RITs	E3 ubiquitin-protein ligase ZNRF3	0.0147	0.0163	0.3879
**prot-a-984**	acute RITs	Endoplasmic reticulum resident protein 29	-0.0131	0.0096	0.1960
**prot-a-902**	acute RITs	Ephrin-B1	-0.0015	0.0102	0.8866
**prot-a-907**	acute RITs	Ephrin-B3	-0.0166	0.0112	0.1660
**prot-a-2071**	acute RITs	Epididymal secretory protein E1	0.0014	0.0048	0.7780
**prot-a-1835**	acute RITs	Epididymis-specific alpha-mannosidase	-0.0010	0.0088	0.9089
**prot-a-977**	acute RITs	Epiregulin	-0.0197	0.0100	0.0643
**prot-a-308**	acute RITs	ES1 protein homolog, mitochondrial	0.0147	0.0092	0.1249
**prot-a-2892**	acute RITs	Estrogen sulfotransferase	0.0089	0.0066	0.1918
**prot-a-1001**	acute RITs	Exosome complex component CSL4	-0.0030	0.0098	0.7602
**prot-a-1003**	acute RITs	Exostosin-like 2	-0.0090	0.0098	0.3708
**prot-a-1019**	acute RITs	Fas apoptotic inhibitory molecule 3	-0.0036	0.0083	0.6680
**prot-a-1102**	acute RITs	Fibroblast growth factor receptor 2	-0.0010	0.0086	0.9124
**prot-a-1117**	acute RITs	Filamin-A	0.0001	0.0125	0.9932
**prot-a-567**	acute RITs	Galectin-10	0.0169	0.0083	0.0549
**prot-a-1295**	acute RITs	Glucoside xylosyltransferase 1	-0.0033	0.0057	0.5727
**prot-a-1285**	acute RITs	Glutathione S-transferase A4	-0.0139	0.0087	0.1235
**ebi-a-GCST90002248**	acute RITs	Glycated hemoglobin levels	-0.0012	0.0044	0.7848
**prot-a-1046**	acute RITs	Glycosaminoglycan xylosylkinase	-0.0012	0.0080	0.8868
**prot-a-1277**	acute RITs	Granulins	0.0053	0.0041	0.2056
**prot-a-1268**	acute RITs	GRB2-related adapter protein	0.0069	0.0123	0.5874
**prot-a-747**	acute RITs	Gro-beta/gamma	-0.0246	0.0117	0.0540
**prot-b-55**	acute RITs	growth differentiation factor 15	0.0124	0.0088	0.1789
**prot-a-1272**	acute RITs	Growth factor receptor-bound protein 7	-0.0150	0.0075	0.0588
**prot-a-1196**	acute RITs	Growth/differentiation factor 5	-0.0024	0.0041	0.5669
**prot-a-1197**	acute RITs	Growth/differentiation factor 9	-0.0002	0.0132	0.9882

**prot-a-1199**	acute RITs	GTP-binding protein GEM	-0.0012	0.0040	0.7641
**prot-a-1178**	acute RITs	Guanylate-binding protein 6	-0.0111	0.0095	0.2605
**prot-a-1387**	acute RITs	Heat shock 70 kDa protein 1-like	-0.0154	0.0077	0.0716
**prot-a-1310**	acute RITs	Hepatitis A virus cellular receptor 1	0.0026	0.0066	0.6976
**prot-a-1366**	acute RITs	Heterogeneous nuclear ribonucleoprotein K	0.0013	0.0105	0.9044
**prot-a-2224**	acute RITs	High affinity cGMP-specific 3',5'-cyclic phosphodiesterase 9A	0.0096	0.0096	0.3276
**prot-a-2121**	acute RITs	High affinity nerve growth factor receptor	-0.0075	0.0153	0.6338
**prot-a-1338**	acute RITs	Histidine triad nucleotide-binding protein 1	-0.0049	0.0125	0.7018
**prot-a-1607**	acute RITs	Histone acetyltransferase KAT6A	-0.0177	0.0103	0.1139
**prot-a-1304**	acute RITs	Histone H2A.z	0.0279	0.0137	0.0630
**ebi-a-GCST90002116**	acute RITs	HLA DR on B cell	0.0006	0.0060	0.9238
**prot-a-1393**	acute RITs	Hyaluronidase-1	0.0027	0.0114	0.8140
**prot-a-1071**	acute RITs	Immunoglobulin alpha Fc receptor	0.0274	0.0122	0.0662
**prot-a-1576**	acute RITs	Immunoglobulin superfamily containing leucine-rich repeat protein 2	0.0049	0.0064	0.4546
**prot-a-2141**	acute RITs	Inositol polyphosphate 5-phosphatase OCRL-1	-0.0031	0.0078	0.6970
**prot-a-1593**	acute RITs	Inositol-trisphosphate 3-kinase A	-0.0115	0.0115	0.3423
**prot-a-1435**	acute RITs	Interferon lambda-1	-0.0101	0.0130	0.4530
**prot-a-1547**	acute RITs	Interleukin enhancer-binding factor 3	0.0248	0.0120	0.0619
**ebi-a-GCST004444**	acute RITs	Interleukin-10 levels	-0.0037	0.0055	0.5193
**prot-a-1479**	acute RITs	Interleukin-16	-0.0061	0.0058	0.3068
**ebi-a-GCST004448**	acute RITs	Interleukin-1-beta levels	0.0060	0.0169	0.7447
**prot-a-1517**	acute RITs	Interleukin-27 receptor subunit alpha	0.0031	0.0083	0.7139
**prot-a-1524**	acute RITs	Interleukin-34	-0.0067	0.0083	0.4258
**prot-a-1528**	acute RITs	Interleukin-36 gamma	0.0067	0.0099	0.5050
**prot-a-1533**	acute RITs	Interleukin-4 receptor subunit alpha	0.0085	0.0100	0.4121
**prot-a-1535**	acute RITs	Interleukin-5	0.0054	0.0097	0.5878
**prot-a-1544**	acute RITs	Interleukin-7 receptor subunit alpha	0.0036	0.0072	0.6232
**prot-a-1595**	acute RITs	Intersectin-1	-0.0140	0.0113	0.2315
**prot-a-81**	acute RITs	Intestinal-type alkaline phosphatase	0.0091	0.0101	0.3822
**ebi-a-GCST90012012**	acute RITs	kallikrein-11 levels	-0.0044	0.0035	0.2232
**prot-a-1664**	acute RITs	Kallikrein-5	-0.0013	0.0129	0.9235
**prot-a-1640**	acute RITs	Killer cell immunoglobulin-like receptor 2DL2	-0.0112	0.0063	0.0961
**prot-a-1635**	acute RITs	Kinesin-like protein KIF16B	-0.0112	0.0076	0.1561
**prot-b-20**	acute RITs	KIT ligand	0.0005	0.0044	0.9170
**prot-a-2825**	acute RITs	Kunitz-type protease inhibitor 3	-0.0033	0.0055	0.5571
**prot-a-2825**	acute RITs	Kunitz-type protease inhibitor 3	-0.0033	0.0055	0.5571

**prot-a-1714**	acute RITs	Lactase-like protein	0.0000	0.0145	0.9983
**prot-a-1797**	acute RITs	Leucine-rich repeat transmembrane neuronal protein 4	0.0193	0.0095	0.0579
**prot-a-1076**	acute RITs	Low affinity immunoglobulin gamma Fc region receptor III-B	0.0028	0.0063	0.6602
**prot-a-2666**	acute RITs	L-Selectin	0.0025	0.0046	0.5970
**prot-a-2666**	acute RITs	L-Selectin	0.0025	0.0046	0.5970
**prot-a-1735**	acute RITs	Lutropin subunit beta	0.0019	0.0079	0.8170
**prot-a-454**	acute RITs	Lymphocyte function-associated antigen 3	-0.0055	0.0149	0.7219
**prot-a-3236**	acute RITs	Lymphotactin	0.0138	0.0074	0.0837
**prot-a-1912**	acute RITs	Macrophage metalloelastase	-0.0054	0.0063	0.4079
**prot-a-1931**	acute RITs	MAGUK p55 subfamily member 6	-0.0066	0.0107	0.5455
**prot-a-1854**	acute RITs	MAP kinase-activated protein kinase 5	-0.0028	0.0049	0.5714
**prot-a-1916**	acute RITs	Matrix metalloproteinase-16	-0.0081	0.0098	0.4258
**prot-a-1896**	acute RITs	Melanoma-derived growth regulatory protein	0.0024	0.0037	0.5246
**prot-a-1908**	acute RITs	Membrane metallo-endopeptidase-like 1	0.0113	0.0081	0.1777
**ebi-a-GCST90006924**	acute RITs	Merkel cell polyomavirus VP1 antibody levels	0.0004	0.0093	0.9677
**prot-a-1052**	acute RITs	MIP18 family protein FAM96A	-0.0038	0.0127	0.7719
**prot-a-5**	acute RITs	Monoacylglycerol lipase ABHD12	0.0059	0.0092	0.5327
**prot-b-75**	acute RITs	mucin 16, cell surface associated	0.0083	0.0079	0.3180
**prot-b-75**	acute RITs	mucin 16, cell surface associated	0.0083	0.0079	0.3180
**prot-a-1967**	acute RITs	Mucin-1	-0.0042	0.0059	0.4854
**prot-a-1995**	acute RITs	N-acetyl-D-glucosamine kinase	-0.0001	0.0064	0.9830
**prot-a-2057**	acute RITs	NACHT, LRR and PYD domains-containing protein 4	0.0160	0.0145	0.2866
**prot-a-2026**	acute RITs	NADH dehydrogenase [ubiquinone] flavoprotein 2, mitochondrial	0.0010	0.0162	0.9530
**prot-a-2081**	acute RITs	Neuronal pentraxin-2	-0.0208	0.0100	0.0551
**prot-a-345**	acute RITs	Neuron-specific vesicular protein calcyon	-0.0021	0.0092	0.8236
**prot-a-2099**	acute RITs	Neuropilin-1	0.0150	0.0085	0.0901
**prot-a-2050**	acute RITs	Nidogen-2	0.0007	0.0069	0.9175
**prot-a-1674**	acute RITs	NKG2-D type II integral membrane protein	0.0061	0.0102	0.5575
**prot-a-1872**	acute RITs	Nuclear protein MDM1	-0.0011	0.0138	0.9377
**prot-a-2086**	acute RITs	Nuclear receptor subfamily 1 group D member 2	0.0158	0.0086	0.0820
**prot-a-2093**	acute RITs	Nuclear receptor-binding protein	-0.0039	0.0076	0.6146
**prot-a-2150**	acute RITs	Oligophrenin-1	-0.0098	0.0096	0.3170
**prot-a-2155**	acute RITs	Oncostatin-M	-0.0163	0.0085	0.0702
**prot-a-2272**	acute RITs	Paired immunoglobulin-like type 2 receptor alpha	0.0075	0.0121	0.5426
**prot-a-2344**	acute RITs	Peptidyl-prolyl cis-trans isomerase-like 2	-0.0073	0.0116	0.5373
**prot-a-2425**	acute RITs	Phosphatidylinositol 3,4,5-trisphosphate 3-phosphatase and dual-specificity protein phosphatase PTEN	0.0010	0.0091	0.9115

**prot-a-2294**	acute RITs	Phospholipase B-like 1	0.0012	0.0115	0.9204
**prot-a-2698**	acute RITs	Pigment epithelium-derived factor	-0.0003	0.0063	0.9606
**prot-a-2299**	acute RITs	Pleckstrin homology domain-containing family A member 1	-0.0026	0.0096	0.7933
**prot-a-1171**	acute RITs	Polypeptide N-acetylgalactosaminyltransferase 2	0.0000	0.0116	0.9984
**prot-a-1613**	acute RITs	Potassium voltage-gated channel subfamily E regulatory beta subunit 5	0.0005	0.0087	0.9554
**prot-a-1615**	acute RITs	Potassium voltage-gated channel subfamily G member 4	-0.0121	0.0098	0.2313
**prot-a-2209**	acute RITs	Procollagen C-endopeptidase enhancer 1	0.0167	0.0081	0.0535
**prot-a-2409**	acute RITs	Proteasome subunit alpha type-1	0.0026	0.0115	0.8237
**prot-a-827**	acute RITs	Protein delta homolog 1	0.0096	0.0060	0.1281
**prot-a-810**	acute RITs	Protein DGCR6	-0.0209	0.0104	0.0583
**prot-a-2232**	acute RITs	Protein disulfide-isomerase A3	0.0037	0.0093	0.6953
**prot-a-2233**	acute RITs	Protein disulfide-isomerase A5	0.0032	0.0062	0.6093
**prot-a-1027**	acute RITs	Protein FAM163A	0.0006	0.0070	0.9283
**prot-a-1049**	acute RITs	Protein FAM3B	0.0060	0.0089	0.5133
**prot-a-2619**	acute RITs	Protein S100-A5	-0.0041	0.0050	0.4205
**prot-a-3093**	acute RITs	Protein-tyrosine sulfotransferase 2	0.0004	0.0086	0.9600
**prot-a-3093**	acute RITs	Protein-tyrosine sulfotransferase 2	0.0004	0.0086	0.9600
**prot-a-2203**	acute RITs	Protocadherin beta-1	-0.0160	0.0085	0.0769
**prot-a-2207**	acute RITs	Protocadherin gamma-C5	0.0161	0.0093	0.0983
**prot-a-2191**	acute RITs	Pterin-4-alpha-carbinolamine dehydratase	0.0045	0.0094	0.6369
**prot-a-2711**	acute RITs	Pulmonary surfactant-associated protein C	-0.0060	0.0064	0.3593
**prot-a-2477**	acute RITs	Ras-related protein Rab-39B	-0.0004	0.0106	0.9694
**prot-a-2444**	acute RITs	Receptor-type tyrosine-protein phosphatase H	0.0146	0.0083	0.0977
**prot-a-2524**	acute RITs	Resistin	0.0034	0.0075	0.6523
**prot-a-2497**	acute RITs	Retinoblastoma-binding protein 5	-0.0123	0.0126	0.3505
**prot-a-2602**	acute RITs	Ribonucleoside-diphosphate reductase subunit M2 B	-0.0139	0.0141	0.3434
**prot-a-1945**	acute RITs	Ribosome-recycling factor, mitochondrial	0.0082	0.0076	0.2944
**prot-a-2584**	acute RITs	Ribulose-phosphate 3-epimerase	0.0053	0.0114	0.6516
**prot-a-2569**	acute RITs	RING finger protein 215	-0.0148	0.0081	0.0874
**prot-a-2575**	acute RITs	rRNA methyltransferase 3, mitochondrial	-0.0070	0.0044	0.1270
**prot-a-2641**	acute RITs	Scavenger receptor class F member 2	0.0071	0.0095	0.4683
**prot-a-2642**	acute RITs	Scavenger receptor class F member 2	-0.0011	0.0086	0.9022
**prot-a-2709**	acute RITs	Secreted frizzled-related protein 1	0.0050	0.0102	0.6321
**prot-a-2669**	acute RITs	Semaphorin-3A	-0.0108	0.0066	0.1180
**prot-a-2671**	acute RITs	Semaphorin-3C	0.0112	0.0119	0.3689
**prot-a-2838**	acute RITs	Serine/arginine-rich splicing factor 6	-0.0204	0.0115	0.1049

**prot-a-2170**	acute RITs	Serine/threonine-protein kinase PAK 4	0.0042	0.0108	0.7050
**prot-a-2690**	acute RITs	Serpin A12	-0.0042	0.0092	0.6477
**prot-a-2730**	acute RITs	Sialic acid-binding Ig-like lectin 8	-0.0004	0.0060	0.9484
**prot-a-2655**	acute RITs	Signal peptide, CUB and EGF-like domain-containing protein 1	-0.0059	0.0153	0.7070
**prot-a-2775**	acute RITs	Single-pass membrane and coiled-coil domain-containing protein 2	0.0023	0.0112	0.8390
**prot-a-2714**	acute RITs	Small glutamine-rich tetratricopeptide repeat-containing protein alpha	0.0081	0.0093	0.3937
**prot-a-2763**	acute RITs	Sodium- and chloride-dependent glycine transporter 1	0.0280	0.0148	0.0849
**prot-a-200**	acute RITs	Sodium/potassium-transporting ATPase subunit beta-2	0.0061	0.0087	0.4941
**prot-a-352**	acute RITs	Soluble calcium-activated nucleotidase 1	-0.0155	0.0100	0.1411
**prot-a-2795**	acute RITs	Sorting nexin-7	-0.0080	0.0080	0.3320
**prot-a-2804**	acute RITs	Sperm acrosome membrane-associated protein 3	0.0120	0.0073	0.1183
**prot-b-68**	acute RITs	spondin 1	0.0051	0.0066	0.5218
**ebi-a-GCST90002074**	acute RITs	SSC-A on CD14+ monocyte	-0.0032	0.0046	0.4877
**ebi-a-GCST90002081**	acute RITs	SSC-A on CD4+ T cell	-0.0100	0.0083	0.2463
**ebi-a-GCST90002079**	acute RITs	SSC-A on lymphocyte	-0.0303	0.0176	0.1059
**ebi-a-GCST90019393**	acute RITs	Stromal cell-derived factor 2 measurement	-0.0113	0.0055	0.0597
**prot-a-2468**	acute RITs	Sulfhydryl oxidase 1	-0.0017	0.0100	0.8660
**prot-a-2894**	acute RITs	Sulfotransferase 4A1	0.0058	0.0101	0.5752
**prot-a-2901**	acute RITs	Sushi domain-containing protein 1	0.0111	0.0098	0.2708
**prot-a-2885**	acute RITs	Syntaxin-1A	0.0132	0.0075	0.0945
**prot-a-2933**	acute RITs	TATA-box-binding protein	0.0064	0.0134	0.6370
**prot-a-3068**	acute RITs	Tenascin-R	0.0044	0.0080	0.5895
**prot-a-3084**	acute RITs	Thiamin pyrophosphokinase 1	-0.0104	0.0101	0.3210
**prot-a-3102**	acute RITs	Thyrotropin-releasing hormone	-0.0023	0.0098	0.8164
**prot-a-3076**	acute RITs	Torsin-1A-interacting protein 1	-0.0081	0.0063	0.2198
**prot-a-3115**	acute RITs	TPA-induced transmembrane protein	-0.0087	0.0148	0.5700
**prot-a-2963**	acute RITs	Transforming growth factor beta-1-induced transcript 1 protein	0.0070	0.0147	0.6371
**prot-a-918**	acute RITs	Translation initiation factor eIF-2B subunit alpha	-0.0099	0.0109	0.3806
**prot-a-2844**	acute RITs	Translocon-associated protein subunit alpha	-0.0210	0.0105	0.0682
**prot-a-2992**	acute RITs	Transmembrane and coiled-coil domain-containing protein 5A	-0.0180	0.0099	0.0807
**prot-a-2993**	acute RITs	Transmembrane emp24 domain-containing protein 10	0.0095	0.0053	0.0936
**prot-a-285**	acute RITs	Transmembrane protein C16orf54	0.0000	0.0096	0.9997
**prot-a-1883**	acute RITs	tRNA (guanine-N(7)-)-methyltransferase	-0.0027	0.0107	0.8067
**prot-a-3029**	acute RITs	Tumor necrosis factor	0.0186	0.0100	0.0844
**prot-a-3059**	acute RITs	Tumor necrosis factor ligand superfamily member 15	0.0003	0.0084	0.9706
**prot-a-3128**	acute RITs	TYMS opposite strand protein	0.0006	0.0139	0.9658

**prot-a-3129**	acute RITs	Tyrosine-protein kinase receptor TYRO3	0.0158	0.0089	0.0921
**prot-a-2582**	acute RITs	Tyrosine-protein kinase transmembrane receptor ROR2	0.0172	0.0093	0.0773
**prot-a-598**	acute RITs	UMP-CMP kinase	0.0143	0.0103	0.1859
**prot-a-295**	acute RITs	Uncharacterized protein C1orf115	-0.0036	0.0095	0.7086
**prot-a-311**	acute RITs	Uncharacterized protein C2orf66	0.0140	0.0070	0.0628
**prot-a-3155**	acute RITs	UTP--glucose-1-phosphate uridylyltransferase	-0.0002	0.0133	0.9873
**prot-a-3197**	acute RITs	Vascular endothelial growth factor A, isoform 121	-0.0059	0.0041	0.1733
**prot-a-3197**	acute RITs	Vascular endothelial growth factor A, isoform 121	-0.0059	0.0041	0.1733
**ebi-a-GCST004422**	acute RITs	Vascular endothelial growth factor levels	-0.0042	0.0051	0.4286
**ebi-a-GCST90011995**	acute RITs	Vascular endothelial growth factor levels	-0.0059	0.0037	0.1337
**ebi-a-GCST90011995**	acute RITs	Vascular endothelial growth factor levels	-0.0059	0.0037	0.1337
**ebi-a-GCST90011995**	acute RITs	Vascular endothelial growth factor levels	-0.0059	0.0037	0.1337
**prot-a-3212**	acute RITs	Vesicle transport through interaction with t-SNAREs homolog 1A	0.0011	0.0099	0.9168
**prot-a-1179**	acute RITs	Vitamin D-binding protein	-0.0032	0.0123	0.7948
**prot-a-3230**	acute RITs	Wnt inhibitory factor 1	0.0127	0.0104	0.2391
**prot-a-3231**	acute RITs	WNT1-inducible-signaling pathway protein 1	-0.0090	0.0048	0.0775
**prot-a-3276**	acute RITs	Zinc finger protein 774	0.0126	0.0088	0.1629
**prot-a-208**	acute RITs	Zinc-alpha-2-glycoprotein	-0.0165	0.0085	0.0712
**ukb-a-406**	acute RITs	Current employment status: Looking after home and/or family	0.0111	0.0097	0.2708
**ukb-d-22601_41123205**	acute RITs	Job coding: civil service administrative officer or assistant or clerk, tax collector, prison service clerk, law courts clerk	-0.0076	0.0078	0.3444
**ukb-d-22601_35393271**	acute RITs	Job coding: management information officer, conference/events co-ordinator/organiser, exhibition officer, work study engineer/officer/analyst, contract adviser/agent, election agent, business system analyst	0.0011	0.0088	0.9036
**ukb-d-22601_23193405**	acute RITs	Job coding: other teaching professional including private tutor, tefl (teaching english as a foreign language) teacher; tutor at adult education centre, etc.	0.0043	0.0120	0.7263
**ukb-d-22601_23213026**	acute RITs	Job coding: scientific researcher, scientific officer, medical research associate, experimental officer	-0.0155	0.0088	0.0934
**ukb-d-22601_23163403**	acute RITs	Job coding: special needs teaching professional (including head teacher)	-0.0053	0.0119	0.6595
**ukb-e-826_AFR**	acute RITs	Job involves shift work	-0.0007	0.0063	0.9074
**ukb-d-22617_1151**	acute RITs	Job SOC coding: Financial institution managers	-0.0112	0.0094	0.2465
**ukb-d-22617_2321**	acute RITs	Job SOC coding: Scientific researchers	-0.0115	0.0088	0.2041
**ukb-d-22617_3231**	acute RITs	Job SOC coding: Youth and community workers	-0.0053	0.0098	0.5975
**ukb-b-18099**	acute RITs	Qualifications: O levels/GCSEs or equivalent	-0.0008	0.0043	0.8513
**ukb-a-368**	acute RITs	Work/job satisfaction	0.0121	0.0082	0.1548
**ebi-a-GCST90013919**	acute RITs	Cholesterol lowering medication use (UKB data field 6177_1) (Firth correction)	-0.0023	0.0021	0.2781
**ebi-a-GCST90013969**	acute RITs	Cholesterol lowering medication use (UKB data field 6177_1) (SPA correction)	-0.0027	0.0021	0.2149
**ukb-a-488**	acute RITs	Medication for cholesterol blood pressure or diabetes: Cholesterol lowering medication	0.0009	0.0027	0.7398
**ukb-b-11740**	acute RITs	Medication for cholesterol, blood pressure or diabetes: Cholesterol lowering medication	0.0003	0.0021	0.9039
**ukb-e-6153_p2_AFR**	acute RITs	Medication for cholesterol, blood pressure, diabetes, or take exogenous hormones	0.0059	0.0053	0.2776

**ebi-a-GCST90018984**	acute RITs	Medication use (antihypertensives)	-0.0007	0.0047	0.8884
**ebi-a-GCST90018767**	acute RITs	Medication use (calcium channel blockers)	0.0004	0.0043	0.9208
**ebi-a-GCST90018985**	acute RITs	Medication use (diuretics)	0.0008	0.0021	0.7225
**ebi-a-GCST90018993**	acute RITs	Medication use (drugs affecting bone structure and mineralization)	-0.0044	0.0048	0.3580
**ukb-b-12753**	acute RITs	Recent medication for hayfever or allergic rhinitis	-0.0002	0.0118	0.9858
**ukb-b-15918**	acute RITs	Treatment speciality of consultant (recoded): Dermatology	-0.0036	0.0084	0.6740
**ukb-b-3255**	acute RITs	Treatment speciality of consultant (recoded): Upper gastrointestinal surgery	0.0691	0.0680	0.3560
**ukb-e-20003_p118_AFR**	acute RITs	Treatment/medication code	-0.0089	0.0111	0.4335
**ukb-b-9207**	acute RITs	Treatment/medication code: amlodipine	0.0019	0.0043	0.6514
**ukb-b-11862**	acute RITs	Treatment/medication code: beconase 50micrograms nasal spray	-0.0012	0.0215	0.9567
**ukb-b-7385**	acute RITs	Treatment/medication code: codeine	0.0446	0.0427	0.3549
**ukb-b-8668**	acute RITs	Treatment/medication code: doxazosin	-0.0043	0.0123	0.7264
**ukb-a-162**	acute RITs	Treatment/medication code: perindopril	-0.0049	0.0094	0.6109
**ukb-a-141**	acute RITs	Treatment/medication code: prednisolone	-0.0220	0.0112	0.0703
**ukb-b-11895**	acute RITs	Treatment/medication code: ramipril	0.0008	0.0055	0.8888
**ukb-a-130**	acute RITs	Treatment/medication code: senna	-0.0014	0.0077	0.8523
**ukb-b-16956**	acute RITs	Treatment/medication code: thyroxine sodium	-0.0046	0.0125	0.7213
**ukb-a-120**	acute RITs	Treatment/medication code: tranexamic acid	0.0041	0.0086	0.6366
**ukb-b-17616**	acute RITs	Treatment/medication code: xalatan 0.005% eye drops	0.0084	0.0269	0.7608
**ukb-e-6155_p5_AFR**	acute RITs	Vitamin and mineral supplements	-0.0161	0.0179	0.3918
**ukb-e-6155_p8_AFR**	acute RITs	Vitamin and mineral supplements	0.0060	0.0075	0.4407
**ukb-a-464**	acute RITs	Vitamin and mineral supplements: Multivitamins +/- minerals	-0.0019	0.0063	0.7644
**ukb-b-19550**	acute RITs	Vitamin and/or mineral supplement use: Calcium	-0.0252	0.0558	0.6749
**ukb-b-18593**	acute RITs	Vitamin D	-0.0098	0.0073	0.1938
**ukb-e-Z51_AFR**	acute RITs	Z51 Other medical care	0.0030	0.0092	0.7517
**met-c-846**	acute RITs	3-hydroxybutyrate	0.0028	0.0087	0.7476
**met-d-bOHbutyrate**	acute RITs	3-Hydroxybutyrate	0.0019	0.0045	0.6852
**met-a-311**	acute RITs	3-hydroxybutyrate (BHBA)	-0.0057	0.0073	0.4590
**met-a-365**	acute RITs	3-methoxytyrosine	0.0158	0.0095	0.1167
**ieu-a-1**	acute RITs	Adiponectin	-0.0020	0.0029	0.5014
**met-a-537**	acute RITs	Adrenate (22:4n6)	-0.0010	0.0114	0.9340
**bbj-a-9**	acute RITs	Albumin	-0.0007	0.0041	0.8657
**met-a-616**	acute RITs	Alpha-hydroxyisovalerate	0.0093	0.0086	0.3037
**met-d-ApoA1**	acute RITs	Apolipoprotein A1	-0.0002	0.0015	0.8769
**ieu-b-107**	acute RITs	apolipoprotein A-I	-0.0015	0.0008	0.0581
**met-c-842**	acute RITs	Apolipoprotein A-I	0.0036	0.0049	0.4708

**ieu-b-108**	acute RITs	apolipoprotein B	-0.0012	0.0010	0.1967
**ieu-b-108**	acute RITs	apolipoprotein B	-0.0012	0.0010	0.1967
**met-d-ApoB**	acute RITs	Apolipoprotein B	0.0026	0.0017	0.1183
**ebi-a-GCST90092809**	acute RITs	Apolipoprotein B levels	0.0026	0.0017	0.1266
**prot-a-134**	acute RITs	Apolipoprotein L1	0.0096	0.0056	0.1043
**met-a-638**	acute RITs	Asparagine	0.0061	0.0033	0.0754
**ieu-b-4869**	acute RITs	Bioavailable Testosterone	0.0006	0.0016	0.6842
**ebi-a-GCST90012102**	acute RITs	Bioavailable testosterone levels	0.0007	0.0014	0.6023
**ebi-a-GCST90012104**	acute RITs	Bioavailable testosterone levels	0.0000	0.0017	0.9879
**ebi-a-GCST90012104**	acute RITs	Bioavailable testosterone levels	0.0000	0.0017	0.9879
**bbj-a-10**	acute RITs	Blood sugar	0.0021	0.0054	0.7044
**ebi-a-GCST90018948**	acute RITs	Blood urea nitrogen levels	-0.0020	0.0013	0.1182
**ebi-a-GCST90014002**	acute RITs	C reactive protein levels (UKB data field 30710)	0.0015	0.0009	0.1037
**met-a-481**	acute RITs	Caprylate (8:0)	-0.0006	0.0042	0.8890
**met-c-887**	acute RITs	Cholesterol esters in large VLDL	0.0047	0.0048	0.3365
**met-c-899**	acute RITs	Cholesterol esters in medium HDL	-0.0113	0.0071	0.1278
**met-c-899**	acute RITs	Cholesterol esters in medium HDL	-0.0113	0.0071	0.1278
**met-d-M_HDL_C**	acute RITs	Cholesterol in medium HDL	-0.0003	0.0016	0.8407
**met-d-M_VLDL_C**	acute RITs	Cholesterol in medium VLDL	0.0012	0.0017	0.4818
**met-d-S_LDL_C**	acute RITs	Cholesterol in small LDL	0.0003	0.0017	0.8772
**ebi-a-GCST90014000**	acute RITs	Cholesterol levels (UKB data field 30690)	0.0006	0.0011	0.6107
**ebi-a-GCST90092956**	acute RITs	Cholesterol levels in small LDL	0.0012	0.0016	0.4490
**met-d-M_HDL_C_pct**	acute RITs	Cholesterol to total lipids ratio in medium HDL	-0.0018	0.0014	0.2071
**ebi-a-GCST90092918**	acute RITs	Cholesteryl ester levels in medium VLDL	0.0002	0.0018	0.9078
**ebi-a-GCST90060634**	acute RITs	Cholesteryl ester(18:3)_[M+NH4]1+ levels	0.0030	0.0066	0.6530
**met-d-HDL_CE**	acute RITs	Cholesteryl esters in HDL	-0.0021	0.0013	0.1067
**met-d-L_LDL_CE**	acute RITs	Cholesteryl esters in large LDL	0.0003	0.0016	0.8695
**met-d-LDL_CE**	acute RITs	Cholesteryl esters in LDL	0.0001	0.0017	0.9715
**met-d-M_HDL_CE**	acute RITs	Cholesteryl esters in medium HDL	0.0001	0.0016	0.9751
**met-d-M_VLDL_CE**	acute RITs	Cholesteryl esters in medium VLDL	-0.0002	0.0019	0.9228
**met-d-S_HDL_CE**	acute RITs	Cholesteryl esters in small HDL	-0.0006	0.0018	0.7549
**met-d-IDL_CE_pct**	acute RITs	Cholesteryl esters to total lipids ratio in IDL	-0.0018	0.0014	0.2127
**met-d-L_HDL_CE_pct**	acute RITs	Cholesteryl esters to total lipids ratio in large HDL	-0.0010	0.0013	0.4435
**met-d-L_HDL_CE_pct**	acute RITs	Cholesteryl esters to total lipids ratio in large HDL	-0.0010	0.0013	0.4435
**met-d-M_HDL_CE_pct**	acute RITs	Cholesteryl esters to total lipids ratio in medium HDL	-0.0009	0.0014	0.5131
**ebi-a-GCST90007307**	acute RITs	circulating leptin levels	-0.0034	0.0098	0.7342

**ebi-a-GCST90007309**	acute RITs	circulating leptin levels	0.0145	0.0232	0.5594
**ebi-a-GCST90007310**	acute RITs	circulating leptin levels	0.0056	0.0132	0.6866
**ebi-a-GCST90007312**	acute RITs	circulating leptin levels	0.0012	0.0452	0.9791
**ebi-a-GCST90007313**	acute RITs	circulating leptin levels	0.0051	0.0187	0.7973
**ebi-a-GCST90007316**	acute RITs	circulating leptin levels	0.0050	0.0183	0.7973
**ebi-a-GCST90007319**	acute RITs	circulating leptin levels adjusted for BMI	0.0003	0.0108	0.9771
**ebi-a-GCST90007320**	acute RITs	circulating leptin levels adjusted for BMI	0.0406	0.0327	0.2818
**ebi-a-GCST90007321**	acute RITs	circulating leptin levels adjusted for BMI	0.0184	0.0181	0.3430
**ebi-a-GCST90007322**	acute RITs	circulating leptin levels adjusted for BMI	-0.0094	0.0155	0.5656
**ebi-a-GCST90007324**	acute RITs	circulating leptin levels adjusted for BMI	-0.0231	0.0153	0.1821
**ebi-a-GCST90007327**	acute RITs	circulating leptin levels adjusted for BMI	0.0016	0.0193	0.9389
**met-d-Clinical_LDL_C**	acute RITs	Clinical LDL cholesterol	0.0005	0.0016	0.7537
**met-d-HDL_P**	acute RITs	Concentration of HDL particles	0.0003	0.0016	0.8754
**ebi-a-GCST90092887**	acute RITs	Concentration of LDL particles	0.0031	0.0018	0.0807
**met-d-LDL_P**	acute RITs	Concentration of LDL particles	0.0029	0.0017	0.0905
**met-d-M_HDL_P**	acute RITs	Concentration of medium HDL particles	-0.0001	0.0015	0.9408
**ebi-a-GCST90092963**	acute RITs	Concentration of small LDL particles	0.0025	0.0017	0.1323
**met-d-S_LDL_P**	acute RITs	Concentration of small LDL particles	0.0026	0.0017	0.1155
**met-d-XL_VLDL_P**	acute RITs	Concentration of very large VLDL particles	0.0007	0.0015	0.6325
**met-a-744**	acute RITs	Cyclo(leu-pro)	0.0165	0.0084	0.0738
**ebi-a-GCST90060606**	acute RITs	Diacylglycerol(34:2)_[M+H-H2O]1+ levels	0.0154	0.0075	0.0571
**ebi-a-GCST90060606**	acute RITs	Diacylglycerol(34:2)_[M+H-H2O]1+ levels	0.0154	0.0075	0.0571
**ebi-a-GCST90060625**	acute RITs	Diacylglycerol(36:2)_[M+NH4]1+ levels	-0.0067	0.0049	0.1910
**ebi-a-GCST90025954**	acute RITs	Direct low density lipoprotein cholesterol levels	-0.0005	0.0010	0.6232
**met-a-586**	acute RITs	Eicosenoate (20:1n9 or 11)	-0.0164	0.0112	0.1718
**ebi-a-GCST90019407**	acute RITs	Electron transfer flavoprotein subunit alpha, mitochondrial measurement	0.0006	0.0044	0.8915
**ebi-a-GCST90012105**	acute RITs	Estradiol levels	0.0022	0.0047	0.6349
**ebi-a-GCST000571**	acute RITs	Fasting blood insulin	-0.0206	0.0101	0.0680
**ebi-a-GCST90060570**	acute RITs	Fatty acid(15:0)_[M-H]1- levels	-0.0028	0.0068	0.6897
**ebi-a-GCST90060571**	acute RITs	Fatty acid(16:1)_[M-H]1- levels	0.0063	0.0094	0.5176
**met-d-HDL_FC**	acute RITs	Free cholesterol in HDL	-0.0004	0.0013	0.7765
**met-d-IDL_FC**	acute RITs	Free cholesterol in IDL	-0.0005	0.0016	0.7308
**met-c-900**	acute RITs	Free cholesterol in medium HDL	-0.0113	0.0057	0.0614
**met-d-M_HDL_FC**	acute RITs	Free cholesterol in medium HDL	-0.0003	0.0016	0.8742
**met-d-M_LDL_FC**	acute RITs	Free cholesterol in medium LDL	0.0010	0.0017	0.5658
**met-d-M_HDL_FC_pct**	acute RITs	Free cholesterol to total lipids ratio in medium HDL	-0.0020	0.0013	0.1203

**met-d-S_HDL_FC_pct**	acute RITs	Free cholesterol to total lipids ratio in small HDL	-0.0003	0.0016	0.8506
**ebi-a-GCST90025966**	acute RITs	Gamma glutamyl transferase levels	-0.0005	0.0008	0.5047
**met-a-564**	acute RITs	Gamma-glutamylthreonine*	-0.0051	0.0111	0.6581
**ebi-a-GCST90018735**	acute RITs	Glucose levels	0.0003	0.0030	0.9255
**ebi-a-GCST005058**	acute RITs	HDL cholesterol	-0.0095	0.0062	0.1446
**ebi-a-GCST90018736**	acute RITs	HDL cholesterol	-0.0011	0.0018	0.5324
**ebi-a-GCST90018956**	acute RITs	HDL cholesterol	-0.0014	0.0008	0.0634
**ieu-b-109**	acute RITs	HDL cholesterol	-0.0012	0.0007	0.0963
**ieu-b-4843**	acute RITs	HDL cholesterol	-0.0011	0.0024	0.6378
**ieu-b-4844**	acute RITs	HDL cholesterol	-0.0011	0.0016	0.4813
**met-d-HDL_C**	acute RITs	HDL cholesterol	-0.0016	0.0013	0.2338
**ukb-e-30760_AFR**	acute RITs	HDL cholesterol	-0.0097	0.0057	0.1016
**ebi-a-GCST90025956**	acute RITs	HDL cholesterol levels	-0.0010	0.0007	0.1939
**ebi-a-GCST90002309**	acute RITs	Hemoglobin concentration	0.0011	0.0035	0.7505
**ebi-a-GCST008035**	acute RITs	High density lipoprotein cholesterol levels	-0.0055	0.0028	0.0594
**ebi-a-GCST90014007**	acute RITs	High density lipoprotein cholesterol levels (UKB data field 30760)	-0.0014	0.0008	0.0902
**bbj-a-24**	acute RITs	High-density-lipoprotein cholesterol	-0.0009	0.0019	0.6374
**ebi-a-GCST90014008**	acute RITs	IGF 1 (UKB data field 30770)	0.0001	0.0009	0.9387
**ukb-e-30770_CSA**	acute RITs	IGF-1	-0.0095	0.0068	0.1707
**ukb-e-recode1_CSA**	acute RITs	Indirect bilirubin	-0.0009	0.0057	0.8819
**met-a-323**	acute RITs	Isoleucine	-0.0161	0.0077	0.0534
**met-a-652**	acute RITs	Isovalerylcarnitine	0.0083	0.0044	0.0779
**ieu-b-110**	acute RITs	LDL cholesterol	0.0006	0.0009	0.4940
**ieu-b-5089**	acute RITs	LDL cholesterol	-0.0004	0.0014	0.7745
**met-d-LDL_C**	acute RITs	LDL cholesterol	0.0003	0.0016	0.8556
**ebi-a-GCST90025993**	acute RITs	Lipoprotein (a) levels	0.0006	0.0017	0.7009
**ebi-a-GCST90002412**	acute RITs	Low density lipoprotein cholesterol levels	-0.0009	0.0008	0.2606
**ebi-a-GCST90019485**	acute RITs	MAP/microtubule affinity-regulating kinase 3 measurement	0.0067	0.0072	0.3578
**ebi-a-GCST90019404**	acute RITs	Medium-chain specific acyl-CoA dehydrogenase, mitochondrial measurement	0.0012	0.0040	0.7671
**ebi-a-GCST90012047**	acute RITs	Myoglobin levels	0.0047	0.0070	0.5073
**met-a-477**	acute RITs	Myristoleate (14:1n5)	-0.0014	0.0089	0.8751
**met-a-576**	acute RITs	Palmitoleate (16:1n7)	0.0072	0.0113	0.5517
**met-c-919**	acute RITs	Phenylalanine	-0.0003	0.0079	0.9724
**met-a-430**	acute RITs	Phenyllactate (PLA)	0.0074	0.0067	0.2848
**ebi-a-GCST90014010**	acute RITs	Phosphate levels (UKB data field 30810)	0.0001	0.0012	0.9129
**ebi-a-GCST90060687**	acute RITs	Phosphatidate(34:0)_[M+OAc]1- levels	0.0088	0.0087	0.3404

**ebi-a-GCST90060914**	acute RITs	Phosphatidylcholine(38:7)_[M+OAc]1-/Phosphatidylserine(42:6)_[M-H]1- levels	-0.0131	0.0070	0.0844
**ebi-a-GCST90060671**	acute RITs	Phosphatidylcholine-O(32:0)_[M+H]1+/Phosphatidylethanolamine-O(35:0)_[M+H]1+ levels	0.0046	0.0092	0.6254
**ebi-a-GCST90060801**	acute RITs	Phosphatidylcholine-O(38:4)_[M+H]1+/Phosphatidylcholine-P(38:3)_[M+H]1+ levels	-0.0062	0.0041	0.1512
**ebi-a-GCST90060961**	acute RITs	Phosphatidylserine(40:5)_[M+OAc]1- levels	-0.0055	0.0052	0.2974
**met-d-XL_VLDL_PL**	acute RITs	Phospholipids in very large VLDL	0.0006	0.0015	0.6854
**met-d-L_VLDL_PL_pct**	acute RITs	Phospholipids to total lipids ratio in large VLDL	0.0025	0.0016	0.1027
**met-d-M_HDL_PL_pct**	acute RITs	Phospholipids to total lipids ratio in medium HDL	0.0022	0.0014	0.1247
**ebi-a-GCST90092977**	acute RITs	Phospholipids to total lipids ratio in small VLDL	-0.0008	0.0015	0.5707
**ieu-a-1012**	acute RITs	Plasma cortisol	-0.0061	0.0071	0.4210
**met-a-678**	acute RITs	Pro-hydroxy-pro	-0.0065	0.0082	0.4426
**met-a-574**	acute RITs	Pseudouridine	0.0001	0.0104	0.9930
**met-a-501**	acute RITs	Pyroglutamine*	-0.0132	0.0073	0.0904
**met-d-SFA_pct**	acute RITs	Ratio of saturated fatty acids to total fatty acids	-0.0057	0.0032	0.0824
**met-d-TG_by_PG**	acute RITs	Ratio of triglycerides to phosphoglycerides	0.0016	0.0014	0.2346
**met-d-Remnant_C**	acute RITs	Remnant cholesterol (non-HDL, non-LDL -cholesterol)	0.0015	0.0017	0.3756
**ebi-a-GCST005059**	acute RITs	Serum albumin level	0.0047	0.0077	0.5529
**ebi-a-GCST90018722**	acute RITs	Serum alkaline phosphatase levels	0.0010	0.0017	0.5622
**ebi-a-GCST90025991**	acute RITs	Serum urea levels	-0.0011	0.0013	0.3654
**ebi-a-GCST90019384**	acute RITs	SPARC-related modular calcium-binding protein 1 measurement	-0.0086	0.0046	0.0726
**ebi-a-GCST90060683**	acute RITs	Sphingomyelin(32:1)_[M+OAc]1- levels	0.0089	0.0069	0.2097
**ebi-a-GCST90060633**	acute RITs	Sphingomyelin(32:1)_[M-CH3]1- levels	-0.0020	0.0076	0.7932
**ebi-a-GCST90060643**	acute RITs	Sphingomyelin(34:2)_[M-CH3]1- levels	0.0053	0.0132	0.6971
**ebi-a-GCST90060776**	acute RITs	Sphingomyelin(40:1)_[M+H]1+ levels	-0.0052	0.0036	0.1634
**ebi-a-GCST90060742**	acute RITs	Sphingomyelin(40:2)_[M-CH3]1- levels	0.0010	0.0060	0.8746
**ebi-a-GCST90060814**	acute RITs	Sphingomyelin(41:0)_[M+H]1- levels	0.0181	0.0086	0.0683
**ebi-a-GCST90060835**	acute RITs	Sphingomyelin(42:2)_[M+H]1+ levels	0.0040	0.0084	0.6409
**ebi-a-GCST90060812**	acute RITs	Sphingomyelin(42:8)_[M+H]1+ levels	-0.0207	0.0110	0.0970
**met-d-Total_C**	acute RITs	Total cholesterol	-0.0006	0.0018	0.7448
**met-c-898**	acute RITs	Total cholesterol in medium HDL	-0.0028	0.0056	0.6249
**ebi-a-GCST90018974**	acute RITs	Total cholesterol levels	-0.0005	0.0009	0.6137
**ebi-a-GCST90025953**	acute RITs	Total cholesterol levels	0.0000	0.0009	0.9742
**met-d-non_HDL_C**	acute RITs	Total cholesterol minus HDL-C	0.0016	0.0018	0.3672
**met-d-Total_P**	acute RITs	Total concentration of lipoprotein particles	-0.0005	0.0018	0.7764
**met-d-Total_CE**	acute RITs	Total esterified cholesterol	-0.0009	0.0018	0.6137
**met-d-Total_FC**	acute RITs	Total free cholesterol	-0.0002	0.0017	0.9090
**met-c-957**	acute RITs	Total lipids in chylomicrons and largest VLDL particles	0.0054	0.0057	0.3544

**met-d-HDL_L**	acute RITs	Total lipids in HDL	-0.0006	0.0015	0.7021
**met-d-L_LDL_L**	acute RITs	Total lipids in large LDL	0.0008	0.0016	0.5901
**met-d-M_HDL_L**	acute RITs	Total lipids in medium HDL	0.0004	0.0016	0.7906
**met-d-S_LDL_L**	acute RITs	Total lipids in small LDL	0.0016	0.0015	0.2950
**met-d-XL_VLDL_L**	acute RITs	Total lipids in very large VLDL	0.0005	0.0015	0.7400
**ukb-e-30860_CSA**	acute RITs	Total protein	0.0158	0.0077	0.0526
**ebi-a-GCST90060736**	acute RITs	Triacylglycerol(44:1)_[M+NH4]1+ levels	-0.0026	0.0070	0.7155
**ebi-a-GCST90060999**	acute RITs	Triacylglycerol(56:3)_[M+NH4]1+ levels	0.0040	0.0099	0.6946
**ukb-e-30870_CSA**	acute RITs	Triglycerides	-0.0001	0.0057	0.9803
**met-d-L_HDL_TG_pct**	acute RITs	Triglycerides to total lipids ratio in large HDL	0.0018	0.0014	0.1897
**met-d-L_HDL_TG_pct**	acute RITs	Triglycerides to total lipids ratio in large HDL	0.0018	0.0014	0.1897
**met-d-M_HDL_TG_pct**	acute RITs	Triglycerides to total lipids ratio in medium HDL	0.0018	0.0014	0.1867
**met-d-M_HDL_TG_pct**	acute RITs	Triglycerides to total lipids ratio in medium HDL	0.0018	0.0014	0.1867
**met-d-M_LDL_TG_pct**	acute RITs	Triglycerides to total lipids ratio in medium LDL	0.0026	0.0015	0.0805
**met-d-M_LDL_TG_pct**	acute RITs	Triglycerides to total lipids ratio in medium LDL	0.0026	0.0015	0.0805
**met-d-S_HDL_TG_pct**	acute RITs	Triglycerides to total lipids ratio in small HDL	0.0013	0.0014	0.3426
**met-d-S_HDL_TG_pct**	acute RITs	Triglycerides to total lipids ratio in small HDL	0.0013	0.0014	0.3426
**ieu-a-786**	acute RITs	Urate	-0.0061	0.0116	0.6374
**met-c-940**	acute RITs	Valine	-0.0010	0.0057	0.8611
**ebi-a-GCST90019468**	acute RITs	von Willebrand factor A domain-containing protein 2 measurement	0.0096	0.0056	0.1048
**met-a-499**	acute RITs	X-11334	-0.0032	0.0065	0.6265
**met-a-520**	acute RITs	X-11470	0.0105	0.0110	0.3678
**met-a-548**	acute RITs	X-11820	-0.0133	0.0063	0.0626
**met-a-610**	acute RITs	X-12442--5,8-tetradecadienoate	0.0048	0.0089	0.6009
**met-a-701**	acute RITs	X-13671	0.0225	0.0119	0.0866
**met-a-715**	acute RITs	X-14056	0.0019	0.0092	0.8409
**ukb-b-17102**	acute RITs	External causes: Y83.6 Removal of other organ (partial) (total)	0.0223	0.0305	0.4883
**ukb-b-11247**	acute RITs	Main speciality of consultant (recoded): Cardiothoracic surgery	0.0006	0.0102	0.9505
**ukb-b-17277**	acute RITs	Operation code: anal surgery	0.0110	0.0313	0.7339
**ukb-b-6863**	acute RITs	Operation code: caesarean section / caesarian section	0.0090	0.0061	0.1491
**ukb-b-6235**	acute RITs	Operation code: cholecystectomy/gall bladder removal	0.0014	0.0018	0.4245
**ukb-b-6235**	acute RITs	Operation code: cholecystectomy/gall bladder removal	0.0014	0.0018	0.4245
**ukb-b-9263**	acute RITs	Operation code: inguinal/femoral hernia repair	0.0017	0.0035	0.6248
**ukb-b-847**	acute RITs	Operation code: reduction or fixation of bone fracture	0.0009	0.0076	0.9070
**ukb-b-13003**	acute RITs	Operation code: sinus surgery	-0.0695	0.0617	0.3233
**ukb-b-15592**	acute RITs	Operation code: varicose vein surgery	0.0036	0.0019	0.0684

**ukb-b-7609**	acute RITs	Operative procedures - main OPCS: B28.2 Partial excision of breast NEC	0.0055	0.0070	0.4414
**ukb-b-4944**	acute RITs	Operative procedures - main OPCS: W85.2 Endoscopic irrigation of knee joint	0.1589	0.1100	0.2856
**ukb-e-41200_p138_AFR**	acute RITs	Operative procedures - main OPCS4	-0.0112	0.0057	0.0630
**ukb-e-41200_p26_CSA**	acute RITs	Operative procedures - main OPCS4	-0.0064	0.0067	0.3529
**ukb-e-41200_p356_AFR**	acute RITs	Operative procedures - main OPCS4	-0.0009	0.0069	0.8952
**ukb-e-41200_p834_AFR**	acute RITs	Operative procedures - main OPCS4	-0.0014	0.0074	0.8480
**ukb-e-41200_p991_CSA**	acute RITs	Operative procedures - main OPCS4	-0.0108	0.0140	0.4661
**ukb-b-4820**	acute RITs	Operative procedures - secondary OPCS: E13.6 Puncture of maxillary antrum	0.0279	0.0931	0.7838
**ukb-b-4373**	acute RITs	Operative procedures - secondary OPCS: T85.2 Block dissection of axillary lymph nodes	0.0076	0.0087	0.3970
**ukb-b-7815**	acute RITs	Operative procedures - secondary OPCS: U05.1 Computed tomography of head	-0.0328	0.0563	0.5914
**ukb-b-13551**	acute RITs	Operative procedures - secondary OPCS: X72.1 Delivery of complex chemotherapy for neoplasm including prolonged infusional treatment at first attendance	0.0694	0.0430	0.1822
**ukb-b-13683**	acute RITs	Operative procedures - secondary OPCS: Y58.8 Other specified harvest of skin for graft	-0.1712	0.2722	0.6426
**ukb-b-9816**	acute RITs	Operative procedures - secondary OPCS: Y80.4 Intravenous anaesthetic NEC	-0.0322	0.0393	0.4394
**ukb-b-13121**	acute RITs	Operative procedures - secondary OPCS: Z27.1 Oesophagus	-0.0334	0.0210	0.1506
**ukb-b-5222**	acute RITs	Operative procedures - secondary OPCS: Z94.1 Bilateral operation	-0.0184	0.0110	0.1136
**ukb-e-Y83_CSA**	acute RITs	Y83 Surgical operation and other surgical procedures as the cause of abnormal reaction of the patient, or of later complication, without mention of misadventure at the time of the procedure	-0.0141	0.0080	0.1002
**ukb-b-13350**	acute RITs	PCT responsible for patient data: NOTTINGHAM CITY PCT	-0.0273	0.0209	0.2132
**ukb-b-6873**	acute RITs	PCT responsible for patient data: WESTERN CHESHIRE PCT	-0.1970	0.0824	0.0751
**ukb-b-1539**	acute RITs	PCT where patients GP was registered: BROXTOWE AND HUCKNALL PCT	-0.0222	0.0246	0.3796
**ukb-b-6547**	acute RITs	PCT where patients GP was registered: BURY PCT	-0.0038	0.0119	0.7540
**ukb-b-8205**	acute RITs	PCT where patients GP was registered: CAMDEN PCT	-0.0716	0.0742	0.3555
**ukb-b-2838**	acute RITs	PCT where patients GP was registered: DURHAM AND CHESTER-LE-STREET PCT	-0.0106	0.0288	0.7165
**ukb-b-19050**	acute RITs	PCT where patients GP was registered: EAST LANCASHIRE TEACHING PCT	0.1932	0.2078	0.4211
**ukb-b-14654**	acute RITs	PCT where patients GP was registered: ROCHDALE PCT	-0.0305	0.0515	0.5702
**ukb-b-8842**	acute RITs	PCT where patients GP was registered: SOLIHULL PCT	-0.0002	0.0430	0.9973
**ukb-b-7288**	acute RITs	3mm asymmetry angle (left)	-0.0060	0.0063	0.3514
**ukb-e-5108_CSA**	acute RITs	3mm asymmetry angle (right)	0.0032	0.0075	0.6751
**ukb-e-5156_AFR**	acute RITs	3mm asymmetry index (left)	0.0007	0.0050	0.8927
**ukb-e-5107_AFR**	acute RITs	3mm strong meridian angle (right)	-0.0029	0.0108	0.7918
**ukb-b-4874**	acute RITs	6mm asymmetry angle (right)	-0.0065	0.0073	0.3813
**ukb-e-5306_CSA**	acute RITs	6mm index of best keratometry results (left)	-0.0009	0.0058	0.8837
**ieu-a-1095**	acute RITs	Age at menarche	0.0021	0.0028	0.4588
**ukb-d-4700_irnt**	acute RITs	Age cataract diagnosed	-0.0163	0.0104	0.1381
**ukb-b-1061**	acute RITs	Age high blood pressure diagnosed	0.0031	0.0055	0.5678

**ukb-e-2217_AFR**	acute RITs	Age started wearing glasses or contact lenses	-0.0093	0.0064	0.1571
**ukb-b-5090**	acute RITs	Astigmatism angle (left)	-0.0036	0.0077	0.6453
**ukb-e-B96_CSA**	acute RITs	B96 Other bacterial agents as the cause of diseases classified to other chapters	-0.0030	0.0088	0.7340
**ukb-e-1677_CSA**	acute RITs	Breastfed as a baby	0.0028	0.0083	0.7483
**ukb-e-1468_p1_CSA**	acute RITs	Cereal type	0.0059	0.0061	0.3437
**ukb-e-24014_AFR**	acute RITs	Close to major road	0.0081	0.0103	0.4443
**ukb-e-5086_AFR**	acute RITs	Cylindrical power (left)	0.0062	0.0074	0.4191
**ukb-b-7196**	acute RITs	Delivery methods: Elective caesarean section	-0.0025	0.1217	0.9855
**ukb-e-894_AFR**	acute RITs	Duration of moderate activity	0.0082	0.0093	0.3910
**ukb-e-874_p1_AFR**	acute RITs	Duration of walks	0.0005	0.0043	0.9161
**ukb-e-404_CSA**	acute RITs	Duration to first press of snap-button in each round	0.0042	0.0073	0.5665
**ukb-d-20411_0**	acute RITs	Ever been injured or injured someone else through drinking alcohol: No	0.0009	0.0083	0.9189
**ukb-e-2296_CSA**	acute RITs	Falls in the last year	-0.0040	0.0073	0.5941
**ukb-e-4935_CSA**	acute RITs	FI1 : numeric addition test	0.0034	0.0078	0.6685
**ukb-d-4979**	acute RITs	FI5 : family relationship calculation	-0.0015	0.0075	0.8479
**ukb-b-17738**	acute RITs	Fractured bone site(s): Other bones	-0.0025	0.0069	0.7184
**ukb-a-439**	acute RITs	Fractured bone site(s): Wrist	-0.0086	0.0072	0.2446
**ukb-b-9571**	acute RITs	Fractured bone site(s): Wrist	-0.0040	0.0064	0.5418
**ebi-a-GCST90013913**	acute RITs	Fractured bones in last 5 years (UKB data field 2463) (Firth correction)	-0.0084	0.0043	0.0562
**ukb-a-2**	acute RITs	Frequency of light DIY in last 4 weeks	0.0103	0.0091	0.2653
**ukb-e-4570_AFR**	acute RITs	Friendships satisfaction	-0.0118	0.0060	0.0635
**ebi-a-GCST90019399**	acute RITs	Gelsolin measurement	0.0017	0.0101	0.8723
**ukb-a-340**	acute RITs	Hearing aid user	0.0156	0.0078	0.0578
**ukb-b-19060**	acute RITs	Hearing aid user	0.0027	0.0069	0.7013
**ukb-e-4849_p2_AFR**	acute RITs	Hearing test done	0.0055	0.0037	0.1449
**ukb-e-6141_p2_AFR**	acute RITs	How are people in household related to participant	-0.0011	0.0058	0.8566
**ukb-e-6145_p2_AFR**	acute RITs	Illness, injury, bereavement, stress in last 2 years	-0.0002	0.0046	0.9735
**ukb-e-6145_p4_CSA**	acute RITs	Illness, injury, bereavement, stress in last 2 years	-0.0045	0.0094	0.6387
**ukb-b-14449**	acute RITs	Index of best refractometry result (left)	0.0085	0.0095	0.3836
**ukb-e-41244_p2_CSA**	acute RITs	Intended management of patient (recoded)	0.0012	0.0056	0.8363
**ukb-b-16311**	acute RITs	Interval between previous point and current one in alphanumeric path (trail #2)	-0.0030	0.0049	0.5422
**ebi-a-GCST90013453**	acute RITs	Lack of behavioral control	-0.0098	0.0156	0.5581
**ukb-e-4728_CSA**	acute RITs	Leg pain on walking	-0.0034	0.0059	0.5690
**ukb-e-6160_p5_AFR**	acute RITs	Leisure/social activities	0.0001	0.0067	0.9921
**ukb-e-41245_p20_CSA**	acute RITs	Main speciality of consultant (recoded)	0.0124	0.0128	0.3579
**ukb-e-41245_p6_AFR**	acute RITs	Main speciality of consultant (recoded)	0.0020	0.0046	0.6651

**ukb-a-392**	acute RITs	Maximum workload during fitness test	0.0017	0.0086	0.8447
**ebi-a-GCST90002397**	acute RITs	Mean spheric corpuscular volume	0.0005	0.0007	0.4869
**ukb-e-41249_p4_AFR**	acute RITs	Methods of admission to hospital (recoded)	0.0067	0.0065	0.3164
**ukb-b-5477**	acute RITs	Methods of admission to hospital (recoded): Elective admission	0.0024	0.0025	0.3509
**ukb-b-10655**	acute RITs	Methods of admission to hospital (recoded): Emergency admission	0.0012	0.0060	0.8412
**ukb-b-4540**	acute RITs	Methods of admission to hospital (recoded): Emergency admission: Non-injury	-0.0015	0.0038	0.7047
**ukb-b-19790**	acute RITs	Methods of admission to hospital (recoded): Maternity admission: Post-partum	0.0067	0.0088	0.4578
**ukb-e-41250_p1_AFR**	acute RITs	Methods of discharge from hospital (recoded)	-0.0085	0.0042	0.0563
**ukb-e-52_p9_CSA**	acute RITs	Month of birth	0.0201	0.0212	0.4140
**ukb-a-12**	acute RITs	Nap during day	-0.0017	0.0027	0.5260
**ukb-d-2654_8**	acute RITs	Non-butter spread type details: Other low or reduced fat spread	-0.0120	0.0102	0.2555
**ukb-d-2654_9**	acute RITs	Non-butter spread type details: Other type of spread/margarine	-0.0193	0.0232	0.4323
**ukb-d-2654_4**	acute RITs	Non-butter spread type details: Soft (tub) margarine	0.0004	0.0070	0.9553
**ukb-e-4291_CSA**	acute RITs	Number of attempts	0.0044	0.0109	0.7002
**ieu-b-4827**	acute RITs	Number of children ever born measurement	-0.0063	0.0088	0.4827
**ukb-b-2988**	acute RITs	Number of fluid intelligence questions attempted within time limit	-0.0045	0.0040	0.2646
**ukb-e-399_CSA**	acute RITs	Number of incorrect matches in round	-0.0191	0.0087	0.0559
**ukb-b-1209**	acute RITs	Number of live births	-0.0020	0.0060	0.7425
**ukb-b-6891**	acute RITs	Number of triplets attempted (left)	-0.0055	0.0102	0.6009
**ukb-b-4909**	acute RITs	Number of triplets attempted (right)	0.0147	0.0087	0.1034
**ukb-e-4276_AFR**	acute RITs	Number of triplets attempted (right)	0.0006	0.0280	0.9853
**ebi-a-GCST90012790**	acute RITs	Percentage of invited food questionnaires completed	0.0030	0.0047	0.5170
**ieu-b-4860**	acute RITs	Physical activity	0.0154	0.0104	0.1547
**ebi-a-GCST90061410**	acute RITs	Physical activity (Total log acceleration 2am-4am)	-0.0046	0.0056	0.4188
**ukb-e-20018_AFR**	acute RITs	Prospective memory result	0.0112	0.0149	0.4694
**ukb-a-421**	acute RITs	Reason for glasses/contact lenses: For just reading/near work as you are getting older (called 'presbyopia')	-0.0021	0.0060	0.7237
**ukb-a-420**	acute RITs	Reason for glasses/contact lenses: For long-sightedness i.e. for distance and near but particularly for near tasks like reading (called 'hypermetropia')	-0.0109	0.0063	0.0935
**ukb-d-2664_5**	acute RITs	Reason for reducing amount of alcohol drunk: Other reason	0.0083	0.0055	0.1440
**ukb-e-20019_AFR**	acute RITs	Speech-reception-threshold (SRT) estimate (left)	0.0071	0.0065	0.2942
**ukb-a-343**	acute RITs	Time from waking to first cigarette	0.0126	0.0075	0.1046
**ukb-b-2732**	acute RITs	Time from waking to first cigarette	0.0001	0.0081	0.9886
**ukb-a-7**	acute RITs	Time spent driving	0.0118	0.0062	0.0623
**ukb-d-20531**	acute RITs	Victim of sexual assault	0.0039	0.0099	0.6982
**ukb-b-3709**	acute RITs	Wants to stop smoking	-0.0172	0.0102	0.1112
**ukb-e-3659_CSA**	acute RITs	Year immigrated to UK (United Kingdom)	0.0011	0.0079	0.8869
**ukb-e-Z53_AFR**	acute RITs	Z53 Persons encountering health services for specifie procedures, not carried out	0.0126	0.0063	0.0658

**ukb-e-23119_CSA**	acute RITs	Arm fat percentage (right)	-0.0015	0.0079	0.8525
**ukb-e-23121_AFR**	acute RITs	Arm fat-free mass (right)	-0.0018	0.0043	0.6780
**ukb-b-8909**	acute RITs	Body fat percentage	0.0001	0.0012	0.9115
**ukb-e-23099_AFR**	acute RITs	Body fat percentage	0.0006	0.0058	0.9235
**ukb-e-23104_AFR**	acute RITs	Body mass index (BMI)	-0.0013	0.0052	0.8001
**ebi-a-GCST90092504**	acute RITs	Carotid Intima-media thickness (mean of the maximum cIMT)	-0.0186	0.0092	0.0559
**ebi-a-GCST008029**	acute RITs	Diastolic blood pressure	-0.0045	0.0051	0.3837
**ieu-b-106**	acute RITs	FEV1/FVC < 0.7	0.0003	0.0019	0.8563
**ieu-b-4853**	acute RITs	Forced expiratory volume in 1-second	0.0403	0.0173	0.0594
**ukb-e-46_AFR**	acute RITs	Hand grip strength (left)	-0.0082	0.0077	0.2998
**ukb-b-8875**	acute RITs	Heel bone mineral density (BMD)	-0.0001	0.0009	0.9304
**ukb-a-500**	acute RITs	Heel bone mineral density (BMD) T-score automated	0.0006	0.0011	0.6038
**ukb-a-500**	acute RITs	Heel bone mineral density (BMD) T-score automated	0.0006	0.0011	0.6038
**ukb-a-361**	acute RITs	Heel bone mineral density (BMD) T-score automated (left)	0.0005	0.0014	0.7359
**ukb-a-361**	acute RITs	Heel bone mineral density (BMD) T-score automated (left)	0.0005	0.0014	0.7359
**ukb-a-362**	acute RITs	Heel bone mineral density (BMD) T-score automated (right)	0.0012	0.0014	0.3664
**ukb-a-362**	acute RITs	Heel bone mineral density (BMD) T-score automated (right)	0.0012	0.0014	0.3664
**ukb-b-20124**	acute RITs	Heel bone mineral density (BMD) T-score, automated	0.0001	0.0009	0.9229
**ukb-b-11364**	acute RITs	Heel bone mineral density (BMD), manual entry	-0.0005	0.0024	0.8263
**ukb-b-17612**	acute RITs	Heel bone ultrasound T-score, manual entry	0.0034	0.0032	0.2887
**ukb-b-17612**	acute RITs	Heel bone ultrasound T-score, manual entry	0.0034	0.0032	0.2887
**ukb-b-17952**	acute RITs	Heel Broadband ultrasound attenuation (BUA), manual entry	-0.0013	0.0031	0.6879
**ukb-b-17952**	acute RITs	Heel Broadband ultrasound attenuation (BUA), manual entry	-0.0013	0.0031	0.6879
**ukb-b-5447**	acute RITs	Heel broadband ultrasound attenuation (left)	0.0010	0.0013	0.4324
**ukb-b-5447**	acute RITs	Heel broadband ultrasound attenuation (left)	0.0010	0.0013	0.4324
**ukb-b-6027**	acute RITs	Heel broadband ultrasound attenuation (right)	0.0024	0.0012	0.0562
**ukb-b-6027**	acute RITs	Heel broadband ultrasound attenuation (right)	0.0024	0.0012	0.0562
**ukb-b-6027**	acute RITs	Heel broadband ultrasound attenuation (right)	0.0024	0.0012	0.0562
**ukb-b-15851**	acute RITs	Heel Broadband ultrasound attenuation, direct entry	-0.0001	0.0010	0.9270
**ukb-b-19234**	acute RITs	Heel quantitative ultrasound index (QUI), direct entry	0.0001	0.0009	0.9235
**ukb-b-19234**	acute RITs	Heel quantitative ultrasound index (QUI), direct entry	0.0001	0.0009	0.9235
**ukb-b-17848**	acute RITs	Heel quantitative ultrasound index (QUI), manual entry	-0.0015	0.0025	0.5458
**ukb-e-49_AFR**	acute RITs	Hip circumference	0.0125	0.0107	0.2602
**ukb-e-23110_AFR**	acute RITs	Impedance of arm (left)	0.0059	0.0066	0.3811
**ebi-a-GCST90020194**	acute RITs	Inferior Posterior lobe of cerebellar volume (including Crus II to IX hemispheric lobules)	0.0045	0.0071	0.5280
**ukb-b-19277**	acute RITs	Intra-ocular pressure, corneal-compensated (right)	0.0016	0.0026	0.5577

**bbj-a-37**	acute RITs	Mean arterial pressure	0.0011	0.0042	0.7942
**ebi-a-GCST90018743**	acute RITs	Mean arterial pressure	-0.0064	0.0036	0.0745
**ukb-e-MAP_p2_AFR**	acute RITs	Mean arterial pressure, automated reading, adjusted by medication	0.0047	0.0066	0.4847
**ukb-e-MAP_p4_AFR**	acute RITs	Mean arterial pressure, combined automated + manual reading, adjusted by medication	0.0045	0.0068	0.5203
**ukb-d-30050_irnt**	acute RITs	Mean corpuscular haemoglobin	-0.0016	0.0009	0.0710
**ebi-a-GCST004630**	acute RITs	Mean corpuscular hemoglobin	-0.0003	0.0011	0.7895
**ebi-a-GCST90002390**	acute RITs	Mean corpuscular hemoglobin	-0.0005	0.0008	0.5175
**ebi-a-GCST90002391**	acute RITs	Mean corpuscular hemoglobin concentration	-0.0002	0.0011	0.8341
**ebi-a-GCST90002391**	acute RITs	Mean corpuscular hemoglobin concentration	-0.0002	0.0011	0.8341
**ebi-a-GCST90025962**	acute RITs	Mean corpuscular hemoglobin concentration	0.0004	0.0007	0.5770
**ebi-a-GCST004602**	acute RITs	Mean corpuscular volume	-0.0001	0.0011	0.9556
**ebi-a-GCST90002392**	acute RITs	Mean corpuscular volume	0.0000	0.0007	0.9996
**ebi-a-GCST90025963**	acute RITs	Mean corpuscular volume	-0.0007	0.0007	0.3450
**ukb-d-30040_irnt**	acute RITs	Mean corpuscular volume	-0.0003	0.0008	0.7096
**ebi-a-GCST90013979**	acute RITs	Mean corpuscular volume (UKB data field 30040)	-0.0011	0.0008	0.1851
**ukb-a-251**	acute RITs	Overall health rating	0.0031	0.0030	0.2984
**ieu-a-274**	acute RITs	Packed cell volume	0.0001	0.0044	0.9845
**ebi-a-GCST90000065**	acute RITs	Pulse pressure	-0.0009	0.0014	0.5210
**ukb-e-PP_p1_CSA**	acute RITs	Pulse pressure, automated reading	-0.0018	0.0102	0.8655
**ukb-e-R19_CSA**	acute RITs	R19 Other symptoms and signs involving the digestive system and abdomen	-0.0009	0.0099	0.9275
**ebi-a-GCST90020192**	acute RITs	Superior Posterior lobe of cerebellar volume (including VI to Crus I hemispheric lobules)	-0.0113	0.0059	0.0576
**ebi-a-GCST005349**	acute RITs	Total body bone mineral density (age over 60)	0.0010	0.0032	0.7591
**ebi-a-GCST90020190**	acute RITs	Total cerebellar volume (excluding Crus I vermis)	-0.0049	0.0083	0.5514
**ukb-e-23128_AFR**	acute RITs	Trunk fat mass	-0.0060	0.0065	0.3663
**ukb-e-23127_AFR**	acute RITs	Trunk fat percentage	-0.0021	0.0053	0.6942
**ebi-a-GCST90002228**	acute RITs	Two-hour glucose	0.0007	0.0095	0.9428
**ebi-a-GCST90013422**	acute RITs	Ultradistal forearm bone mineral density	0.0079	0.0045	0.0865
**ieu-a-61**	acute RITs	Waist circumference	0.0001	0.0034	0.9667
**ieu-a-72**	acute RITs	Waist-to-hip ratio	0.0009	0.0045	0.8401
**ebi-a-GCST90095035**	acute RITs	Waist-to-hip ratio adjusted for BMI	-0.0173	0.0091	0.0666
**ukb-d-5610_3**	acute RITs	Which eye(s) affected by presbyopia: Both eyes	0.0039	0.0091	0.6739
**ukb-e-Z09_CSA**	acute RITs	Z09 Follow-up examination after treatment for conditions other than malignant neoplasms	-0.0040	0.0077	0.6144
**ukb-e-Z12_CSA**	acute RITs	Z12 Special screening examination for neoplasms	0.0099	0.0104	0.3622
**ukb-b-5779**	acute RITs	Alcohol intake frequency.	0.0022	0.0016	0.1571
**ukb-a-32**	acute RITs	Alcohol intake versus 10 years previously	0.0094	0.0051	0.0728
**ukb-e-1628_CSA**	acute RITs	Alcohol intake versus 10 years previously	0.0039	0.0079	0.6274

**ieu-b-4825**	acute RITs	Cigarettes smoked per day	-0.0083	0.0083	0.3313
**ukb-b-1572**	acute RITs	Difficulty not smoking for 1 day	-0.0056	0.0067	0.4112
**ukb-d-20541**	acute RITs	Difficulty stopping worrying during worst period of anxiety	0.0162	0.0091	0.0959
**ukb-d-20425**	acute RITs	Ever worried more than most people would in similar situation	0.0027	0.0083	0.7436
**ebi-a-GCST006944**	acute RITs	Experiencing mood swings	0.0032	0.0036	0.3795
**ieu-b-117**	acute RITs	HOMA-B	-0.0113	0.0113	0.3328
**ukb-a-47**	acute RITs	Irritability	-0.0022	0.0044	0.6080
**ebi-a-GCST90013875**	acute RITs	Irritability (UKB data field 1940) (Firth correction)	-0.0002	0.0036	0.9639
**ebi-a-GCST90013925**	acute RITs	Irritability (UKB data field 1940) (SPA correction)	-0.0002	0.0036	0.9543
**ebi-a-GCST90013873**	acute RITs	Mood swings (UKB data field 1920) (Firth correction)	-0.0027	0.0033	0.4171
**ebi-a-GCST90013923**	acute RITs	Mood swings (UKB data field 1920) (SPA correction)	-0.0027	0.0033	0.4178
**ukb-a-342**	acute RITs	Number of cigarettes currently smoked daily (current cigarette smokers)	-0.0042	0.0074	0.5812
**ukb-a-238**	acute RITs	Pack years adult smoking as proportion of life span exposed to smoking PREVIEW ONLY	-0.0055	0.0037	0.1458
**ukb-a-237**	acute RITs	Pack years of smoking PREVIEW ONLY	-0.0032	0.0037	0.3954
**ieu-a-1046**	acute RITs	Pallidum volume	-0.0227	0.0119	0.0861
**ebi-a-GCST008027**	acute RITs	Smoking behavior (cigarettes smoked per day)	0.0024	0.0054	0.6645
**ukb-e-2010_AFR**	acute RITs	Suffer from 'nerves'	0.0044	0.0134	0.7474
**ebi-a-GCST90025968**	acute RITs	Systolic blood pressure	0.0000	0.0012	0.9718
**ieu-b-38**	acute RITs	systolic blood pressure	-0.0010	0.0010	0.3260
**ebi-a-GCST90014018**	acute RITs	Systolic blood pressure automated reading (UKB data field 4080)	-0.0018	0.0015	0.2485
**ukb-e-SBP_p1_AFR**	acute RITs	Systolic blood pressure, automated reading, adjusted by medication	0.0047	0.0054	0.3925
**ukb-e-SBP_p2_AFR**	acute RITs	Systolic blood pressure, combined automated + manual reading	-0.0009	0.0074	0.9103
**ukb-e-SBP_p3_AFR**	acute RITs	Systolic blood pressure, combined automated + manual reading, adjusted by medication	0.0080	0.0066	0.2439
**ukb-e-6164_p6_CSA**	acute RITs	Types of physical activity in last 4 weeks	-0.0013	0.0124	0.9180
**ukb-d-22612_2**	acute RITs	Worked with materials containing asbestos: Often	0.0071	0.0083	0.4080
**ukb-d-22609_0**	acute RITs	Workplace very dusty: Rarely/never	0.0117	0.0102	0.2668
**ieu-a-1010**	acute RITs	Years of schooling	-0.0044	0.0050	0.3841
**ukb-e-Z88_AFR**	acute RITs	Z88 Personal history of allergy to drugs, medicaments and biologocal substances	-0.0109	0.0073	0.1559

Table S5 Multivariable Mendelian randomization of biologically related traits

**Exposure**	**SNPn**	***β***	**Se**	**lo_ci**	**up_ci**	***P* value**	**OR**	**OR_lci95**	**OR_uci95**	**Group**
**Systolic blood pressure**	313	0.009	0.036	-0.022	0.037	0.590	1.020	0.950	1.090	Blood Pressure
**Diastolic blood pressure**	1	NA	NA	NA	NA	NA	NA	NA	NA	Blood Pressure
**Systemic lupus erythematosus**	13	-0.013	0.010	-0.018	-0.004	0.010	0.970	0.960	0.990	Autoimmune disease
**Inflammatory bowel disease**	99	0.000	0.000	-0.009	0.009	0.970	1.000	0.980	1.020	Autoimmune disease

**Crohn's disease**	32	0.000	0.000	-0.009	0.009	0.840	1.000	0.980	1.020	Autoimmune disease
**Ulcerative colitis**	56	0.000	0.000	-0.009	0.009	0.960	1.000	0.980	1.020	Autoimmune disease
**Irritability (UKB data field 1940) (SPA correction)**	28	0.045	0.040	0.013	0.076	0.010	1.110	1.030	1.190	Mental Problems
**Experiencing mood swings**	34	-0.013	0.076	-0.102	0.057	0.690	0.970	0.790	1.140	Mental Problems
**Seen doctor (GP) for nerves anxiety tension or depression**	13	-0.013	0.220	-0.310	0.161	0.890	0.970	0.490	1.450	Mental Problems
**Low density lipoprotein cholesterol levels**	125	0.004	0.020	-0.013	0.017	0.660	1.010	0.970	1.040	Cholesterol Levels
**High density lipoprotein cholesterol levels (UKB data field 30760)**	106	0.029	0.030	0.000	0.053	0.040	1.070	1.000	1.130	Cholesterol Levels
**Total cholesterol levels**	97	-0.004	0.029	-0.022	0.017	0.730	0.990	0.950	1.040	Cholesterol Levels

Table S6 Associations of lipoprotein or cholesterol levels with acute radiation-induced toxicities

**Exposure ID**	**Trait name**	**Population**	**Method**	**b**	**Se**	**P value**	**OR**	**OR_lci95**	**OR_uci95**
**ukb-b-8909**	Body fat percentage	European	IVW	0.071	0.034	0.036	1.074	1.005	1.148
**ebi-a-GCST90002412**	Low density lipoprotein cholesterol levels	European	IVW	0.057	0.02	0.004	1.058	1.018	1.1
**ebi-a-GCST90014007**	High density lipoprotein cholesterol levels (UKB data field 30760)	European	IVW	0.055	0.022	0.013	1.057	1.012	1.103
**ebi-a-GCST90025953**	Total cholesterol levels	European	IVW	0.055	0.023	0.018	1.057	1.01	1.106
**ebi-a-GCST90092809**	Apolipoprotein B levels	European	IVW	0.061	0.025	0.014	1.063	1.012	1.116
**ebi-a-GCST90092887**	Concentration of LDL particles	European	IVW	0.067	0.025	0.008	1.069	1.018	1.123
**ieu-b-110**	LDL cholesterol	European	IVW	0.066	0.024	0.006	1.068	1.019	1.119
**ieu-b-4844**	HDL cholesterol	European	IVW	0.052	0.02	0.009	1.053	1.013	1.095
**met-d-HDL_C**	HDL cholesterol	European	IVW	0.051	0.022	0.019	1.052	1.008	1.098
**met-d-HDL_P**	Concentration of HDL particles	European	IVW	0.066	0.025	0.009	1.068	1.017	1.122
**met-d-LDL_P**	Concentration of LDL particles	European	IVW	0.061	0.025	0.014	1.063	1.012	1.116
**met-d-M_HDL_C**	Cholesterol in medium HDL	European	IVW	0.062	0.025	0.012	1.064	1.014	1.117
**met-d-M_HDL_P**	Concentration of medium HDL particles	European	IVW	0.055	0.025	0.026	1.056	1.007	1.108
**met-d-M_HDL_PL_pct**	Phospholipids to total lipids ratio in medium HDL	European	IVW	-0.063	0.023	0.006	0.939	0.898	0.982
**ebi-a-GCST90060571**	Fatty acid(16:1)_[M-H]1- levels	South Asian	IVW	-0.104	0.035	0.003	0.902	0.842	0.965
**ebi-a-GCST90060625**	Diacylglycerol(36:2)_[M+NH4]1+ levels	South Asian	IVW	-0.134	0.047	0.004	0.874	0.798	0.958
**ebi-a-GCST90060634**	Cholesteryl ester(18:3)_[M+NH4]1+ levels	South Asian	IVW	0.125	0.047	0.008	1.133	1.033	1.242
**ebi-a-GCST90060671**	Phosphatidylcholine-O(32:0)_[M+H]1+/Phosphatidylethanolamine-O(35:0)_[M+H]1+ levels	South Asian	IVW	0.141	0.059	0.017	1.151	1.026	1.292
**ebi-a-GCST90060999**	Triacylglycerol(56:3)_[M+NH4]1+ levels	South Asian	IVW	-0.125	0.039	0.001	0.882	0.817	0.953

Table S7 Associations of circulating leptin levels with acute radiation-induced toxicities

Exposure ID	Trait name	Population	Method	b	Se	P value	OR	OR_lci95	OR_uci95
**ebi-a-GCST90007322**	Circulating leptin levels adjusted for BMI	European	IVW	-0.18	0.069	0.009	0.835	0.73	0.956
**ebi-a-GCST90007319**	Circulating leptin levels adjusted for BMI	Mixed	IVW	-0.173	0.065	0.008	0.841	0.74	0.955
**ebi-a-GCST90007309**	Circulating leptin levels	Mixed	IVW	-0.179	0.07	0.01	0.836	0.729	0.959
**ebi-a-GCST90007313**	Circulating leptin levels	Mixed	IVW	-0.141	0.056	0.012	0.869	0.779	0.969
**ebi-a-GCST90007307**	Circulating leptin levels	Mixed	IVW	-0.156	0.059	0.008	0.856	0.762	0.961
